# The impacts of biological invasions

**DOI:** 10.1002/brv.70124

**Published:** 2025-12-30

**Authors:** Phillip J. Haubrock, Teun Everts, Neil Angelo S. Abreo, Jamie Bojko, Victor Deklerck, James W. E. Dickey, Ana Clara S. Franco, Emili García‐Berthou, Stelios Katsanevakis, Natalia I. Kirichenko, Stefano Mammola, Martin A. Nuñez, Ben Parker, Riccardo Scalera, Ismael Soto, Diederik Strubbe, Ali Serhan Tarkan, Lorenzo Vilizzi, Tim Adriaens, Paride Balzani, Dagmara Błońska, Elizabeta Briski, Rein Brys, Amy L. Burgess, James E. Byers, Carlos Cano‐Barbacil, Giuseppe Castaldelli, Jaimie T.A. Dick, Victoria Dominguez Almela, Romina D. Dimarco, Margarita Florencio, Antonín Kouba, Melina Kourantidou, Irmak Kurtul, Irene Martín‐Forés, Olivier Morissette, Julian D. Olden, Bruno E. Soares, Jakub Truszkowski, Hugo Verreycken, Marc Kenis, Ronaldo Sousa, J. Robert Britton

**Affiliations:** ^1^ Department of Life and Environmental Sciences, Faculty of Science and Technology Bournemouth University Talbot Campus, Fern Barrow Poole BH12 5BB UK; ^2^ South Bohemian Research Center of Aquaculture and Biodiversity of Hydrocenoses, Faculty of Fisheries and Protection of Waters University of South Bohemia in České Budějovice Zátiší 728/II Vodňany 389 01 Czech Republic; ^3^ Genetic Diversity Research Institute for Nature and Forest Gaverstraat 4 Geraardsbergen 9500 Belgium; ^4^ Biology Department KU Leuven Kasteelpark Arenberg 31 Heverlee 3001 Belgium; ^5^ Mapua Malayan Colleges Mindanao, Tulip Drive, Bajada Davao City 8000 Philippines; ^6^ National Horizons Centre Teesside University 14 John Dixon Lane Darlington DL1 1HG UK; ^7^ Meise Botanic Garden Nieuwelaan 38 Meise 1860 Belgium; ^8^ National Laboratory for Health Security, HUN‐REN Centre for Ecological Research Karolina út 29 Budapest 1113 Hungary; ^9^ GRECO Institute of Aquatic Ecology, University of Girona Campus Montilivi Girona 17003 Catalonia Spain; ^10^ Department of Marine Sciences University of the Aegean Mytilene 81100 Lesvos Greece; ^11^ Sukachev Institute of Forest, Siberian Branch of the Russian Academy of Sciences, Federal Research Center “Krasnoyarsk Science Center SB RAS” Akademgorodok 50/28 Krasnoyarsk 660036 Russia; ^12^ Institute of Ecology and Geography, Siberian Federal University Svobodny Prospekt 79 Krasnoyarsk 660041 Russia; ^13^ Slovenian Forestry Institute Večna pot 2 Ljubljana 1000 Slovenia; ^14^ Molecular Ecology Group (MEG) Water Research Institute (IRSA), National Research Council of Italy (CNR) Corso Tonolli 50 Verbania 28922 Italy; ^15^ Finnish Museum of Natural History University of Helsinki Pohjoinen Rautatiekatu 13 Helsinki 00100 Finland; ^16^ NBFC, National Biodiversity Future Center Via Ammiraglio Rizzo 5 Palermo 90142 Italy; ^17^ Department of Biology and Biochemistry University of Houston, Science & Research Building 2 3455 Cullen Blvd Houston 77204 TX USA; ^18^ Department of Biosciences, Faculty of Health and Life Sciences University of Exeter Stocker Road Exeter EX4 4QD UK; ^19^ Department of Science Roma Tre University Viale Guglielmo Marconi 446 Rome 00146 Italy; ^20^ Centre for Research on Ecology, Cognition and Behaviour of Birds (ECoBird) K.L. Ledeganckstraat 35 Ghent 9000 Belgium; ^21^ Faculty of Biology and Environmental Protection, Department of Ecology and Vertebrate Zoology University of Lodz ul. Banacha 12/16 Łódź 90‐237 Poland; ^22^ Department of Basic Sciences, Faculty of Fisheries Muğla Sıtkı Koçman University Kötekli Mahallesi Muğla 48000 Türkiye; ^23^ InvasionsLab, Research Institute for Nature and Forest (INBO) Havenlaan 88, bus 73 Brussels 1000 Belgium; ^24^ GEOMAR Helmholtz Centre for Ocean Research Kiel Wischhofstrasse 1‐3 Kiel 24148 Germany; ^25^ Odum School of Ecology University of Georgia 140 E Green Street Athens 30602 GA USA; ^26^ Departamento de Biodiversidad y Biología Evolutiva Museo Nacional de Ciencias Naturales, CSIC Calle José Gutiérrez Abascal 2 Madrid 28006 Spain; ^27^ Department of Environmental and Prevention Sciences University of Ferrara Via Luigi Borsari 46 Ferrara 44121 Italy; ^28^ School of Biological Sciences Queen's University Belfast 97 Lisburn Road Belfast BT9 7BL UK; ^29^ School of Geography and Environmental Sciences, University of Southampton University Road Southampton SO17 1BJ UK; ^30^ Estación Biológica de Doñana, CSIC Avenida Américo Vespucio 26 Sevilla 41092 Spain; ^31^ CNRS, Univ Brest, Ifremer, IRD, EMR 6004, AMURE, IUEM Plouzané 29280 France; ^32^ Department of Business and Sustainability University of Southern Denmark Degnevej 14 Esbjerg 6705 Denmark; ^33^ Marine and Inland Waters Sciences and Technology Ege University Bornova İzmir 35100 Türkiye; ^34^ The University of Adelaide South Australia, North Terrace Campus Adelaide 5005 SA Australia; ^35^ TERN, School of Biological Sciences The University of Adelaide Benham Building, North Terrace Adelaide 5005 SA Australia; ^36^ Université du Québec à Chicoutimi 555 Boulevard de l'Université, Chicoutimi QC G7H 2B1 Canada; ^37^ School of Aquatic and Fishery Sciences University of Washington 1122 NE Boat Street Seattle 98105 WA USA; ^38^ University of Regina Saskatchewan, 3737 Wascana Parkway Regina S4S 0A2 SK Canada; ^39^ Chalmers University of Technology Chalmersplatsen 4 Göteborg 412 96 Sweden; ^40^ CABI Rue des Grillons 1 Delémont 2800 Switzerland; ^41^ CBMA – Centre for Molecular and Environmental Biology/ARNET‐Aquatic Research Network/ IB‐S, Institute of Science and Innovation for Bio‐Sustainability, Department of Biology University of Minho Campus de Gualtar Braga 4710‐057 Portugal

**Keywords:** biological invasions, invasion impacts, ecological effects, impact assessment, risk analysis

## Abstract

The Anthropocene is characterised by a continuous human‐mediated reshuffling of the distributions of species globally. Both intentional and unintentional introductions have resulted in numerous species being translocated beyond their native ranges, often leading to their establishment and subsequent spread – a process referred to as biological invasion. Biological invasions are associated with profound changes in the composition, structure, and functioning of recipient ecosystems, plus substantial financial losses and disruptions to society, culture, and human well‐being. These ecological, economic, and socio‐cultural impacts are interrelated, ubiquitous, and detrimental, yet they are often subjectively perceived or inaccurately quantified. Persistent knowledge gaps remain, however, which limit our understanding of the complex and multifaceted causes and mechanisms of invasion impacts. To overcome these gaps and comprehensively capture all related facets pertaining to the nature and diversity of invasion impact, this scoping review of academic studies, grey literature, and expert reports provides a conceptual model for interpreting invasion impacts, structured around three interrelated pillars: impact domains, challenges in the study of impacts, and available risk‐ and impact assessments. We initially explore the various mechanisms and consequences of ecological, economic, and socio‐cultural invasion impacts and their temporal dynamics, substantiating these with relevant empirical examples. We then review common challenges and fallacies in studying invasion impacts, including context specificity and inter‐comparability of impact magnitudes, challenges associated with quantifying non‐ecological impacts, and research biases, before synthesising how risks are analysed and impacts assessed, and how these assessments ultimately inform management decisions. Our review underscores the multifaceted and complex nature of invasion impacts, and that effectively addressing biological invasions requires more than isolated, reactive interventions; it calls for globally coordinated, proactive action underpinned by reliable scientific knowledge, sincere political commitment, and broad public engagement. Drawing on nearly a century of literature and global expert contributions, this work offers a comprehensive, nuanced, and timely overview of the potential consequences of biological invasions, providing a valuable foundation for informing future research directions, management interventions, and policy development.

## I. INTRODUCTION

The concept of biological invasions has been far from static over time, evolving in response to changing human perspectives in ecology, biogeography, and socio‐economics. At the global level, by fundamentally eroding many biogeographical barriers that kept organisms isolated, humans have facilitated an unprecedented interchange of species (Briski *et al*., 2013; Capinha *et al*., 2015). This exchange has contributed to the alteration of recipient ecosystems, resulting in notable modifications and impacts ranging from biodiversity loss to changes in ecosystem functions (Charles & Dukes, 2007; Bellard, Bernery & Leclerc, 2021). Biological invasions are recognised as a growing concern worldwide due to their extensive ecological, economic, and socio‐cultural impacts (Blackburn, Bellard & Ricciardi, 2019; Roy *et al*., 2023c; Turbelin *et al*., 2023). Increasing introduction rates over past decades and growing impacts became of great interest for many naturalists throughout human history (Seebens *et al*., 2017; Haubrock *et al*., 2023a). What began as a collection of early anecdotal observations by Charles Darwin and others has developed into a rigorous scientific discipline that integrates insights from multiple established fields (e.g. ecology, economics, sociology) focused on predicting, managing, and mitigating the consequences of biological invasions (Darwin, 1889; Ricciardi & MacIsaac, 2008; Vaz *et al*., 2017). Today, the threat posed by non‐native species introductions is recognised by stakeholders and politicians alike, with biological invasions being explicitly mentioned in national (Banerjee *et al*., 2021; Mayer *et al*., 2023) and international agreements and conservation targets (McGeoch *et al*., 2023) like the Convention on Biological Diversity's (CBD) Aichi biodiversity target 9 (CBD, 2010) and target 6 of the Kunming–Montreal Global Biodiversity Framework (www.cbd.int/gbf/targets/6).

The impacts of biological invasions, however, are often nuanced and context dependent, and in many cases, challenging to quantify (Crystal‐Ornelas & Lockwood, 2020; Grimm *et al*., 2020). For instance, certain non‐native species present measurable benefits alongside their harmful ecological impacts (Sax, Schlaepfer & Olden, 2022; Carneiro *et al*., 2024a), complicating legislative changes and the application of management measures (Kourantidou *et al*., 2022). Some introduced plants, such as the black locust (*Robinia pseudoacacia*), provide valuable ecosystem services, including soil stabilisation and nectar provision (Zhang *et al*., 2016), despite negatively affecting native biodiversity (Kato‐Noguchi & Kato, 2024). Introduced honeybees (*Apis mellifera*) and bumblebees (*Bombus* spp.) play an important role in crop pollination worldwide (Russo, 2016) and, at the same time, are known to threaten native insects and disturb the pollination of native plants while enhancing that of non‐native plants (Goulson, 2003; Goulson, Lye & Darvill, 2008). Similarly, non‐native fishes like the dusky spinefoot (*Siganus luridus*) and the marbled spinefoot (*S. rivulatus*) have devastating impacts on Mediterranean reefs, transforming algal forests into rocky barrens (Sala *et al*., 2011), yet rank first in both catch volume and value in Cyprus' commercial and recreational fisheries, where they are considered a high‐quality resource (Michailidis, Katsanevakis & Chartosia, 2020). Fish species like the North American rainbow trout (*Oncorhynchus mykiss*) sustain significant aquaculture production and recreational fisheries in Europe (Lyach, 2022), whereas the European brown trout (*Salmo trutta*) sustains major recreational fisheries but also causes notable ecological impacts in North America and New Zealand (Budy & Gaeta, 2017; Jones & Closs, 2017). In Japan both species are considered established non‐native fishes serving similar roles (Hasegawa, 2020), while simultaneously threatening native communities (Miyamoto, Fukuda & Michita, 2024; Peterson *et al*., 2024). Beyond these examples, the ecological, economic, or socio‐cultural impacts of non‐native species remain uncertain due to limited, difficult‐to‐obtain empirical data (Simberloff *et al*., 2013; Latombe *et al*., 2023), complexity of interactions (Essl *et al*., 2020), long time lags before effects become apparent (e.g. ‘sleeper populations’; Spear *et al*., 2021), or an inherent inability to quantify socio‐cultural effects (e.g. on cultural identity, recreation, or traditional practices; Simberloff *et al*., 2013; Read *et al*., 2020).

Despite considerable recent advances in the conceptual understanding of biological invasions (e.g. Roy *et al*., 2023a; Haubrock *et al*., 2025c), critical knowledge gaps persist in our understanding of the multifaceted impacts that result from introductions of non‐native species. These include, but are not limited to, (*i*) the measurement of impacts, (*ii*) the complex interplay of ecological, economic, and socio‐cultural factors, (*iii*) the variability in ecosystem responses to biological invasions, and (*iv*) the underlying context‐dependent nature of impacts. Furthermore, we introduce a three‐pillar conceptual framework that distinguishes ecological, economic, and socio‐cultural impacts and explicitly links them to methodological challenges and management implications. The aim of this review is thus to examine the nature and diversity of invasion impacts, emphasising the conceptual and methodological challenges inherent in their assessment, and to build upon them. By addressing these challenges and exploring future research directions, we seek to clarify the understanding of the impacts of biological invasions, leveraging past efforts that have greatly advanced this knowledge, and to guide management strategies and policy decisions better. Accordingly, we synthesise insights from a broad body of literature based on the collective expertise of the authors, including peer‐reviewed studies, grey literature, and expert reports. This approach seeks to offer a comprehensive and conceptually grounded overview of the current knowledge on the impacts of biological invasions.

## II. THE STUDY OF IMPACTS

### (1) What are invasion impacts?

In the context of biological invasions, ‘impact’ generally refers to any measurable ‘change’ or ‘effect’ (negative, neutral, or positive) on biodiversity, ecosystems, economies, or human society caused by the introduction of non‐native species (Larson & Kueffer, 2013; Barney & Tekiela, 2020). Definitions and perceptions of impacts can vary widely depending on ecological perspective, economic considerations and interests, and cultural contexts, leading to significant debate and inconsistencies (Lockwood, Hoopes & Marchetti, 2013; Pereyra *et al*., 2024, 2025). Past studies have revealed a diversity of effects associated with non‐native species introductions (Schlaepfer, Sax & Olden, 2011; Simberloff *et al*., 2013; Sax *et al*., 2022) and therefore used impacts as a practical and immediate approach to define the invasiveness of a non‐native species. This is especially the case from a management and legislative point of view (Ricciardi & Cohen, 2007; Pearson *et al*., 2016) as in the European Union Regulation on Invasive Alien Species (EU Regulation No. 1143/2014 hereafter), which defines ‘invasive alien species’ as alien species whose introduction or spread has been found to threaten or adversely impact upon biodiversity and related ecosystem services (Martín‐Forés *et al*., 2024). This approach presents several shortcomings, such as the frequent absence of conducted impact assessments and the difficulty of attributing impacts to the introduction of some species due to confounding effects (e.g. habitat alteration, pollution, climate change; Soto *et al*., 2024a). Rather, as the term ‘invasive’ mainly relates to a species' capacity to spread into a new area (*sensu* Soto *et al*., 2024a), impacts should not be the principal element used to define the invasiveness of a non‐native species, especially as a form of impact occurs at every stage of the invasion (Blackburn *et al*., 2011). Nevertheless, assessing the impacts of non‐native species remains equally important because they determine the urgency and necessity of selective pre‐invasion biosecurity measures and post‐invasion management interventions, thus helping prioritisation (Robertson *et al*., 2021).

Invasion impacts are usually categorised as ecological, economic, or socio‐cultural. Ecological impacts focus on changes in native biodiversity (Dorcas *et al*., 2012), habitat structure, or physico‐chemical composition (Sousa, Gutiérrez & Aldridge, 2009), species interactions [e.g. predation and competition (Kamaru *et al*., 2024), community structure alterations (Everts *et al*., 2024)], and ecosystem functioning (Sousa *et al*., 2011). Economic impacts centre on the valuation of the monetary costs incurred due to non‐native species, such as agricultural losses, infrastructure damage, or management expenses (Farnsworth *et al*., 2017; Diagne *et al*., 2021; Ahmed *et al*., 2023; Tambo *et al*., 2023). Socio‐cultural impacts encompass effects on human health, cultural values, recreational activities, and general well‐being and quality of life (Jones, 2017; Mazza & Tricarico, 2018), although sometimes they are merged with economics as in the Socio‐Economic Impact Classification of Alien Taxa (SEICAT) framework (Bacher *et al*., 2018). Overarching categories of invasion impacts focus on different but complementary aspects that are often largely interconnected (e.g. ecological economy; Cook *et al*., 2007). Economic and social costs are increasingly acknowledged, often through ecosystem services and Nature's Contributions to People frameworks (Katsanevakis *et al*., 2014; Bacher *et al*., 2018; Tsirintanis *et al*., 2022), whereas the assessment of socio‐cultural impacts generally still lags behind ecological and economic dimensions, partly due to limited interdisciplinary integration. The recent focus of invasion scientists on assessing economic and socio‐cultural impacts, however, only followed after the investigation of ecological impacts due to their difficult assessment and quantification (Diagne *et al*., 2021). These impact categories are also more immediately understandable to stakeholders, policymakers, and the public, which has made them especially effective for raising awareness about biological invasions and securing funding for research and management (McGeoch *et al*., 2010; Scalera, 2010). However, no impact categories can be considered a proxy for all impacts as, for example, a non‐native species can have detrimental ecological impacts but benign or even positive impacts on human economy or health, and *vice versa*.

**Fig. 1:**
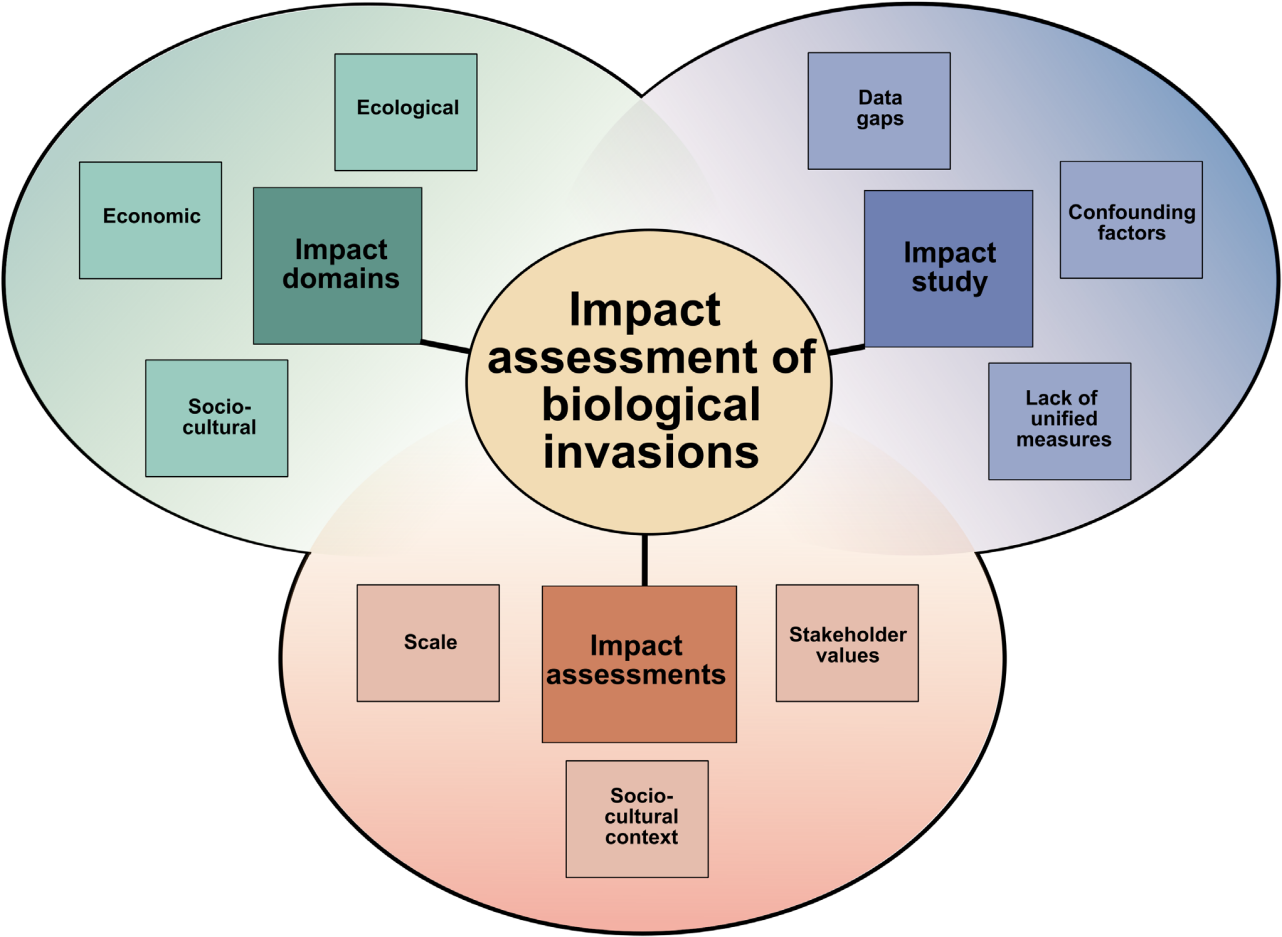
Conceptual framework for understanding invasion impacts rests on three interrelated pillars: impact domains, challenges in the study of impacts, and available risk‐ and impact assessments. First, impacts can manifest across ecological, economic, and socio‐cultural domains (individually or in combination) producing distinct yet interconnected consequences. Second, evaluating the impacts of biological invasions faces practical and methodological challenges, including confounding factors, data deficiencies, and often a lack of empirical evidence. Third, the significance and perception of impacts are deeply subjective, shaped by spatial and temporal scales, stakeholder values, and socio‐cultural context. This tripartite, hierarchical structure provides a foundation for the more detailed analysis in the following sections, where we explore specific dimensions and examples of invasion impacts.

A central challenge in assessing invasion impacts lies in determining what constitutes a ‘significant’ ecological, economic, or socio‐cultural impact (Fig. 1). Any such assessment is inherently subjective and conceptually challenging as perceptions of significance vary across different perspectives and disciplinary frameworks (Carlton, 2002; Simberloff *et al*., 2013). The absence of a ‘significant’ effect, for instance, does not equate to a lack of impact. An impact that is considered substantial or intolerable by one individual, scientist, stakeholder, or policymaker may be perceived as negligible or even beneficial by another, highlighting the subjectivity inherent in impact assessments. The scale of impacts may occur at individual, local, or broader levels as a localised non‐native species might cause socio‐economic harm, such as property damage or health issues, without posing a national concern. Similarly, small populations of non‐native species can prey on individuals of native species, without affecting overall populations, raising questions about whether such localised impacts are significant or if thresholds should apply. Should assessments focus on the greatest impact, possibly neglecting other effects? The deeply subjective nature of evaluating the impacts of biological invasions thus depends on both the epistemological and cultural contexts (Moon, Blackman & Brewer, 2015), reflecting deeper societal values and trade‐offs between economic gains, ecological integrity, and competing interests (Löfqvist *et al*., 2023).

### (2) The history of studying invasion impacts

From the moment that humans began relocating species, whether intentionally or unintentionally, the consequences of these movements have attracted the attention of naturalists. Among the earliest human migrations, the Austronesian expansion (~3000–1500 BC) was a well‐documented large‐scale migration that introduced various species to previously uninhabited remote Pacific Islands (Chang *et al*., 2015; Kirch, 2017). Some of these introductions (e.g. the Pacific rat, *Rattus exulans*) persist today and have profound, lasting ecological impacts that have permanently altered the ecological trajectories of these islands (Matisoo‐Smith & Robins, 2004). During the reign of Augustus (27 BC–14 AD), the Roman Empire launched one of the earliest recorded eradication efforts after European rabbits (*Oryctolagus cuniculus*) devastated crops and food supplies on the Balearic Islands, contributing to famine (Brunel *et al*., 2013). From the late 19th century, with the acceleration of the trade in plants and plant products among continents, several harmful non‐native plant pests started to threaten the survival of entire agricultural sectors, such as the cottony cushion scale (*Icerya purchasi*) threatening the citrus industry in California, the grapevine Phylloxera (*Viteus vitifoliae*) devastating the wine industry in Europe, and the Colorado potato beetle (*Leptinotarsa decemlineata*) seriously affecting potato production in Europe (Planchon, 1874; Riley, 1887; Perpillou, 1933). While, at that time, quantified economic impacts were not properly assessed, the problems were considered sufficiently severe to generate long and expensive management programmes, including extensive studies in the area of origin of the pests to select natural enemies for introduction in the newly invaded regions (Clausen, 1978). For most other non‐native species, biological invasions and the study of their impacts were long viewed more as anecdotal events rather than one of the greatest threats to global biodiversity and ecosystems (Brunel *et al*., 2013). It was not until the studies of naturalists such as Charles Darwin, Joseph D. Hooke, and Alfred R. Wallace, among others, that biological invasions and associated impacts on native species were recorded in detail (Hooker, 1864; Brunel *et al*., 2013; Barnard, 2015). A major turning point came with British ecologist Charles S. Elton and his seminal work *The Ecology of Invasions by Animals and Plants* published in 1958, which is considered the starting point of invasion science as a scientific discipline (Richardson & Pyšek, 2008).

**Fig. 2:**
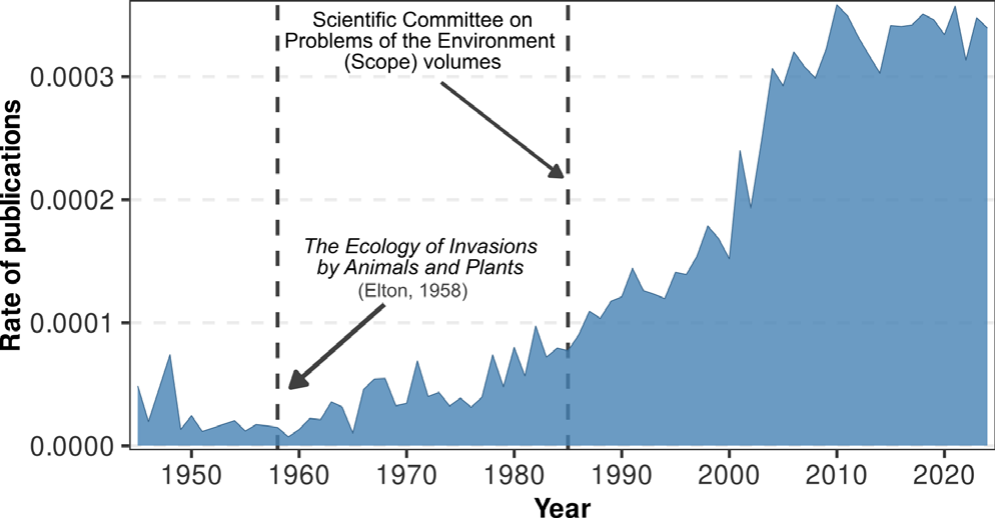
Annual rate of publications related to the study of the impact of non‐native species out of all publications listed in the *Web of Science* highlighting the rise of invasion biology in the context of overall science output. A comparison against all publications within the field of Ecology in the *Web of Science* is provided in Fig. S1. Details on data extraction can be found in Appendix S1. Since its explosion in the 1980s, the relative rate of publications grew exponentially until 2010.

Elton warned that ‘ecological explosions’ (i.e. invasions) were escalating in impact and could fundamentally alter ecosystems, calling for the conservation of native diversity. The growing interest in biological invasions (and their impacts) precipitated the publication of the Scientific Committee on Problems of the Environment (SCOPE) volumes, which highlighted the threat posed by non‐native species (Lockwood *et al*., 2013). Numerous books and journal articles followed and inspired a new generation of researchers dedicated to understanding this ‘new’ environmental concern (Vitousek *et al*., 1996; Lockwood *et al*., 2013), although the broader recognition that impacts of non‐native species extend beyond the ecological realm is a relatively recent development in the historical timeline (i.e. 1980s). The interest in the ‘impact’ of non‐native species (Figs 2 and S1, see online Supporting Information, Appendix S1, for details of construction of these figures) has driven the development of conceptual frameworks and classification systems (such as the framework of Parker *et al*., 1999) aimed at comparing and quantifying impacts across taxa and ecosystems (Ricciardi, 2003). These include models linking impact to species traits and distribution, as well as standardised classifications like the Environmental Impact Classification for Alien Taxa (EICAT; Hawkins *et al*., 2015) and SEICAT, which assess ecological and socio‐economic effects, although notably excluding monetary costs (Soto *et al*., 2023d). As the issue continues to escalate, the Intergovernmental Science‐Policy Platform on Biodiversity and Ecosystem Services (IPBES) released the Invasive Species Assessment in 2023, which recognises that biological invasions pose a global threat to biodiversity and ecosystems, affecting local, regional, and national economies, food and water security, and human health, while further exacerbating social inequalities (Linders *et al*., 2020; Diagne *et al*., 2021; Bacher *et al*., 2023a).

## III. TYPES OF INVASION IMPACTS

Biological invasions generate a wide range of ecological, economic, and socio‐cultural impacts that differ in severity, visibility, and measurability (Simberloff *et al*., 2013; Shackleton *et al*., 2019b; Diagne *et al*., 2021). While some non‐native species cause rapid or drastic biodiversity loss and ecosystem disruptions, others primarily exert financial burdens on specific industries or sectors like agriculture, forestry, and fisheries (Gallardo *et al*., 2024) or affect human well‐being by transforming cultural landscapes, traditions, or public health (Pejchar & Mooney, 2009). These different types of impacts are complex and can have far‐reaching consequences, but do not occur in isolation; rather, they are highly interconnected. Ecological changes can lead to economic losses, while socio‐cultural values may shape how humans perceive and respond to biological invasions (Pfeiffer & Voeks, 2008).

### (1) Ecological impacts

Invasion science first recognised ecological effects, which rapidly generated different perceptions (Davis, 2011; Tassin & Kull, 2015; Sax *et al*., 2022). Ecological impacts extend across multiple levels of biological organisation, including the individual (e.g. fitness, behaviour or growth), population (e.g. population size), species (e.g. species range change), community (e.g. community structure), and ecosystem level (e.g. primary and secondary production, decomposition, nutrient cycling), with complex, bidirectional feedbacks between these levels (Vilà *et al*., 2024; Carneiro *et al*., 2025). The mechanisms by which biological invasions disrupt the natural equilibrium are multifaceted (Buckley & Catford, 2016) and can manifest in diverse ways, including predation, parasitism, herbivory, and competition (Doherty *et al*., 2016), the spread of infectious diseases (Hulme, 2014), behavioural alterations in native species (Ruland & Jeschke, 2020), disruption of ecosystem services such as pollination (Russo, 2016) and even modifications to the abiotic environment (Doherty‐Bone *et al*., 2019). Moreover, invasion impacts may act synergistically with other stressors, such as climate change, habitat fragmentation, overexploitation, or pollution (Bellard, Cassey & Blackburn, 2016; Ricciardi *et al*., 2021).

The extent to which non‐native species affect recipient environments depends on a range of factors, including the species' traits, local population abundance, density, biomass, distribution, functional role in the trophic web (e.g. its trophic guild or position) and the ecosystem (e.g. keystone, hinge, or ecosystem engineer species), functional distinctiveness, as well as the biotic and abiotic characteristics of the invaded habitat (Strayer, 2012; Thomsen *et al*., 2014a; Everts *et al*., 2024; Rilov, Canning‐Clode & Guy‐Haim, 2024). In naturally diverse ecosystems, biological resistance from native species may constrain the ability of non‐native species to establish, proliferate, and cause significant ecological impacts (‘biotic resistance hypothesis’; Stachowicz, Whitlatch & Osman, 1999; but see Jeschke *et al*., 2012; Jeschke & Heger, 2018). Conversely, degraded habitats or stressed native communities tend to be more vulnerable to invasion impacts (Byers, 2002; Cadotte *et al*., 2017; Liu *et al*., 2023), whereas relatively pristine ecosystems characterised by high habitat or environmental heterogeneity may buffer the impacts of non‐native species (Melbourne *et al*., 2007; Boon *et al*., 2023).

**Fig. 3:**
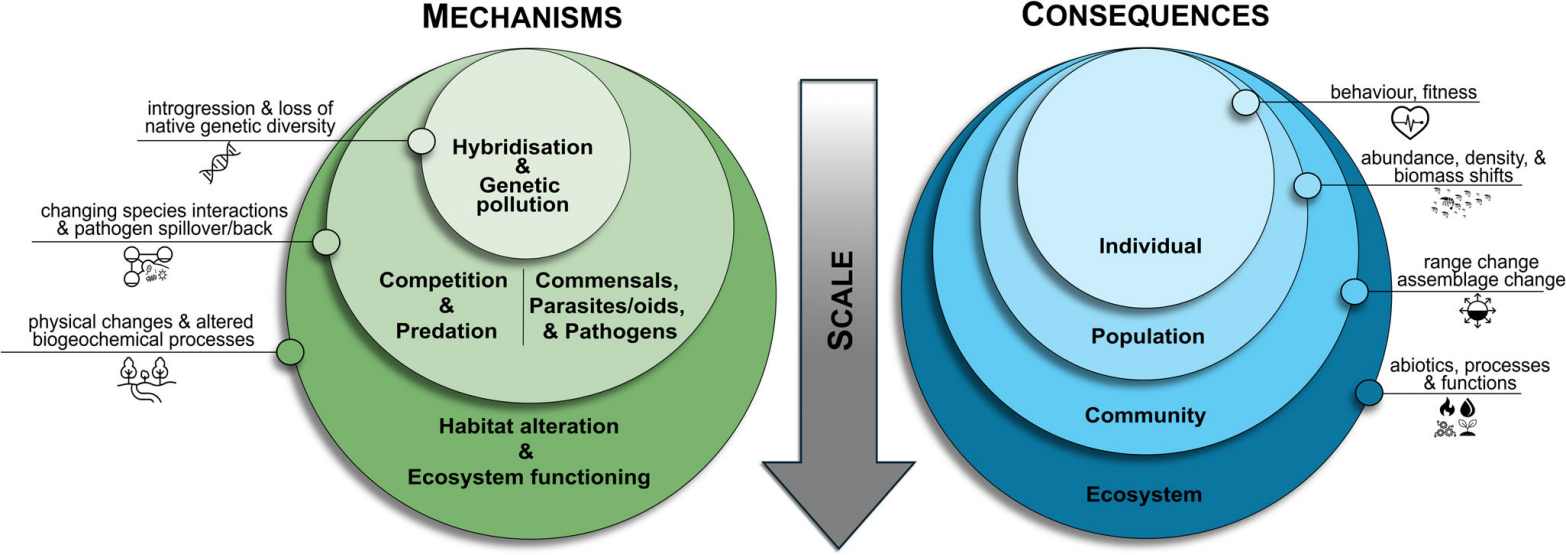
Conceptual illustration of the order and scaling of mechanisms and consequences of how non‐native species exert ecological effects.

**Fig. 4:**
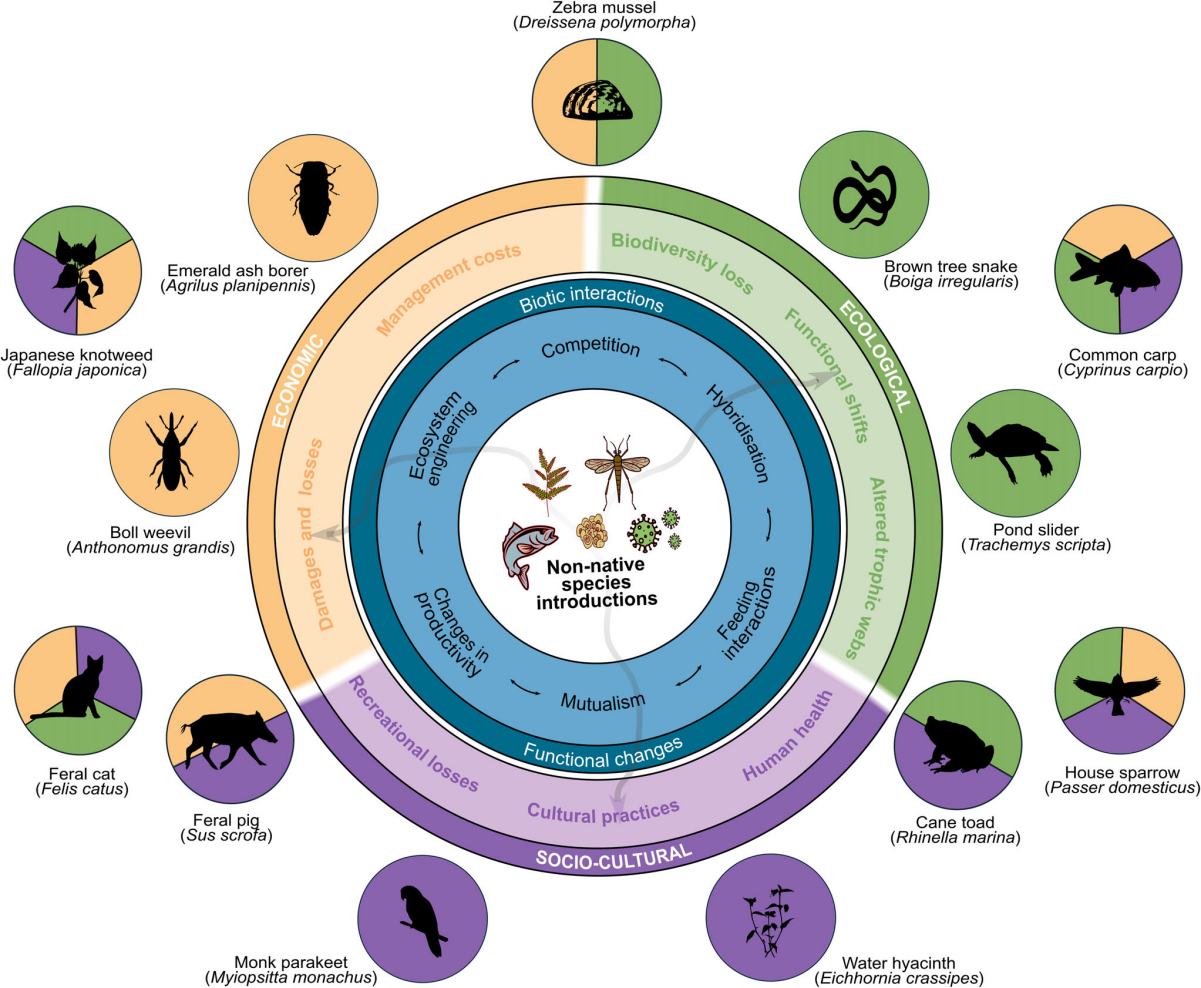
Conceptual illustration of how non‐native species exert ecological, economic, and socio‐cultural effects (negative, positive, or neutral) through biotic interaction mechanisms (see Table 1). The non‐native species is placed at the centre, surrounded by direct and indirect biotic interactions and functional changes (inner ring), which mediate impacts across three broad domains (outer ring; highlighting example categories). Arrows indicate that the underlying impact mechanisms and effects are interconnected, and that boundaries between mechanisms and domains are often fluid or overlapping. Categories (e.g. ‘biodiversity loss’) are used as shorthand for clusters of well‐recognised consequences such as species declines, community shifts, or altered ecosystem processes, and are intended as illustrative rather than exhaustive. Examples of species and known associated impacts, reflecting only their most widely recognised or primary impacts, include: *Boiga irregularis* (ecological – bird loss), *Dreissena polymorpha* (ecological & economic – ecosystem disruption and pipe clogging), *Agrilus planipennis* (economic – tree damage), *Anthonomus grandis* (economic – crop loss), *Myiopsitta monachus* (socio‐cultural – noise), *Eichhornia crassipes* (socio‐cultural – blocked waterways), *Trachemys scripta* (ecological – competition), *Rhinella marina* (ecological & socio‐cultural – predator poisoning, food chain disruption), *Sus scrofa* (economic & socio‐cultural – crop and cultural site damage), *Cyprinus carpio* (ecological, economic & socio‐cultural – vegetation uprooting, fishery decline, cultural waterway degradation), *Felis catus* (ecological, economic & socio‐cultural – wildlife predation, poultry/tourism impact, indigenous species relations), *Passer domesticus* (ecological, economic & socio‐cultural – native bird competition, crop/building damage, cultural symbolism), and *Fallopia japonica* (ecological, economic & socio‐cultural – native plant displacement, infrastructure damage, diminished cultural landscape value).

Ultimately, ecological invasion impacts can be portrayed from a number of different angles (Cucherousset & Olden, 2011; Lockwood *et al*., 2013). In this review, we adopt the Britton (2023) framework for ecological impacts, which offers a distinct approach compared to more traditional models by categorising impacts across hierarchical levels of biological organisation, and by clearly distinguishing between the ecological process or pathway by which change is mediated (i.e. the mechanism) and the level of biological organisation that is affected (i.e. the consequence) (Figs 3 and 4). While often conflated, distinguishing between mechanisms and consequences is essential for accurately assessing and clearly communicating ecological impacts (Carneiro *et al*., 2025). In what follows, we explore the most common mechanisms by which non‐native species influence ecosystems – often arising from direct or indirect biotic interactions (Table 1), although the boundaries between these mechanisms can be ambiguous – accompanied by several exemplary case studies, and their ecological consequences. These mechanisms are organised thematically, while broadly following the dominant ecological level at which their impacts manifest. We then proceed to review the ecological consequences (i.e. impacts) of these mechanisms across the different levels of biological organisation. Finally, we examine the distinct ecological impact dynamics of non‐native species and their co‐introduced symbionts, given their unique characteristics and the ways in which they differ from other types of ecological impacts.

**Table 1:** Direct biotic interaction mechanisms and consequences through which non‐native species can affect native populations and ecological communities. The listed categories represent functional pathways rather than outcomes, describing how non‐native species engage with, alter, or displace native species and their ecological roles.

Biotic interaction type	Definition	Typical consequence (example)	Key reference
Predation	Consumption of native species by non‐native predators, often leading to population declines or local extinctions.	Mortality of native species → reduced recruitment → community simplification → trophic cascade	Doherty *et al*. (2016)
Herbivory, feeding, and grazing	Feeding on native plants by non‐native herbivores, which can alter plant communities and reduce native plant fitness.	Loss of native biomass → reduced recruitment of palatable species → dominance of tolerant taxa → altered nutrient cycling	Courchamp *et al*. (2003)
Competition	Non‐native species may compete with natives for shared resources such as food, shelter, or breeding sites. This can occur through (*i*) exploitative competition, where resources are depleted before others can access them; and (*ii*) interference competition, where direct interactions prevent access to resources.	Reduced growth of native species → population decline → altered community structure → ecosystem function change	Bertolino *et al*. (2014)
Mutualism formation and disruption	Non‐native species may interfere with, replace, or form new mutualistic relationships, affecting key ecological functions. Examples include (*i*) disruption or monopolisation of pollination and seed dispersal, (*ii*) alteration of microbial or mycorrhizal associations, and (*iii*) formation of novel and disruption of existing facilitative interactions with native species.	Disrupted pollination/seed dispersal → reduced regeneration of natives → decline of dependent fauna → collapse of mutualistic networks	Kamaru *et al*. (2024)
Commensalism	Asymmetric interactions in which non‐native species benefit from native species without reciprocation or negatively affect natives without direct benefit to themselves.	Benefit to invader without reciprocal effect → increased invader success → competitive disadvantage for natives → shifts in community structure	Hulme‐Beaman *et al*. (2016)
Hybridisation and genetic pollution	Interbreeding between non‐native and native species, which can lead to loss of genetic integrity, reduced fitness, or outbreeding depression in native populations.	Reduced fitness of native lineage → genetic swamping → loss of local adaptations → decreased resilience	Blackwell *et al*. (2021)
Allelopathy	Release of biochemicals by non‐native species (primarily plants) that inhibit germination, growth, or reproduction of native species. This can also include chemical signalling disruption (e.g. olfactory crypsis) or palatability in animals.	Inhibition of native germination/growth → reduced native abundance → altered community composition → reduced ecosystem resilience	Kalisz *et al*. (2021)
Trophic cascades	Indirect ecological effects resulting from changes in species abundances across multiple trophic levels, often initiated by non‐native predators or herbivores.	Changes in abundance at one trophic level → secondary population responses → restructuring of food web → altered ecosystem processes	Walsh *et al*. (2016)
Ecosystem engineering	Physicochemical alteration of the environment by non‐native species through activities such as burrowing, digging, dam‐building, vegetation, or soil and water modifications. These changes can restructure habitats, influence resource availability, and affect the distribution and interactions of native species.	Physico‐chemical habitat alteration → community reassembly → long‐term ecosystem state shift	Rilov *et al*. (2024)
Parasitism and disease transmission	Introduction or amplification of parasites and pathogens by non‐native species, which can infect native hosts and disrupt population dynamics through novel or intensified disease pressures.	Infection of native species → population crash → community turnover → altered ecosystem functioning	Crowl *et al*. (2008)

#### (a) Ecological impact mechanisms

##### (i) Competition, mutualism, and beyond

The introduction of non‐native species can affect native species through the direct effects of these novel interactions, or by modifying pre‐existing interspecific relationships (Čuda *et al*., 2015; Sarabeev *et al*., 2022). Non‐native species can, for instance, affect native species through competition for shared and limited resources, including food, shelter, breeding or nesting sites in animals (Savvides, Louca & Sfenthourakis, 2015; Charter *et al*., 2016), and light, pollinators, space, or nutrients in plants (Dybzinski & Tilman, 2007). Competition can either take the form of interference competition, where the non‐native species directly affects one another's access to resources, exploitative competition, in which species indirectly compete by depleting shared resources (Human & Gordon, 1996; Byers, 2000; Damas‐Moreira *et al*., 2020; Ficetola *et al*., 2024), or behavioural interference, by modifying the behaviour of the native species at the expense of the latter (Liu *et al*., 2007). While competition for food is a form of intraguild (i.e. within the same trophic level) impact (Revilla, 2002), competition for other critical resources can involve species from different trophic levels but sharing similar traits (e.g. nesting in similar environments; Sergio, Marchesi & Pedrini, 2003).

The outcome of competition strongly depends, among other factors, on the availability of shared resources and prevailing disturbance regimes. Nutrient availability and abiotic stress often regulate the intensity of competition and competitive hierarchies among species (Emery, Ewanchuk & Bertness, 2001). Additionally, niche partitioning may occur in nutrient‐poor conditions, thereby reducing or preventing direct competition (Chesson, 2000). Nonetheless, niche partitioning can still have negative consequences for native biodiversity (e.g. Guerin *et al*., 2019), as it often forces native species to shift towards suboptimal resources in response to the presence of non‐native species (Curtis *et al*., 2017). For instance, an increased reliance of native species over a less‐profitable food source can reduce the energy intake and thus affect the fitness of the population (Sih *et al*., 2010). Similarly, the shift of native species' shelters or breeding or nesting sites towards more exposed or environmentally less suitable areas can directly negatively affect the survival probabilities of the native species and its offspring (Robertson & Chalfoun, 2016), thus reducing fitness. In extreme cases, such fitness reductions can even culminate in regional species extinctions (Bertolino *et al*., 2014). Stable conditions, on the other hand, allow populations to increase in abundance with minimal disruption, thereby intensifying competitive interactions. Conversely, high disturbance regimes continually reduce population densities and, by creating unoccupied niches, may shift the balance from competition towards facilitation (Zhang & Wang, 2016). Under stable, nutrient‐rich conditions, fast‐growing species often monopolise available resources, leading to intense competitive exclusion. In nutrient‐poor environments subjected to frequent disturbances, competition is generally reduced, as species either buffer harsh environmental conditions or facilitate nutrient acquisition (He, Bertness & Altieri, 2013).

Although the competitive impacts of non‐native species are frequently examined in terms of nutrient and energy uptake or microhabitat use, other outcomes may also manifest. For example, the calling activity of invasive American bullfrogs (*Lithobates catesbeianus*) can induce sympatric native frog species to adjust the spectral properties of their advertisement calls, thereby influencing mate attraction and territorial signalling (Both & Grant, 2012). Similar subtle changes can also occur in non‐native plant species with significant impact on plant–animal interactions and biotic relationships at higher trophic levels. For instance, introduced entomophilous plants depend on resident pollinators for reproduction, thereby competing with native flora for pollination services (Brown, Mitchell & Graham, 2002; Morales & Traveset, 2009). This can disrupt the structure and stability of entire plant–pollinator networks (reviewed in Parra‐Tabla & Arceo‐Gómez, 2021). Nevertheless, non‐native plants often attract more pollinators, even during early stages of invasions, potentially lowering the reproductive success of native plants and disrupting long‐established eco‐evolutionary dynamics (Kandori *et al*., 2009; Vilà *et al*., 2009). In some cases, plant–pollinator interactions may shift and non‐native plants may act as ‘magnet species’, enhancing pollinator visitation to nearby native plants (Aizen, Morales & Morales, 2008; Bartomeus, Vilà & Santamaría, 2008).

Non‐native species can also gain competitive advantages by introducing traits or interactions that are unfamiliar to the invaded ecosystem (i.e. the ‘novel weapons' hypothesis; Callaway & Ridenour, 2004), which can take various different forms. Allelopathy is a common trait in this regard, pervasive in non‐native plants (Kalisz, Kivlin & Bialic‐Murphy, 2021), referring to plants releasing biochemicals that inhibit the growth or reproduction of native species, disrupting plant community dynamics (Callaway & Ridenour, 2004). In animals, invasive red lionfish (*Pterois volitans*) in the western Atlantic have been shown to use a form of chemical camouflage, or ‘olfactory crypsis’, to avoid detection by native prey species, allowing them to hunt more effectively and outcompete native predators (Lönnstedt & McCormick, 2013). Similarly, the unpalatability of non‐native American bullfrogs to native predators is thought to contribute to their invasion success (Szuroczki & Richardson, 2011).

Competitive and chemical mechanisms may also interact synergistically with other types of biotic interactions in ways that exacerbate ecological impacts. One such process is posited in the ‘invasional meltdown’ hypothesis (Simberloff & Von Holle, 1999), where the presence of one non‐native species facilitates the establishment, spread, or ecological effects of another. For example, the presence of a non‐native plant, *Conyza canadensis*, facilitated the increase in aboveground biomass of other non‐native plants over native species in high‐nutrient substrates – an effect not observed in the absence of *C. canadensis* – possibly promoting establishment success of other non‐native species and overcoming biotic resistance (Sun *et al*., 2024). Related to this are secondary invasions, which differ from the concept of invasional meltdown in that only the success of one non‐native species (i.e. the secondary invader) is dependent on the presence or impact of another non‐native species (i.e. the primary invader; O'Loughlin & Green, 2017). Apparent competition is another mechanism that occurs when the presence of one species indirectly affects another species at the same trophic level through the increased presence of a shared enemy (Holt & Bonsall, 2017). Apparent competition between non‐native and native species has been observed primarily in plants (Dangremond, Pardini & Knight, 2010), but also occasionally in other taxonomic groups and trophic levels, such as herbivorous insects (Settle & Wilson, 1990) or aquatic molluscs (Castorani & Hovel, 2015).

Beyond competition, non‐native species can also establish or disrupt other biological interactions with notable consequences for native ecosystems. Mutualisms, for instance, are widely affected. In East African savannas, whistling thorns (*Vachellia drepanolobium*) maintain a mutualistic relationship with native acacia ants (*Crematogaster* spp.), offering food and shelter in exchange for protection against herbivores. However, this mutualism is disrupted when native ants are displaced by the non‐native big‐headed ant (*Pheidole megacephala*), which fails to provide effective chemical defence for the trees, leading to cascading ecological consequences (Kamaru *et al*., 2024). By contrast, some invasions create new mutualisms, such as the facilitative relationship between an invasive seaweed and a native tubeworm (Kollars, Byers & Sotka, 2016). Non‐native species may also engage in commensal or amensal relationships, where one species benefits or is harmed while the other remains unaffected. Though often subtle and difficult to detect, these asymmetrical interactions can alter species distributions, resource use, or population dynamics over time (Mougi, 2016; Northfield *et al*., 2018). Finally, it is important not to assume that competition is the primary mechanism driving impacts among species within the same ecological guild (i.e. those using similar resources), as direct interactions such as intraguild predation can play a more significant role (Polis, Myers & Holt, 1989). For example, many crustacean invaders kill and consume native ones, with predation being a more accurate explanation for species displacement than resource competition (Dick & Platvoet, 2000). Similarly, the invasive Harlequin ladybird (*Harmonia axyridis*) has caused the decline of some native ladybird species both in Europe (Kenis *et al*., 2020), North America (Bahlai *et al*., 2015), and South America (Grez *et al*., 2016). However, it is not clear whether the main mechanism explaining the decline is competition for food or intra‐guild predation.

##### (ii) Consumer–resource interactions

An increasing number of studies have demonstrated the immediate direct or indirect top‐down effects of non‐native predators (Snyder & Evans, 2006; Martin‐Albarracin *et al*., 2015). Notorious in this regard causing decline and extirpation of native species is the introduced predatory rosy wolfsnail (*Euglandina rosea*) in Pacific islands (Régnier, Fontaine & Bouchet, 2009), the brown tree snake (*Boiga irregularis*) on Guam (Savidge, 1987; Fritts & Rodda, 1998), the California kingsnake (*Lampropeltis getula*) in the Canary Islands (Piquet & López‐Darias, 2021) or the Nile perch (*Lates niloticus*) introduced into Lake Victoria (Pringle, 2005). Observed population declines and extirpations caused by non‐native predators represent extensive shifts in biodiversity and ecosystem functioning, with cascading effects at several trophic levels (Rogers *et al*., 2017). For example, Kurle, Croll & Tershy (2008) found that invasive brown rat (*Rattus norvegicus*) reduced glaucous‐winged gull (*Larus glaucescens*) and black oystercatcher (*Haematopus bachmani*) populations, releasing intertidal invertebrates from predation pressure. This shifted the community from algal to invertebrate dominance due to increased grazing by snails and limpets and space from algal decline. Similar effects can arise from the introduction of non‐native herbivores (e.g. goats, rabbits, and deer), which may affect native plants either directly through consumption or also by disrupting their pollinators or seed dispersers, ultimately resulting in the loss of vegetation and, potentially, increased erosion (Courchamp, Chapuis & Pascal, 2003; Gormley *et al*., 2012). Non‐native herbivorous arthropods and plant pathogens, such as the emerald ash borer (*Agrilus planipennis*), the hemlock woolly adelgid (*Adelges tsugae*), the chestnut blight (*Cryphonectria parasitica*), and others, have caused severe declines of North American forest ecosystems (Anagnostakis, 1987; Herms & McCullough, 2014; Ellison *et al*., 2018), with various consequences across all trophic levels and ecosystem functions (Kenis *et al*., 2009).

Consumer–resource interaction impacts can have far‐reaching ecological consequences, especially when the non‐native predators or herbivores become firmly established and when the prey/host species are already rare or endangered, making early detection and rapid response critical in management interventions (Taillie *et al*., 2021; Guzy *et al*., 2023). In some cases, the eradication of non‐native predators and herbivores is followed by a rapid recovery of the impacted native species (Schreiner & Nafus, 1993; Chapuis *et al*., 2011; Prior *et al*., 2018). In other cases, recovery can be a long process (Guzy *et al*., 2023). Moreover, the removal of non‐native species may also lead to the co‐extirpation of associated pathogens (Hossack *et al*., 2023), further facilitating the recovery and resilience of native species. Eradication programmes may result in unexpected changes in population sizes and community composition of native species (Prior *et al*., 2018), such as sudden population growth in non‐native plants that are no longer under such herbivory pressure (Courchamp *et al*., 2003). Following the eradication of non‐native apex predators on islands, smaller‐bodied predator species often increase in abundance. This type of trophic cascade, known as ‘mesopredator release’, can unexpectedly amplify negative impacts on native prey species, potentially undermining the effectiveness of implemented management efforts (Courchamp, Langlais & Sugihara, 1999). Such dynamics are clearly demonstrated by Rayner *et al*. (2007), who investigated the long‐term impacts of feral cats (*Felis catus*) and an introduced mesopredator, the Pacific rat, on the breeding success of a small burrowing bird (Cook's petrel, *Pterodroma cookii*). They revealed that removing apex predators actually resulted in a decline in the nesting success of their bird prey due to increased predation pressure from rats, and that removal of the meso‐predator resulted in increased success to a level above that when both non‐native species were present. In a similar fashion, the eradication of feral cats rapidly precipitated increases in European rabbits on Macquarie Island, Australia (Bergstrom *et al*., 2009). Collectively, these examples underscore the complexities involved in managing biological invaders that exert top‐down trophic effects on the recipient ecosystems through predation and herbivory (Prior *et al*., 2018).

##### (iii) Hybridisation and genetic pollution

An often‐overlooked yet insidious ecological consequence of non‐native species introductions is their impact on native species through hybridisation and introgression (Huxel, 1999; Largiadèr, 2007; Todesco *et al*., 2016; Porretta & Canestrelli, 2023). Hybridisation refers to the successful reproduction between genetically distinct lineages, regardless of their taxonomic status (e.g. species, subspecies, or distinct populations). Introgression involves the transfer of genes between these genetically distinct forms through repeated backcrossing of hybrids with the parent species or populations. Approximately one‐quarter of plant and one‐tenth of animal species are involved in natural interspecific hybridisation and potential introgression (Mallet, 2005). Although the exact frequency of hybridisation between native and non‐native species is uncertain, it is expected to occur more frequently than between native species, as human activities increase the likelihood of interspecific interactions leading to hybridisation (Olden *et al*., 2004; Todesco *et al*., 2016). Additionally, multiple non‐native species within overlapping invaded ranges may hybridise, leading to unpredictable consequences for co‐occurring native species (Brys *et al*., 2025).

Over successive generations, the genetic material of the native species becomes progressively diluted, while certain genes from both hybridising parent are retained (Largiadèr, 2007). Genetic pollution occurs when the gene pool of native species is diluted or altered, resulting from hybridisation with non‐native species (Butler, 1994). While introgression and genetic pollution following hybridisation are bidirectional processes, their outcomes are frequently asymmetrical (Orive & Barton, 2002). These asymmetries can result not only from competitive interactions between species, where the native species is displaced by the invader, but also in the absence of direct competition in sympatric regions. Indeed, asymmetric genetic pollution may arise from demographic imbalances at the invasion front, where the invading species occurs at lower densities (Currat *et al*., 2008). Notably, hybridisation and introgression are not necessarily interlinked. Hybridisation can occur without introgression, particularly when F1 hybrids (i.e. first‐generation offspring) are sterile (Konishi & Takata, 2004). However, empirical evidence for this scenario remains limited, potentially due to its infrequent occurrence or the rapid replacement of local populations when the loss of reproductive value enhances a demographic decline for one (or both) parental species, making detection of this scenario challenging (Wolf, Takebayashi & Rieseberg, 2001; Largiadèr, 2007). Hybridisation without introgression can also occur with fertile F1 hybrids, when offspring are clonal or hemiclonal, transmitting a single parental genome (Quilodrán, Montoya‐Burgos & Currat, 2020). A striking example is sperm theft, as seen in the Gibel carp (*Carassius gibelio*), where females use sperm from males of other species to trigger egg development without incorporating the male's genetic material (Docherty, 2016).

Hybridisation and introgression between native and non‐native species affects genetic diversity at both inter‐ and intraspecific levels. While such intraspecific changes can be neutral or even beneficial (e.g. introducing adaptive traits or the evolutionary rescue of small, inbred populations), they can also negatively affect native species in several ways, depending on the ecological context (Seehausen *et al*., 2008; Quilodrán *et al*., 2020). First, introgression can reduce genetic diversity within native populations, potentially homogenising their gene pools and limiting the genetic variation available for future allopatric speciation or adaptation to changing environmental conditions (Kirkpatrick & Ravigné, 2002; Reed *et al*., 2024). An example of this occurs during the invasion of tilapia species in African lakes, where the non‐native Nile tilapia (*Oreochromis niloticus*) and blue‐spotted tilapia (*Oreochromis leucostictus*) frequently hybridise with native tilapia species such as the Wami tilapia (*Oreochromis urolepis*), resulting in significant changes in gene pools and morphological traits (Shechonge *et al*., 2018), potentially leading to irreplaceable loss of genetic resources (Blackwell *et al*., 2021).

Second, gene pool mixing can result in the expression of maladapted genes or the disruption of co‐adapted gene complexes, leading to reduced fitness in individuals known as outbreeding depression (Waser & Price, 1994; Rius & Darling, 2014). Indeed, hybridisation between non‐native rainbow trout and native westslope cutthroat trout (*Oncorhynchus clarkii lewisi*) has led to significant declines in the reproductive fitness of the native trout (Muhlfeld *et al*., 2009). Simultaneously, hybridisation was found to lead to an enhanced dispersal rate, further driving the expansion of maladaptive hybridisation (Bourret *et al*., 2022).

Third, hybridisation may unpredictably disrupt the behaviour of wild animals, particularly when involving domesticated species that have been artificially selected for traits aligned with human lifestyles. When these domesticated animals spread their genes in natural environments, they can influence entire networks of ecological interactions (Ellington & Murray, 2015). Similarly, hybridisation may introduce intraspecific variability in species behaviour, enhancing the hybrid species' ability to adapt to diverse environments, thereby increasing its invasive potential (D'Amore, Popescu & Morris, 2019; Fournier & Aron, 2021).

Fourth, hybridisation may reduce the effective population size of the interacting species with major consequences for rare or threatened species, which often already have a reduced number of breeders (Palstra & Ruzzante, 2008). An example is the non‐native Oriental weatherfish (*Misgurnus anguillicaudatus*) that, largely due to the pet trade, was introduced outside its native range (Cano‐Barbacil, Haubrock & Radinger, 2025), where it may potentially hybridise with the threatened European weatherfish (*Misgurnus fossilis*). The latter species is experiencing dramatic population collapses across its native range (Wanzenböck *et al*., 2021), and hybridisation exacerbates the prevailing negative effects of land‐use change (Meyer & Hinrichs, 2000) and pollution (Schreiber *et al*., 2017). Moreover, introgression may cause European weatherfish to lose their legal protection status upon *in situ* occurrence of hybridisation, hampering ongoing conservation efforts (Wayne & Shaffer, 2016).

Overall, hybridisation can threaten genomic integrity and fitness, interact with non‐reproductive processes (e.g. predation, competition, parasitism, mutualism, and commensalism) with broad implications for community structure and ecosystem functioning, and may in the most extreme cases lead to extinctions of the parental species (Ellington & Murray, 2015; Todesco *et al*., 2016; Reed *et al*., 2024); a process known as ‘hybrid swarm phenomenon' (Campbell *et al*., 2024). Despite growing awareness of the eco‐evolutionary consequences of hybridisation, much remains to be understood (Porretta & Canestrelli, 2023).

##### (iv) Physical ecosystem engineering

Some non‐native species modify the abiotic and physical structure of the environment to varying degrees when entering a new ecosystem. Consequently, these species may induce changes to ecosystem properties that lead to the creation or destruction of habitats for other species, alter the regimes of physical disturbances, or influence the transport and distribution of resources across ecosystems (reviewed in Rilov *et al*., 2024). Such species are commonly referred to as ecosystem engineers (see Jones, Lawton & Shachak, 1994, 1997). Non‐native ecosystem engineers can also modify the environment for entire biological communities by providing novel habitats for other species, including other non‐native species (Simberloff, 2006), representing a key mechanism that can drive changes in biodiversity and ecosystem functioning (Crooks, 2002; Emery‐Butcher, Beatty & Robson, 2020; Rilov *et al*., 2024). These effects can vary along environmental gradients and are often more pronounced in stressful environmental conditions, where ecosystem engineers may either ameliorate or worsen conditions for other species (Bruno, Stachowicz & Bertness, 2003; Crain & Bertness, 2006; Byers, 2024).

One of the primary mechanisms by which non‐native ecosystem engineers influence ecosystems is through the creation of novel physical structures (Burlakova, Karatayev & Karatayev, 2012; Harvey *et al*., 2019). Although many examples involving non‐native species focus on biodiversity loss resulting from habitat modification (Crooks, 2002), some invasions may instead increase or alter patterns of diversity – directly through habitat provision and indirectly by offering shelter from predation or competition, or by modifying abiotic conditions (Crooks, 2002; Katsanevakis *et al*., 2014). For example, non‐native bivalves such as Asian clams (*Corbicula* spp.), Pacific oyster (*Magallana gigas*), zebra mussel (*Dreissena polymorpha*), golden mussel (*Limnoperna fortunei*), Asian date mussel (*Musculista senhousia*), and Mediterranean mussel (*Mytilus galloprovincialis*) are a well‐known faunal group capable of increasing habitat complexity and heterogeneity (Burlakova *et al*., 2012). The main mechanism by which non‐native bivalves can influence invaded habitats involves the provision of colonisable substrate and refuges through their shells, which can alter the abundance and diversity of macrozoobenthic species (Sousa *et al*., 2009). However, changes in sediment chemistry, grain size, and organic matter content through sediment reworking, increased water transparency resulting from filter feeding, and alterations in near‐bed flow dynamics and shear stress caused by the presence of shells can also play important roles (Sousa *et al*., 2009). Similarly, the non‐native reef‐building polychaete *Ficopomatus enigmaticus* and the ascidian *Pyura praeputialis* contribute additional structural complexity to invaded aquatic ecosystems. By modifying the physical environment, both species influence habitat structure, alter sediment transport, and affect water current dynamics. These new substrates can cover extensive areas, leading to significant impacts on community composition (Castilla, Lagos & Cerda, 2004; Schwindt, Iribarne & Isla, 2004; Bruschetti *et al*., 2009). Collectively, these examples point to the potential changes mediated by non‐native ecosystem engineers in both the abiotic and biotic components of the invaded ecosystem. Furthermore, in some cases, their influence extends to surrounding areas. For instance, reefs formed by non‐native oysters can have effects far beyond the reef structure itself (Ruesink *et al*., 2005). Similarly, dams built by non‐native beavers (*Castor canadensis*) can impact downstream areas and adjacent terrestrial areas due to changes in river flow (Henn, Anderson & Martínez Pastur, 2016). In many cases, native species may be negatively affected (e.g. species displaced by the new structure), while others may benefit from the introduction of ecosystem engineers (e.g. species that increase in abundance and biomass due to the presence of the new structure or new environmental conditions; Ilarri *et al*., 2012).

Some non‐native species can remove physical structures through their engineering activities or as they become dominant, leading to changes in ecological properties at the landscape scale (Crooks, 2002). Examples include the nutria (*Myocastor coypus*), the muskrat (*Ondatra zibethicus*), and the red swamp crayfish (*Procambarus clarkii*), which through grazing and burrowing activities can weaken riverbanks, accelerating erosion, and increasing the risk of flooding of dykes, as well as river and lake embankments (Gherardi, 2006; Bertolino & Genovesi, 2007; Bertolino *et al*., 2012; Haubrock *et al*., 2019). In extreme cases, invasions can alter erosion regimes, thereby altering habitat suitability for other species. Species such as *Sphaeroma quoyanum*, a small burrowing isopod native to Australia but introduced to the saltmarshes of San Diego and San Francisco bays, USA, create galleries that reduce sediment stability, increase erosion rates, and alter the sediment dynamics and flow (Talley, Crooks & Levin, 2001). In the marine environment, the burrowing activity of several non‐native invertebrates, such as *Marenzelleria* spp., *Mya arenaria*, *Anadara kagoshimensis*, *Anadara transversa*, and *Ruditapes philippinarum*, can enhance sediment oxygenation and promote the exchange of dissolved substances between the sediments and the overlying water column. These processes ultimately influence nutrient cycling, and can lead to increased sediment erosion and resuspension, resulting in significant alterations to both benthic and pelagic habitat structure and function (Katsanevakis *et al*., 2014).

In particular cases, non‐native species can also either introduce, enhance, or suppress disturbance regimes in both disrupted and intact systems, with cascading effects in ecosystem processes that influence community composition and structure (Vitousek, 1990; Mack & D'Antonio, 1998). For example, fire is a natural form of disturbance in many ecosystems and native plants often have a suite of characteristics that allow them variously to promote, resist, and thrive after fire. In addition to being resilient to losing much above‐ground biomass to fire, many plants have seeds whose germination success is increased after exposure to elevated temperatures or scarring. However, the establishment of non‐native plants can often alter the fire regime – affecting both the intensity and frequency of fires – or the introduced plants may be better adapted to fire than the species they are replacing (D'Antonio & Vitousek, 1992). Non‐native species can also influence geomorphological disturbance regimes (Mack & D'Antonio, 1998). Non‐native plant species such as *Acacia* spp. increase geomorphological disturbances through their uprooting during high flow periods (Macdonald & Richardson, 1986), while species with extensive root systems (e.g. stolon‐ or rhizome‐forming species) decrease geomorphological disturbances such as landslides and hill erosion (Mack & D'Antonio, 1998). In South African fynbos ecosystems, substrate stabilisation has driven up to a tenfold increase in above‐ground biomass, and the resulting rise in transpiration has reduced streamflow in affected catchments by approximately 50% (Van Wilgen & Richardson, 1985). The introduction of earthworms has also received attention in this regard, principally in North America where temperate and boreal forests previously lacked these organisms (Holdsworth, Frelich & Reich, 2007). Major alterations in geomorphological properties can result from the different strategies employed by earthworm species, which include: (*i*) physically disrupting the organic layer by consuming and mixing soil layers, leading to an homogenised organic forest floor, as seen in epigeic earthworms; (*ii*) mixing organic material with underlying minerals, as exhibited by endogeic earthworms; and (*iii*) removing surface litter by pulling it into the mineral layer and depositing casts of mixed organic and mineral material on the soil surface, a behaviour characteristic of anecic earthworms (Addison, 2009).

##### (v) Engineering of ecosystem functioning and biogeochemical processes

Ecosystem function refers to the capacity of natural processes and components to generate goods and services that support human needs, either directly or indirectly (De Groot, Wilson & Boumans, 2002). Ecosystem functions are considered a subset of broader ecological processes and encompass key processes such as primary and secondary production, nutrient cycling, pollination, and water regulation. These ecological processes arise from complex interactions between biotic and abiotic components of ecosystems, governed by the fundamental flows of matter and energy (Gutiérrez, Jones & Sousa, 2014). The introduction and establishment of non‐native species can substantially alter these functions and processes, underlining the far‐reaching consequences of the ecological disturbances brought about by non‐native species (reviewed in Ehrenfeld, 2010). Primary and secondary production (i.e. the formation of biomass by autotrophic or heterotrophic organisms), for instance, can be profoundly altered by non‐native species, either directly through the replacement of native autotrophs and heterotrophs, or indirectly by modifying other ecosystem components that, in turn, influence these organisms (Ehrenfeld, 2010). An increase in primary production in aquatic ecosystems can occur alongside seasonal blooms of non‐native phytoplankton species. For instance, *Coscinodiscus wailesii*, a large diatom invading the Atlantic brackish and marine waters from France to Norway, can constitute up to 90% of the total protist plankton biomass during blooms. Similarly, the invasion of the common reed (*Phragmites australis*) can lead to a significant increase, by an order of magnitude, in marshland aboveground primary production (Meyerson, Chambers & Vogt, 1999). The presence of non‐native plants in terrestrial ecosystems can also increase primary production (Ge *et al*., 2015; South *et al*., 2015; Helsen *et al*., 2018). For instance, North American mixed‐grass fragments invaded by the Eurasian perennial grass (*Agropyron cristatum*) exhibit increases in above‐ground biomass and litter production by 63% and 89%, respectively (Henderson & Naeth, 2005). Research on secondary production demonstrates that non‐native molluscs can contribute substantially to ecosystem biomass and alter ecosystem processes and functions. Examples include the New Zealand mud snail (*Potamopyrgus antipodarum*) (Hall Jr, Dybdahl & VanderLoop, 2006), the Pacific oyster and the Manila clam (*Ruditapes philippinarum*) (Ruesink *et al*., 2006), and the Asian clam (*Corbicula fluminea*; Sousa *et al*., 2008). These high rates of secondary production contribute to alterations in food webs, providing a new prey source for predators, although much of the production may enter the detritus food web (Sousa *et al*., 2008).

Other types of ecosystem engineers include chemical and light engineers (Berke, 2010). Chemical engineers modify the chemical properties of their environment. For example, the grasses *Spartina alterniflora* and *S. anglica* enhance sediment oxidation and microbial mineralisation in vegetated marshes (Gribsholt & Kristensen, 2002), the green alga *Caulerpa cylindracea* can form dense mats that promote anoxic conditions (Piazzi *et al*., 2007) and the gastropod *Crepidula fornicata*, through its mucous‐rich pseudofaeces, transforms sandy substrates into oxygen‐depleted mud with high organic content, which rapidly becomes anoxic (Thieltges, Strasser & Reise, 2006). Collectively, these species act as drivers of chemical transformation in marine sediments, reshaping benthic communities, trophic structures, and ecosystem functioning (Katsanevakis *et al*., 2014). The impact of non‐native species on chemical properties may also occur indirectly, e.g. when non‐native herbivores defoliate trees and modify tree composition, causing temporary or permanent modifications in ecosystem functions, such as carbon allocation and nitrogen cycling, or hydrological processes (Lovett *et al*., 2002, 2006; Ford & Vose, 2007). Several non‐native species act as light engineers, either reducing or enhancing light penetration and thereby altering ecosystem structure and function, primarily through biogeochemical processes related to energy flow and photosynthesis, although sometimes involving physical changes in water clarity. Many non‐native seaweeds, such as *Caulerpa taxifolia*, *Sargassum muticum*, *Lophocladia lallemandii*, and *Womersleyella setacea*, and non‐native freshwater floating plants, such as *Pontederia crassipes* and *Azolla filiculoides*, act as light competitors, restricting light to understorey native producers. Conversely, non‐native filter feeders, such as the Pacific oyster and zebra mussel, the gastropod *C. fornicata*, and the reef‐forming polychaete *F. enigmaticus*, can increase water transparency by reducing suspended particulates, thereby enhancing light penetration and supporting deeper colonisation by macrophytes (e.g. Fahnenstiel *et al*., 1995). Zebra mussels are known to filter feed large quantities of plankton from the water column in North American and European rivers and lakes. This both enhances water clarity and also mediates the transfer of pelagic resources into the benthos (Strayer *et al*., 1999; Gergs, Rinke & Rothhaupt, 2009). Other non‐native species can potentially modify the nutrient inputs (nitrogen and phosphorus) of invaded ecosystems, such as the aquatic fern *A. filiculoides*, which grows over the surface of the invaded fresh waters inducing a depletion in water quality (Pinero‐Rodríguez *et al*., 2021), or the tree *Cinnamomum verum*, which may accelerate nutrient cycling in the nutrient‐poor soils of the Seychelles, to the detriment of the less‐competitive native species (Kueffer *et al*., 2008). By contrast, the benthic foraging activities of non‐native common carp (*Cyprinus carpio*) result in the resuspension of sediments and uprooting of aquatic macrophytes, increasing turbidity, nutrient levels, and phytoplankton production (Britton, 2023) whereas massive aggregations of non‐native animals, such as the wels catfish (*Silurus glanis*), can create biogeochemical hotspots promoted through nutrient excretion (Boulêtreau *et al*., 2011).

#### (b) Ecological consequences of invasion impacts

##### (i) Individual level

Biological invasions can induce rapid and often maladaptive changes in the behaviour, feeding rates, growth, and reproduction of native species at the individual level, as they respond to novel pressures imposed by non‐native organisms (Carneiro *et al*., 2025). One of the most widely observed consequences is the alteration of antipredator behaviour in response to biological invasions (reviewed in Ruland & Jeschke, 2020). Native prey may either overreact to harmless non‐native species, resulting in unnecessary energy expenditure or reduced energy uptake, or underreact to real threats due to predator naïveté, increasing their vulnerability to predation (Carthey & Banks, 2014). Such responses may include changes in vigilance, escape tactics, or risk‐assessment strategies (Sih, Ferrari & Harris, 2011) and can not only alter the behaviour of native species, but also directly reduce their survival rates and reproductive success, ultimately leading to reduced individual fitness (e.g. Burns, 2013). Similarly, foraging behaviour can shift dramatically in the presence of non‐native competitors or predators, often leading to reduced foraging efficiency or increased shelter usage (Preisser, Bolnick & Benard, 2005). In the same vein, non‐native species can alter their behaviour in the presence of native competitors. For example, the non‐native American mink (*Neogale vison*) consumed less fish and more terrestrial prey and shifted its activity pattern from nocturnal to diurnal when coexisting with native competitors such as Eurasian otters (*Lutra lutra*) and polecats (*Mustela putorius*) (Harrington *et al*., 2009). Similar trophic shifts by native species in the presence of non‐native species have been observed for freshwater fishes (Rogosch & Olden, 2020).

Non‐native species can also disrupt habitat selection patterns, forcing native species into suboptimal environments and reducing shelter availability, increasing exposure to stressors, or elevating interspecific interactions (Sih *et al*., 2010; Carthey & Banks, 2014). Moreover, some behavioural responses to invasions, such as shifts in activity patterns or temporal niche use, reflect attempts by native species to minimise spatiotemporal overlap with non‐native species, whether competitors or predators (Gaynor *et al*., 2018). For instance, diurnal species may become more nocturnal to avoid non‐native predators with fixed activity cycles (Daly *et al*., 1992). Learning and behavioural plasticity play a critical role in mediating these responses, as species with higher cognitive flexibility may be better able to adapt to rapidly changing ecological contexts brought about by invasions (Sol *et al*., 2011). However, plasticity has limits, and when behavioural adaptation is insufficient or too slow, it can lead to population declines or local extinctions (Mooney & Cleland, 2001). Overall, behavioural shifts are a key dimension of invasion impacts, with consequences that ripple through population dynamics, community interactions, and ecosystem functioning.

##### (ii) Population level

At the population level, non‐native species can induce substantial shifts in abundance, density, biomass, and demographic structure of native populations (Carneiro *et al*., 2025), with cascading effects on their viability and ecological functions. These changes often result from direct mechanisms such as predation, herbivory, interspecific competition, parasitism and diseases, and hybridisation, as well as indirect mechanisms including apparent competition, habitat modification, and consequent changes in environmental factors or altered resource dynamics (Simberloff *et al*., 2013). Population declines may be gradual or abrupt, with some native species experiencing numerical reductions without local extinction, i.e. population suppression (Sax & Gaines, 2008). Such declines can impair key ecological functions, particularly when the affected species are dominant, keystone, or ecosystem engineers (Côté, Darling & Brown, 2016) resulting in cascading effects that lead to the decline or local extirpation of numerous other species belonging to different trophic levels (Rabenold *et al*., 1998; Mitchell *et al*., 2016). Some native populations may experience increases in abundance, either through release from competition or predation (enemy release effect) or due to facilitative interactions with non‐native species, which can also disrupt ecosystem balance (Keane & Crawley, 2002; Ilarri *et al*., 2012; Novais *et al*., 2015). Altered population densities can shift species' roles in trophic networks, for instance, by weakening top‐down or bottom‐up controls (Estes *et al*., 2011). Additionally, demographic changes, such as skewed age structures, reduced genetic diversity or reproductive output, or sex‐ratio imbalances, can further destabilise populations and reduce resilience to other stressors like climate change or habitat fragmentation (Salguero‐Gómez *et al*., 2015; Sousa *et al*., 2019). Non‐native species may also cause evolutionary shifts in life‐history traits (e.g. body size, dispersal ability, phenology), potentially leading to rapid but maladaptive responses (Phillips & Shine, 2004). Ultimately, population‐level changes serve as critical early indicators of broader community and ecosystem disruptions, underscoring their importance in both impact assessment and conservation prioritisation.

##### (iii) Community level

Non‐native species can drive significant changes in biodiversity at the community level (encompassing both individual species and species assemblages), causing range changes in native species and thereby affecting alpha (local), beta (compositional dissimilarity), and gamma (regional) diversity in complex and often contradictory ways (Haubrock & Soto, 2023; Carneiro *et al*., 2025). The introduction and establishment of non‐native species may initially increase local species richness (alpha diversity) by adding novel taxa, creating an illusion of biodiversity gain (Simberloff *et al*., 2013). However, these species richness increases, sometimes referred to as *pseudo‐richness*, can mask underlying ecological degradation, particularly when the arrival of non‐natives does not immediately lead to declines or extinctions of native species (i.e. invasion debt; Rouget *et al*., 2016). Such native species losses reduce true local diversity and homogenise community composition across sites, resulting in decreased beta diversity (McKinney & Lockwood, 1999). Even seemingly subtle changes in alpha and beta diversity can lead to profound ecological effects, such as altered soundscapes that may impair the reproductive fitness of native species (Hopkins, Edwards & Schwarzkopf, 2022). At the regional level, gamma diversity may increase, decline, or remain stable in response to biological invasions, depending on the balance between extinctions and introductions, especially when non‐native species become widespread and ecologically dominant (Sax & Gaines, 2008). For example, while many oceanic islands have doubled their plant gamma diversity following human colonisation due to introductions (Sax & Gaines, 2008), invasive predators such as snakes, rats, and cats have caused severe declines in endemic vertebrate species, often reducing alpha diversity and in some cases driving global extinctions (Blackburn *et al*., 2004; Clavero & García‐Berthou, 2005; Dueñas *et al*., 2021). Similarly, worldwide introductions of cosmopolitan fish species like largemouth bass (*Micropterus nigricans*) and common carp have led to a marked decline in beta diversity, with historically distinct river basins now sharing many of the same species (Rahel, 2000; Villéger *et al*., 2011; Marr *et al*., 2013).

Beyond taxonomic changes, invasions also affect the functional and phylogenetic dimensions of biodiversity. Non‐native species may occupy novel or redundant ecological roles relative to displaced native species, potentially leading to functional homogenisation and reduced phylogenetic distinctiveness, even in cases where overall species richness remains stable or increases (Olden *et al*., 2004; Winter, Devictor & Schweiger, 2013). For example, the global introduction of non‐native bird species has not compensated for the lost functional space caused by the extinction of insular birds; instead, these introductions have often resulted in the loss of functionally complementary species (Sobral, Lees & Cianciaruso, 2016). Non‐native species can also establish a higher number of interactions within the invaded community network than their native counterparts, therefore acting as keystone species; a role that can be difficult to identify, potentially exacerbating the threat they pose, and complicating management and eradication efforts (de Miguel *et al*., 2016; Rio‐Hortega *et al*., 2022). In addition to observed diversity, non‐native species may also influence *dark diversity*—the subset of species that are ecologically suitable for a region but are currently locally absent (Pärtel, Szava‐Kovats & Zobel, 2011; Pärtel *et al*., 2025). While non‐natives may increase observed gamma diversity, they can simultaneously widen the gap between the observed and potential species pools by displacing natives or modifying habitats, thus reducing the ecological suitability for previously present species (Lewis & Maslin, 2015). In this context, the discrepancy between observed diversity and dark diversity – shaped by the concurrent losses of native taxa and additions of non‐native ones – offers a nuanced lens to assess the legacy of invasions. Integrating taxonomic, functional, and phylogenetic aspects of both observed and dark diversity is crucial for revealing hidden biodiversity erosion beneath apparent gains and underscores the importance of multiscalar, multidimensional approaches in ecological impact assessments.

##### (iv) Ecosystem level

The introduction of non‐native species can result in profound changes at the ecosystem level (Carneiro *et al*., 2025; reviewed in Ehrenfeld, 2010), including the alteration of functions and services (Jeschke *et al*., 2014), the availability of habitat or refugia (Gallardo *et al*., 2016), and the abiotic environment (Zedler & Kercher, 2004). While changes in ecological functions and primary and secondary productivity can be considered as both an impact mechanism (i.e. a non‐native species introduces a new function or increases or decreases productivity; Simberloff *et al*., 2013) and an ecological impact (i.e. the presence of a non‐native species alters provided functions or decreases the productivity of an ecosystem; Estes *et al*., 2011), trophic cascades (i.e. changes in food webs; Sih *et al*., 2010) are a common consequence of biological invasions and can affect entire ecosystems. There are many examples of how invasion by non‐native predatory species results in strong top‐down effects, especially in cases with high ecological novelty. These impacts can be particularly devastating when the non‐native predator is a generalist capable of exploiting a wide range of prey, or when the prey species are small‐bodied, have low reproductive rates, or are behaviourally ill‐equipped to avoid novel predation pressures (Doherty *et al*., 2016).

Non‐native species can induce trophic cascades, where the effects of changes in the abundance of a species at a high trophic level affect multiple trophic levels (Terborgh & Estes, 2013). However, trophic cascades can also originate from bottom‐up impacts, where invasions affect lower trophic levels and subsequently influence higher levels of the ecosystem, potentially leading to major ecological changes. An interesting and intriguing example is the establishment of the zebra mussel and round goby (*Neogobius melanostomus*) in the Laurentian Great Lakes, where integration of these two species in the system is introducing biotoxins into the food web (Hebert *et al*., 2014; Essian *et al*., 2016). Zebra mussels filter water and accumulate toxins produced by the naturally occurring bacteria *Clostridium botulinum*. The toxin is consequently transferred to molluscivorous birds or first eaten by round goby and then transferred to piscivorous birds, most probably causing die‐offs of large numbers of waterbirds. Bottom‐up effects can profoundly influence entire ecosystems when foundation species, i.e. those that are spatially dominant and highly connected within ecological networks, are affected by non‐native species. By outcompeting and preying upon native ants, non‐native ants increased the vulnerability of trees to browsing by African elephants (*Loxodonta africana*), thus altering predator–prey dynamics (i.e. lions *Panthera leo* were less effective in killing zebra *Equus quagga*, due to an increase in landscape visibility) and resulting in ecosystem‐level impacts (Kamaru *et al*., 2024). A well‐documented case of a bottom‐up trophic cascade caused by a marine invasive species is the introduction of the ctenophore *Mnemiopsis leidyi* into the Black Sea during the 1980s *via* ballast water. It reached very high densities, primarily feeding on zooplankton, ichthyoplankton, and pelagic fish eggs, leading to a dramatic decline in zooplankton populations. This reduction in zooplankton disrupted the food web by decreasing the availability of prey for small pelagic fishes, which suffered massive population declines. The collapse of small pelagic fish stocks had cascading bottom‐up effects on higher trophic levels, including piscivorous fish and marine mammals, also severely impacting fisheries (Katsanevakis *et al*., 2014).

Non‐native species can also drive bottom‐up processes by providing a novel trophic subsidy for native species at higher trophic levels, such as non‐native crayfish species being an abundant food source for many native bird and mammal species (Beja, 1996; Tablado *et al*., 2010) or when non‐native crayfish were the main prey of native snakes in a pond community (Stellati *et al*., 2019; Bissattini *et al*., 2021). Trowbridge (2004) also reported two introduced subspecies of the alga *Codium fragile* being preferred over native *Codium* species by herbivorous sea slugs after a few years of introduction. Similarly, non‐native cane toads (*Rhinella marina*) exert significant impacts on native predator populations through the production of bufotoxins – potent chemical compounds to which many vertebrates lack evolutionary exposure, resulting in high mortality upon ingestion (Shine, 2010).

The disruption of green food webs (ecological networks based on plants or algae; Odum, 1969) by altering plant–herbivore–predator interactions, outcompeting native species, or introducing new predators and diseases, is very well studied and quantified (Ehrenfeld, 2010). By contrast, the influence of non‐native species on brown food webs (ecological networks based on dead organic matter or detritus; Odum, 1969), which involve decomposition and nutrient cycling, is largely overlooked (Van der Putten, Klironomos & Wardle, 2007; Ehrenfeld, 2010). These belowground terrestrial and aquatic networks, driven by fungi, bacteria, and detritivores, are equally crucial for ecosystem health (Grossart *et al*., 2019). Non‐native species can significantly alter brown food webs by changing the quality or quantity of organic matter input, outcompeting native decomposers, or introducing novel interactions. For example, non‐native plants may produce litter that decomposes more slowly or releases allelopathic compounds, inhibiting microbial activity (Van der Putten *et al*., 2007), non‐native earthworms can change decomposition rates leading to nutrient imbalances and shifts in soil communities (Bohlen *et al*., 2004c), and non‐native microbes can also change decomposition processes although with possibly low impact due to their limited specificity and great functional redundancy (Van der Putten *et al*., 2007). Invasions by nitrogen‐fixing species like the firetree (*Morella faya*) and *Acacia* spp. can dramatically alter nutrient cycling and native forest structure by increasing soil nitrogen (Yelenik, Stock & Richardson, 2007; Marchante *et al*., 2008; Castro‐Díez *et al*., 2009, 2014), while non‐fixing invaders such as Monterey pine (*Pinus radiata*) and beach rose (*Rosa rugosa*) can indirectly affect soil properties through changes in land use and vegetation structure (Amiotti *et al*., 2000; Vanderhoeven, Dassonville & Meerts, 2005; Helsen *et al*., 2021; Woch *et al*., 2023). Similarly, dense mats of the ice plant (*Carpobrotus edulis*) can alter ecosystem function by increasing litter accumulation, modifying soil chemistry (e.g. pH and calcium levels), and reducing light and water availability, which impairs native plant growth and seedling establishment (D'Antonio & Mahall, 1991; D'Antonio, 1993; Molinari *et al*., 2007). Despite their possible ecological significance, the impact of non‐native species on brown food webs remains under‐researched and requires future attention.

A prominent ecosystem consequence of non‐native species is a change in the availability of habitats or refugia. These encompass both declines, when, for instance, non‐native crayfish outcompete native crayfish for shelter (Twardochleb, Olden & Larson, 2013), but also cases where species such as zebra mussel create dense mussel beds, thereby providing habitat and shelter for other Ponto‐Caspian species like killer shrimp (*Dikerogammarus villosus*; Gergs & Rothhaupt, 2008). More complex are ecosystem‐level impacts, including changes in the fire regime, hydrology, nutrient availability, microclimate, and soil structure (Brooks *et al*., 2004; Gaertner *et al*., 2014; Catford, 2017; Garcia & Clusella‐Trullas, 2025). Non‐native species can alter or even eliminate the soil structure, leading to cascading effects on nutrient cycles that influence the distribution and retention of carbon, nitrogen, and phosphorus (Bohlen *et al*., 2004a,b), and change the community composition (Bohlen *et al*., 2004c; Peltzer *et al*., 2010). It is also important to note that boreal forests, for instance, contain significant amounts of dead organic matter, making them a key carbon sink (Peltzer *et al*., 2010). Therefore, the potential for non‐native earthworms to release nutrients or reduce soil carbon storage in the topsoil layers could have substantial implications for the global carbon cycle (Alban & Berry, 1994). Grasses, such as cheatgrass (*Bromus tectorum*) in the western USA, and Colombian bluestem (*Schizachyrium condensatum*) and molasses grass (*Melinis minutiflora*) in Hawai'i have increased fire frequency and intensity, resulting in drastic changes in the structure and species identity of the invaded ecosystems (D'Antonio & Vitousek, 1992; Brooks *et al*., 2004). The addition of non‐native trees into South African fynbos shrublands has also influenced the fire regime, increasing fuel load and fire intensity (Richardson & Higgins, 2000). Conversely, non‐native species can also decrease fire frequency and intensity in fire‐maintained ecosystems (Doren & Whiteaker, 1990). Changes in water regimes may also take place after the introduction of non‐native plants. For example, the introduction of salt cedar (*Tamarix* spp.) has replaced much of the native riparian vegetation of the western USA, where it consumes large quantities of water, narrows river channels, increases soil solutes, enhances productivity, and increases surface litter and salts (Zavaleta, Hobbs & Mooney, 2001). Additionally, the introduction of non‐native plants could substantially alter micro‐climatic conditions, with severe consequences for other species (Garcia & Clusella‐Trullas, 2025).

#### (c) Co‐invasive symbionts: impacts of commensals, parasites and pathogens

Symbiotic organisms remain a relatively understudied dimension of biological invasions. As invaders in their own right, but also as potential forces of impact, we include them here in a stand‐alone subsection to emphasise their independent agency, while recognising that their effects also permeate the broader ecological mechanisms discussed in this review. We chose this structure deliberately to highlight that co‐invasive symbionts can generate impacts through multiple pathways: sometimes directly as invasive entities, sometimes indirectly *via* interactions with their hosts. Accordingly, we treat their dynamics, negative effects, and positive effects in separate subsections below, to reflect both their complexity and their distinct role in invasion processes.

##### (i) Dynamics behind parasite introductions leading to impact

Biological invasions will, in all cases, be associated with microbial species (Bojko, Dunn & Blakeslee, 2023), and microbes themselves can also be considered non‐native organisms (Nuñez, Pauchard & Ricciardi, 2020). Species generally house a vast symbiotic microbial diversity, collectively referred to as its ‘microbiome’, ‘symbiome’, or ‘pathobiome’, which includes bacteria, archaea, viruses, and other microorganisms that can act as mutualists, commensalists, or parasites (Bass *et al*., 2019). The microbial diversity housed by a given host is not static and may undergo shifts in its microbiome composition depending on diet (Zmora, Suez & Elinav, 2019), habitat (Holt *et al*., 2020), and susceptibility to infection by a parasite or pathogen (Bass *et al*., 2019). Because organisms evolve alongside their microbiomes in natural settings, understanding how this relationship changes during biological invasions has become an important area of research. This has implications for pathogenic risks to humans (Juliano & Philip Lounibos, 2005; Roy *et al*., 2023c), cultured species (Wood *et al*., 2023), and wildlife (Roy *et al*., 2017; Bezerra‐Santos *et al*., 2023), as well as for advancing core concepts in disease ecology (Ogden *et al*., 2019). As a non‐native species travels, arrives, and establishes in a new location, it will carry with it a symbiotic complement. However, it is unlikely that this complement will house all of the co‐evolved symbionts that it would naturally interact with in its own native range. If co‐evolved members of the native symbiosis are left behind in the native range, but were pathogenic to the invader, it will have escaped their negative influence and have undergone ‘enemy release’ (Miura & Torchin, 2023). Arrival of symbionts with the non‐native propagule can have several outcomes: (*i*) the co‐non‐native symbiont may persist in the non‐native host; (*ii*) the symbiont may not persist and instead be lost from the new population over time; (*iii*) the co‐non‐native symbiont may be replaced by a native microbial species in the newly invaded environment; (*iv*) if parasitic, the co‐non‐native symbiont may transmit to congeners or native species (spillover) in the newly invaded environment; and (*v*) if capable of infecting native species, it may also ‘spillback’ into the non‐native population (Dunn *et al*., 2012; Hatcher, Dick & Dunn, 2012). Some variations of the above hypothesis are expected, since different symbionts have different driving factors influencing their transmission and persistence.

Once a non‐native species has established and its population begins to grow, there are further opportunities for symbionts from the native range to utilise it as a resource, often increasing the abundance of a native parasite beyond what typically would be expected (Dunn *et al*., 2012; Hatcher *et al*., 2012). Pathogen spillover and spillback can play a key role in the establishment, spread, and impact of a non‐native species (Roy *et al*., 2017). For instance, pathogen spillover facilitated the success of the non‐native Eastern grey squirrel (*Sciurus carolinensis*), which introduced a pox virus (largely asymptomatic in the invader) as a biological weapon against the native red squirrel (*Sciurus vulgaris*), causing native population declines up to 25 times faster in areas where the co‐introduced squirrel pox virus was present, as it is largely resistant to this virus (Rushton *et al*., 2006). Similarly, amphibian invasions are often associated with increased pathogen diversity, higher prevalence and infection intensity, as well as reduced host fitness due to pathogen exposure and infection (Atkinson & Savage, 2023). The introduction of two fungal species causing chytridiomycosis through the amphibian pet trade has caused the decline of at least 501 amphibian species over the past half‐century, including 90 presumed extinctions (Scheele *et al*., 2019). In addition, ranaviruses (highly infectious pathogens affecting amphibians, reptiles, and fish) have been increasingly linked to the presence of non‐native amphibian species that act as reservoirs and vectors for disease transmission (Sharifian‐Fard *et al*., 2011; Price *et al*., 2014; Peñafiel‐Ricaurte *et al*., 2023; Campião *et al*., 2024). Co‐introduced symbionts are therefore increasingly recognised as unavoidable components of the ecological impact of biological invasions, with consequences manifesting across all levels of biological organisation (Carneiro *et al*., 2025), leading to either a negative or positive influence upon their host's invasion success and potential for impact.

##### (ii) Negative effects

The negative influence of parasites on invaded ecosystems is a commonly observed phenomenon and can manifest in several ways. These include direct competition with native microbial flora (including both pathogenic and innocuous species) for resources, direct infection of native species resulting in mortality and consequent loss of ecosystem services, and indirect effects by affecting their invasive non‐native hosts or infecting native hosts without imposing mortality. Alternatively, all of the above can take place at once, or an invasive symbiont may only affect its non‐native host.

The introduction of co‐invasive parasites can alter local host abundance and overall species diversity, with the capability to impact the structure and functioning of ecosystems (Britton, 2013). Several examples have been reported, where dramatic change to host species diversity is evident after the arrival of an infectious disease, such as ash dieback, the American chestnut blight, Dutch elm disease, chytridiomycosis, avian pox, avian malaria, rinderpest virus, canine distemper virus, and crayfish plague. For instance, the African rinderpest epidemic decimated approximately 90% of East African domestic cattle and 95% of the African buffalo (*Syncerus caffer*) and wildebeest (*Connochaetes taurinus*) (Spinage, 2012). Following rinderpest control, wildlife populations rapidly recovered, leading to increased densities of carnivores such as lions and hyenas (*Crocuta crocuta*). Conversely, decreases in the abundance of gazelle (e.g. *Eudorcas thomsonii*) was evident, resulting from a higher predation pressure and the almost extirpation of wild dogs (*Lycaon pictus*), possibly driven by intensified competition with lions and hyenas.

Negative effects can also result from the infection of one or more native species, incurring higher mortality rates in the population, as observed with the crayfish plague caused by the oomycete *Aphanomyces astaci* (Svoboda *et al*., 2017). This aetiological agent was introduced to Europe in the second half of the 19th century, triggering massive outbreaks in native crayfish populations (Holdich *et al*., 2009). The resulting population declines and the accompanied collapses of fisheries stimulated introduction of mainly North American crayfish species, which were later discovered to be asymptomatic carriers of the disease (Jussila *et al*., 2021). To sustain itself, the parasite reduced in virulence, and at least some populations of native species partly adapted. As a result, chronically infected native crayfish populations have been recently documented, albeit these reports remain rare (Ungureanu *et al*., 2020; Mojžišová *et al*., 2022). On the contrary, under certain conditions, crayfish plague can re‐emerge as a deadly disease even in North American crayfish populations that were temporarily free of this parasite and thus lost their originally strong immunity (Thomas *et al*., 2020; Boštjančić *et al*., 2022). The ecological impact of a non‐native host can be exacerbated when it carries a non‐native disease, the so‐called ‘novel weapon hypothesis’ (Callaway & Ridenour, 2004), where the invasive host acts as a vector, introducing the parasite to native species and causing their decline, thereby reducing competition and amplifying its negative ecological impact.

##### (iii) Positive effects

Non‐native hosts are often considered to have an overall negative impact on an ecosystem (Carneiro *et al*., 2024b); however, this is not necessarily the case for the parasites and pathogens they may carry. In many instances, a symbiont carried by a non‐native host can induce behavioural changes, cause the death of the host, or regulate the non‐native host population, which can lead to an overall reduction in its impact and therefore mitigate damage to the ecosystem (a phenomenon likened to ‘natural biological control’; Torchin & Mitchell, 2004). Examples include demon shrimp (*Dikerogammarus haemobaphes*), which is regulated by a combination of microsporidian and viral pathogens, where the viruses appear specific to this host (Bojko *et al*., 2019). This mitigating factor is considered a strong positive effect, linked to biological control, where parasites and pathogens are used to control pests, for example insects in agriculture (Lacey *et al*., 2015). Similarly to biological control, an overall positive effect must balance with the capacity for the parasite to infect and harm other species. In instances where a parasite is introduced alongside an invasive host, and it reduces the host's impact but does not cause other direct or indirect impacts (e.g. infecting native species and therefore reducing the ecosystem services it provides), one may consider the parasite to have an overall positive controlling effect on its invasive host. In the case of a non‐competent or less competent non‐native host, dilution may occur, potentially benefiting native species by reducing infection prevalence in the principal host. This has been observed with Lyme disease in North America, where diverse vertebrate communities lower *Borrelia* transmission from the highly competent white‐footed mouse (*Peromyscus leucopus*), and in Ireland, where the invasive bank vole (*Myodes glareolus*), likely a non‐competent host, reduced *Bartonella* prevalence in native wood mice (*Apodemus sylvaticus*) (Ostfeld & Keesing, 2000; Telfer *et al*., 2005; Levi *et al*., 2016).

There are several examples throughout nature where a co‐introduced parasite has mitigated the behaviour or population size of its host. In aquatic systems, studies with the non‐native amphipod *D. haemobaphes* have shown that this invader translocated a wide array of symbiotic species during its invasion (Hatcher *et al*., 2019). Further study into the effects of these parasites on the activity and survival of this host highlighted a reduction in both activity and survival (Bojko *et al*., 2019). In detail, the microsporidian parasite *Cucumispora ornata* (Bojko *et al*., 2015) infects the musculature of the host, among other tissues, and causes a significant decrease in the infected amphipods' capacity for movement and activity. A viral pathogen carried by *D. haemobaphes*, identified as ‘Dikerogammarus haemobaphes mininucleovirus’ has been shown to cause rapid mortality (Bojko *et al*., 2019; Subramaniam *et al*., 2020). This combination of reduced activity and survival is considered to limit the impact of this invader, therefore helping to conserve the natural ecosystem into which the host and parasite were introduced. This is relative to the high ecological impact caused by the sister species, the killer shrimp, which carried few parasites to the UK and remains largely parasite‐free (enemy release; Bojko *et al*., 2013), while populations of *D. villosus* on continental Europe have maintained their relatively high parasite diversity (Wattier *et al*., 2007).

In terrestrial ecosystems, examples exist of parasites and pathogens affecting non‐native plants (Roberts *et al*., 2022), vertebrates (Chinchio *et al*., 2022), and invertebrates (Hajek, Gardescu & Delalibera, 2021). A lucrative area of pest control lies within the invasive weed control sector, where pathogens of invasive plants are commonly used to reduce their impacts on crops or native vegetation; plant viruses, for example, can reduce plant competitiveness, growth, and survival (Roberts *et al*., 2022). Outside of direct pest control by reintroducing parasites to invasive populations that have escaped them, an example of an invasive terrestrial species that has carried a parasite alongside its invasion is the tawny crazy ant (*Nylanderia fulva*), and its microsporidian parasite (*Myrmecomorba nylanderiae*), which reduces the development rate of pupae, altering the speed at which ant colonies grow, and indirectly slowing the environmental impacts imposed by the ants (Plowes *et al*., 2015; LeBrun, Ottens & Gilbert, 2018).

### (2) Economic impacts

Among the known types of non‐native species impacts, economic consequences have historically been under‐quantified and inconsistently assessed by invasion scientists. This is despite the recognition of substantial economic damage caused by non‐native pests to key agricultural and forestry sectors in the late 19th century (Planchon, 1874; Perpillou, 1933; Clausen, 1978; Brunel *et al*., 2013). Economic impact assessments for plant health (Wightman, 1979; Hare, 1980; Kingsolver, Melching & Bromfield, 1983) and forestry sectors (White & Schneeberger, 1981) have since become routine for actual (Farnsworth *et al*., 2017; Eschen *et al*., 2021; Tambo *et al*., 2023) and prospective impacts (Soliman *et al*., 2010; Kenis *et al*., 2017). However, the first widely cited work that addressed damages incurred by non‐native species in monetary terms more broadly was Pimentel, Zuniga & Morrison (2005), which was deemed biased due to methodological inconsistencies, speculative extrapolations, and weak traceability of estimates (Hoffmann & Broadhurst, 2016). Several types of impacts, such as loss of native biodiversity, reduction in ecosystem services (e.g. pollination, water purification), and shifts in cultural values tied to landscapes, remain difficult if not impossible to quantify in monetary terms, and non‐market costs thus remain underrepresented due to valuation difficulties and methodological constraints that limit our ability to quantify these costs fully (Ahmed *et al*., 2023). Yet, these impacts can erode natural capital in ways that impose long‐term liabilities on societies and economies, manifesting as increased costs for water treatment, reduced agricultural productivity, and heightened vulnerability to natural disasters – effects with downstream economic implications. Over time, these liabilities can accumulate, placing substantial financial burdens on future generations. Moreover, if these introductions are not addressed promptly, the costs can escalate exponentially, as outlined in Ahmed *et al*. (2022) and Henry *et al*. (2023).

In recent years, methodological advancements have enhanced our capacity to assess and quantify the economic impacts of non‐native species. Notably, the application of choice experiments has allowed for more precise valuation of non‐market costs, proving particularly helpful in gauging public willingness to pay for the preservation of ecological functions or for measures to prevent non‐native species introductions (e.g. Rajmis, Thiele & Marggraf, 2016). With the recent development of the *InvaCost* database (Diagne *et al*., 2020), a first standardised, traceable, and global synthesis of the monetary costs associated with non‐native species was created, allowing researchers and policymakers to access sources of data (from peer‐reviewed to grey literature) on the monetary burden of invasions across spatial, sectoral, and taxonomic scales. From this database, economic impacts were assessed for numerous taxonomic groups, including e.g. fish, crustaceans, and bivalves (Haubrock *et al*., 2022b,d; Kouba *et al*., 2022), aquatic macrophytes (Macêdo *et al*., 2024), trees (Fernandez *et al*., 2023), ants (Angulo *et al*., 2022), birds (Evans *et al*., 2023), feral animals (Soto *et al*., 2024b), and, among others, herpetofauna (Soto *et al*., 2022). Notably, the cumulative global cost of biological invasions documented in *InvaCost* already surpasses USD $2 trillion (in 2017 values). Yet, this number is likely a conservative estimate due to underreporting and gaps in data availability, especially from the Global South (Henry *et al*., 2023; Soto *et al*., 2025). This compiled estimate included both damage costs and monetary losses – such as those affecting agriculture, fisheries, infrastructure, and forestry – and management costs (e.g. for surveillance, control, containment, and eradication efforts), which have been shown to reduce damage costs significantly when implemented early and strategically (Leung *et al*., 2002), as delays in intervention often escalate damages and require exponentially higher expenditures later (Ahmed *et al*., 2022).

While the bulk of the literature (and all information compiled in *InvaCost*) focuses on negative economic impacts, certain non‐native species can generate positive economic outcomes in specific sectors (Kourantidou *et al*., 2022). These include timber (Castro‐Díez *et al*., 2019), aquaculture, and commercial harvesting (Oficialdegui *et al*., 2025), but also tourism (Subalusky *et al*., 2023) and benefits to recreation. However, such benefits are highly context specific, often short term, and tend to accrue to different stakeholders than those bearing the costs (Carneiro *et al*., 2024a). For instance, while the aquaculture industry may profit from the farming of species such as Atlantic salmon (*Salmo salar*) or red swamp crayfish outside their respective native ranges, the environmental and economic burdens (e.g. on local fisheries, ecosystems, and water management) if these species escape are disproportionately borne by local communities (Oficialdegui *et al*., 2025). This disconnect highlights a fundamental issue of distributional injustice: the economic ‘winners’ (i.e. those benefiting from non‐native species) are often corporations or actors located far from the invaded ecosystems, whereas the economic ‘losers’ include local populations, public budgets, and biodiversity itself (Reaser *et al*., 2007). Moreover, benefits are frequently path dependent, emerging predominantly because ecosystems have already been altered or degraded, masking deeper systemic costs such as lost ecosystem functions or services (Lant, Ruhl & Kraft, 2008). In the eastern Mediterranean Sea, a hotspot of climate‐driven local extinctions and native biodiversity decline (Givan *et al*., 2018; Albano *et al*., 2021; Nikolaou & Katsanevakis, 2023), thermophilic Suezian (‘Lessepsian’) species introduced from the tropical Red Sea are thriving and sustaining key ecosystem functions and services (Tsirintanis *et al*., 2022), including constituting approximately three‐quarters of the catches in Israeli trawl fisheries, with several species regarded as a boon to the fishing industry (Katsanevakis *et al*., 2025).

Finally, the socioeconomic context significantly influences the economic impacts of non‐native species. Regions with limited financial resources, weak governance or lower levels of biodiversity awareness are often less equipped to manage and mitigate these impacts effectively (Bradshaw *et al*., 2024). Such economic disparities can lead to uneven abilities to respond to non‐native species and invest in management or biosecurity, often exacerbating the challenges faced by vulnerable human communities.

### (3) Social, cultural, and human health impacts

Human societies and biodiversity are deeply intertwined. Biodiversity sustains human well‐being in numerous ways, from food production and regulation services to recreational activities, artistic inspiration, and spiritual practices (Haines‐Young & Potschin‐Young, 2018). Biological invasions can therefore affect social and cultural practices in both negative and positive ways. For example, non‐native species may pose risks to human health and displace culturally important native species (Nuñez, Dimarco & Simberloff, 2018; Nuñez *et al*., 2020). The non‐native box tree moth (*Cydalima perspectalis*) causes the decline of box trees (*Buxus* spp.), which had important cultural and religious consequences in the Eastern Black Sea region (Mitchell *et al*., 2018). At the same time, non‐native biodiversity can foster positive cultural shifts, such as creating new ways of interacting with nature. An example could be sport fishing in Patagonia (Argentina and Chile) for non‐native salmonids like rainbow trout, which negatively affect native fish through predation and competition, but also generate significant revenues through the tourism (including ecotourism) industry in the region and thus, affect local traditions and livelihoods (Pascual *et al*., 2007). A recent example from Northern Norway demonstrates how the spread of pink salmon (*Oncorhynchus gorbuscha*) is reshaping local fishing cultures traditionally centred on the iconic Atlantic salmon, by making salmon fishing more accessible. While their ecological impacts remain under scrutiny, their presence is already sparking debates about species belonging, cultural identity, and the future of recreational fisheries in the region (Guay *et al*., 2024). Additionally, over time, species perceptions can shift, fostering naturalisation and cultural integration (Gaertner *et al*., 2017). For instance, the little owl (*Athene noctua*) in Great Britain shifted from being perceived as a threat in the 1930s to a cherished species among ornithologists today (Rotherham, 2021). Similar dynamics occur globally, where introduced species have become culturally central: bananas in Ecuador, coffee in Colombia, and cannabis in Jamaica exemplify how non‐native species can be recast as cultural keystone species (Nuñez & Simberloff, 2005).

Historically, several non‐native species were intentionally introduced for cultural and societal purposes, such as enhancing the aesthetics of gardens (as perceived by colonisers upon their return; Hoyle, Hitchmough & Jorgensen, 2017) or establishing new lines of food or fibre production. This is the case, for example, with numerous plants and birds introduced into European gardens or to the Americas during colonial times (Crosby, 2003, 2004). Some of these species eventually escaped and became established in natural ecosystems, leading to long‐term ecological transformations that remain measurable today (Lenzner *et al*., 2022). Cultural impacts are also often linked to traditional human foraging activities and cuisine. For instance, several non‐native marine species can affect traditional fisheries and other sectors (e.g. tourism) by reducing the occurrence and abundance of native species, including their economic viability. A recent example is the arrival of two Portunidae blue crab species, *Callinectes sapidus* and *Portunus segnis*, in the Mediterranean, which affected traditional fisheries, especially small‐scale ones (Marchessaux *et al*., 2023; Gavioli *et al*., 2025). In response to its rapid spread, the Italian government has promoted its consumption, so both species are increasingly incorporated into traditional Italian cuisine with a positive attitude toward blue crab consumption in Apulia (Frem *et al*., 2024) but with no tangible return in the northern Adriatic, where the invasion had catastrophic effects on e.g. Manila clam farming (Chiesa *et al*., 2025). Similarly, recent invasions of pelagic *Sargassum* spp. in the tropical Atlantic show diverse socio‐cultural impacts. Excessive blooms and beaching events significantly disrupt tourism and local livelihoods due to their unpleasant smell and potential health impacts (e.g. skin irritation from decomposition; Dominguez Almela *et al*., 2023b). However, there are emerging economic and cultural opportunities through the valorisation of *Sargassum* biomass in products like fertilisers or biofuel, reflecting adaptation and innovative responses to non‐native species (Dominguez Almela *et al*., 2023a). Nowadays, many ‘Cultural keystone species’, i.e. species that are culturally outstanding and that characterise the identity of a cultural group, are indeed non‐native species (Nuñez & Simberloff, 2005). For example, cattle (*Bos taurus*) introduced into Latin America during European colonisation, is now deeply embedded in regional identities, being a core symbol in the Amazonian ‘Boi Bumbá' folk festival as a symbol of cultural and spiritual heritage (Silva, 2022). Similarly, mango (*Mangifera indica*) trees introduced from southeastern Asia to many colonial cities are not only key species for landscaping, but also defining urban identity: Belém, an Amazonian city in northern Brazil, is known as the ‘City of the mango trees' (Loureiro & Barbosa, 2010). Additionally, religious practices, such as Buddhist ‘mercy release' ceremonies, have historically contributed to the intentional introduction of non‐native species, as adherents release captive animals into the wild for spiritual merit – a tradition widespread across East and Southeast Asia and increasingly practiced in Western countries by migrant communities (Liu, McGarrity & Li, 2012; Liu *et al*., 2013; Stringham & Lockwood, 2018).

Despite their ecological harm, numerous non‐native species have been embraced in cultural narratives, sometimes making their removal controversial (Oficialdegui *et al*., 2019), leading to changes in how people engage with nature for recreational and artistic purposes. This intersection between ecology and culture highlights how non‐native species, even as ecological threats, can become embedded in human identity and artistic expression (Nuñez & Simberloff, 2005), sometimes even obscuring their status as non‐native (Cordeiro *et al*., 2020; Jarić *et al*., 2024). For example, old introductions could be culturally accepted, potentially resulting in the disregard of scientific evidence concerning their potential long‐term negative impacts (Florencio, Lobo & Bini, 2019) or even leading to their inclusion as targets for conservation efforts (Clavero, 2014). Colourful birds, even if non‐native, can attract the attention of birdwatchers and nature enthusiasts, potentially enriching local wildlife experiences and generating income through tourism. Similarly, urban parks dominated by non‐native, fast‐growing tree species can provide valuable and accessible green spaces within densely built environments. These parks may significantly enhance human well‐being by offering aesthetic and recreational benefits, mitigating urban heat island effects, and improving air quality (Almas, 2017). Moreover, non‐native species can influence artistic endeavours by shaping cultural landscapes and inspiring artistic movements. For instance, *Eucalyptus* trees, introduced from Australia to California in the 19th century, became a defining feature of the state's environment and even gave rise to the Eucalyptus School, a major artistic movement that depicted landscapes dominated by these trees (Moure, 1982). Similarly, the European periwinkle snail (*Littorina littorea*), introduced to North America in the 19th century, initially transformed the coastal ecosystems of Maine and has since extended its range, which subsequently became a central theme in local paintings and literature. Cultural representations of non‐native species also extend to national media, including movies, toys, video games, and memes, possibly shaping public perception. The red‐eared slider turtle (*Trachemys scripta elegans*), a non‐native species associated with the pet trade, was normalised in Japan through widespread cultural exposure as toys (Lovich & Yamamoto, 2016; Wong, 2024). Many non‐native species have also been incorporated into local pharmacopoeias, frequently being used as remedies, tonics, and herbal treatments (Stepp & Moerman, 2001; Siqueira *et al*., 2018). For instance, the painted nettle (*Coleus barbatus*) is widely used in Brazilian traditional communities for diseases in the digestive system (Baptista *et al*., 2013; Siqueira *et al*., 2018) and the Madagascar periwinkle (*Catharanthus roseus*) is used to treat sexually transmitted diseases in South Africa (Semenya, Potgieter & Erasmus, 2013).

There are also less‐positive sides to these phenomena. Biological invasions have significant and multifaceted impacts on human health; the range of effects can include both direct physical suffering through to effects upon mental health, along with the facilitation of disease transmission (Donovan *et al*., 2013). The range expansion of species that evoke biophobia, such as spiders, snakes, and other animals often perceived as threatening, may lead to psychological discomfort and heightened fear of nature, a seemingly growing phenomenon in recent times, often hyperbolised by media reports (Mammola *et al*., 2020; Correia & Mammola, 2024). This, in turn, could contribute to a growing sense of disconnection from the natural world, further exacerbating the human–nature divide (Soga *et al*., 2023). Problematic non‐native species, such as the zebra mussel, can cause injuries to swimmers and fishers, while venomous marine invaders like the striped eel catfish (*Plotosus lineatus*) and the silver‐cheeked toadfish (*Lagocephalus sceleratus*) present serious health risks (Galanidi, Zenetos & Bacher, 2018; Galil, 2018). Additionally, allergenic and toxic plants, such as the common ragweed (*Ambrosia artemisiifolia*) and the giant hogweed (*Heracleum mantegazzianum*), trigger severe allergic reactions and dermatitis, affecting humans in newly invaded regions (Déchamp, 1999; Klimaszyk *et al*., 2014). Furthermore, aggressive non‐native insect species can have severe direct impacts on human health, including death (Nentwig, Mebs & Vilà, 2017). For example, the red imported fire ant (*Solenopsis invicta*) is responsible for numerous cases of painful stings and life‐threatening anaphylaxis, particularly in North America and China (Jemal & Hugh‐Jones, 1993; Prahlow & Barnard, 1998; Zhang *et al*., 2007; Xu *et al*., 2012). Similarly, non‐native species like the lionfish *Pterois miles* not only threaten native fish populations with the consequent cascading economic impacts on local fisheries and communities dependent on marine resources but also present a threat to humans (Mitchell & Dominguez Almela, 2025). In the Levantine Sea, massive swarms of the jellyfish *Rhopilema nomadica* have caused numerous hospitalisations of swimmers and fishers due to their painful stings, negatively impacting tourism revenues by threatening swimmer safety and deterring coastal visitation (Tsirintanis *et al*., 2022). Beyond direct injuries, biological invasions play a crucial role in the spread of infectious diseases. Non‐native mosquitoes (e.g. *Aedes* spp., *Anopheles stephensi*, *Culex quinquefasciatus*) have facilitated the transmission of malaria, dengue, chikungunya, and Zika virus, among others, in newly invaded areas (Juliano & Philip Lounibos, 2005; Romi *et al*., 2018; Roy *et al*., 2023a; Yan, Mackay & Stone, 2024). Similarly, non‐native gastropods like the giant African land snail (*Lissachatina fulica*) serve as intermediate hosts for rat lungworm (*Angiostrongylus cantonensis*), a parasite that can cause severe neurological damage in humans (Iwanowicz *et al*., 2015; Barratt *et al*., 2016). Non‐native plants such as mesquite (*Prosopis juliflora*) and the parthenium weed (*Parthenium hysterophorus*) further exacerbate the problem by providing suitable habitats for malaria‐carrying mosquitoes, thereby increasing transmission potential (Nyasembe *et al*., 2015; Tyagi *et al*., 2015; Muller *et al*., 2017).

### (4) Temporal dynamics of impacts

Invasion impacts are not necessarily static; rather, they can be subject to substantial variability in their magnitude and trajectories over time, complicating their assessment. Temporal changes often reflect shifts in human perception or values (Strayer *et al*., 2006), but also in population densities and biomass, which can strongly influence impact strength (Yokomizo *et al*., 2009; Jackson, Ruiz‐Navarro & Britton, 2015). Since invasion impacts stem from the consequences of individuals within a population, it is the intertwined nature of ecological interactions, environmental factors, and species‐specific traits that modulate invasion impacts (Haubrock *et al*., 2024b). As modern viewpoints recognise these numerous contextual factors influencing biological invasions, it is increasingly acknowledged that invasion processes may be more accurately described as components of an ‘adaptive network’ – a system in which populations of non‐native species and their interactions co‐evolve in response to ecological change (Blackburn *et al*., 2011; Soto *et al*., 2024a). A critical shortcoming of impact‐based classification of non‐native species is therefore that it disregards the population level, as established populations might not currently cause significant harm but could do so under different environmental conditions (i.e. ‘sleeper populations’; Spear *et al*., 2021). For example, species whose impact is presently considered to be benign could become a threat when they spread or due to climate change, altered land use, or ecosystem degradation (Crooks, 2005). The failure to account for potential time lags associated with impacts further undermines the effectiveness of enacted policies, making them reactive rather than proactive (Crooks, 2005).

**Fig. 5:**
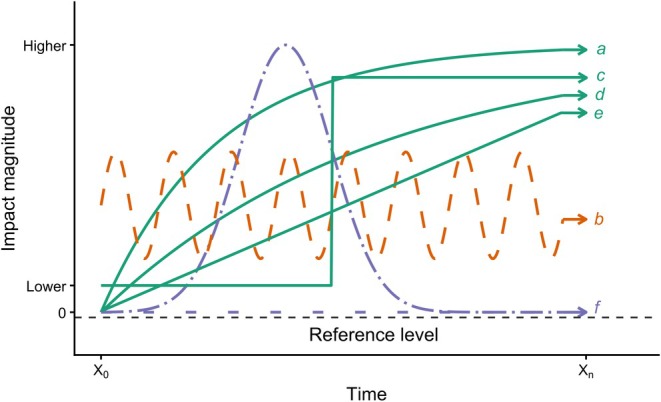
Temporal dynamics of the magnitude of non‐native species impact over time following different potential trajectories. Lines of different colours and shapes represent distinct classes of temporal dynamics. The green lines (*a*, *c*, *d*, *e*) depict unidirectional increases in impact over time; the orange dashed line (*b*) illustrates cyclical fluctuations in impact, characterised by repeated increases and decreases; and the blue dot‐dashed line (*f*) captures a sharp initial rise in impact followed by a subsequent decline.

Ecological impacts are often accumulated over a long time and go unnoticed until surpassing a critical threshold, at which point the consequences become apparent and potentially irreversible. Initially, non‐native species may have negligible or even positive effects on recipient communities at low abundance. However, over the long term, their presence often leads to profound disruptions, resulting in catastrophic consequences for both the compositional and functional structure of ecosystems (Soto *et al*., 2024c). Notably, the same is true for economic or socio‐cultural impacts, albeit being substantially less studied and more dependent on the respectively impacted economy or society (Turbelin *et al*., 2024). Indeed, because the magnitude of impacts has traditionally been associated with the abundance of the invader (Parker *et al*., 1999; but see non‐linearities in Sofaer, Jarnevich & Pearse, 2018) or the extent of the area it occupies (Katsanevakis, Tempera & Teixeira, 2016), it follows that impact can either increase over time until plateauing (trajectory *a* in Fig. 5) while often fluctuating over time in response to changes in the invader's population dynamics (trajectory *b* in Fig. 5; Le Hen *et al*., 2023; Soto *et al*., 2024c). ‘Boom–bust’ dynamics, a recurring cycle of the rise of a population to outbreak levels, followed by a sharp decline (trajectory *f* in Fig. 5; Strayer *et al*., 2017), challenge the assumption that non‐native species and their impacts will persist, suggesting that some may naturally decline over time (Haubrock *et al*., 2022a; Santamaría *et al*., 2022; Soto *et al*., 2023a). Some impacts may show a steady increase over time as the non‐native population gradually adapts to the new conditions (trajectories *a*, *d*, and *e* in Fig. 5), expanding their range, and integrating into the ecosystem. Alternatively, impacts may exhibit a time lag before suddenly increasing in magnitude (trajectory *c* in Fig. 5; Crooks, 2005).

Although non‐native species often are released from their natural enemies (e.g. predators, competitors, parasites, and diseases; ‘enemy release hypothesis’) in the invaded range and can consequently reach high densities (Torchin *et al*., 2003; Colautti *et al*., 2004; Roy *et al*., 2011), the manifestation of their impacts may be delayed due to initially low population densities, often resulting from strong biotic resistance (Haubrock *et al*., 2022a), inadequate colonisation pressure that hinders successful establishment, or suboptimal environmental conditions. Subsequently, when environmental conditions shift or density‐dependent factors facilitate exponential population growth, impacts can intensify rapidly following an initial time lag (Crooks, Soulé & Sandlund, 1999; Spear *et al*., 2021). For instance, an analysis of 197 non‐native plants found lag‐phase durations ranging from 3 to 140 years before a species became markedly invasive (Larkin, 2012). Similar to being subject to an initial change, populations of non‐native species exhibit dynamic impact patterns over time as they are influenced by periodic environmental changes such as natural hazards (e.g. droughts, fires, etc.; Doubledee, Muller & Nisbet, 2003), fluctuations in resource availability (Yang *et al*., 2017), or simply seasonality (trajectory *b* in Fig. 5; Everts *et al*., 2024). Finally, not all non‐native populations will cause persistent or severe negative impacts, as they may be constrained by predation, competition, or unfavourable habitat conditions, preventing large‐scale ecological consequences. Thus, the impact may temporarily diminish during unfavourable conditions but may (or may not) resurge when conditions ameliorate (Spear *et al*., 2021).

One of the first large‐scale and long‐term assessments of biological invasions using true time series was by Haubrock *et al*. (2022a), who analysed abundance and environmental data (e.g. runoff, temperature, precipitation) for the non‐native New Zealand mud snail across 306 European sites from 1979 to 2020 to assess its large‐scale ecological impact. The number of non‐native populations was found to increase steadily over time, with impacts peaking approximately two decades after the first detection, leading to significant ecological consequences influenced by local abiotic conditions. Similarly, Soto *et al*. (2023a) analysed 96 European time series from 1994 to 2019 with meta‐regression modelling to assess trends in the relative abundance of killer shrimp and identify invasion drivers, estimate invasion speed, and examine its impact on recipient community metrics. The results revealed that killer shrimp has become dominant in European waters, with a multidecadal lag phase of ~28 years before spatial expansion (resembling trajectory *e* in Fig. 5), while its increasing abundance was linked to declines in taxa richness, community turnover, and Shannon diversity index. Despite the importance of long‐term studies for the detection of non‐native species (Haubrock *et al*., 2023b) and study of invasion effects over time (Haubrock & Soto, 2023), assessments of impacts over multiple years or even decades are rare (Pergl *et al*., 2019). Consequently, inferences of cumulative impacts (i.e. the joint effects of all present non‐native species collectively) are often based on the (questionable) assumption that ecological, economic, or socio‐cultural impacts will increase gradually with the number of cumulatively reported non‐native species (Seebens *et al*., 2017).

Society's subjective perceptions of impacts, along with how they are assessed, valued, and managed over time, ultimately shape what data are collected, where they are gathered, and the temporal scale of monitoring efforts. While long‐term biodiversity monitoring data have facilitated notable insights into the spatial and temporal dynamics of biological invasions and, thus, ecological impacts (Haubrock *et al*., 2023c; Soto *et al*., 2023a), economic and socio‐cultural impacts are assessed using fundamentally different measures. It is therefore difficult to assess temporal trajectories of these types of impacts associated with biological invasions. Yet, recent studies of the monetary burdens presented by biological invasions concluded generally increasing monetary costs (Cuthbert *et al*., 2021), potentially affected by a mixture of factors as reported costs followed introduction rates and reflected research and awareness (Haubrock *et al*., 2022c).

## IV. CHALLENGES AND FALLACIES IN STUDYING IMPACTS

### (1) The context specificities of invasion impacts

Biological invasions are context‐dependent population‐level phenomena (Haubrock *et al*., 2024b; Sousa, Nogueira & Padilha, 2024), where the interplay between a non‐native species' traits and the characteristics of the recipient ecosystem can influence its success and impacts (Vilà *et al*., 2024). Understanding these dimensions is needed to inform impact assessment and strategic management (Novoa *et al*., 2020). In particular, impacts exerted by non‐native species typically co‐occur with other environmental changes, such as habitat modification, overexploitation, climate change, and pollution, creating myriad multiple stressor interactions and potential emergent effects (Ricciardi *et al*., 2021; Haines *et al*., 2024). This co‐occurrence can create challenges when inferring the prevailing driver of ecological, economic and socio‐cultural impacts, as combinations of drivers can interact antagonistically or synergistically, with effects difficult to predict based on the sum of single stressors (Gissi *et al*., 2021). Specifically, aggression and resource competition by invasive mosquitofish (*Gambusia holbrooki*) increase with temperature (Carmona‐Catot, Magellan & García‐Berthou, 2013) and climate change is predicted to favour many similar non‐native fishes at the expense of native species (Radinger & García‐Berthou, 2020). Cane toads and the signal crayfish (*Pacifastacus leniusculus*) have similarly shown rapid evolutionary changes through space and time (alongside changes in affected natives) that influence their impacts and complicate management strategies (Shine, 2012; Alves *et al*., 2025). Whether non‐native species are drivers rather than ‘passengers’ of ecological change has thus been a topic of interest in the field (Didham *et al*., 2005; MacDougall & Turkington, 2005), with individual study systems or species having multidirectional outcomes (Vilà *et al*., 2024). For instance, wels catfish show negative predation impacts on native fish communities only in habitats disturbed by human activities (Cucherousset *et al*., 2018; Lenhardt *et al*., 2021), which are the rule in large European rivers (Moncada *et al*., 2025). Sport fishes, such as peacock basses of the *Cichla* genus and the common carp, often are mainly problematic in artificially modified systems like reservoirs or impoundments (Benito *et al*., 2015; Franco *et al*., 2022), with habitat simplification exacerbating ecological vulnerabilities to fish invasions (Alexander *et al*., 2015). Under specific circumstances, impacts of non‐native red swamp crayfish are only marginal on macrophyte communities unless occurring in the presence of substantial nutrient pollution (Dercksen *et al*., 2025; but see Arribas, Díaz‐Paniagua & Gomez‐Mestre, 2014). Similarly, terrestrial invasions, such as the spread of cheatgrass in the western USA, illustrate how anthropogenic disturbances, particularly frequent fires and overgrazing, facilitate spread and exacerbate ecological impacts through positive feedback mechanisms (D'Antonio & Vitousek, 1992). We note, however, that alternative perspectives, such as the ‘novel ecosystems’ debate and critiques of invasion alarmism (e.g. Larson, 2007; Davis *et al*., 2011), also form part of the discourse around invasion impacts, although a full treatment of these debates is beyond the scope of this review (but see Section (5)).

Ecological impacts can be highly variable, as a species that exerts strong negative effects in one system may generate positive effects in another (Vilà *et al*., 2024). Nevertheless, in a global meta‐analysis, interactions between invasions and environmental changes were no worse than impacts of invasions alone (Lopez *et al*., 2022), suggesting that the management of invasions should be a primary objective to improve environmental outcomes in the face of multiple global changes (Keck *et al*., 2025b). This perspective is reinforced by conservation interventions aimed at the control of biological invasions being highly effective when compared to other types of interventions (Langhammer *et al*., 2024). However, impacts from individual populations of single non‐native species can be triggered by rapid environmental changes (Ricciardi *et al*., 2021; Spear *et al*., 2021; Haubrock *et al*., 2022a), thus the same species may shift from beneficial to harmful (and *vice versa*) over time, depending on interactions with climate, land‐use or socio‐political changes (de Carvalho‐Souza *et al*., 2024). A species classified as low risk in one area may become a significant invader in another, especially if its spread continues unchecked into more vulnerable ecosystems (Soto *et al*., 2024a). This in turn complicates management in the context of ‘invasion debts’ (Essl *et al*., 2011a), as future impactful non‐native species could already be present, but remain undetected or unmanaged owing to an absence of impact under current conditions (Spear *et al*., 2021). These changes to impacts at the population‐level can be influenced by several factors, such as the arrival of novel genetic material, adaptations and plasticities, as well as creation of more favourable environmental conditions through processes such as habitat disturbance, ecosystem engineering, niche construction, climate change or the prior invasion of a facilitating species (*cf*. ‘invasional meltdown hypothesis’). The invasion of the green crab (*Carcinus maenas*) exemplifies this, with invasion impacts differing substantially between invaded regions in Canada and the USA where it had substantial negative effects on shellfish *versus* in South Africa where ecological impacts have rarely been observed (Howard *et al*., 2018).

In particular, it is probable that future global warming will exacerbate the impacts of thermophilic biological invasions (particularly for poikilothermic animals), should conditions approach their thermal optima and possibly provide longer temporal windows of activity. For example, effects of a non‐native mysid *Hemimysis anomala* have shown strong impact variations along thermal gradients, suggesting that temperature is a key mediator of impact in inland waters (Iacarella *et al*., 2015a). This species has shown strong spatial variations in individual performance towards the ‘invasion front’ (Iacarella, Dick & Ricciardi, 2015b), suggesting that spatiotemporal structuring of populations according to their traits further mediates impact propensities. In the eastern Mediterranean, rising sea surface temperatures have accelerated the decline and local extinctions of cold‐affinity species and the concurrent ‘tropicalisation’ of the marine community, which is increasingly dominated by warm‐affinity non‐native species originating from the Red Sea [Suezian (‘Lessepsian’) migrants (Givan *et al*., 2018; Albano *et al*., 2021)]. This region has become an extinction hotspot, with climate change being the primary driver of local marine extinctions (Nikolaou & Katsanevakis, 2023). This process is further exacerbated by extreme summer temperatures, which have been shown experimentally to drive key native species loss (Yeruham *et al*., 2015; Rilov, 2016). Additionally, competition with non‐native Suezian species has further reduced the resilience of native populations (Yeruham *et al*., 2020). With continued climate warming and intensifying marine heatwaves in the Mediterranean (Garrabou *et al*., 2022), native species are increasingly being pushed beyond their thermal limits, leading to irreversible biodiversity shifts (Albano *et al*., 2021). Consequently, even the most effective conservation measures are unlikely to halt the ongoing dominance of thermophilic non‐native species, rendering further tropicalisation of the Mediterranean an inevitable trend over coming decades (Schickele *et al*., 2021). In such profoundly altered ecosystems, where entire food webs and ecological interactions have been reshaped (Corrales *et al*., 2017), assessing the specific impacts of individual non‐native species on native biodiversity becomes increasingly complex, as their effects are embedded within a broader framework of systemic change.

The connectivity or complexity of the landscape is also important in understanding the extent to which invasion impacts will propagate, with higher connectivity between complex ecosystems facilitating spread of invaders and their effects (Radinger & García‐Berthou, 2020; Dolan *et al*., 2025). Furthermore, impacts often cross ecosystem boundaries in unexpected ways that might only be apparent later. For example, introductions of fishes that consume aquatic invertebrate larvae can reduce the insect emergence into terrestrial environments that subsequently limits prey availability for riparian taxa (Baxter, Fausch & Saunders, 2005). Introductions of freshwater fishes for sport angling, which can alter the trophic web and fish population structure, can disrupt the interconnectedness of freshwater and terrestrial compartments, emphasising the often‐overlooked cross‐system consequences of invasions (Britton *et al*., 2024). Trophic niche shifts may also be functional in contrast to structural changes as non‐native species may force native taxa into constricted trophic niches or increase trophic niche overlap, thus reducing ecological resilience without necessarily changing abundance or richness (Balzani *et al*., 2016; Dominguez Almela, South & Britton, 2021).

The potential vulnerability of insular freshwater habitats to non‐native species impacts was further evidenced in a meta‐analysis (Faria *et al*., 2025), with resource‐use efficiency by insular invaders particularly exacerbated compared to their trophically analogous native comparators. For plants, enemy release following invasion can lead to the evolution of increased competitive ability and heightened resource use (Callaway & Ridenour, 2004), whereas ecological novelty linked to defence and selective foraging could alter biotic resistance levels from native herbivores (Verhoeven *et al*., 2009). Importantly, changes through adaptive (e.g. selection) and non‐adaptive (e.g. plasticity, drift, species sorting, etc.) processes could rapidly alter impact across invasion stages and ecological interactions within trophic networks (Zenni *et al*., 2014). Even pre‐introduction processes affect invasions (Sinclair *et al*., 2020), with urban environments and transport conditions potentially selecting for more robust ‘bridgehead’ populations that exacerbate their invasiveness and potential impact (Briski *et al*., 2018, 2025). Overall, the success and impacts of biological invasions are mediated by a plethora of biotic and abiotic context dependencies, meaning that effects among populations are highly variable depending on the traits and characteristics of the recipient environment or economy. Cryptic invasions represent an additional layer of complexity. The brown seaweed *Rugulopteryx okamurae* was initially misidentified (overlooked due to morphological similarities with a native species) in the Strait of Gibraltar and its invasive potential went unnoticed until favourable conditions (e.g. high temperatures) facilitated an explosive bloom (García‐Gómez *et al*., 2020). The case of this seaweed also illustrates the difficulty of predicting the invasion and strong impacts of some non‐native species (Williams & Smith, 2007), given the very limited invasion history of this species (García‐Gómez *et al*., 2020). This problem of detection is not limited to species misidentification (Brys *et al*., 2025), but often species go under‐monitored due to spatially fragmented and methodologically inconsistent methods (Dominguez Almela *et al*., 2023b), with this situation especially true for less‐charismatic taxonomic groups (Mammola *et al*., 2023).

Eco‐evolutionary contexts between non‐native species and recipient communities must also be considered to understand fully impact magnitude and its variations (Penk *et al*., 2017). In particular, naïveté towards non‐native species, especially concerning non‐native predators that are often linked to severe impacts, can render native populations highly susceptible to impacts. For instance, the introduction of the non‐native brown tree snake to the island of Guam led to the extinction of numerous species of birds, mammals, and reptiles, given that they were maladapted in the absence of analogous native predators (Fritts & Rodda, 1998). Impacts have often been more severe on islands owing to higher levels of ecological novelty (Blackburn *et al*., 2004; Haines *et al*., 2024); however, similar impact variations according to insularity can be seen across habitat types. In a global meta‐analysis of prey naïveté, Anton *et al*. (2020) found that effects were strongest in aquatic habitats, with limited overall evidence for the phenomenon in terrestrial ecosystems. They further found that time since introduction influenced naïveté, with around 200 generations required for anti‐predator responses to develop sufficiently (Anton *et al*., 2020). However, there are examples of faster development of effective biotic resistance mechanisms, such as the case of the non‐native green seaweed *Caulerpa cylindracea* in the Mediterranean. Previously considered as the most impactful non‐native species in the basin (Katsanevakis *et al*., 2016), it soon collapsed in most areas as Mediterranean herbivores adapted and began consuming the invader (Santamaría *et al*., 2022).

### (2) Interpreting and comparing ecological impacts

Non‐native species impacts are shaped by environmental filtering, biotic resistance, ecosystem resilience, and human disturbance, often varying across regions despite similar abundances. The analysis of 160 time series of non‐native crayfish in Europe sampled between 1983 and 2019 by Soto *et al*. (2023b) demonstrated the problem with extrapolating species‐level impacts at small spatial scales to wider impacts at broader scales (e.g. geographical region), and may also be the case when comparing different habitats, such as islands and mainlands, where islands might be more isolated with smaller habitat areas and more endemic species than mainland areas, and thus be more susceptible to invasion impacts (Reaser *et al*., 2007). Recognising these differences at which impact mechanisms manifest is crucial in defining and measuring impacts, particularly when comparing and considering management implications. Not all impacts are equally apparent and those at the population and community levels may be easy to detect and measure (e.g. shifts in population abundances and species diversity). By contrast, lower‐level impacts are likely to be more widespread but harder to detect and measure, such as lower physiological condition (Bódis, Tóth & Sousa, 2014; Ferreira‐Rodríguez, Sousa & Pardo, 2018) or short‐term dietary shifts caused by interspecific interactions between the invader and native populations (Amaral *et al*., 2021). This ambiguity of defining impacts and impact thresholds results in indirect or cascading impacts of biological invasions being difficult to quantify and predict, despite having the potential to be even more damaging (and costly) than direct impacts (Walsh, Carpenter & Vander Zanden, 2016). The invasion of yellow crazy ants (*Anoplolepis gracilipes*) on Bird Island (Seychelles) exemplifies the cascading effects of non‐native species, promoting coccid infestations in *Pisonia grandis*, leading to tree mortality, defoliation, and reduced invertebrate diversity, key prey for insectivorous birds (Hill *et al*., 2003). Similarly, the introduction of Burmese pythons (*Python molurus bivittatus*) in the Florida Everglades caused major declines in small‐ to mid‐sized mammals (Dorcas *et al*., 2012), triggering food web restructuring and degrading ecosystem function (Guzy *et al*., 2023). Rodents, more resistant to predation, became dominant, shifting host use by *Culex cedecei* mosquitoes towards hispid cotton rats (*Sigmodon hispidus*) and increasing Everglades virus transmission to humans (Burkett‐Cadena *et al*., 2021).

Beyond ecological complexity and other modulating factors, impact severity is also shaped by cultural, economic, and social perceptions, which influence whether a non‐native species is perceived as harmful, neutral, or even beneficial (Bacher *et al*., 2018; Kapitza *et al*., 2019). In Western cultures, impacts are often framed through economic and biodiversity loss metrics, with greater emphasis on provisioning and regulating ecosystem services, whereas in other parts of the world, cultural ecosystem services may be valued more highly and the disruption of spiritual or cultural relationships with native species and landscapes may constitute the most significant invasion impact (Reo & Ogden, 2018). Moreover, socio‐economic disparities shape how the effects of biological invasions are perceived and managed as wealthier nations often have greater resources for biosecurity and mitigation (Reaser *et al*., 2007; Bacher *et al*., 2018). They may tolerate ecological impacts if economic gains from, for example, commercial fisheries (Acevedo‐Limón *et al*., 2020) or forestry (Dickie *et al*., 2014) are substantial and/or ecosystem function is maintained despite the biodiversity loss (Gozlan, 2008). Poorer regions, however, often bear disproportionate costs from non‐native species introductions due to their reliance on subsistence agriculture and local ecosystems, underscoring that those suffering the most from invasions are rarely the ones who eventually benefit from and are equipped to deal with them (Reaser *et al*., 2007). For example, the recent invasion of pelagic sargassum (*Sargassum fluitans* and *S*. *natans*) in Ghana has caused ecological and livelihood disruptions. However, communities frequently misattribute the cause of these events to offshore oil and gas activities, with this misperception resulting from the limited access of communities to trusted scientific information coupled with pre‐existing tensions with extractive industries (Atiglo *et al*., 2024). This is in contrast to communities in the Caribbean and Central America, where early warning systems and re‐use strategies have emerged from biological invasion (Dominguez Almela *et al*., 2023a).

Ecological impacts are also not equal in significance across different levels of biological organisation. For instance, while a reduction in individual fitness may seem to be relatively straightforward to evaluate, particularly if it affects reproductively important individuals, the consequences of a minor change in community structure could have far‐reaching consequences by altering future ecosystem resistance and resilience, making comparisons between the two levels of impact much more complex. Invasions are often controlled at the population level, yet management is typically directed at the ecosystem level where invasion impacts may be better understood and thus addressed (Gutiérrez *et al*., 2014). Correctly scaling the multitude of different direct and indirect impacts may also be further compounded by temporal factors acting on the strength and direction of ecological impacts (Soto *et al*., 2023a). These issues are then further compounded by differences in sampling methodologies and metrics, but also detection probabilities (e.g. in aquatic environments; Katsanevakis & Moustakas, 2018), and a lack of standardisation that make consistent impact assessments difficult (Barney *et al*., 2013), as well as various generalised ethical and conceptual considerations that can make properly defining and measuring impacts extremely difficult (Haubrock *et al*., 2025a). In many cases, the impacts of non‐native species are entirely unknown, as exemplified by a recent comprehensive breakdown of impacts within Germany identifying ‘unknown impacts' for 97.9% of 1,962 established species (Haubrock *et al*., 2025b).

### (3) Challenges in quantifying social and cultural impacts

Assessing the socio‐cultural impacts of biological invasions is often challenging due to the difficulty of quantifying non‐material values, such as cultural identity, traditional ecological knowledge, and aesthetic appreciation of landscapes, often leading to biases and misconceptions (Table 2). Information on non‐material values from indigenous communities and tribes whose lives are intrinsically intertwined with their surrounding natural resources can be difficult to access, due to linguistic barriers and lack of accessibility. Unlike economic losses or biodiversity declines, which rely on measurable indicators (respectively, money and loss of biodiversity as measured through different proxies), socio‐cultural impacts often lack clear metrics and standardised methodologies, making them harder to integrate into management decisions. For instance, non‐native tree species like *Acacia* spp. have altered landscapes in regions such as South Africa, where they threaten native fynbos ecosystems and alter microbial communities (Le Roux *et al*., 2011, 2018), yet they are also culturally valued for their use in firewood and charcoal production, creating conflicts between conservation priorities and local livelihoods (Shackleton *et al*., 2014). Invasive cacti such as *Opuntia* spp. and *Cylindropuntia* spp. are some of the most damaging plant invasions worldwide, yet at the same time are valued as a nutritious and healthy fruit crop, for cochineal production, and are promoted as an option to minimise the impacts of global climate change and land degradation on food security in developing countries (Novoa *et al*., 2016). Islands often endure significant environmental degradation, species extinction, and high levels of social poverty, stemming from colonial settlement and extensive exploitation of natural resources, which have led to the development of fragile economies (Cronk, 1989). A notable example is New Zealand flax (*Phormium tenax*), introduced to St. Helena, which initially brought prosperity due to high demand for flax rope during the World Wars. However, by the 1960s, the industry's decline was triggered by the rise of cotton, synthetic fibres, and increased shipping costs. Today, New Zealand flax has overrun large areas of the island's endemic cloud forest, dominating the vegetation and elevating the risk of soil erosion on steep cliffs. This has necessitated substantial conservation efforts, involving local communities, focused on its removal and the repatriation of endemic species (Maunder *et al*., 1995).

**Table 2:** Common biases and misconceptions in assessing invasion impacts.

Bias/misconception	Description	Implication
Publication bias	Studies with strong or negative impacts are more likely to be published	Overestimation of impact severity and frequency
Geographic bias	Research is concentrated in Europe/North America	Under‐representation of impacts in e.g. the Global South
Taxonomic bias	Focus on well‐known groups (e.g. vertebrates)	Neglect of under‐studied taxa and many impacts
Temporal bias	Short‐term studies dominate	Failure to detect lag phases or long‐term cumulative effects
Negativity bias	Impacts (and species) assumed detrimental by default	Positive or neutral effects are overlooked, skewing interpretation
Mechanism *versus* consequence bias	Pathways of impacts are conflated with system‐level outcomes	Reduced clarity on causality and scaling of impacts
Single‐driver assumption	Invasions are assessed in isolation from other stressors	Over‐simplification of interacting mechanisms

Conservation frameworks emphasising scientific data over local knowledge will thus likely overlook important cultural dimensions, often creating conflicts. For example, variations in how impacts can be perceived by different cultural groups often result in conflicts over management applications, particularly when eradication efforts target species that have become socially or economically integrated (Shackleton *et al*., 2019a). A striking example is the European rabbit in Australia, where its devastating impact on native vegetation and agricultural systems led to large‐scale control measures, including the introduction of viral biocontrol agents (Strive & Cox, 2019). However, the same species is also non‐native to part of Europe, but remains deeply embedded in European cultural traditions, with a long history of hunting and farming (Lloveras *et al*., 2016; Delibes‐Mateos *et al*., 2018). Similarly, debates over the culling of feral cats and horses (*Equus ferus caballus*) in North America, Eastern grey squirrels in the UK, or monk parakeets (*Myiopsitta monachus*) in Spain highlight how biological invasions intersect with ethical and psychological concerns, as some groups advocate for their removal due to ecological damage, while others view them as symbols of heritage and freedom (Dunn *et al*., 2018; Deak *et al*., 2019). In Italy, attempts to control the non‐native Eastern grey squirrel led to animalist associations stopping the activity and even bringing scientists and managers to court, due to the perception of these activities as unethical and familiarity with this introduced mammal species (Bertolino & Genovesi, 2003).

### (4) Assumptions and fallacies in impact studies

Compounding the challenges around context specifics of impacts is the difficulty of standardising impact assessments. Methodologies vary widely across taxa, ecosystems, and management frameworks, leading to inconsistencies in impact quantification. This inconsistency is further exacerbated by the advent of novel monitoring approaches (Fricke & Olden, 2023), such as environmental DNA (Everts *et al*., 2024), ecoacoustics (Chhaya *et al*., 2021), or the use of drones (Ribeiro‐Silva *et al*., 2018), for which no baseline or pre‐invasion data exist. Extrapolating data from one region or ecosystem to another is often problematic due to an array of context dependencies associated with environmental conditions and the species involved in the interactions. Impact assessments are further complicated by the reliance on data inferred from experimental laboratory studies. While these experiments can provide indications of potential effects (Alexander *et al*., 2014), they often lack the ecological complexity of real‐world environments, including natural species interactions (Britton, 2018). Additionally, many invasion impact assessments focus on short‐term or highly visible consequences, while more subtle, long‐term effects, such as genetic homogenisation, trophic disruptions, or gradual ecosystem shifts, remain underrepresented in the literature.

The assumption that a non‐native species' impact will be consistent across different regions is a common fallacy, as invasion outcomes are highly context dependent (Haubrock *et al*., 2024b; Sousa *et al*., 2024). A species that causes ‘significant’ ecosystem disruptions in one area may be able to integrate more seamlessly in another if it has adequate traits that benefit establishment (Mahoney *et al*., 2015), despite differences in habitat structure, biotic interactions, or local disturbances (Vilà *et al*., 2024). For example, the recorded impacts of many marine non‐native species occur predominantly within their thermal niche of origin and the severity of impacts displays a hump‐shaped relationship with temperature (Bennett *et al*., 2021). Highlighting how the severity of non‐native species impacts is shaped by factors like ecosystem resilience, i.e. the ability of an ecosystem to absorb disturbances and maintain functionality (Chaffin *et al*., 2016), systems that have already been degraded by human activities are often prone to experience exacerbation of existing problems, whereas in more intact ecosystems, native species and processes may buffer against their effects (Hou, Bai & Si, 2023).

The ‘invasional meltdown’ hypothesis describes how multiple non‐native species can facilitate each other's spread and intensify their respective ecological impacts, further complicating impact assessments (Simberloff & Von Holle, 1999). In many freshwater systems, non‐native bivalves such as the zebra mussel provide a hard substrate that facilitates the establishment of other non‐native species, often from its native region, the Ponto Caspian (Ricciardi, 2001; Soto *et al*., 2023c). These include non‐native macrophytes and predatory fish and this facilitative process creates cascading effects that amplify ecological disruptions (Britton *et al*., 2010). Another example is the pumpkinseed sunfish (*Lepomis gibbosus*), which preys on odonate larvae, thereby reducing predation pressure on non‐native American bullfrog tadpoles in Europe and indirectly aiding its establishment (Adams, Pearl & Bruce Bury, 2003). Importantly, not all invasion impacts are purely additive; some interactions may lead to non‐linear effects, where the introduction of one species mitigates or even counterbalances the effects of another. For example, the non‐native red swamp crayfish limits the predatory effects of American bullfrogs on native amphibians by serving as an alternative prey, yet this predation pressure triggers increased reproductive output in red swamp crayfish, potentially leading to higher population densities and greater overall ecosystem impact (Bissattini, Buono & Vignoli, 2018).

### (5) Biases in invasion impact research

Research on the impacts of non‐native species is influenced by various biases. The field has long debated the conceptualisation, methodology, and ethical implications of biological invasions, with increasing awareness of taxonomic, geographic, methodological, and even emotional biases. Such biases can lead to inaccurate assessments of the effects of non‐native species, ultimately affecting conservation policies and management strategies.

One of the key conceptual biases in invasion science (see Table 2) arises from the definition of ‘invasive’ itself (Appendix S2). It is crucial to note that even the inclusion of ‘impact’ in the definition of invasive species remains subject to considerable debate within the field. While some invasion scientists argue against including impact in the definition of invasiveness, preferring the link to a non‐native species' ability to spread, others argue that impact is the criterion that ultimately matters (Soto *et al*., 2024a). Policymakers and managers, however, rely on empirical data on impacts to justify biosafety measures and management programmes, as interventions may be difficult to implement without demonstrable harm (Davis *et al*., 2011; Davidson & Hewitt, 2014). Public awareness and political support for non‐native species management also tends to be stronger when tangible negative effects, such as biodiversity loss or economic damage, are documented (Crystal‐Ornelas & Lockwood, 2020). Additionally, impact‐driven research is more likely to secure funding, influencing the focus of invasion science studies (Wilson *et al*., 2007; Pyšek *et al*., 2008). However, some authors caution that an exclusive focus on demonstrated impacts may underestimate long‐term or subtle ecological effects, particularly in marine systems where impacts are more difficult to assess (Ojaveer *et al*., 2015) and could bias research towards species already known to cause harm while overlooking potentially problematic ones (Crystal‐Ornelas & Lockwood, 2020; Watkins *et al*., 2021). Social and ethical biases also shape the field of invasion science. The terminology used, such as ‘invasion’, ‘war’, and ‘enemy’, evokes emotional responses that can influence public perception and management policies (Larson, 2011; Ahmed *et al*., 2025). Furthermore, decisions regarding non‐native species management are often based on underlying value judgments, whether instrumental (focused on human utility) or intrinsic (emphasising the inherent worth of species and biodiversity) (Cassini, 2020). The way researchers frame non‐native species and their impacts thus has significant ethical and policy implications. Currently, some argue that non‐native species are framed negatively in published literature, often associated with harmful impacts (Pereyra *et al*., 2024, 2025; Simberloff *et al*., 2024), with the recent surge in studies about economic costs of non‐native species highlighting the bias towards emphasising detrimental effects, often neglecting cases where such species provide ecosystem services or economic benefits (Schlaepfer *et al*., 2011; Sax *et al*., 2022; Boltovskoy *et al*., 2022).

Methodological biases also pervade impact studies, particularly in distinguishing correlation from causation (Gurevitch & Padilla, 2004; Hulme *et al*., 2013). Many studies document correlations between non‐native species presence and ecological changes without establishing direct mechanisms and causal links (Cassini, 2020). Moreover, studies reporting negative impacts are more likely to be published and cited, leading to a publication bias that overemphasises detrimental effects while underreporting neutral or positive outcomes (Davidson & Hewitt, 2014; Katsanevakis *et al*., 2014; Tsirintanis *et al*., 2022). Statistical limitations further compound these biases, as ecological studies often suffer from low statistical power due to small sample sizes and short time series, increasing the risk of Type II errors (Rosnow & Rosenthal, 1992; Davidson & Hewitt, 2014). Taxonomic, geographic, and accessibility biases significantly skew impact assessments in invasion science. Research disproportionately focuses on specific taxonomic groups, such as primary producers in marine invasion studies, even though they represent only a fraction of introduced species (Watkins *et al*., 2021). Similarly, invasion studies are heavily concentrated in Europe and North America, while megadiverse regions such as Africa, Asia, and other parts of the Southern Hemisphere remain underrepresented (Hulme *et al*., 2013; Bellard & Jeschke, 2016; Crystal‐Ornelas & Lockwood, 2020; Munro *et al*., 2024; Prestes *et al*., 2024). This imbalance limits the understanding of invasion mechanisms in unique ecosystems, hinders the development of globally applicable ecological principles, underestimates the impacts of biological invasions in less‐studied areas, weakens management and conservation strategies, and results in resource allocation based on incomplete data (Pyšek *et al*., 2008; Florencio *et al*., 2019; Watkins *et al*., 2021). Moreover, accessibility biases lead studies to be conducted in easily reachable locations, such as areas near roads or research institutions, which may distort perceptions of invasion severity (Munro *et al*., 2024). Marine invasion research also exhibits biases in species selection and geographic focus, often overrepresenting invasions in English‐speaking regions and favouring well‐known non‐native species, while potentially overlooking other impactful non‐native species (Watkins *et al*., 2021). Similarly, plant invasion research tends to focus on species with demonstrated impacts, neglecting earlier invasion stages, such as naturalisation, which are crucial for understanding long‐term ecological consequences (Pyšek *et al*., 2008).

Finally, broader critiques of invasion science question the extent to which non‐native species' impacts are exaggerated. Some scholars argue that invasion science has historically placed disproportionate emphasis on negative effects, often neglecting cases where non‐native species contribute positively to ecosystems (Davis *et al*., 2011). A systematic review of competition studies in invasion science suggests the presence of context bias, where the framing of studies, particularly the use of ‘boilerplate’ (i.e. formulaic or standardised) introductions emphasising the negative impacts of non‐native species, might influence the interpretation of results, especially in observational studies (Warren *et al*., 2017). While the use of such biased language has shown a declining trend over time, it still warrants consideration in the evaluation of invasion science literature. This debate has even led to accusations of ‘invasion denialism’ (Ricciardi & Ryan, 2018). Invasion denialism, however, goes beyond academic critique and encompasses the systematic rejection of empirical evidence, often employing questionable (rhetorical) tactics similar to those found in other forms of science denialism (Simberloff & Meyerson, 2024). These tactics include cherry‐picking data, misrepresenting scientific findings, and discrediting experts through accusations of bias or conspiracy (Ricciardi & Ryan, 2018). Some critics dismiss invasion science as ‘pseudoscience’ or a form of ‘green xenophobia’, despite strong empirical evidence demonstrating the ecological and economic harms of biological invasions. While it is important to acknowledge biases in invasion research and foster a more nuanced discussion, these efforts should not be conflated with denialism, which seeks to manufacture doubt about well‐established scientific consensus. Recognising and addressing both research biases and denialist rhetoric in invasion science is difficult, but remains crucial for improving the accuracy and relevance of impact studies. A more nuanced, evidence‐based approach that accounts for conceptual, methodological, social, taxonomic, and geographic biases will lead to more effective conservation and management strategies (Jarić *et al*., 2020; Vimercati *et al*., 2020). By refining research methodologies and adopting a more critical perspective, the field can move beyond overly simplistic narratives and towards a more objective assessment of non‐native species in ecosystems.

## V. NON‐NATIVE SPECIES RISK ANALYSIS AND IMPACT ASSESSMENT

In invasion science, non‐native species risk analysis is a proactive framework that consists of three sequential components: risk identification (or screening), risk assessment, and risk management (e.g. Vilizzi *et al*., 2022a,b). Risk analysis aims to detect potentially invasive species, assess their likelihood of introduction, establishment and spread, assess the magnitude of actual or potential ecological, socio‐economic and health impacts, and identify options to prevent or mitigate such impacts. Risk analysis therefore acts as the umbrella under which impact assessments are implemented following a full evaluation of the potential risks posed by (invasive) non‐native species, which are generally prioritised in terms of their risk ranking (e.g. low, medium, and high risk) as part of the screening phase. While risk analysis is a proactive process, impact assessment is reactive, focusing on evaluating and quantifying the ecological, economic, and socio‐cultural consequences of an already established non‐native species (Roy *et al*., 2018). While risk analysis helps in anticipating and preventing biological invasions, impact assessment is crucial for quantifying the damage caused by species that have already spread (Andersen *et al*., 2004). The two processes are closely linked, as impact assessment provides empirical data that can improve future risk analysis, refining predictions and enhancing management strategies. This aligns with the dynamic nature of non‐native species risk analysis (Vilizzi *et al*., 2022a), which involves a periodic review‐and‐revision approach concerning both the risk analysis process and the management strategy in the light of impact assessment for the non‐native species of concern (Mumford *et al*., 2010).

### (1) Non‐native species risk analysis

To evaluate the risks posed by non‐native species in terms of their likelihood of introduction, establishment, spread, and impact, several risk identification and assessment schemes have been developed (reviewed in Srėbalienė *et al*., 2019). The currently available schemes and, in some cases, related decision support tools, differ in their focus, scope, and methodological approach, with some designed for (early‐stage) risk identification and others for (follow‐up) risk assessment (and risk management). Overall, these schemes are essential for prioritising management actions and ensuring that conservation resources are allocated efficiently once impact assessments have been fully implemented (Britton *et al*., 2011). Risk screening and assessments have historically been applied at the species level, focusing on general traits and invasion potential (D'hondt *et al*., 2015). However, this approach can overlook critical population‐level differences, leading to inaccurate predictions (Haubrock *et al*., 2024b). This is because many populations remain undetected until they enter an exponential growth phase (i.e. sleeper populations), making them difficult to control before significant ecological and economic damage occurs (Soto *et al*., 2023a). Additionally, societal, economic, and regulatory constraints often limit management capacity, particularly in low‐ and middle‐income regions where funding and ecological data are scarce (Bradshaw *et al*., 2024). These challenges highlight the need for robust, accessible, easily deployable, and adaptable decision support tools that consider both species‐ and population‐level variation in invasion dynamics (Haubrock *et al*., 2024b).

One of the earliest and most widely applied decision support tools for risk identification is the Australian Weed Risk Assessment (WRA), originally developed for non‐native terrestrial plants (‘weeds’) and then adapted to aquatic plants (Pheloung, Williams & Halloy, 1999; Gordon *et al*., 2010). The WRA's derivatives, comprising the first‐generation Invasiveness Screening Kit (ISK) tools, namely the freshwater Fish Invasiveness Screening Kit (FISK) (Copp, Garthwaite & Gozlan, 2005a,b; Lawson *et al*., 2013) and its ‘sister’ toolkits (Copp, 2013), have been employed for the risk identification of some aquatic taxonomic groups (Vilizzi *et al*., 2019). More recent advancements have led to the development of the second‐generation ISK tools, which include the Aquatic Species Invasiveness Screening Kit (AS‐ISK: Copp *et al*., 2016b, 2021) applicable to all aquatic organisms (i.e. freshwater, brackish, and marine animals and plants), the Terrestrial Animal Species Invasiveness Screening Kit (TAS‐ISK: Vilizzi *et al*., 2022b), and the Terrestrial Plant Species Invasiveness Screening Kit (TPS‐ISK: (Vilizzi *et al*., 2024) applicable to terrestrial animals and plants, respectively (Vilizzi *et al*., 2025). These second‐generation WRA‐type decision support tools incorporate climate change scenarios, confidence levels, multilingual support, and structured decision‐support methodologies to improve predictive capacity and comply with the ‘minimum standards’ for screening non‐native species under EU Regulation No. 1143/2014 on the prevention and management of the introduction and spread of ‘invasive alien species’ (EU, 2014; Roy *et al*., 2018).

These assessments, and those that have evolved in the last decade, follow strictly the provisions of the Commission Delegated EU Regulation No. 2018/968 of 30 April 2018 supplementing Regulation No. 1143/2014 of 22 October 2014 with regard to risk assessments in relation to non‐native species (European Commission, 2022b). As turnkey decision support tools, they have been widely used worldwide in a broad range of applications (Vilizzi *et al*., 2021, 2024), exceeding those of the other screening tools available in a semi‐automated workbook format. These tools include: (*i*) Harmonia+, which integrates ecological, economic, and human health risks, and the related Pandora+, which assesses the threats posed by pathogens and parasites associated with biological invasions, a feature not available in the other screening tools (D'hondt *et al*., 2015, 2025); (*ii*) the Canadian Marine Invasive Screening Tool (CMIST; Drolet *et al*., 2016), originally designed for marine invertebrates and more recently adapted to freshwater invertebrates (Brown & Therriault, 2022); (*iii*) the lesser‐known Fish Invasiveness Screening Test (FIST; Singh & Lakra, 2011); and (*iv*) the recently developed Non‐Indigenous Species Screening Tool (NISST) for plants, invertebrates, and fish, which also accounts for climate change predictions and provides assessors with additional flexibility when screening species by the incorporation of Monte Carlo procedure‐generated scores (Wilcox *et al*., 2025). Most of these tools include an evaluation of the ‘potential’ impacts posed by the species under screening. Further, the ISK tools, Harmonia+, and CMIST comply with all the key principles (except for comprehensiveness) of risk assessment tools identified by Srėbalienė *et al*. (2019), namely effectiveness, transparency, consistency, risk management, precautionary, science‐based, and continuous improvement.

For risk assessment, the European Non‐Native Species in Aquaculture Risk Analysis Scheme (ENSARS) offers a structured, modular approach that evaluates a species' introduction pathways, establishment potential, and socio‐economic consequences (Copp *et al*., 2016a; Tarkan *et al*., 2020; Li *et al*., 2025). The ENSARS consists of eight modules, with seven being the (core) assessment, namely entry, pre‐screening (*cf*. ISK tools), pathway, facility, organism, infectious agent, and socio‐economic. The eighth module serves as a risk summary and risk management component, integrating the outcomes of the preceding assessments to inform decision‐making. Beyond a trial evaluation of the 24 non‐native species listed in Annex IV of European Council Regulation No. 708/2007 concerning use of non‐native and locally absent species in aquaculture (Copp *et al*., 2016a), the ENSARS has since been applied in Türkiye, Brazil, and China (Tarkan *et al*., 2020; De Camargo, Cunico & Gomes, 2022; Li *et al*., 2025). For risk management, the Modular Management Tool for non‐native aquatic species and population risks has been developed to prioritise introduced species by risk and then assess the potential impacts of management options and their impacts for the control or eradication of the target populations (Britton *et al*., 2011). This scheme comprises four modules: (*i*) prioritisation of introduced species; (*ii*) species risk to the receiving water body; (*iii*) impact of the management action; and (*iv*) cost of the management action.

Similar to impact assessment, these tools for non‐native species risk analysis are instrumental in shaping regulatory policies, guiding decisions on species trade restrictions, quarantine measures, and early warning systems. However, despite their advantages, these tools face some limitations. Some rely heavily on expert judgement, which may introduce subjectivity and potential biases in species rankings (González‐Moreno *et al*., 2019; Tarkan *et al*., 2024b; Błońska *et al*., 2024). Additionally, most of these schemes do not account fully for population‐level differences, meaning that local adaptations and genetic variability within species are often overlooked (Haubrock *et al*., 2024b). Another significant challenge is the lack of integration of economic and socio‐cultural factors, particularly in tools primarily focused on ecological impacts (Tarkan *et al*., 2024a). Moreover, the quantification of uncertainty remains a major limitation in many frameworks (González‐Moreno *et al*., 2019). This variability often stems from differences in assessor expertise, protocol structure, and scoring criteria, indicating that many tools struggle to produce reliable and replicable outcomes. Without robust methods to quantify and manage these uncertainties, such as clearer scoring guidelines, better training, and collaborative decision‐making, the accuracy and credibility of impact assessments may be compromised.

### (2) Evolution of quantitative impact assessments

One of the earliest formalised approaches to assessing invasion impacts was proposed by Parker *et al*. (1999), who introduced the simple formula (henceforth, the Parker–Lonsdale equation): Impact = Range size × Abundance × *Per‐capita* effect. This equation was appealing due to its intuitive structure, offering a straightforward way to estimate ecological impact based on key invasion parameters. However, its limitations quickly became apparent, as it failed to account for context‐dependent variation, species interactions, and the challenges of defining *per‐capita* effect in a standardised way. The term encapsulates the ecological, economic, or socio‐cultural consequences of an invasion, yet varies depending on species traits, environmental conditions, and the scale at which impacts are observed.

While biomass, abundance, and range are useful proxies for impact assessment, their applicability varies widely across taxa and ecosystems. Biomass, for instance, may be an effective metric for certain organisms, particularly within the same trophic level or functional group (Dickey *et al*., 2020), but it is inadequate for plants, pathogens, or microorganisms for which density, area occupied, or number of affected hosts may be more relevant (Cowan *et al*., 2011). Additionally, for many aquatic species, biomass alone does not necessarily reflect impact severity, as different species with similar biomass can exert vastly different ecological pressures (García‐Berthou *et al*., 2005; Ricciardi *et al*., 2013). Density, in some cases, may offer a better metric, but even this can be misleading when comparing different life stages or functional roles within ecosystems (Jeschke *et al*., 2014). The challenge of using simplistic measures becomes particularly evident in systems with multiple non‐native species interacting, where direct and indirect effects alter impact dynamics, while environmental conditions can mediate impact severity. For instance, certain species may only exert strong effects in degraded or human‐altered ecosystems, making it difficult to generalise impact predictions based on range or abundance or biomass alone. Thus, while the Parker–Lonsdale equation (Parker *et al*., 1999) laid the groundwork for impact quantification, it fails to capture the complexity of biological invasions.

Impact assessment frameworks have since evolved to consider not only species abundance and distribution but also temporal and spatial variations in impact severity, ecological resilience, and broader socio‐economic consequences. Since Parker *et al*. (1999), several methods have been put forward that retain the ‘pillars’ of the Parker–Lonsdale equation. For example, Nentwig, Kühnel & Bacher (2010) proposed ‘potential’ and ‘actual impact scores’, with the former accounting for documented ecological and economic impacts, and the latter accounting for the percentage of area occupied in Europe. Narščius *et al*. (2012) proposed the Biological Invasion Impact/Biopollution Assessment System (BINPAS), an online application for non‐native species impact assessment based on a classification of the abundance and distribution range of non‐native species related to the magnitude of their impacts on communities, habitats, and ecosystem functioning. Based on the Parker–Lonsdale equation, Latombe *et al*. (2022) developed GIRAE (Generalised impact = Range size × Abundance × Per‐unit effect), which extends the Parker–Lonsdale equation by allowing modelling for both single and multi‐species scenarios, as well as non‐linear, context‐dependent relationships between these components to reflect real‐world ecological complexity better.

Another method, aiming to retain the relative simplicity of the Parker–Lonsdale equation while addressing some of its shortcomings, is the Relative Impact Potential (RIP) metric (Dick *et al*., 2017; Laverty *et al*., 2017; Dickey *et al*., 2020). This method, focused solely on ecological impacts, assesses the potential impact of a non‐native species relative to that of a trophically analogous native species or other non‐native species, based on its *per capita* feeding rate and a proxy of numerical response, such as abundance, density, fecundity, or catch per unit effort. This approach is a logical extension of the proposal that the Comparative Functional Response (CFR) method be used as a universal, *per capita* measure of the Parker–Lonsdale equation's ‘effect’ (Dick *et al*., 2014; Iacarella *et al*., 2015a) The CFR method has proved effective and popular for assessing and, crucially, predicting non‐native species impacts over the past decade (Faria *et al*., 2023, 2025). Indeed, the simplicity and flexibility of the CFR method means that it can be applied to any consumer and resource interaction, under myriad biotic and abiotic contexts, and calculated based on laboratory or field observations. While effective at highlighting a damaging non‐native species by itself (resource acquisition rates are at the core of many invasion ecology hypotheses), accounting for some numerical response proxy converts this *per capita* effect into an ecosystem‐level impact, while allowing the benefits of the former to be retained. Ultimately, it allows the potential relative impact of a non‐native species to be quantified in a simple manner, with scores above 1 indicating a non‐native species exerting greater impact than its control reference (e.g. the native species), those equal to 1 predicting similar impacts, and those less than 1 predicting less‐severe impacts. RIP scores have also been shown to correlate positively with actual ecological impacts of non‐native species in the field (Laverty *et al*., 2017).

Furthermore, through its modular nature, the RIP is capable of being fine‐tuned depending on the assessment type required. For example, the Relative Invasion Risk metric combines species impact with their availability within the pet trade (Dickey *et al*., 2022), a common proxy for propagule pressure (e.g. Montgomery *et al*., 2023), while the Resource Reproduction Qualifier accounts for resource abundance dynamics under different abiotic contexts (South *et al*., 2022). While these provide a straightforward method of assessing and predicting the impacts of non‐native species to facilitate effective prioritisation, there remain limitations. Ultimately, the *per capita* nature of the CFR does not account for synergies or antagonisms emerging from multiple consumers interacting. Although efforts have been made to address this through the Relative Total Impact Potential (Dickey *et al*., 2021) – a method for assessing the combined impact on a system as trophically analogous native and non‐native species fluctuate across different invasion stages (Dick *et al*., 2017) – the role of inter‐ and intraspecific interactions in influencing *per‐capita* consumption rates remains understudied (Augustyniak *et al*., 2025).

Tools such as *InvaCost* have introduced monetary valuation into impact assessments, providing estimates of the financial burden that non‐native species have imposed on economies by causing damages or requiring management efforts (Diagne *et al*., 2020). However, economic impact assessments come with their own challenges, as they often fail to capture long‐term, indirect, or cascading effects that extend beyond immediate financial losses. Additionally, recent studies have emphasised the importance of temporal scales, recognising that invasion impacts may change over time as some species exhibit ‘boom–bust’ population dynamics, where initial high impacts diminish as populations stabilise, while others gradually accumulate more severe effects over decades (Haubrock *et al*., 2022a; Soto *et al*., 2023a). A further advancement in quantitative impact assessment is the Cumulative IMPacts of invasive ALien species calculator (CIMPAL), which spatially integrates species distributions, habitat data, and impact scores to identify cumulative invasion hotspots (Katsanevakis *et al*., 2016). Still, it requires robust data and may overestimate impacts due to assumed spatial homogeneity and a uniform impact magnitude across individuals of a given species (Magliozzi *et al*., 2020; Polce *et al*., 2023).

### (3) Types of impact assessments

With biological invasions globally rising, standardised impact assessment protocols (Table 3) have become essential tools for prioritising management actions. These frameworks evaluate the ecological and/or socio‐economic impacts of non‐native species, providing structured approaches for comparing risks across taxa and regions (González‐Moreno *et al*., 2019; Roy *et al*., 2023a). Protocols vary in scope and focus. Some emphasise ecological effects, while others also incorporate socio‐economic dimensions (Blackburn *et al*., 2011; Hawkins *et al*., 2015; Nentwig *et al*., 2016; Bacher *et al*., 2018), offering a more comprehensive view of species invasion impacts. EICAT, adopted by the IUCN, categorises and assesses species by the severity of their negative impacts on native biodiversity (Blackburn *et al*., 2011; Hawkins *et al*., 2015), further expanded into EICAT+ to offer a standardised method of classifying positive impacts on the individual performance, population size, and area of occupancy of a native species (Vimercati *et al*., 2022). Its counterpart SEICAT assesses resulting disruptions to human activities and livelihoods (Bacher *et al*., 2018). Other commonly used protocols are the Generic Impact Scoring System (GISS), which assigns numerical scores to quantify ecological and economic impacts, Harmonia+, and the Invasive Species Environmental Impact Assessment (ISEIA), which integrate invasion potential with policy‐relevant risk criteria (Branquart, 2009; D'hondt *et al*., 2015; Vanderhoeven *et al*., 2015). National schemes such as the Great Britain Non‐Native Risk Assessment (GB‐NNRA) (Baker *et al*., 2008; Mumford *et al*., 2010) and the Norwegian Generic Ecological Impact Assessment of Alien Species (NGEIAAS) (Gederaas *et al*., 2012; Sandvik *et al*., 2013) offer tailored approaches that account for country‐specific conservation priorities. The recently proposed Dispersal‐Origin‐Status‐Impact (DOSI) framework (Soto *et al*., 2024a) adds nuance by assessing invasions at the population level, considering dispersal methods (assisted or independent), origin (native or non‐native), status (expanding, stationary, or shrinking), and impact type, enabling flexible and context‐sensitive evaluations (Tarkan *et al*., 2024b; Błońska *et al*., 2024; Haubrock *et al*., 2025c). These impact assessment protocols play a crucial role in the management of biological invasions, offering structured approaches to evaluating risks (González‐Moreno *et al*., 2019). One of their main strengths is standardisation, which enables comparisons across regions and taxa. By providing a clear classification system, protocols such as EICAT and GISS help identify high‐risk species and guide resource allocation for management interventions (Blackburn *et al*., 2011; Hawkins *et al*., 2015; Nentwig *et al*., 2016). Many of these assessments also have direct policy relevance, informing biosecurity regulations, blacklists, and early warning systems that support conservation planning (Essl *et al*., 2011b; Gederaas *et al*., 2012). Additionally, protocols like Harmonia+ and the GB‐NNRA, primarily comprehensive risk assessment frameworks, also offer scalability for impact assessments, allowing their application at different spatial levels, from local conservation areas to national policy contexts (Baker *et al*., 2008; Branquart, 2009; Mumford *et al*., 2010).

**Table 3:** Summary of different impact assessment methods, addressing the impact assessed (e.g. ecological, economic, socio‐ecological), how the method is applied, and the nature of the method, whether proactive (impact can be assessed with no prior knowledge of the species' invasion history) or reactive (reliant on impacts exerted elsewhere).

Method	Impact assessed	Description	Proactive/reactive	Reference
Relative Impact Potential	Ecological	Non‐native species impact calculated and compared to that of trophic analogue (e.g. native, other non‐native). Impact defined as the product of *per capita* resource consumption rate and a proxy of numerical response	Both	Dick *et al*. (2017); Laverty *et al*. (2017); Dickey *et al*. (2020)
InvaCost	Economic	Global database of the monetary costs of invasive species	Reactive	Diagne *et al*. (2020)
EICAT/EICAT+	Ecological	Twelve ecological impact categories considered by EICAT: (1) Competition; (2) Predation; (3) Hybridisation; (4) Transmission of diseases to native species; (5) Parasitism; (6) Poisoning/toxicity; (7) Biofouling; (8) Grazing/herbivory/browsing; (9, 10, 11) Chemical, physical, or structural impact on ecosystem; and (12) Interaction with other non‐native species. Severity of impacts classified as Minimal Concern (MC), Minor (MN), Moderate (MO), Major (MR), Massive (MV). Ten ecological impact categories considered by EICAT+: (1) Provision of trophic resources; (2) Overcompensation; (3) Hybridisation; (4) Disease reduction; (5) Dispersal facilitation; (6) Epibiosis or other direct provisions of habitat; (7) Chemical impact on ecosystem; (8) Physical impact on ecosystem; (9) Structural impact on ecosystem; and (10) Indirect impacts through interactions with other taxa. Strength of impacts classified as Minimal Positive Impact (ML+), Minor Positive Impact (MN+), Moderate Positive Impact (MO+), Major Positive Impact (MR+) and Massive Positive Impact (MV+).	Reactive	Hawkins *et al*. (2015); Evans *et al*. (2016); Volery *et al*. (2020); Vimercati *et al*. (2022)
SEICAT	Socio‐economic	Impact categories: (1) Safety; (2) Material and immaterial assets; (3) Health; (4) social, spiritual, and cultural relations. Severity classified as per EICAT.	Reactive	Bacher *et al*. (2018); Galanidi *et al*. (2018)
GISS (generic impact scoring system	Ecological, socio‐economic	Two classes (ecological and socio‐economic), six questions each, impact scores between 0 (no data) and 5 (major, large‐scale impact) for each question. Potential impact scores out of 60. Actual Impact Scores account for the percentage of area occupied in Europe.	Reactive	Nentwig *et al*. (2010, 2016); Kumschick & Nentwig (2010); Vaes‐Petignat & Nentwig (2014); Laverty *et al*. (2015); van der Veer & Nentwig (2015); Novoa *et al*. (2016)
Ricciardi & Cohen system	Ecological	Impact ranked on ordinal scale, with the highest rank reserved for invaders that have caused near‐total extirpations of multiple native species in multiple regions. Rankings assigned based on maximum impact documented at any site within invaded range.	Reactive	Ricciardi & Cohen (2007)
Biological Invasion Impact / Biopollution Assessment System (BINPAS)	Ecological	Online application for non‐native species impact assessment based on a classification of the abundance and distribution range of non‐native species related to the magnitude of their impacts on communities, habitats, and ecosystem functioning. A ‘Biopollution Level’ (BPL), ranging from ‘no measurable impact’ (BPL = 0) to ‘massive impact’ (BPL = 4) is assigned.	Reactive	Narščius *et al*. (2012)

However, despite their widespread use, these protocols differ in their approaches, data requirements, and applicability, which can lead to inconsistencies in how species are ranked and managed (Essl *et al*., 2011b; González‐Moreno *et al*., 2019). Assessor subjectivity and context dependency remain persistent limitations, often leading to conflicting outcomes. As a result, different protocols may yield different rankings for the same species (Vilà *et al*., 2019). A key challenge is subjectivity and assessor bias, as many impact assessments rely on expert judgment, which can lead to inconsistencies in scoring (Gilovich, Griffin & Kahneman, 2002; Cano‐Barbacil, Radinger & García‐Berthou, 2020). This can be mitigated by assessments involving multiple experts working jointly (e.g. Dodd *et al*., 2022) – an approach that has been shown to increase the level of confidence (Vilizzi *et al*., 2022a). Additionally, many protocols do not account fully for uncertainty, making it difficult to evaluate species with limited impact data or those whose effects vary depending on environmental conditions (Molnar *et al*., 2008). Differences in assessment scope and criteria can also lead to discrepancies between protocols, as some prioritise ecological impacts while others incorporate socio‐economic dimensions (Blackburn *et al*., 2011; Bacher *et al*., 2018). Also, the rigidity and oversimplification of these tools often fail to capture cascading effects, ecosystem‐level interactions, or long‐term consequences. Exerted impacts can be highly variable across different contexts, including ecosystems and environmental conditions, meaning that a species classified as harmful in one region may have negligible effects or even beneficial effects in another (Green & Crowe, 2014; Kuebbing, 2020). For instance, context‐dependent impacts like those observed in the pumpkinseed sunfish highlight the challenges of species‐level classifications (Jackson *et al*., 2016; Copp *et al*., 2017) depending on biotic pressures and habitat structure (Top *et al*., 2016; Santamaría *et al*., 2022). The reliance on species‐level assessments also disregards the role of ‘invasion syndromes’, where multiple invaders interact in ways that amplify or mitigate impacts (Novoa *et al*., 2020).

Impact assessments guide species prioritisation, resource allocation, and the development of biosecurity measures, including blacklist and early warning systems (Essl *et al*., 2011b; Leung *et al*., 2012). For example, EICAT and GISS have been instrumental in identifying species that pose significant ecological threats, enabling proactive management before widespread damage occurs (Blackburn *et al*., 2011; Hawkins *et al*., 2015; Nentwig *et al*., 2016). In addition to species prioritisation, impact assessments inform regulatory measures such as restrictions on trade and transport, quarantine protocols, and eradication programmes (Schrader *et al*., 2012), including numerous national and international policies (Turbé *et al*., 2017). They are integral to horizon scanning exercises that predict emerging threats before establishment by evaluating the species‐specific likelihood of introduction, establishment, spread, and impact (Roy *et al*., 2014; Peyton *et al*., 2019, 2020). Although forecasting impacts is inherently more difficult than predicting arrival or establishment, structured expert elicitation can help address knowledge gaps (Cano‐Barbacil *et al*., 2023). Finally, impact assessments support transparent stakeholder communication and science‐informed decision‐making, but further harmonisation and integration of uncertainty remain key areas for improvement (Vanderhoeven *et al*., 2017). By presenting non‐native species impacts in a structured manner, these assessments help facilitate discussions among scientists, decision‐makers, and the public, ensuring that conservation strategies are both scientifically informed and socially acceptable. Despite their influence, inconsistencies across frameworks can lead to challenges in policy implementation. Different methodologies may produce conflicting assessments for the same species, resulting in varying management recommendations and highlighting the need for greater harmonisation between frameworks (González‐Moreno *et al*., 2019) and future directions of assessment‐related research (Appendix S3).

### (4) Challenges in standardising impact assessments

The integration of socio‐cultural impacts into impact assessment frameworks remains a developing field, as these impacts are often subjective and difficult to quantify. However, accounting for these impacts can make non‐native species management more democratic and socially legitimate, whereas failing to account for these can lead to inequity, and distrust and resentment from stakeholders who are affected by unwanted legislation or eradication projects (Crowley, Hinchliffe & McDonald, 2017b). The SEICAT protocol focuses on how non‐native species affect human well‐being, using the impact categories of (*i*) safety, (*ii*) material and immaterial assets, (*iii*) health, and (*iv*) social, spiritual and cultural relations. Bacher *et al*. (2018) give the example of non‐native species such as wasps, mosquitos, and jellyfish that can render outdoor recreation areas unsuitable for activities due to the threats they pose to human health. Even though application of the SEICAT is dependent on available data, human perceptions of data‐deficient non‐native species can be acquired through interviews and questionnaires with the general public, specific communities, business owners, and wildlife managers (e.g. Moesch *et al*., 2024). Although not strictly qualifying as impact assessments, newer multi‐factor frameworks such as EICAT and SEICAT aim to incorporate multiple dimensions of impact while accounting for regional and ecosystem‐specific variability. Further, they attempt to assess the ‘contextual severity’ of invasions by incorporating factors such as trophic interactions, ecosystem feedback loops, and environmental degradation levels.

The increasing complexity of impact assessment protocols has therefore highlighted the inherent trade‐off between simplicity and accuracy. While simple metrics like biomass or abundance offer ease of use and broad applicability, they often fail to capture the nuanced effects of non‐native species, especially when interactions with other species, functional distinctiveness, or environmental factors play a crucial role (Parker *et al*., 1999). Conversely, multi‐factor approaches provide a more detailed assessment but require substantial data input and can introduce subjectivity in scoring impact severity (Hawkins *et al*., 2015). A major unresolved issue is how to value social and cultural impacts, as these dimensions are shaped by human perception and vary across regions and cultures (Shackleton *et al*., 2019c). This is further complicated in scenarios where social, cultural, and economic benefits are derived from a species known to be ecologically damaging, thereby complicating management decisions. Additionally, differences in spatial scale further complicate impact classification, i.e. what is deemed a severe impact at a local level might be negligible when considered globally, and *vice versa*. The need for more standardised, context‐sensitive methodologies remains critical, yet finding a balance between broad applicability and site‐specific relevance continues to be a challenge. Future impact assessment protocols must aim for greater adaptability, integrating ecological, economic, and socio‐cultural factors while ensuring that assessments remain practical for management and policy applications.

## VI. HOW IMPACTS AFFECT MANAGEMENT DECISIONS

### (1) Policy frameworks and legislative measures

Political actions are central to managing the spread and impact of non‐native species, as they shape regulatory frameworks, allocate resources, and drive international cooperation (Early *et al*., 2016). Strong legislative measures, trade regulations, and enforcement mechanisms are essential to mitigating the ecological, economic, and social impacts posed by biological invasions (Genovesi *et al*., 2015; Banerjee *et al*., 2021). A common problem related to any political action against biological invasions is that institutions responsible for non‐native species management vary by region and jurisdiction, but typically include governmental environment agencies, international regulatory bodies, and non‐governmental organisations (Shine, Williams & Gündling, 2000). These institutions assess non‐native species impacts based on scientific studies, risk assessments, and economic analyses. However, their effectiveness is often inadequate due to inconsistent criteria, political considerations, and a lack of coordination and enforcement of the already existent law across regions and larger political entities (Haubrock *et al*., 2024a). For example, the European Union's list of Invasive Alien Species of Union Concern is a legally binding document for Member States, with its selection process being based on a review of documented impacts and potential spread (i.e. evidence: Genovesi *et al*., 2015). By contrast, national and local agencies may have differing criteria, leading to inconsistencies or even contradictions in how species are managed (Balzani *et al*., 2022). Additionally, economic and political interests frequently influence these decisions, with certain species being overlooked or downplayed due to their commercial value or lack of immediate economic harm. Therefore, policymakers must prioritise science‐based decision‐making, strengthen biosecurity protocols, and foster global and intersectoral collaboration to prevent the introduction and establishment of non‐native species (Perrings *et al*., 2005). Additionally, integrating public awareness campaigns, stakeholder engagement, and adaptive governance strategies can enhance policy effectiveness, ensuring long‐term ecological resilience and sustainable management of non‐native species (Novoa *et al*., 2017; Hulme, 2024). Nevertheless, 83% of countries globally do not have national legislation or regulations specifically on invasive non‐native species (Roy *et al*., 2023b). In addition, invasion management increasingly interacts with climate adaptation and mitigation strategies, which may create both conflicts (e.g. afforestation with non‐native species) and synergies (e.g. ecosystem restoration improving resilience to invasions and climate extremes). Recognising these overlaps is important to avoid contradictory policy goals and to harness opportunities where biodiversity protection and climate adaptation align.

Among the countries with specific legislation on the issue, the European Union (EU) has established comprehensive policy frameworks and legislative measures to address the threats posed by non‐native species. A cornerstone of these efforts is EU Regulation No 1143/2014 ‘on the prevention and management of the introduction and spread of invasive alien species’ (EU, 2014). Member States are obligated to implement measures to prevent the introduction and spread of these species, conduct monitoring, and, where considered feasible, eradicate or manage populations to mitigate their impact (Tsiamis *et al*., 2017). To support these legislative measures, the EU has developed tools such as the European Alien Species Information Network (EASIN) (https://easin.jrc.ec.europa.eu/easin; Katsanevakis *et al*., 2015), which facilitates access to data on non‐native species reported in Europe, aiding in the implementation of this Regulation by providing information crucial for risk assessments, monitoring, and management strategies. Also, Australia and New Zealand have established comprehensive biosecurity frameworks, including Australia's Biosecurity Act 2015 and New Zealand's Biosecurity Act 1993, supported by targeted programmes such as the National Pest Plant Accord and species‐specific initiatives to prevent and manage non‐native species (Newfield & Champion, 2010; Durant & Faunce, 2018). Similarly, the USA has implemented stringent biosecurity measures and management strategies targeting non‐native species (Burkett *et al*., 2021; U.S. Department of the Interior, 2021).

### (2) Species‐based ‘black lists’, challenges, and limitations

Management decisions often rely on assessments of impact, reflecting a shift in priorities from preventing species spread to minimising damage, especially when limited resources demand careful prioritisation. One approach to identify priorities for management actions is the compilation of lists of potentially problematic species. Impact‐based non‐native species lists, often referred to as ‘black lists’ or ‘deny lists’, are widely used by stakeholders and invasion scientists to identify and regulate species deemed as problematic due to their potential ecological, economic, or socio‐cultural impacts (Essl *et al*., 2011b; Appendix S4). These lists serve as important tools for policy and conservation, allowing for targeted prevention, early detection (i.e. biosecurity), and control and containment strategies (Simberloff, 2006), and can be implemented at various administrative levels, despite administrative complexities (see Appendix S5). The Japan Invasive Alien Species Act, enacted in 2004, provides a noteworthy example of such a blacklist‐based approach. By restricting the importation, possession, rearing, and release of approximately 100 designated invasive species, it has been associated with marked declines in the importation of listed taxa, including mammals, birds, amphibians, reptiles, and ornamental fish (Goka, 2010).

A first attempt to create a black list of non‐native species in Europe was compiled by Genovesi & Scalera (2007) and was formally approved by the Standing Committee of the Bern Convention through Recommendation No. 125 (Council of Europe, 2007) of the Standing Committee on trade in invasive and potentially invasive non‐native species in Europe. Another prominent example is EU Regulation No. 1143/2014, which requires EU Member States to ban the trade, use, transportation, breeding, and possession of non‐native species in the Union list (Genovesi *et al*., 2015; Tollington *et al*., 2017). As of August 2025, this list includes 114 species (European Commission, 2022a). While no blacklists exist at the regional level as defined by the EU (i.e. covering multiple Member States), specific lists of species of national concern are being developed in accordance with EU Regulation No. 1143/2014. Cerri *et al*. (2022) noted that even before the Regulation, 25 EU countries had established some sort of national blacklists, and four have also adopted subnational (within‐country) lists. It is also worth noting that other types of lists exist, always for the purpose of prioritising efforts for management and surveillance, for example watch lists and alert lists which have been developed through screening, including dedicated horizon scanning exercises (see Roy *et al*., 2019) or similar prioritisation exercises (Carboneras *et al*., 2018).

## VII. CONCLUSIONS

(1) Biological invasions are a global threat to biodiversity and human well‐being. Governance responses, however, remain largely fragmented across scales, sectors, and borders. Addressing biological invasions and their impacts therefore requires more than isolated interventions: it demands a globally coordinated framework rooted in scientific information, political will, and public engagement (Li *et al*., 2024).
(2) The transboundary nature of biological invasions and their impacts challenge existing legal and institutional architectures, which often resemble a patchwork of reactive and regionally limited measures. Strengthening international agreements and fostering sustainable, transdisciplinary networks such as INVASIVESNET provide a pathway toward effective, long‐term solutions through shared knowledge, resource pooling, and harmonised strategies (Lucy *et al*., 2016). Such networks must explicitly integrate ecology, economics, social sciences, and policy to generate actionable, context‐sensitive solutions that transcend disciplinary silos.
(3) Global integration must remain responsive to the complexity and context dependencies that define invasion impacts. These impacts vary by the numerous contexts, including species, ecosystems, and cultural settings; what is ecologically harmful in one region may be ecologically benign in another with potentially differing socio‐economic implications that might be beneficial or detrimental.
(4) As such, coordinated actions and assessments must embrace flexibility and be locally informed, rather than applying a one‐size‐fits‐all mandate globally (Haubrock *et al*., 2024b; Sousa *et al*., 2024).
(5) The fragmentation of methodologies and persistent data asymmetries across regions hinder cross‐comparability and strategic foresight. A key pillar of coordination must therefore involve standardising monitoring and impact assessments, enhancing data interoperability, and investing in open‐access, global databases that equitably represent diverse geographies (Pergl *et al*., 2019).
(6) Societal perceptions – deeply rooted in cultural, economic, and historical contexts – profoundly shape how invasion impacts are defined, interpreted, and acted upon. Effective governance cannot rely solely on scientific consensus; it must also engage with pluralistic values, local knowledge systems, and participatory decision‐making (Kapitza *et al*., 2019).

## AUTHOR CONTRIBUTIONS

The study was conceptualised by P. J. H., with key input and support from T. E., R. Sou., and J. R. B. P. J. H. and T. E. contributed equally as first authors and led the development of the manuscript, carrying out the majority of the preliminary writing, editing, and coordination. The first draft was collaboratively written with significant contributions from N. A. S. A., J. B., V. D., J. W. E. D., A. C. S. F., E. G.‐B., S. K., N. I. K., S. M., M. A. N., B. P., R. Sca., I. S., D. S., A. S. T., and L. V., and critically reviewed, complemented, and edited by T. A., P. B., E. B., R. B., A. L. B., D. B., J. E. B., C. C.‐B., G. C., J. T. A. D., V. D. A., R. D. D., M. F., M. Ken., A. K., M. Kou., I. K., I. M.‐F., O. M., J. D. O., B. E. S., J. T., and H. V. Senior authors R. Sou. and J. R. B. provided supervisory roles throughout the project and made substantial contributions to refining and structuring the manuscript, with J. R. B. additionally serving as a key editorial lead during the final stages.

## CONFLICTS OF INTEREST

None of the authors have a conflict of interest to disclose.

## Supporting information


**Appendix S1.** Conducted literature search.

**Appendix S2.** The need for a standardised terminological framework.

**Appendix S3.** Directions for future research.

**Appendix S4.** Criticism of so‐called ‘black lists’.

**Appendix S5.** Conflicting goals in environmental governance.

**Fig. S1.** Annual number of publications on the impact of non‐native species and as a proportion of all publications listed in the field of Ecology in the Web of Science, highlighting the rise of invasion biology in the context of overall science output.

## Data Availability

Data sharing not applicable to this article as no datasets were generated or analysed during the current study.

## References

[brv70124-bib-0001] Acevedo‐Limón, L. , Oficialdegui, F. J. , Sánchez, M. I. & Clavero, M. (2020). Historical, human, and environmental drivers of genetic diversity in the red swamp crayfish (*Procambarus clarkii*) invading the Iberian Peninsula. Freshwater Biology 65, 1460–1474.

[brv70124-bib-0002] Adams, M. J. , Pearl, C. A. & Bruce Bury, R. (2003). Indirect facilitation of an anuran invasion by non‐native fishes. Ecology Letters 6, 343–351.

[brv70124-bib-0003] Addison, J. A. (2009). Distribution and impacts of invasive earthworms in Canadian forest ecosystems. Biological Invasions 11, 59–79.

[brv70124-bib-0004] Ahmed, D. A. , Haubrock, P. J. , Cuthbert, R. N. , Bang, A. , Soto, I. , Balzani, P. , Tarkan, A. S. , Macêdo, R. L. , Carneiro, L. , Bodey, T. W. , Oficialdegui, F. J. , Courtois, P. , Kourantidou, M. , Angulo, E. , Heringer, G. , *et al*. (2023). Recent advances in availability and synthesis of the economic costs of biological invasions. Bioscience 73, 560–574. 37680688 10.1093/biosci/biad060 PMC10481418

[brv70124-bib-0005] Ahmed, D. A. , Hudgins, E. J. , Cuthbert, R. N. , Kourantidou, M. , Diagne, C. , Haubrock, P. J. , Leung, B. , Liu, C. , Leroy, B. , Petrovskii, S. , Beidas, A. & Courchamp, F. (2022). Managing biological invasions: the cost of inaction. Biological Invasions 24, 1927–1946.

[brv70124-bib-0006] Ahmed, D. A. , Sousa, R. , Bortolus, A. , Aldemir, C. , Angeli, N. F. , Błońska, D. , Briski, E. , Britton, J. R. , Cano‐Barbacil, C. , Clark‐Ginsberg, A. , Culic, I. , Cuthbert, R. N. , Dick, J. , Dimarco, R. D. , Essl, F. , *et al*. (2025). Parallels and discrepancies between non‐native species introductions and human migration. Biological Reviews 100, 1365–1395. 39980263 10.1111/brv.70004 PMC12120396

[brv70124-bib-0007] Aizen, M. A. , Morales, C. L. & Morales, J. M. (2008). Invasive mutualists erode native pollination webs. PLoS Biology 6, e31. 18271628 10.1371/journal.pbio.0060031 PMC2235906

[brv70124-bib-0008] Alban, D. H. & Berry, E. C. (1994). Effects of earthworm invasion on morphology, carbon, and nitrogen of a forest soil. Applied Soil Ecology 1, 243–249.

[brv70124-bib-0009] Albano, P. G. , Steger, J. , Bošnjak, M. , Dunne, B. , Guifarro, Z. , Turapova, E. , Hua, Q. , Kaufman, D. S. , Rilov, G. & Zuschin, M. (2021). Native biodiversity collapse in the eastern Mediterranean. Proceedings of the Royal Society B 288, 20202469. 33402072 10.1098/rspb.2020.2469 PMC7892420

[brv70124-bib-0010] Alexander, M. E. , Dick, J. T. A. , Weyl, O. L. F. , Robinson, T. B. & Richardson, D. M. (2014). Existing and emerging high impact invasive species are characterized by higher functional responses than natives. Biology Letters 10, 20130946. 24522629 10.1098/rsbl.2013.0946 PMC3949369

[brv70124-bib-0011] Alexander, M. E. , Kaiser, H. , Weyl, O. L. F. & Dick, J. T. A. (2015). Habitat simplification increases the impact of a freshwater invasive fish. Environmental Biology of Fishes 98, 477–486.

[brv70124-bib-0012] Almas, A. D. (2017). Native Trees, Urban Forest Management Planning, and Residents: Knowledge, Attitudes, and Actions. University of Toronto, Toronto, ON, Canada.

[brv70124-bib-0013] Alves, H. , Gonçalves, D. , Nogueira, A. B. , Teixeira, A. , Padilha, J. & Sousa, R. (2025). Intrapopulation differences in biological traits and impacts in a highly invasive freshwater species. NeoBiota 97, 325–349.

[brv70124-bib-0014] Amaral, J. R. , Manna, L. R. , Mazzoni, R. , Neres‐Lima, V. , Marques, P. S. , El‐Sabaawi, R. W. & Zandonà, E. (2021). Testing the short‐term effects of a fish invader on the trophic ecology of a closely related species. Hydrobiologia 848, 2305–2318.

[brv70124-bib-0015] Amiotti, N. M. , Zalba, P. , Sanchez, L. F. & Peinemann, N. (2000). The impact of single trees on properties of loess‐derived grassland soils in Argentina. Ecology 81, 3283–3290.

[brv70124-bib-0016] Anagnostakis, S. L. (1987). Chestnut blight: the classical problem of an introduced pathogen. Mycologia 79, 23–37.

[brv70124-bib-0017] Andersen, M. C. , Adams, H. , Hope, B. & Powell, M. (2004). Risk assessment for invasive species. Risk Analysis 24, 787–793. 15357799 10.1111/j.0272-4332.2004.00478.x

[brv70124-bib-0018] Angulo, E. , Hoffmann, B. D. , Ballesteros‐Mejia, L. , Taheri, A. , Balzani, P. , Bang, A. , Renault, D. , Cordonnier, M. , Bellard, C. , Diagne, C. , Ahmed, D. A. , Watari, Y. & Courchamp, F. (2022). Economic costs of invasive alien ants worldwide. Biological Invasions 24, 2041–2060.

[brv70124-bib-0019] Anton, A. , Geraldi, N. R. , Ricciardi, A. & Dick, J. T. A. (2020). Global determinants of prey naiveté to exotic predators. Proceedings of the Royal Society B 287, 20192978. 32486977 10.1098/rspb.2019.2978 PMC7341919

[brv70124-bib-0020] Arribas, R. , Díaz‐Paniagua, C. & Gomez‐Mestre, I. (2014). Ecological consequences of amphibian larvae and their native and alien predators on the community structure of temporary ponds. Freshwater Biology 59, 1996–2008.

[brv70124-bib-0021] Atiglo, D. Y. , Jayson‐Quashigah, P.‐N. , Sowah, W. , Tompkins, E. L. & Addo, K. A. (2024). Misperception of drivers of risk alters willingness to adapt in the case of sargassum influxes in West Africa. Global Environmental Change 84, 102779.

[brv70124-bib-0022] Atkinson, M. S. & Savage, A. E. (2023). Invasive amphibians alter host‐pathogen interactions with primarily negative outcomes for native species. Biological Conservation 286, 110310.

[brv70124-bib-0023] Augustyniak, M. , Preiszner, B. , Kobak, J. , Czeglédi, I. , Kakareko, T. , Erős, T. , Cuthbert, R. N. & Jermacz, Ł. (2025). Global warming affects foraging efficiency of fish by influencing mutual interference. Journal of Animal Ecology 94, 811–1111. 10.1111/1365-2656.70003 39935274

[brv70124-bib-0024] Bacher, S. , Blackburn, T. M. , Essl, F. , Genovesi, P. , Heikkilä, J. , Jeschke, J. M. , Jones, G. , Keller, R. , Kenis, M. , Kueffer, C. , Martinou, A. F. , Nentwig, W. , Pergl, J. , Pyšek, P. , Rabitsch, W. , *et al*. (2018). Socio‐economic impact classification of alien taxa (SEICAT). Methods in Ecology and Evolution 9, 159–168.

[brv70124-bib-0025] Bacher, S. , Galil, B. , Nunez, M. , Ansong, M. , Cassey, P. , Dehnen‐Schmutz, K. , Fayvush, G. , Hiremath, A. , Ikegami, M. , Martinou, A. , McDermott, S. , Preda, C. , Vila, M. , Weyl, O. L. F. , Fernandez, R. , *et al*. (2023a). Impacts of invasive alien species on nature, nature's contributions to people, and good quality of life. In IPBES Invasive Alien Species Assessment, pp. 1–222. IPBES Secretariat, Bonn, Germany.

[brv70124-bib-0026] * Bacher, S. , Galil, B. S. , Nuñez, M. A. , Ansong, M. , Cassey, P. , Dehnen‐Schmutz, K. , Fayvush, G. , Hiremath, A. J. , Ikegami, M. , Martinou, A. F. , McDermott, S. M. , Preda, C. , Vilà, M. , Weyl, O. L. F. , Fernandez, R. D. , *et al*. (2023 *b*). Chapter 4: impacts of invasive alien species on nature, nature's contributions to people, and good quality of life. In Thematic Assessment Report on Invasive Alien Species and their Control of the Intergovernmental Science‐Policy Platform on Biodiversity and Ecosystem Services (eds H. E. Roy , A. Pauchard , P. Stoett and T. Renard Truong ). IPBES secretariat, Bonn, Germany.

[brv70124-bib-0027] * Bae, M.‐J. , Murphy, C. A. & García‐Berthou, E. (2018). Temperature and hydrologic alteration predict the spread of invasive Largemouth Bass (*Micropterus salmoides*). Science of the Total Environment 639, 58–66. 29778682 10.1016/j.scitotenv.2018.05.001

[brv70124-bib-0028] Bahlai, C. A. , Colunga‐Garcia, M. , Gage, S. H. & Landis, D. A. (2015). The role of exotic ladybeetles in the decline of native ladybeetle populations: evidence from long‐term monitoring. Biological Invasions 17, 1005–1024.

[brv70124-bib-0029] * Banha, F. , Diniz, A. & Anastácio, P. M. (2019). Patterns and drivers of aquarium pet discharge in the wild. Ecological Indicators 106, 105513.

[brv70124-bib-0030] Baker, R. , Black, R. , Copp, G. H. , Haysom, K. , Hulme, P. E. , Thomas, M. & Ellis, M. (2008). The UK risk assessment scheme for all non‐native species. In Biological Invasions: From Ecology to Conservation (eds W. Rabitsch , F. Essl and F. Klingenstein ), pp. 46–57. Institute of Ecology of the TU Berlin, Berlin.

[brv70124-bib-0031] Balzani, P. , Dekoninck, W. , Feldhaar, H. , Freitag, A. , Frizzi, F. , Frouz, J. , Masoni, A. , Robinson, E. , Sorvari, J. & Santini, G. (2022). Challenges and a call to action for protecting European red wood ants. Conservation Biology 36, e13959. 35638587 10.1111/cobi.13959 PMC10086985

[brv70124-bib-0032] Balzani, P. , Vizzini, S. , Santini, G. , Masoni, A. , Ciofi, C. , Ricevuto, E. & Chelazzi, G. (2016). Stable isotope analysis of trophic niche in two co‐occurring native and invasive terrapins, *Emys orbicularis* and *Trachemys scripta elegans* . Biological Invasions 18, 3611–3621.

[brv70124-bib-0033] Banerjee, A. K. , Khuroo, A. A. , Dehnen‐Schmutz, K. , Pant, V. , Patwardhan, C. , Bhowmick, A. R. & Mukherjee, A. (2021). An integrated policy framework and plan of action to prevent and control plant invasions in India. Environmental Science & Policy 124, 64–72.

[brv70124-bib-0034] Baptista, M. M. , Ramos, M. A. , de Albuquerque, U. P. , Coelho‐de‐Souza, G. & Ritter, M. R. (2013). Traditional botanical knowledge of artisanal fishers in southern Brazil. Journal of Ethnobiology and Ethnomedicine 9, 1–16. 23898973 10.1186/1746-4269-9-54 PMC3737040

[brv70124-bib-0035] Barnard, T. P. (2015). The annotated Malay archipelago by Alfred Russel Wallace. Journal of the Malaysian Branch of the Royal Asiatic Society 88, 183–184.

[brv70124-bib-0036] Barney, J. N. & Tekiela, D. R. (2020). Framing the concept of invasive species “impact” within a management context. Invasive Plant Science and Management 13, 37–40.

[brv70124-bib-0037] Barney, J. N. , Tekiela, D. R. , Dollete, E. S. J. & Tomasek, B. J. (2013). What is the “real” impact of invasive plant species? Frontiers in Ecology and the Environment 11, 322–329.

[brv70124-bib-0038] Barratt, J. , Chan, D. , Sandaradura, I. , Malik, R. , Spielman, D. , Lee, R. , Marriott, D. , Harkness, J. , Ellis, J. & Stark, D. (2016). *Angiostrongylus cantonensis*: a review of its distribution, molecular biology and clinical significance as a human pathogen. Parasitology 143, 1087–1118. 27225800 10.1017/S0031182016000652

[brv70124-bib-0039] Bartomeus, I. , Vilà, M. & Santamaría, L. (2008). Contrasting effects of invasive plants in plant–pollinator networks. Oecologia 155, 761–770. 18188603 10.1007/s00442-007-0946-1

[brv70124-bib-0040] Bass, D. , Stentiford, G. D. , Wang, H.‐C. , Koskella, B. & Tyler, C. R. (2019). The pathobiome in animal and plant diseases. Trends in Ecology & Evolution 34, 996–1008. 31522755 10.1016/j.tree.2019.07.012 PMC7479508

[brv70124-bib-0041] Baxter, C. V. , Fausch, K. D. & Saunders, W. C. (2005). Tangled webs: reciprocal flows of invertebrate prey link streams and riparian zones. Freshwater Biology 50, 201–220.

[brv70124-bib-0042] Beja, P. R. (1996). An analysis of otter *Lutra lutra* predation on introduced American crayfish *Procambarus clarkii* in Iberian streams. Journal of Applied Ecology 33, 1156–1170.

[brv70124-bib-0043] Bellard, C. , Bernery, C. & Leclerc, C. (2021). Looming extinctions due to invasive species: irreversible loss of ecological strategy and evolutionary history. Global Change Biology 27, 4967–4979. 34337834 10.1111/gcb.15771

[brv70124-bib-0044] Bellard, C. , Cassey, P. & Blackburn, T. M. (2016). Alien species as a driver of recent extinctions. Biology Letters 12, 20150623. 26888913 10.1098/rsbl.2015.0623 PMC4780541

[brv70124-bib-0045] Bellard, C. & Jeschke, J. M. (2016). A spatial mismatch between invader impacts and research publications. Conservation Biology 30, 230–232. 26308661 10.1111/cobi.12611

[brv70124-bib-0046] Benito, J. , Benejam, L. , Zamora, L. & García‐Berthou, E. (2015). Diel cycle and effects of water flow on activity and use of depth by common carp. Transactions of the American Fisheries Society 144, 491–501.

[brv70124-bib-0047] Bennett, S. , Santana‐Garcon, J. , Marbà, N. , Jorda, G. , Anton, A. , Apostolaki, E. T. , Cebrian, J. , Geraldi, N. R. , Krause‐Jensen, D. , Lovelock, C. E. , Martinetto, P. , Pandolfi, J. M. & Duarte, C. M. (2021). Climate‐driven impacts of exotic species on marine ecosystems. Global Ecology and Biogeography 30, 1043–1055.

[brv70124-bib-0048] Bergstrom, D. M. , Lucieer, A. , Kiefer, K. , Wasley, J. , Belbin, L. , Pedersen, T. K. & Chown, S. L. (2009). Indirect effects of invasive species removal devastate world Heritage Island. Journal of Applied Ecology 46, 73–81.

[brv70124-bib-0049] Berke, S. K. (2010). Functional groups of ecosystem engineers: a proposed classification with comments on current issues. Integrative and Comparative Biology 50, 147–157. 21558195 10.1093/icb/icq077

[brv70124-bib-0050] Bertolino, S. & Genovesi, P. (2003). Spread and attempted eradication of the grey squirrel (*Sciurus carolinensis*) in Italy, and consequences for the red squirrel (*Sciurus vulgaris*) in Eurasia. Biological Conservation 109, 351–358.

[brv70124-bib-0051] Bertolino, S. & Genovesi, P. (2007). Semiaquatic mammals introduced into Italy: case studies in biological invasion. In Biological Invaders in Inland Waters: Profiles, Distribution, and Threats Pp, pp. 75–191. Springer Netherlands, Dordrecht.

[brv70124-bib-0052] Bertolino, S. , Guichón, M. L. , Carter, J. & Francis, R. A. (2012). Myocastor Coypus Molina (Coypu). A Handbook of Global Freshwater Invasive Species, 357–368. Earthscan, London, UK.

[brv70124-bib-0053] Bertolino, S. , di Montezemolo, N. C. , Preatoni, D. G. , Wauters, L. A. & Martinoli, A. (2014). A grey future for Europe: *Sciurus carolinensis* is replacing native red squirrels in Italy. Biological Invasions 16, 53–62.

[brv70124-bib-0054] Bezerra‐Santos, M. A. , Dantas‐Torres, F. , Mendoza‐Roldan, J. A. , Thompson, R. C. A. , Modry, D. & Otranto, D. (2023). Invasive mammalian wildlife and the risk of zoonotic parasites. Trends in Parasitology 39, 786–798. 37429777 10.1016/j.pt.2023.06.004

[brv70124-bib-0055] Bissattini, A. M. , Buono, V. & Vignoli, L. (2018). Field data and worldwide literature review reveal that alien crayfish mitigate the predation impact of the American bullfrog on native amphibians. Aquatic Conservation: Marine and Freshwater Ecosystems 28, 1465–1475.

[brv70124-bib-0056] Bissattini, A. M. , Haubrock, P. J. , Buono, V. , Balzani, P. , Borgianni, N. , Stellati, L. , Inghilesi, A. F. , Tancioni, L. , Martinoli, M. , Tricarico, E. & Vignoli, L. (2021). Trophic structure of a pond community dominated by an invasive alien species: insights from stomach content and stable isotope analyses. Aquatic Conservation: Marine and Freshwater Ecosystems 31, 948–963.

[brv70124-bib-0057] Blackburn, T. M. , Bellard, C. & Ricciardi, A. (2019). Alien versus native species as drivers of recent extinctions. Frontiers in Ecology and the Environment 17, 203–207.

[brv70124-bib-0058] Blackburn, T. M. , Cassey, P. , Duncan, R. P. , Evans, K. L. & Gaston, K. J. (2004). Avian extinction and mammalian introductions on oceanic islands. Science 305, 1955–1958. 15448269 10.1126/science.1101617

[brv70124-bib-0059] * Blackburn, T. M. , Essl, F. , Evans, T. , Hulme, P. E. , Jeschke, J. M. , Kühn, I. , Kumschick, S. , Marková, Z. , Mrugała, A. & Nentwig, W. (2014). A unified classification of alien species based on the magnitude of their environmental impacts. PLoS Biology 12, e1001850. 24802715 10.1371/journal.pbio.1001850 PMC4011680

[brv70124-bib-0060] Blackburn, T. M. , Pyšek, P. , Bacher, S. , Carlton, J. T. , Duncan, R. P. , Jarošík, V. , Wilson, J. R. U. & Richardson, D. M. (2011). A proposed unified framework for biological invasions. Trends in Ecology & Evolution 26, 333–339. 21601306 10.1016/j.tree.2011.03.023

[brv70124-bib-0061] Blackwell, T. , Ford, A. G. P. , Ciezarek, A. G. , Bradbeer, S. J. , Gracida Juarez, C. A. , Smith, A. M. , Ngatunga, B. P. , Shechonge, A. , Tamatamah, R. , Etherington, G. , Haerty, W. , Di Palma, F. , Turner, G. F. & Genner, M. J. (2021). Newly discovered cichlid fish biodiversity threatened by hybridization with non‐native species. Molecular Ecology 30, 895–911. 33063411 10.1111/mec.15638

[brv70124-bib-0062] * Bliss, C. , Visseren‐Hamakers, I. J. & Liefferink, D. (2023). Most (un) wanted: explaining emerging relationships between “invasive alien” species and animal governance. Global Environmental Politics 23, 26–51.

[brv70124-bib-0063] Błońska, D. , Grabowska, J. , Tarkan, A. S. , Soto, I. & Haubrock, P. J. (2024). Prioritising non‐native fish species for management actions in three polish rivers using the newly developed tool—dispersal‐origin‐status‐impact scheme. PeerJ 12, e18300. 39494268 10.7717/peerj.18300 PMC11531750

[brv70124-bib-0064] Bódis, E. , Tóth, B. & Sousa, R. (2014). Impact of *Dreissena* fouling on the physiological condition of native and invasive bivalves: interspecific and temporal variations. Biological Invasions 16, 1373–1386.

[brv70124-bib-0065] * Boggero, A. , Paganelli, D. , Zaupa, S. , Garzoli, L. , Vilizzi, L. & Kamburska, L. (2025). An integrated evaluation of the invasiveness risk posed by non‐native crayfish in Lake Maggiore (Northwest Italy). Management of Biological Invasions 16, 135–152.

[brv70124-bib-0066] Bohlen, P. J. , Groffman, P. M. , Fahey, T. J. , Fisk, M. C. , Suárez, E. , Pelletier, D. M. & Fahey, R. T. (2004 *a*). Ecosystem consequences of exotic earthworm invasion of north temperate forests. Ecosystems 7, 1–12.

[brv70124-bib-0067] Bohlen, P. J. , Pelletier, D. M. , Groffman, P. M. , Fahey, T. J. & Fisk, M. C. (2004 *b*). Influence of earthworm invasion on redistribution and retention of soil carbon and nitrogen in northern temperate forests. Ecosystems 7, 13–27.

[brv70124-bib-0068] Bohlen, P. J. , Scheu, S. , Hale, C. M. , McLean, M. A. , Migge, S. , Groffman, P. M. & Parkinson, D. (2004 *c*). Non‐native invasive earthworms as agents of change in northern temperate forests. Frontiers in Ecology and the Environment 2, 427–435.

[brv70124-bib-0069] Bojko, J. , Dunn, A. M. & Blakeslee, A. M. H. (2023). Parasites and Biological Invasions. CABI, Wallingford, Oxfordshire, UK.

[brv70124-bib-0070] Bojko, J. , Dunn, A. M. , Stebbing, P. D. , Ross, S. H. , Kerr, R. C. & Stentiford, G. D. (2015). *Cucumispora ornata* n. sp. (Fungi: Microsporidia) infecting invasive ‘demon shrimp’ (*Dikerogammarus haemobaphes*) in the United Kingdom. Journal of Invertebrate Pathology 128, 22–30. 25929755 10.1016/j.jip.2015.04.005

[brv70124-bib-0071] Bojko, J. , Stebbing, P. D. , Bateman, K. S. , Meatyard, J. E. , Bacela‐Spychalska, K. , Dunn, A. M. & Stentiford, G. D. (2013). Baseline histopathological survey of a recently invading Island population of ‘killer shrimp’, *Dikerogammarus villosus* . Diseases of Aquatic Organisms 106, 241–253. 24192001 10.3354/dao02658

[brv70124-bib-0072] Bojko, J. , Stentiford, G. , Stebbing, P. , Hassall, C. , Deacon, A. , Cargill, B. , Pile, B. & Dunn, A. (2019). Pathogens of *Dikerogammarus haemobaphes* regulate host activity and survival, but also threaten native amphipod populations in the UK. Diseases of Aquatic Organisms 136, 63–78. 31575835 10.3354/dao03195

[brv70124-bib-0073] Boltovskoy, D. , Guiaşu, R. , Burlakova, L. , Karatayev, A. , Schlaepfer, M. A. & Correa, N. (2022). Misleading estimates of economic impacts of biological invasions: including the costs but not the benefits. Ambio 51, 1786–1799. 35191001 10.1007/s13280-022-01707-1 PMC9200917

[brv70124-bib-0074] Boon, J. S. , Keith, S. A. , Exton, D. A. & Field, R. (2023). The role of refuges in biological invasions: a systematic review. Global Ecology and Biogeography 32, 1244–1271.

[brv70124-bib-0075] Boštjančić, L. L. , Francesconi, C. , Rutz, C. , Hoffbeck, L. , Poidevin, L. , Kress, A. , Jussila, J. , Makkonen, J. , Feldmeyer, B. , Bálint, M. , Schwenk, K. , Lecompte, O. & Theissinger, K. (2022). Host‐pathogen coevolution drives innate immune response to *Aphanomyces astaci* infection in freshwater crayfish: transcriptomic evidence. BMC Genomics 23, 600. 35989333 10.1186/s12864-022-08571-z PMC9394032

[brv70124-bib-0076] Both, C. & Grant, T. (2012). Biological invasions and the acoustic niche: the effect of bullfrog calls on the acoustic signals of white‐banded tree frogs. Biology Letters 8, 714–716. 22675139 10.1098/rsbl.2012.0412 PMC3440993

[brv70124-bib-0077] Boulêtreau, S. , Cucherousset, J. , Villeger, S. , Masson, R. & Santoul, F. (2011). Colossal aggregations of giant alien freshwater fish as a potential biogeochemical hotspot. PLoS One 6, e25732. 21998687 10.1371/journal.pone.0025732 PMC3187786

[brv70124-bib-0078] Bourret, S. L. , Kovach, R. P. , Cline, T. J. , Strait, J. T. & Muhlfeld, C. C. (2022). High dispersal rates in hybrids drive expansion of maladaptive hybridization. Proceedings of the Royal Society B 289, 20221813. 36350203 10.1098/rspb.2022.1813 PMC9653238

[brv70124-bib-0079] Bradshaw, C. J. A. , Hulme, P. E. , Hudgins, E. J. , Leung, B. , Kourantidou, M. , Courtois, P. , Turbelin, A. J. , McDermott, S. M. , Lee, K. , Ahmed, D. A. , Latombe, G. , Bang, A. , Bodey, T. W. , Haubrock, P. J. , Saltré, F. , *et al*. (2024). Damage costs from invasive species exceed management expenditure in nations experiencing lower economic activity. Ecological Economics 220, 108166.

[brv70124-bib-0080] Branquart, E. (2009). Guidelines for environmental impact assessment and list classification of non‐native organisms in Belgium. Version 2, 6.

[brv70124-bib-0081] Briski, E. , Bailey, S. A. , Casas‐Monroy, O. , DiBacco, C. , Kaczmarska, I. , Lawrence, J. E. , Leichsenring, J. , Levings, C. , MacGillivary, M. L. , McKindsey, C. W. , Nasmith, L. E. , Parenteau, M. , Piercey, G. E. , Rivkin, R. B. , Rochon, A. , *et al*. (2013). Taxon‐and vector‐specific variation in species richness and abundance during the transport stage of biological invasions. Limnology and Oceanography 58, 1361–1372.

[brv70124-bib-0082] Briski, E. , Chan, F. T. , Darling, J. A. , Lauringson, V. , MacIsaac, H. J. , Zhan, A. & Bailey, S. A. (2018). Beyond propagule pressure: importance of selection during the transport stage of biological invasions. Frontiers in Ecology and the Environment 16, 345–353. 31942166 10.1002/fee.1820 PMC6961837

[brv70124-bib-0083] Briski, E. , Langrehr, L. , Kotronaki, S. G. , Sidow, A. , Martinez Reyes, C. G. , Geropoulos, A. , Steffen, G. , Theurich, N. , Dickey, J. W. E. , Hütt, J. C. , Haubrock, P. J. , Soto, I. , Kouba, A. & Cuthbert, R. N. (2025). Urban environments promote adaptation to multiple stressors. Ecology Letters 28, e70074. 39967439 10.1111/ele.70074 PMC11836597

[brv70124-bib-0084] Britton, J. R. (2013). Introduced parasites in food webs: new species, shifting structures? Trends in Ecology & Evolution 28, 93–99. 22995896 10.1016/j.tree.2012.08.020

[brv70124-bib-0085] Britton, J. R. (2018). Empirical predictions of the trophic consequences of non‐native freshwater fishes: a synthesis of approaches and invasion impacts. Turkish Journal of Fisheries and Aquatic Sciences 19, 529–539.

[brv70124-bib-0086] Britton, J. R. (2023). Contemporary perspectives on the ecological impacts of invasive freshwater fishes. Journal of Fish Biology 103, 752–764. 36207758 10.1111/jfb.15240

[brv70124-bib-0087] Britton, J. R. , Andreou, D. , Boardman, R. , Garcia, F. , Gimenez, M. , Imbert, A. , Parker, B. , Warren, B. , Yeldham, M. & Cucherousset, J. (2024). The angled‐web: recreational angling as an underappreciated disruptor to the interconnectedness of terrestrial and freshwater food webs. Freshwater Biology 69, 1739–1747.

[brv70124-bib-0088] Britton, J. R. , Copp, G. H. , Brazier, M. & Davies, G. D. (2011). A modular assessment tool for managing introduced fishes according to risks of species and their populations, and impacts of management actions. Biological Invasions 13, 2847–2860.

[brv70124-bib-0089] Britton, J. R. , Harper, D. M. , Oyugi, D. O. & Grey, J. (2010). The introduced *Micropterus salmoides* in an equatorial lake: a paradoxical loser in an invasion meltdown scenario? Biological Invasions 12, 3439–3448.

[brv70124-bib-0090] Brooks, M. L. , D'antonio, C. M. , Richardson, D. M. , Grace, J. B. , Keeley, J. E. , DiTomaso, J. M. , Hobbs, R. J. , Pellant, M. & Pyke, D. (2004). Effects of invasive alien plants on fire regimes. Bioscience 54, 677–688.

[brv70124-bib-0091] Brown, B. J. , Mitchell, R. J. & Graham, S. A. (2002). Competition for pollination between an invasive species (purple loosestrife) and a native congener. Ecology 83, 2328–2336.

[brv70124-bib-0092] Brown, N. E. M. & Therriault, T. W. (2022). The hidden risk of keystone invaders in Canada: a case study using nonindigenous crayfish. Canadian Journal of Fisheries and Aquatic Sciences 79, 1479–1496.

[brv70124-bib-0093] Brunel, S. , Fernández‐Galiano, E. , Genovesi, P. , Heywood, V. H. , Kueffer, C. & Richardson, D. M. (2013). 20 invasive alien species: a growing but neglected threat? In Late Lessons from Early Warnings: Science, Precaution, Innovation (Volume 30), pp. 518–540. European Environment Agency (EEA), Copenhagen, Denmark.

[brv70124-bib-0094] Bruno, J. F. , Stachowicz, J. J. & Bertness, M. D. (2003). Inclusion of facilitation into ecological theory. Trends in Ecology & Evolution 18, 119–125.

[brv70124-bib-0095] Bruschetti, M. , Bazterrica, C. , Luppi, T. & Iribarne, O. (2009). An invasive intertidal reef‐forming polychaete affect habitat use and feeding behavior of migratory and locals birds in a SW Atlantic coastal lagoon. Journal of Experimental Marine Biology and Ecology 375, 76–83.

[brv70124-bib-0096] Brys, R. , Halfmaerten, D. , Van Driessche, C. , Everts, T. , De Beer, B. , Neyrinck, S. , Decru, E. & Verreycken, H. (2025). First record of black bullhead (*Ameiurus melas* Lesueur, 1819) and the occurrence of hybridization with brown bullhead (*a. nebulosus* Rafinesque, 1820) in Belgium. BioInvasions Record 14, 169–181.

[brv70124-bib-0097] Buckley, Y. M. & Catford, J. (2016). Does the biogeographic origin of species matter? Ecological effects of native and non‐native species and the use of origin to guide management. Journal of Ecology 104, 4–17.

[brv70124-bib-0098] Budy, P. & Gaeta, J. W. (2017). Brown trout as an invader: a synthesis of problems and perspectives in North America. Brown Trout: Biology, Ecology and Management 20, 523–543.

[brv70124-bib-0099] Burkett, D. P. , Barber, J. M. , Steeves, T. B. & Siefkes, M. J. (2021). Sea lamprey control 2020–2040: charting a course through dynamic waters. Journal of Great Lakes Research 47, S809–S814.

[brv70124-bib-0100] Burkett‐Cadena, N. D. , Blosser, E. M. , Loggins, A. A. , Valente, M. C. , Long, M. T. , Campbell, L. P. , Reeves, L. E. , Bargielowski, I. & McCleery, R. A. (2021). Invasive Burmese pythons alter host use and virus infection in the vector of a zoonotic virus. Communications Biology 4, 1–11. 34183751 10.1038/s42003-021-02347-z PMC8239020

[brv70124-bib-0101] Burlakova, L. E. , Karatayev, A. Y. & Karatayev, V. A. (2012). Invasive mussels induce community changes by increasing habitat complexity. Hydrobiologia 685, 121–134.

[brv70124-bib-0102] Burns, C. W. (2013). Predictors of invasion success by *Daphnia* species: influence of food, temperature and species identity. Biological Invasions 15, 859–869.

[brv70124-bib-0103] Butler, D. (1994). Bid to protect wolves from genetic pollution. Nature 370, 497.

[brv70124-bib-0104] Byers, J. E. (2000). Competition between two estuarine snails: implications for invasions of exotic species. Ecology 81, 1225–1239.

[brv70124-bib-0105] Byers, J. E. (2002). Impact of non‐indigenous species on natives enhanced by anthropogenic alteration of selection regimes. Oikos 97, 449–458.

[brv70124-bib-0106] Byers, J. E. (2024). Using ecosystem engineers to enhance multiple ecosystem processes. Functional Ecology 38, 22–36.

[brv70124-bib-0107] Cadotte, M. W. , Yasui, S. L. E. , Livingstone, S. & MacIvor, J. S. (2017). Are urban systems beneficial, detrimental, or indifferent for biological invasion? Biological Invasions 19, 3489–3503.

[brv70124-bib-0108] * Caffrey, J. M. , Baars, J.‐R. , Barbour, J. H. , Boets, P. , Boon, P. , Davenport, K. , Dick, J. T. A. , Early, J. , Edsman, L. , Gallagher, C. , Gross, J. , Heinimaa, P. , Horrill, C. , Hudin, S. & MacIsaac, H. J. (2014). Tackling invasive alien species in Europe: the top 20 issues. Management of Biological Invasions 5, 1–20.

[brv70124-bib-0109] Callaway, R. M. & Ridenour, W. M. (2004). Novel weapons: invasive success and the evolution of increased competitive ability. Frontiers in Ecology and the Environment 2, 436–443.

[brv70124-bib-0110] Campbell, J. N. , Mandeville, E. G. , Lewis, N. C. & Meuser, A. (2024). The ambiguity of “hybrid swarm”: inconsistent definitions and applications in existing research. bioRxiv, 2010–2024.

[brv70124-bib-0111] Campião, K. M. , da Luz Rico, J. A. , de Souza Monteiro, G. , Ash, L. V. , Teixeira, C. P. & Gotelli, N. J. (2024). High prevalence and concomitant infection of Ranavirus and *Eustrongylides* sp. in the invasive American Bullfrog in Brazil. Parasitology International 100, 102875. 38417736 10.1016/j.parint.2024.102875

[brv70124-bib-0112] Cano‐Barbacil, C. , Carrete, M. , Castro‐Díez, P. , Delibes‐Mateos, M. , Jaques, J. A. , López‐Darias, M. , Nogales, M. , Pino, J. , Ros, M. , Traveset, A. , Turon, X. , Vilà, M. , Altamirano, M. , Álvarez, I. , Arias, A. , *et al*. (2023). Identification of potential invasive alien species in Spain through horizon scanning. Journal of Environmental Management 345, 118696. 37549639 10.1016/j.jenvman.2023.118696

[brv70124-bib-0113] Cano‐Barbacil, C. , Haubrock, P. J. & Radinger, J. (2025). Asian loaches: an emerging threat as global invaders. Freshwater Biology 70, e70026.

[brv70124-bib-0115] Capinha, C. , Essl, F. , Seebens, H. , Moser, D. & Pereira, H. M. (2015). The dispersal of alien species redefines biogeography in the Anthropocene. Science 348, 1248–1251. 26068851 10.1126/science.aaa8913

[brv70124-bib-0116] Carboneras, C. , Genovesi, P. , Vilà, M. , Blackburn, T. M. , Carrete, M. , Clavero, M. , D'hondt, B. , Orueta, J. F. , Gallardo, B. , Geraldes, P. , González‐Moreno, P. , Gregory, R. D. , Nentwig, W. , Paquet, J.‐Y. , Pyšek, P. , *et al*. (2018). A prioritised list of invasive alien species to assist the effective implementation of EU legislation. Journal of Applied Ecology 55, 539–547.

[brv70124-bib-0118] Carlton, J. T. (2002). Bioinvasion ecology: assessing invasion impact and scale. In Invasive Aquatic Species of Europe. Distribution, Impacts and Management, pp. 7–19. Kluwer Academic Publishers, Dordrecht, The Netherlands.

[brv70124-bib-0119] Carmona‐Catot, G. , Magellan, K. & García‐Berthou, E. (2013). Temperature‐specific competition between invasive mosquitofish and an endangered cyprinodontid fish. PLoS One 8, e54734. 23382951 10.1371/journal.pone.0054734 PMC3555637

[brv70124-bib-0120] Carneiro, L. , Hulme, P. E. , Cuthbert, R. N. , Kourantidou, M. , Bang, A. , Haubrock, P. J. , Bradshaw, C. J. A. , Balzani, P. , Bacher, S. , Latombe, G. , Bodey, T. W. , Probert, A. F. , Quilodrán, C. S. & Courchamp, F. (2024 *a*). Benefits do not balance costs of biological invasions. Bioscience 74, 340–344.

[brv70124-bib-0121] Carneiro, L. , Leroy, B. , Capinha, C. , Bradshaw, C. J. A. , Bertolino, S. , Catford, J. A. , Camacho‐Cervantes, M. , Bojko, J. , Klippel, G. , Kumschick, S. , Pincheira‐Donoso, D. , Tonkin, J. D. , Fath, B. D. , South, J. , Manfrini, E. , *et al*. (2025). Typology of the ecological impacts of biological invasions. Trends in Ecology & Evolution 40, 563–574. 40280812 10.1016/j.tree.2025.03.010

[brv70124-bib-0122] Carneiro, L. , Miiller, N. O. R. , Cuthbert, R. N. & Vitule, J. R. S. (2024 *b*). Biological invasions negatively impact global protected areas. Science of the Total Environment 948, 174823. 39019276 10.1016/j.scitotenv.2024.174823

[brv70124-bib-0123] Carthey, A. J. R. & Banks, P. B. (2014). Naïveté in novel ecological interactions: lessons from theory and experimental evidence. Biological Reviews 89, 932–949. 25319946 10.1111/brv.12087

[brv70124-bib-0124] Cassini, M. H. (2020). A review of the critics of invasion biology. Biological Reviews 95, 1467–1478. 32515886 10.1111/brv.12624

[brv70124-bib-0125] Castilla, J. C. , Lagos, N. A. & Cerda, M. (2004). Marine ecosystem engineering by the alien ascidian *Pyura praeputialis* on a mid‐intertidal rocky shore. Marine Ecology Progress Series 268, 119–130.

[brv70124-bib-0126] Castorani, M. C. N. & Hovel, K. A. (2015). Invasive prey indirectly increase predation on their native competitors. Ecology 96, 1911–1922. 26378313 10.1890/14-1538.1

[brv70124-bib-0127] Castro‐Díez, P. , Godoy, O. , Alonso, A. , Gallardo, A. & Saldaña, A. (2014). What explains variation in the impacts of exotic plant invasions on the nitrogen cycle? A meta‐analysis. Ecology Letters 17, 1–12. 24134461 10.1111/ele.12197

[brv70124-bib-0128] Castro‐Díez, P. , González‐Muñoz, N. , Alonso, A. , Gallardo, A. & Poorter, L. (2009). Effects of exotic invasive trees on nitrogen cycling: a case study in Central Spain. Biological Invasions 11, 1973–1986.

[brv70124-bib-0129] Castro‐Díez, P. , Vaz, A. S. , Silva, J. S. , Van Loo, M. , Alonso, Á. , Aponte, C. , Bayón, Á. , Bellingham, P. J. , Chiuffo, M. C. , DiManno, N. , Julian, K. , Kandert, S. , La Porta, N. , Marchante, H. , Maule, H. G. , *et al*. (2019). Global effects of non‐native tree species on multiple ecosystem services. Biological Reviews 94, 1477–1501. 30974048 10.1111/brv.12511 PMC6850375

[brv70124-bib-0130] Catford, J. A. (2017). Hydrological impacts of biological invasions. Impact of Biological Invasions on Ecosystem Services 12, 63–80.

[brv70124-bib-0131] CBD (2010). Secretariat of the convention on biological diversity. Global biodiversity outlook 3 (UNEP/CBD/COP/10/INF/12/rev.1). Convention on Biological Diversity, Montreal, QC, Canada.

[brv70124-bib-0132] Cerri, J. , Carnevali, L. , Monaco, A. , Genovesi, P. & Bertolino, S. (2022). Blacklists do not necessarily make people curious about invasive alien species. A case study with Bayesian structural time series and Wikipedia searches about invasive mammals in Italy. NeoBiota 71, 113–128.

[brv70124-bib-0133] Chaffin, B. C. , Garmestani, A. S. , Angeler, D. G. , Herrmann, D. L. , Stow, C. A. , Nyström, M. , Sendzimir, J. , Hopton, M. E. , Kolasa, J. & Allen, C. R. (2016). Biological invasions, ecological resilience and adaptive governance. Journal of Environmental Management 183, 399–407. 27377866 10.1016/j.jenvman.2016.04.040

[brv70124-bib-0134] Chang, C.‐S. , Liu, H.‐L. , Moncada, X. , Seelenfreund, A. , Seelenfreund, D. & Chung, K.‐F. (2015). A holistic picture of Austronesian migrations revealed by phylogeography of Pacific paper mulberry. Proceedings of the National Academy of Sciences 112, 13537–13542. 10.1073/pnas.1503205112 PMC4640734 26438853

[brv70124-bib-0135] Chapuis, J. , Pisanu, B. , Brodier, S. , Villers, A. , Pettex, E. , Lioret, M. & Bretagnolle, V. (2011). Eradication of invasive herbivores: usefulness and limits for biological conservation in a changing world. Animal Conservation 14, 471–473.

[brv70124-bib-0136] Charles, H. & Dukes, J. S. (2007). Impacts of invasive species on ecosystem services. Biological Invasions 13, 217–237.

[brv70124-bib-0137] Charter, M. , Izhaki, I. , Mocha, Y. B. & Kark, S. (2016). Nest‐site competition between invasive and native cavity nesting birds and its implication for conservation. Journal of Environmental Management 181, 129–134. 27341373 10.1016/j.jenvman.2016.06.021

[brv70124-bib-0138] Chesson, P. (2000). Mechanisms of maintenance of species diversity. Annual Review of Ecology and Systematics 31, 343–366.

[brv70124-bib-0139] Chhaya, V. , Lahiri, S. , Jagan, M. A. , Mohan, R. , Pathaw, N. A. & Krishnan, A. (2021). Community bioacoustics: studying acoustic community structure for ecological and conservation insights. Frontiers in Ecology and Evolution 9, 706445.

[brv70124-bib-0140] Chiesa, S. , Petochi, T. , Brusà, R. B. , Raicevich, S. , Cacciatore, F. , Franceschini, G. , Antonini, C. , Vallini, C. , Bernarello, V. , Oselladore, F. , Ciani, M. , Di Blasio, L. , Campolunghi, M. P. , Baldessin, F. , Boldrin, L. , *et al*. (2025). Impacts of the blue crab invasion on Manila clam aquaculture in Po Delta coastal lagoons (northern Adriatic Sea, Italy). Estuarine, Coastal and Shelf Science 312, 109037.

[brv70124-bib-0141] Chinchio, E. , Romeo, C. , Crotta, M. & Ferrari, N. (2022). Knowledge gaps in invasive species infections: alien mammals of European Union concern as a case study. Science of the Total Environment 846, 157448. 35863572 10.1016/j.scitotenv.2022.157448

[brv70124-bib-0142] Clausen, C. P. (1978). Introduced Parasites and Predators of Arthropod Pests and Weedsins: A World Review. United States Department of Agriculture, Washington, DC.

[brv70124-bib-0143] Clavero, M. (2014). Shifting baselines and the conservation of non‐native species. Conservation Biology 28, 1434–1436.

[brv70124-bib-0144] Clavero, M. & García‐Berthou, E. (2005). Invasive species are a leading cause of animal extinctions. Trends in Ecology & Evolution 20, 110. 16701353 10.1016/j.tree.2005.01.003

[brv70124-bib-0145] Colautti, R. I. , Ricciardi, A. , Grigorovich, I. A. & MacIsaac, H. J. (2004). Is invasion success explained by the enemy release hypothesis? Ecology Letters 7, 721–733.

[brv70124-bib-0146] Cook, D. C. , Thomas, M. B. , Cunningham, S. A. , Anderson, D. L. & De Barro, P. J. (2007). Predicting the economic impact of an invasive species on an ecosystem service. Ecological Applications 17, 1832–1840. 17913144 10.1890/06-1632.1

[brv70124-bib-0147] Copp, G. H. (2013). The fish invasiveness screening kit (FISK) for non‐native freshwater fishes: a summary of current applications. Risk Analysis 33, 1394–1396. 23957809 10.1111/risa.12095

[brv70124-bib-0148] Copp, G. H. , Britton, J. R. , Guo, Z. , Ronni Edmonds‐Brown, V. , Pegg, J. , Vilizzi, L. & Davison, P. I. (2017). Trophic consequences of non‐native pumpkinseed *Lepomis gibbosus* for native pond fishes. Biological Invasions 19, 25–41.

[brv70124-bib-0149] Copp, G. H. , Garthwaite, R. & Gozlan, R. E. (2005 *a*). Risk identification and assessment of non‐native freshwater fishes: a summary of concepts and perspectives on protocols for the UK. Journal of Applied Ichthyology 21, 371–373.

[brv70124-bib-0150] Copp, G. H. , Garthwaite, R. & Gozlan, R. E. (2005 *b*). Risk Identification and Assessment of Non‐native Freshwater Fishes: Concepts and Perspectives on Protocols for the UK. Cefas Science Technical Report, Lowestoft.

[brv70124-bib-0151] Copp, G. H. , Russell, I. C. , Peeler, E. J. , Gherardi, F. , Tricarico, E. , Macleod, A. , Cowx, I. G. , Nunn, A. D. , Occhipinti‐Ambrogi, A. , Savini, D. , Mumford, J. & Britton, J. R. (2016 *a*). European non‐native species in aquaculture risk analysis scheme–a summary of assessment protocols and decision support tools for use of alien species in aquaculture. Fisheries Management and Ecology 23, 1–11.

[brv70124-bib-0152] Copp, G. H. , Vilizzi, L. , Tidbury, H. , Stebbing, P. D. , Tarkan, A. S. , Miossec, L. & Goulletquer, P. (2016 *b*). Development of a generic decision‐support tool for identifying potentially invasive aquatic taxa: AS‐ISK. Management of Biological Invasions 7, 343–350.

[brv70124-bib-0153] Copp, G. H. , Vilizzi, L. , Wei, H. , Li, S. , Piria, M. , Al‐Faisal, A. J. , Almeida, D. , Atique, U. , Al‐Wazzan, Z. , Bakiu, R. , Bašić, T. , Bui, T. D. , Canning‐Clode, J. , Castro, N. , Chaichana, R. , *et al*. (2021). Speaking their language–development of a multilingual decision‐support tool for communicating invasive species risks to decision makers and stakeholders. Environmental Modelling & Software 135, 104900.

[brv70124-bib-0154] Cordeiro, B. , Marchante, H. , Castro, P. & Marchante, E. (2020). Does public awareness about invasive plants pays off? An analysis of knowledge and perceptions of environmentally aware citizens in Portugal. Biological Invasions 22, 2267–2281.

[brv70124-bib-0155] Corrales, X. , Ofir, E. , Coll, M. , Goren, M. , Edelist, D. , Heymans, J. J. & Gal, G. (2017). Modeling the role and impact of alien species and fisheries on the Israeli marine continental shelf ecosystem. Journal of Marine Systems 170, 88–102.

[brv70124-bib-0156] Correia, R. A. & Mammola, S. (2024). The searchscape of fear: a global analysis of internet search trends for biophobias. People and Nature 6, 958–972.

[brv70124-bib-0157] Côté, I. M. , Darling, E. S. & Brown, C. J. (2016). Interactions among ecosystem stressors and their importance in conservation. Proceedings of the Royal Society B: Biological Sciences 283, 20152592. 10.1098/rspb.2015.2592 PMC4760168 26865306

[brv70124-bib-0158] * Coughlan, N. E. , Lyne, L. , Cuthbert, R. N. , Cunningham, E. M. , Lucy, F. E. , Davis, E. , Caffrey, J. M. & Dick, J. T. A. (2020). In the black: information harmonisation and educational potential amongst international databases for invasive alien species designated as of union concern. Global Ecology and Conservation 24, e01332.

[brv70124-bib-0159] Council of Europe (2007). Recommendation No. 125 (2007) of the Standing Committee on trade in invasive and potentially invasive alien species in Europe, adopted by the Standing Committee on 29 November 2007. Convention on the Conservation of European Wildlife and Natural Habitats (Bern Convention).

[brv70124-bib-0160] Courchamp, F. , Chapuis, J.‐L. & Pascal, M. (2003). Mammal invaders on islands: impact, control and control impact. Biological Reviews 78, 347–383. 14558589 10.1017/s1464793102006061

[brv70124-bib-0161] Courchamp, F. , Langlais, M. & Sugihara, G. (1999). Cats protecting birds: modelling the mesopredator release effect. Journal of Animal Ecology 68, 282–292.

[brv70124-bib-0162] Cowan, D. A. , Chown, S. L. , Convey, P. , Tuffin, M. , Hughes, K. , Pointing, S. & Vincent, W. F. (2011). Non‐indigenous microorganisms in the Antarctic: assessing the risks. Trends in Microbiology 19, 540–548. 21893414 10.1016/j.tim.2011.07.008

[brv70124-bib-0163] Crain, C. M. & Bertness, M. D. (2006). Ecosystem engineering across environmental gradients: implications for conservation and management. Bioscience 56, 211–218.

[brv70124-bib-0164] Cronk, Q. C. B. (1989). The past and present vegetation of St Helena. Journal of Biogeography 16, 47–64.

[brv70124-bib-0165] Crooks, J. A. (2002). Characterizing ecosystem‐level consequences of biological invasions: the role of ecosystem engineers. Oikos 97, 153–166.

[brv70124-bib-0166] Crooks, J. A. (2005). Lag times and exotic species: the ecology and management of biological invasions in slow‐motion1. Ecoscience 12, 316–329.

[brv70124-bib-0167] Crooks, J. A. , Soulé, M. E. & Sandlund, O. T. (1999). Lag times in population explosions of invasive species: causes and implications. Invasive Species and Biodiversity Management 24, 103–125.

[brv70124-bib-0168] Crosby, A. W. (2003). The Columbian Exchange: Biological and Cultural Consequences of 1492. Bloomsbury Publishing USA, Praeger, Westport, CT.

[brv70124-bib-0169] Crosby, A. W. (2004). Ecological Imperialism: The Biological Expansion of Europe, 900–1900. Cambridge University Press, Cambridge, UK.

[brv70124-bib-0170] Crowl, T. A. , Crist, T. O. , Parmenter, R. R. , Belovsky, G. & Lugo, A. E. (2008). The spread of invasive species and infectious disease as drivers of ecosystem change. Frontiers in Ecology and the Environment 6, 238–246.

[brv70124-bib-0171] * Crowley, S. L. , Hinchliffe, S. & McDonald, R. A. (2017 *a*). Conflict in invasive species management. Frontiers in Ecology and the Environment 15, 133–141.

[brv70124-bib-0172] Crowley, S. L. , Hinchliffe, S. & McDonald, R. A. (2017 *b*). Invasive species management will benefit from social impact assessment. Journal of Applied Ecology 54, 351–357.

[brv70124-bib-0173] * Crowley, S. L. , Hinchliffe, S. & McDonald, R. A. (2019). The parakeet protectors: understanding opposition to introduced species management. Journal of Environmental Management 229, 120–132. 29305043 10.1016/j.jenvman.2017.11.036

[brv70124-bib-0174] Crystal‐Ornelas, R. & Lockwood, J. L. (2020). The ‘known unknowns’ of invasive species impact measurement. Biological Invasions 22, 1513–1525.

[brv70124-bib-0175] Cucherousset, J. , Horky, P. , Slavík, O. , Ovidio, M. , Arlinghaus, R. , Boulêtreau, S. , Britton, R. , García‐Berthou, E. & Santoul, F. (2018). Ecology, behaviour and management of the European catfish. Reviews in Fish Biology and Fisheries 28, 177–190.

[brv70124-bib-0176] Cucherousset, J. & Olden, J. D. (2011). Ecological impacts of non‐native freshwater fishes. Fisheries 36, 215–230.

[brv70124-bib-0177] Čuda, J. , Skálová, H. , Janovský, Z. & Pyšek, P. (2015). Competition among native and invasive impatiens species: the roles of environmental factors, population density and life stage. AoB Plants 7, plv033. 25832103 10.1093/aobpla/plv033 PMC4417208

[brv70124-bib-0178] Currat, M. , Ruedi, M. , Petit, R. J. & Excoffier, L. (2008). The hidden side of invasions: massive introgression by local genes. Evolution 62, 1908–1920. 18452573 10.1111/j.1558-5646.2008.00413.x

[brv70124-bib-0179] Curtis, J. S. , Wall, K. R. , Albins, M. A. & Stallings, C. D. (2017). Diet shifts in a native mesopredator across a range of invasive lionfish biomass. Marine Ecology Progress Series 573, 215–228.

[brv70124-bib-0180] * Cuthbert, R. N. , Diagne, C. , Haubrock, P. J. , Turbelin, A. J. & Courchamp, F. (2022). Are the “100 of the world's worst” invasive species also the costliest? Biological Invasions 24, 1895–1904.

[brv70124-bib-0181] Cuthbert, R. N. , Pattison, Z. , Taylor, N. G. , Verbrugge, L. , Diagne, C. , Ahmed, D. A. , Leroy, B. , Angulo, E. , Briski, E. , Capinha, C. , Catford, J. A. , Dalu, T. , Essl, F. , Gozlan, R. E. , Haubrock, P. J. , *et al*. (2021). Global economic costs of aquatic invasive alien species. Science of the Total Environment 775, 145238. 33715860 10.1016/j.scitotenv.2021.145238

[brv70124-bib-0182] Daly, M. , Behrends, P. R. , Wilson, M. I. & Jacobs, L. F. (1992). Behavioural modulation of predation risk: moonlight avoidance and crepuscular compensation in a nocturnal desert rodent. Dipodomys merriami. Animal Behaviour 44, 1–9.

[brv70124-bib-0183] Damas‐Moreira, I. , Riley, J. L. , Carretero, M. A. , Harris, D. J. & Whiting, M. J. (2020). Getting ahead: exploitative competition by an invasive lizard. Behavioral Ecology and Sociobiology 74, 117.

[brv70124-bib-0184] D'Amore, D. M. , Popescu, V. D. & Morris, M. R. (2019). The influence of the invasive process on behaviours in an intentionally introduced hybrid, *Xiphophorus helleri–maculatus* . Animal Behaviour 156, 79–85.

[brv70124-bib-0185] Dangremond, E. M. , Pardini, E. A. & Knight, T. M. (2010). Apparent competition with an invasive plant hastens the extinction of an endangered lupine. Ecology 91, 2261–2271. 20836448 10.1890/09-0418.1

[brv70124-bib-0186] D'Antonio, C. M. (1993). Mechanisms controlling invasion of coastal plant communities by the alien succulent *Carpobrotus edulis* . Ecology 74, 83–95.

[brv70124-bib-0187] D'Antonio, C. M. & Mahall, B. E. (1991). Root profiles and competition between the invasive, exotic perennial, *Carpobrotus edulis*, and two native shrub species in California coastal scrub. American Journal of Botany 78, 885–894.

[brv70124-bib-0188] D'Antonio, C. M. & Vitousek, P. M. (1992). Biological invasions by exotic grasses, the grass/fire cycle, and global change. Annual Review of Ecology and Systematics 23, 63–87.

[brv70124-bib-0189] Darwin, C. (1889). Journal of Researches into the Natural History and Geology of the Countries Visited during the Voyage of HMS“ Beagle” Round the World: Under the Command of Capt. J. Murray, Fitz Roy, RN.

[brv70124-bib-0190] Davidson, A. D. & Hewitt, C. L. (2014). How often are invasion‐induced ecological impacts missed? Biological Invasions 16, 1165–1173.

[brv70124-bib-0191] Davis, M. A. (2011). Invasion biology. In Encyclopedia of Biological Invasions (eds D. Simberloff and M. Rejmánek ), pp. 364–369. University of California Press, Berkeley, CA.

[brv70124-bib-0192] Davis, M. A. , Chew, M. K. , Hobbs, R. J. , Lugo, A. E. , Ewel, J. J. , Vermeij, G. J. , Brown, J. H. , Rosenzweig, M. L. , Gardener, M. R. , Carroll, S. P. , Thompson, K. , Pickett, S. T. A. , Stromberg, J. C. , Del Tredici, P. , Suding, K. N. , *et al*. (2011). Don't judge species on their origins. Nature 474, 153–154. 21654782 10.1038/474153a

[brv70124-bib-0193] De Camargo, M. P. , Cunico, A. M. & Gomes, L. C. (2022). Biological invasions in neotropical regions: continental ichthyofauna and risk assessment protocols. Environmental Management 70, 307–318. 35695897 10.1007/s00267-022-01671-2

[brv70124-bib-0194] de Carvalho‐Souza, G. F. , Kourantidou, M. , Laiz, I. , Nuñez, M. A. & González‐Ortegón, E. (2024). How to deal with invasive species that have high economic value? Biological Conservation 292, 110548.

[brv70124-bib-0195] * De Groot, M. , O'Hanlon, R. , Bullas‐Appleton, E. , Csóka, G. , Csiszár, Á. , Faccoli, M. , Gervasini, E. , Kirichenko, N. , Korda, M. & Marinšek, A. (2020). Challenges and solutions in early detection, rapid response and communication about potential invasive alien species in forests. Management of Biological Invasions 11, 637–660.

[brv70124-bib-0196] De Groot, R. S. , Wilson, M. A. & Boumans, R. M. J. (2002). A typology for the classification, description and valuation of ecosystem functions, goods and services. Ecological Economics 41, 393–408.

[brv70124-bib-0197] de Miguel, J. M. , Martín‐Forés, I. , Acosta‐Gallo, B. , del Pozo, A. , Ovalle, C. , Sánchez‐Jardón, L. , Castro, I. & Casado, M. A. (2016). Non‐random co‐occurrence of native and exotic plant species in Mediterranean grasslands. Acta Oecologica 77, 18–26.

[brv70124-bib-0198] Deak, B. P. , Ostendorf, B. , Taggart, D. A. , Peacock, D. E. & Bardsley, D. K. (2019). The significance of social perceptions in implementing successful feral cat management strategies: a global review. Animals 9, 617. 31466221 10.3390/ani9090617 PMC6770462

[brv70124-bib-0199] Déchamp, C. (1999). Ragweed, a biological pollutant: current and desirable legal implications in France and Europe. Revue Française d'Allergologie et d'Immunologie Clinique 39, 289–294.

[brv70124-bib-0200] Delibes‐Mateos, M. , Farfán, M. Á. , Rouco, C. , Olivero, J. , Márquez, A. L. , Fa, J. E. , Vargas, J. M. & Villafuerte, R. (2018). A large‐scale assessment of European rabbit damage to agriculture in Spain. Pest Management Science 74, 111–119. 28722344 10.1002/ps.4658

[brv70124-bib-0201] Dercksen, J. A. , Schrama, M. J. J. , Beentjes, K. K. , Bastiaans, B. N. , Blom, R. , van Roon, A. , Lindenburg, P. W. & Trimbos, K. B. (2025). Invasive crayfish: drivers or passengers of degradation in freshwater ecosystems? Environmental DNA 7, e70062.

[brv70124-bib-0202] D'hondt, B. , Vanderhoeven, S. , Adriaens, T. & Branquart, E. (2025). A demarcation of risk categories for invasion risk assessments using the Harmonia+ protocol. Biological Invasions 17, 1869–1883.

[brv70124-bib-0203] D'hondt, B. , Vanderhoeven, S. , Roelandt, S. , Mayer, F. , Versteirt, V. , Adriaens, T. , Ducheyne, E. , San Martin, G. , Grégoire, J.‐C. , Stiers, I. , Quoilin, S. , Cigar, J. , Heughebaert, A. & Branquart, E. (2015). Harmonia+ and Pandora+: risk screening tools for potentially invasive plants, animals and their pathogens. Biological Invasions 17, 1869–1883.

[brv70124-bib-0204] Diagne, C. , Leroy, B. , Gozlan, R. E. , Vaissière, A.‐C. , Assailly, C. , Nuninger, L. , Roiz, D. , Jourdain, F. , Jarić, I. & Courchamp, F. (2020). InvaCost, a public database of the economic costs of biological invasions worldwide. Scientific Data 7, 277. 32901023 10.1038/s41597-020-00586-z PMC7479195

[brv70124-bib-0205] Diagne, C. , Leroy, B. , Vaissière, A.‐C. , Gozlan, R. E. , Roiz, D. , Jarić, I. , Salles, J.‐M. , Bradshaw, C. J. A. & Courchamp, F. (2021). High and rising economic costs of biological invasions worldwide. Nature 592, 571–576. 33790468 10.1038/s41586-021-03405-6

[brv70124-bib-0206] Dick, J. T. A. , Alexander, M. E. , Jeschke, J. M. , Ricciardi, A. , MacIsaac, H. J. , Robinson, T. B. , Kumschick, S. , Weyl, O. L. F. , Dunn, A. M. , Hatcher, M. J. , Paterson, R. A. , Farnsworth, K. D. & Richardson, D. M. (2014). Advancing impact prediction and hypothesis testing in invasion ecology using a comparative functional response approach. Biological Invasions 16, 735–753.

[brv70124-bib-0207] Dick, J. T. A. , Laverty, C. , Lennon, J. J. , Barrios‐O'Neill, D. , Mensink, P. J. , Robert Britton, J. , Médoc, V. , Boets, P. , Alexander, M. E. , Taylor, N. G. , Dunn, A. M. , Hatcher, M. J. , Rosewarne, P. J. , Crookes, S. , MacIsaac, H. J. , *et al*. (2017). Invader relative impact potential: a new metric to understand and predict the ecological impacts of existing, emerging and future invasive alien species. Journal of Applied Ecology 54, 1259–1267.

[brv70124-bib-0208] Dick, J. T. A. & Platvoet, D. (2000). Invading predatory crustacean *Dikerogammarus villosus* eliminates both native and exotic species. Proceedings of the Royal Society of London. Series B: Biological Sciences 267, 977–983. 10.1098/rspb.2000.1099 PMC1690628 10874746

[brv70124-bib-0209] Dickey, J. W. E. , Arnott, G. , McGlade, C. L. O. , Moore, A. , Riddell, G. E. & Dick, J. T. A. (2022). Threats at home? Assessing the potential ecological impacts and risks of commonly traded pet fishes. NeoBiota 73, 109–136.

[brv70124-bib-0210] Dickey, J. W. E. , Coughlan, N. E. , Dick, J. T. A. , Médoc, V. , McCard, M. , Leavitt, P. R. , Lacroix, G. , Fiorini, S. , Millot, A. & Cuthbert, R. N. (2021). Breathing space: deoxygenation of aquatic environments can drive differential ecological impacts across biological invasion stages. Biological Invasions 23, 2831–2847. 34720687 10.1007/s10530-021-02542-3 PMC8550720

[brv70124-bib-0211] Dickey, J. W. E. , Cuthbert, R. N. , South, J. , Britton, J. R. , Caffrey, J. , Chang, X. , Crane, K. , Coughlan, N. E. , Fadaei, E. , Farnsworth, K. D. , Ismar‐Rebitz, S. M. H. , Julius, M. , Laverty, C. , Lucy, F. E. , MacIsaac, H. J. , *et al*. (2020). On the RIP: using relative impact potential to assess the ecological impacts of invasive alien species. NeoBiota 55, 27–60.

[brv70124-bib-0212] Dickie, I. A. , Bennett, B. M. , Burrows, L. E. , Nuñez, M. A. , Peltzer, D. A. , Porté, A. , Richardson, D. M. , Rejmánek, M. , Rundel, P. W. & Van Wilgen, B. W. (2014). Conflicting values: ecosystem services and invasive tree management. Biological Invasions 16, 705–719.

[brv70124-bib-0213] Didham, R. K. , Tylianakis, J. M. , Hutchison, M. A. , Ewers, R. M. & Gemmell, N. J. (2005). Are invasive species the drivers of ecological change? Trends in Ecology & Evolution 20, 470–474. 16701420 10.1016/j.tree.2005.07.006

[brv70124-bib-0214] * Dietterich, T. , Taleghan, M. A. & Crowley, M. (2013). PAC optimal planning for invasive species management: improved exploration for reinforcement learning from simulator‐defined MDPs. In Proceedings of the AAAI Conference on Artificial Intelligence, pp. 1270–1276. AAAI Press, Palo Alto, CA.

[brv70124-bib-0215] * Di Febbraro, M. , Lurz, P. W. W. , Genovesi, P. , Maiorano, L. , Girardello, M. & Bertolino, S. (2013). The use of climatic niches in screening procedures for introduced species to evaluate risk of spread: a case with the American eastern grey squirrel. PLoS One 8, e66559. 23843957 10.1371/journal.pone.0066559 PMC3701016

[brv70124-bib-0216] Docherty, C. H. (2016). Establishment, spread and impact of Prussian carp (*Carassius gibelio*). In A New Invasive Species in Western North America. Master of Science (Thesis), University of Alberta, Edmonton, AB, Canada.

[brv70124-bib-0217] Dodd, J. A. , Copp, G. H. , Tidbury, H. J. , Leuven, R. S. E. W. , Feunteun, E. , Olsson, K. H. , Gollasch, S. , Jelmert, A. , O'Shaughnessy, K. A. , Reeves, D. , Brenner, J. & Verreycken, H. (2022). Invasiveness risks of naked goby, Gobiosoma bosc, to North Sea transitional waters. Marine Pollution Bulletin 181, 113763. 35752508 10.1016/j.marpolbul.2022.113763

[brv70124-bib-0218] Doherty, T. S. , Glen, A. S. , Nimmo, D. G. , Ritchie, E. G. & Dickman, C. R. (2016). Invasive predators and global biodiversity loss. Proceedings of the National Academy of Sciences 113, 11261–11265. 10.1073/pnas.1602480113 PMC5056110 27638204

[brv70124-bib-0219] Doherty‐Bone, T. M. , Dunn, A. M. , Jackson, F. L. & Brown, L. E. (2019). Multi‐faceted impacts of native and invasive alien decapod species on freshwater biodiversity and ecosystem functioning. Freshwater Biology 64, 461–473.

[brv70124-bib-0220] Dolan, E. J. , Soto, I. , Dick, J. T. A. , He, F. & Cuthbert, R. N. (2025). Riverine barrier removals could proliferate biological invasions. Global Change Biology 31, e70093. 40041937 10.1111/gcb.70093

[brv70124-bib-0221] Dominguez Almela, V. , Addo, K. A. , Corbett, J. , Cumberbatch, J. , Dash, J. , Marsh, R. , Oxenford, H. , Tonon, T. , Van Der Plank, S. , Webber, M. & Tompkins, E. L. (2023 *a*). Science and policy lessons learned from a decade of adaptation to the emergent risk of sargassum proliferation across the tropical Atlantic. Environmental Research Communications 5, 061002.

[brv70124-bib-0222] Dominguez Almela, V. , South, J. & Britton, J. R. (2021). Predicting the competitive interactions and trophic niche consequences of a globally invasive fish with threatened native species. Journal of Animal Ecology 90, 2651–2662. 34309851 10.1111/1365-2656.13571

[brv70124-bib-0223] Dominguez Almela, V. , Tompkins, E. L. , Dash, J. & Tonon, T. (2023 *b*). Brown algae invasions and bloom events need routine monitoring for effective adaptation. Environmental Research Letters 19, 013003.

[brv70124-bib-0224] Donovan, G. H. , Butry, D. T. , Michael, Y. L. , Prestemon, J. P. , Liebhold, A. M. , Gatziolis, D. & Mao, M. Y. (2013). The relationship between trees and human health: evidence from the spread of the emerald ash borer. American Journal of Preventive Medicine 44, 139–145. 23332329 10.1016/j.amepre.2012.09.066

[brv70124-bib-0225] Dorcas, M. E. , Willson, J. D. , Reed, R. N. , Snow, R. W. , Rochford, M. R. , Miller, M. A. , Meshaka, W. E. Jr. , Andreadis, P. T. , Mazzotti, F. J. , Romagosa, C. M. & Hart, K. M. (2012). Severe mammal declines coincide with proliferation of invasive Burmese pythons in Everglades National Park. Proceedings of the National Academy of Sciences 109, 2418–2422. 10.1073/pnas.1115226109 PMC3289325 22308381

[brv70124-bib-0226] Doren, R. F. & Whiteaker, L. D. (1990). Effects of fire on different size individuals of *Schinus terebinthifolius* . Natural Areas Journal 10, 107–113.

[brv70124-bib-0227] Doubledee, R. A. , Muller, E. B. & Nisbet, R. M. (2003). Bullfrogs, disturbance regimes, and the persistence of California red‐legged frogs. The Journal of Wildlife Management 67, 424–438.

[brv70124-bib-0228] Drolet, D. , DiBacco, C. , Locke, A. , McKenzie, C. H. , McKindsey, C. W. , Moore, A. M. , Webb, J. L. & Therriault, T. W. (2016). Evaluation of a new screening‐level risk assessment tool applied to non‐indigenous marine invertebrates in Canadian coastal waters. Biological Invasions 18, 279–294.

[brv70124-bib-0229] Dueñas, M.‐A. , Hemming, D. J. , Roberts, A. & Diaz‐Soltero, H. (2021). The threat of invasive species to IUCN‐listed critically endangered species: a systematic review. Global Ecology and Conservation 26, e01476.

[brv70124-bib-0230] Dunn, A. M. , Torchin, M. E. , Hatcher, M. J. , Kotanen, P. M. , Blumenthal, D. M. , Byers, J. E. , Coon, C. A. C. , Frankel, V. M. , Holt, R. D. , Hufbauer, R. A. , Kanarek, A. R. , Schierenbeck, K. A. , Wolfe, L. M. & Perkins, S. E. (2012). Indirect effects of parasites in invasions. Functional Ecology 26, 1262–1274.

[brv70124-bib-0231] Dunn, M. , Marzano, M. , Forster, J. & Gill, R. M. A. (2018). Public attitudes towards “pest” management: perceptions on squirrel management strategies in the UK. Biological Conservation 222, 52–63.

[brv70124-bib-0232] Durant, S. & Faunce, T. A. (2018). Analysis of Australia's new biosecurity legislation. Durant S and Faunce TA Analysis of Australia's New Biosecurity Legislation 2018(25), 647–654. 29978659

[brv70124-bib-0233] Dybzinski, R. & Tilman, D. (2007). Resource use patterns predict long‐term outcomes of plant competition for nutrients and light. The American Naturalist 170, 305–318. 10.1086/519857 17879183

[brv70124-bib-0234] Early, R. , Bradley, B. A. , Dukes, J. S. , Lawler, J. J. , Olden, J. D. , Blumenthal, D. M. , Gonzalez, P. , Grosholz, E. D. , Ibañez, I. , Miller, L. P. , Sorte, C. J. B. & Tatem, A. J. (2016). Global threats from invasive alien species in the twenty‐first century and national response capacities. Nature Communications 7, 12485. 10.1038/ncomms12485 PMC4996970 27549569

[brv70124-bib-0235] Ehrenfeld, J. G. (2010). Ecosystem consequences of biological invasions. Annual Review of Ecology, Evolution, and Systematics 41, 59–80.

[brv70124-bib-0236] Ellington, E. H. & Murray, D. L. (2015). Influence of hybridization on animal space use: a case study using coyote range expansion. Oikos 124, 535–542.

[brv70124-bib-0237] Ellison, A. M. , Orwig, D. A. , Fitzpatrick, M. C. & Preisser, E. L. (2018). The past, present, and future of the hemlock woolly adelgid (*Adelges tsugae*) and its ecological interactions with eastern hemlock (*Tsuga canadensis*) forests. Insects 9, 172. 30477155 10.3390/insects9040172 PMC6316461

[brv70124-bib-0238] Emery, N. C. , Ewanchuk, P. J. & Bertness, M. D. (2001). Competition and salt‐marsh plant zonation: stress tolerators may be dominant competitors. Ecology 82, 2471–2485.

[brv70124-bib-0239] Emery‐Butcher, H. E. , Beatty, S. J. & Robson, B. J. (2020). The impacts of invasive ecosystem engineers in freshwaters: a review. Freshwater Biology 65, 999–1015.

[brv70124-bib-0240] Eschen, R. , Beale, T. , Bonnin, J. M. , Constantine, K. L. , Duah, S. , Finch, E. A. , Makale, F. , Nunda, W. , Ogunmodede, A. , Pratt, C. F. , Thompson, E. , Williams, F. , Witt, A. & Taylor, B. (2021). Towards estimating the economic cost of invasive alien species to African crop and livestock production. CABI Agriculture and Bioscience 2, 1–18.

[brv70124-bib-0241] Essian, D. A. , Chipault, J. G. , Lafrancois, B. M. & Leonard, J. B. K. (2016). Gut content analysis of Lake Michigan waterbirds in years with avian botulism type E mortality, 2010–2012. Journal of Great Lakes Research 42, 1118–1128.

[brv70124-bib-0242] Essl, F. , Dullinger, S. , Rabitsch, W. , Hulme, P. E. , Hülber, K. , Jarošík, V. , Kleinbauer, I. , Krausmann, F. , Kühn, I. , Nentwig, W. , Vilà, M. , Genovesi, P. , Gherardi, F. , Desprez‐Loustau, M.‐L. , Roques, A. , *et al*. (2011 *a*). Socioeconomic legacy yields an invasion debt. Proceedings of the National Academy of Sciences 108, 203–207. 10.1073/pnas.1011728108 PMC3017203 21173227

[brv70124-bib-0243] Essl, F. , Lenzner, B. , Bacher, S. , Bailey, S. , Capinha, C. , Daehler, C. , Dullinger, S. , Genovesi, P. , Hui, C. , Hulme, P. E. , Jeschke, J. M. , Katsanevakis, S. , Kühn, I. , Leung, B. , Liebhold, A. , *et al*. (2020). Drivers of future alien species impacts: an expert‐based assessment. Global Change Biology 26, 4880–4893. 32663906 10.1111/gcb.15199 PMC7496498

[brv70124-bib-0244] Essl, F. , Nehring, S. , Klingenstein, F. , Milasowszky, N. , Nowack, C. & Rabitsch, W. (2011 *b*). Review of risk assessment systems of IAS in Europe and introducing the German–Austrian Black list information system (GABLIS). Journal for Nature Conservation 19, 339–350.

[brv70124-bib-0245] Estes, J. A. , Terborgh, J. , Brashares, J. S. , Power, M. E. , Berger, J. , Bond, W. J. , Carpenter, S. R. , Essington, T. E. , Holt, R. D. , Jackson, J. B. C. , Marquis, R. J. , Oksanen, L. , Oksanen, T. , Paine, R. T. , Pikitch, E. K. , *et al*. (2011). Trophic downgrading of planet earth. Science 333, 301–306. 21764740 10.1126/science.1205106

[brv70124-bib-0246] * Estévez, R. A. , Anderson, C. B. , Pizarro, J. C. & Burgman, M. A. (2015). Clarifying values, risk perceptions, and attitudes to resolve or avoid social conflicts in invasive species management. Conservation Biology 29, 19–30. 25155068 10.1111/cobi.12359

[brv70124-bib-0247] European Commission (2014). Regulation (EU) No 1143/2014 of the European Parliament and of the Council of 22 October 2014 on the prevention and management of the introduction and spread of invasive alien species.

[brv70124-bib-0248] European Commission (2022 *a*). Commission Implementing Regulation (EU) 2022/1203 of 12 July 2022 amending Implementing Regulation (EU) 2016/1141 to update the list of invasive alien species of Union concern.

[brv70124-bib-0249] European Commission (2022 *b*). Directorate‐General for Environment and Umweltbundesamt GmbH, Study on invasive alien species – Development of risk assessments to tackle priority species and enhance prevention – Final report (and annexes).

[brv70124-bib-0250] Evans, T. , Angulo, E. , Bradshaw, C. J. A. , Turbelin, A. & Courchamp, F. (2023). Global economic costs of alien birds. PLoS One 18, e0292854. 37851652 10.1371/journal.pone.0292854 PMC10584179

[brv70124-bib-0251] Evans, T. , Kumschick, S. & Blackburn, T. M. (2016). Application of the environmental impact classification for alien taxa (EICAT) to a global assessment of alien bird impacts. Diversity and Distributions 22, 919–931.

[brv70124-bib-0252] Everts, T. , Van Driessche, C. , Neyrinck, S. , Haegeman, A. , Ruttink, T. , Jacquemyn, H. & Brys, R. (2024). Phenological mismatches mitigate the ecological impact of a biological invader on amphibian communities. Ecological Applications 34, e3017. 39118362 10.1002/eap.3017

[brv70124-bib-0253] Fahnenstiel, G. L. , Bridgeman, T. B. , Lang, G. A. , McCormick, M. J. & Nalepa, T. F. (1995). Phytoplankton productivity in Saginaw Bay, Lake Huron: effects of zebra mussel (*Dreissena polymorpha*) colonization. Journal of Great Lakes Research 21, 464–475.

[brv70124-bib-0254] Faria, L. , Cuthbert, R. N. , Dickey, J. W. E. , Jeschke, J. M. , Ricciardi, A. , Dick, J. T. A. & Vitule, J. R. S. (2023). The rise of the functional response in invasion science: a systematic review. NeoBiota 85, 43–79.

[brv70124-bib-0255] Faria, L. , Cuthbert, R. N. , Dickey, J. W. E. , Jeschke, J. M. , Ricciardi, A. , Dick, J. T. A. & Vitule, J. R. S. (2025). Non‐native species have higher consumption rates than their native counterparts. Biological Reviews 100, 1163–1180. 39807655 10.1111/brv.13179

[brv70124-bib-0256] Farnsworth, D. , Hamby, K. A. , Bolda, M. , Goodhue, R. E. , Williams, J. C. & Zalom, F. G. (2017). Economic analysis of revenue losses and control costs associated with the spotted wing drosophila, *Drosophila suzukii* (Matsumura), in the California raspberry industry. Pest Management Science 73, 1083–1090. 27943618 10.1002/ps.4497

[brv70124-bib-0257] * Fenollosa, E. & Salguero‐Gomez, R. (2025). AI and big data for invasion biology: finding, modelling and forecasting the population dynamics of invaders. EcoEvoRxiv. 10.32942/X2Q62Z.

[brv70124-bib-0258] Fernandez, R. D. , Haubrock, P. J. , Cuthbert, R. N. , Heringer, G. , Kourantidou, M. , Hudgins, E. J. , Angulo, E. , Diagne, C. A. , Courchamp, F. & Nuñez, M. A. (2023). Underexplored and growing economic costs of invasive alien trees. Scientific Reports 13, 8945. 37268662 10.1038/s41598-023-35802-4 PMC10238485

[brv70124-bib-0259] Ferreira‐Rodríguez, N. , Sousa, R. & Pardo, I. (2018). Negative effects of *Corbicula fluminea* over native freshwater mussels. Hydrobiologia 810, 85–95.

[brv70124-bib-0260] Ficetola, G. F. , Melotto, A. , Scali, S. , Sacchi, R. & Salvi, D. (2024). Interference competition with an invasive species as potential driver of rapid extinction in an Island‐endemic lizard. Global Ecology and Conservation 55, e03251.

[brv70124-bib-0261] Florencio, M. , Lobo, J. M. & Bini, L. M. (2019). Biases in global effects of exotic species on local invertebrates: a systematic review. Biological Invasions 21, 3043–3061.

[brv70124-bib-0262] Ford, C. R. & Vose, J. M. (2007). *Tsuga canadensis* (L.) Carr. mortality will impact hydrologic processes in southern Appalachian forest ecosystems. Ecological Applications 17, 1156–1167. 17555225 10.1890/06-0027

[brv70124-bib-0263] Fournier, D. & Aron, S. (2021). Hybridization and invasiveness in social insects—the good, the bad and the hybrid. Current Opinion in Insect Science 46, 1–9. 33484875 10.1016/j.cois.2020.12.004

[brv70124-bib-0264] Franco, A. C. S. , Petry, A. C. , García‐Berthou, E. & dos Santos, L. N. (2022). Invasive peacock basses (*Cichla* spp.) and decreased abundance of small native fish in Brazilian reservoirs. Aquatic Conservation: Marine and Freshwater Ecosystems 32, 1852–1866.

[brv70124-bib-0265] Frem, M. , Nardelli, L. , Petrontino, A. , Navrud, S. , Colonna, M. A. , Fucilli, V. & Bozzo, F. (2024). Public preferences for edible invasive alien marine species‐the Atlantic blue crab in southern Italy. NeoBiota 96, 19–47.

[brv70124-bib-0266] Fricke, R. M. & Olden, J. D. (2023). Technological innovations enhance invasive species management in the Anthropocene. Bioscience 73, 261–279.

[brv70124-bib-0267] Fritts, T. H. & Rodda, G. H. (1998). The role of introduced species in the degradation of Island ecosystems: a case history of Guam. Annual Review of Ecology and Systematics 29, 113–140.

[brv70124-bib-0268] Gaertner, M. , Biggs, R. , Te Beest, M. , Hui, C. , Molofsky, J. & Richardson, D. M. (2014). Invasive plants as drivers of regime shifts: identifying high‐priority invaders that alter feedback relationships. Diversity and Distributions 20, 733–744.

[brv70124-bib-0269] Gaertner, M. , Wilson, J. R. , Cadotte, M. W. , MacIvor, J. S. , Zenni, R. D. & Richardson, D. M. (2017). Non‐native species in urban environments: patterns, processes, impacts and challenges. Biological Invasions 19, 3461–3469.

[brv70124-bib-0270] Galanidi, M. , Zenetos, A. & Bacher, S. (2018). Assessing the socio‐economic impacts of priority marine invasive fishes in the Mediterranean with the newly proposed SEICAT methodology. Mediterranean Marine Science 19, 107–123.

[brv70124-bib-0271] Galil, B. (2018). Poisonous and venomous: marine alien species in the Mediterranean Sea and human health. In Invasive Species and Human Health, pp. 1–15. CAB International Wallingford, Oxfordshire, UK.

[brv70124-bib-0272] Gallardo, B. , Bacher, S. , Barbosa, A. M. , Gallien, L. , González‐Moreno, P. , Martínez‐Bolea, V. , Sorte, C. , Vimercati, G. & Vilà, M. (2024). Risks posed by invasive species to the provision of ecosystem services in Europe. Nature Communications 15, 2631. 10.1038/s41467-024-46818-3 PMC11006939 38600085

[brv70124-bib-0273] Gallardo, B. , Clavero, M. , Sánchez, M. I. & Vilà, M. (2016). Global ecological impacts of invasive species in aquatic ecosystems. Global Change Biology 22, 151–163. 26212892 10.1111/gcb.13004

[brv70124-bib-0274] Garcia, R. A. & Clusella‐Trullas, S. (2025). Microclimatic changes caused by plant invasions and warming: uncovering thermal costs and benefits to a tortoise. *Conservation* . Physiology 13, coaf016. 10.1093/conphys/coaf016 PMC11884760 40051553

[brv70124-bib-0275] García‐Berthou, E. , Alcaraz, C. , Pou‐Rovira, Q. , Zamora, L. , Coenders, G. & Feo, C. (2005). Introduction pathways and establishment rates of invasive aquatic species in Europe. Canadian Journal of Fisheries and Aquatic Sciences 62, 453–463.

[brv70124-bib-0276] García‐Gómez, J. C. , Sempere‐Valverde, J. , González, A. R. , Martínez‐Chacón, M. , Olaya‐Ponzone, L. , Sánchez‐Moyano, E. , Ostalé‐Valriberas, E. & Megina, C. (2020). From exotic to invasive in record time: the extreme impact of *Rugulopteryx okamurae* (Dictyotales, Ochrophyta) in the strait of Gibraltar. Science of the Total Environment 704, 135408. 31836226 10.1016/j.scitotenv.2019.135408

[brv70124-bib-0277] * García‐Díaz, P. , Cassey, P. , Norbury, G. , Lambin, X. , Montti, L. , Pizarro, J. C. , Powell, P. A. , Burslem, D. F. R. P. , Cava, M. , Damasceno, G. , Fasola, L. , Fidelis, A. , Huerta, M. F. , Langdon, B. , Linardaki, E. , *et al*. (2021). Management policies for invasive alien species: addressing the impacts rather than the species. Bioscience 71, 174–185.

[brv70124-bib-0278] * Garcia‐Lozano, C. , Pueyo‐Ros, J. , Canelles, Q. , Latombe, G. , Adriaens, T. , Bacher, S. , Cardoso, A. C. , Cleary, M. , Coromina, L. & Courchamp, F. (2025). Management measures and trends of biological invasions in Europe: a survey‐based assessment of local managers. Global Change Biology 31, e70028. 39825587 10.1111/gcb.70028 PMC11742469

[brv70124-bib-0279] Garrabou, J. , Gómez‐Gras, D. , Medrano, A. , Cerrano, C. , Ponti, M. , Schlegel, R. , Bensoussan, N. , Turicchia, E. , Sini, M. , Gerovasileiou, V. , Teixido, N. , Mirasole, A. , Tamburello, L. , Cebrian, E. , Rilov, G. , *et al*. (2022). Marine heatwaves drive recurrent mass mortalities in the Mediterranean Sea. Global Change Biology 28, 5708–5725. 35848527 10.1111/gcb.16301 PMC9543131

[brv70124-bib-0280] Gavioli, A. , Mancinelli, G. , Turolla, E. , Lanzoni, M. , Paesanti, V. , Soana, E. , Eggleston, D. B. , Christian, R. R. & Castaldelli, G. (2025). Impacts of the invasive blue crab *Callinectes sapidus* on small‐scale fisheries in a Mediterranean lagoon using fishery landing data. Science of the Total Environment 974, 179236. 40154084 10.1016/j.scitotenv.2025.179236

[brv70124-bib-0281] Gaynor, K. M. , Hojnowski, C. E. , Carter, N. H. & Brashares, J. S. (2018). The influence of human disturbance on wildlife nocturnality. Science 360, 1232–1235. 29903973 10.1126/science.aar7121

[brv70124-bib-0282] Ge, Z. , Guo, H. , Zhao, B. & Zhang, L. (2015). Plant invasion impacts on the gross and net primary production of the salt marsh on eastern coast of China: insights from leaf to ecosystem. Journal of Geophysical Research: Biogeosciences 120, 169–186.

[brv70124-bib-0283] Gederaas, L. , Loennechen Moen, T. , Skjelseth, S. & Larsen, L. K. (2012). Alien Species in Norway‐With the Norwegian Black List 2012. The Norwegian Biodiversity Information Centre, Norway.

[brv70124-bib-0284] Genovesi, P. , Carboneras, C. , Vila, M. & Walton, P. (2015). EU adopts innovative legislation on invasive species: a step towards a global response to biological invasions? Biological Invasions 17, 1307–1311.

[brv70124-bib-0285] Genovesi, P. & Scalera, R. (2007). Towards a black list of invasive alien species entering Europe through trade, and proposed responses. In Convention on the Conservation of European Wildlife and Natural Habitats. Standing Committee 27th meeting, Strasbourg, 26–29 November 2007. Council of Europe, Strasbourg, France (T‐PVS/Inf (2007) 9).

[brv70124-bib-0286] Gergs, R. , Rinke, K. & Rothhaupt, K. (2009). Zebra mussels mediate benthic–pelagic coupling by biodeposition and changing detrital stoichiometry. Freshwater Biology 54, 1379–1391.

[brv70124-bib-0287] Gergs, R. & Rothhaupt, K.‐O. (2008). Effects of zebra mussels on a native amphipod and the invasive *Dikerogammarus villosus*: the influence of biodeposition and structural complexity. Journal of the North American Benthological Society 27, 541–548.

[brv70124-bib-0288] Gherardi, F. (2006). Crayfish invading Europe: the case study of *Procambarus clarkii* . Marine and Freshwater Behaviour and Physiology 39, 175–191.

[brv70124-bib-0289] Gilovich, T. , Griffin, D. & Kahneman, D. (2002). Heuristics and Biases: The Psychology of Intuitive Judgment. Cambridge University Press, Cambridge.

[brv70124-bib-0290] Gissi, E. , Manea, E. , Mazaris, A. D. , Fraschetti, S. , Almpanidou, V. , Bevilacqua, S. , Coll, M. , Guarnieri, G. , Lloret‐Lloret, E. , Pascual, M. , Petza, D. , Rilov, G. , Schonwald, M. , Stelzenmüller, V. & Katsanevakis, S. (2021). A review of the combined effects of climate change and other local human stressors on the marine environment. Science of the Total Environment 755, 142564. 33035971 10.1016/j.scitotenv.2020.142564

[brv70124-bib-0291] Givan, O. , Edelist, D. , Sonin, O. & Belmaker, J. (2018). Thermal affinity as the dominant factor changing Mediterranean fish abundances. Global Change Biology 24, e80–e89. 28727210 10.1111/gcb.13835

[brv70124-bib-0292] Goka, K. (2010). Biosecurity measures to prevent the incursion of invasive alien species into Japan and to mitigate their impact. Revue Scientifique et Technique 29, 299. 20919584 10.20506/rst.29.2.1982

[brv70124-bib-0293] González‐Moreno, P. , Lazzaro, L. , Vilà, M. , Preda, C. , Adriaens, T. , Bacher, S. , Brundu, G. , Copp, G. H. , Essl, F. , García‐Berthou, E. , Katsanevakis, S. , Moen, T. L. , Lucy, F. E. , Nentwig, W. , Roy, H. E. , *et al*. (2019). Consistency of impact assessment protocols for non‐native species. NeoBiota 44, 1–25.

[brv70124-bib-0294] Gordon, D. R. , Mitterdorfer, B. , Pheloung, P. C. , Ansari, S. , Buddenhagen, C. , Chimera, C. , Daehler, C. C. , Dawson, W. , Denslow, J. S. , LaRosa, A. , Nishida, T. , Onderdonk, D. A. , Panetta, F. , Pysek, P. , Randall, R. P. , *et al*. (2010). Guidance for addressing the Australian weed risk assessment questions. Plant Protection Quarterly 25, 56–74.

[brv70124-bib-0295] Gormley, A. M. , Penelope Holland, E. , Pech, R. P. , Thomson, C. & Reddiex, B. (2012). Impacts of an invasive herbivore on indigenous forests. Journal of Applied Ecology 49, 1296–1305.

[brv70124-bib-0296] Goulson, D. (2003). Effects of introduced bees on native ecosystems. Annual Review of Ecology, Evolution, and Systematics 34, 1–26.

[brv70124-bib-0297] Goulson, D. , Lye, G. C. & Darvill, B. (2008). Decline and conservation of bumble bees. Annual Review of Entomology 53, 191–208. 10.1146/annurev.ento.53.103106.093454 17803456

[brv70124-bib-0298] Gozlan, R. E. (2008). Introduction of non‐native freshwater fish: is it all bad? Fish and Fisheries 9, 106–115.

[brv70124-bib-0299] Green, D. S. & Crowe, T. P. (2014). Context‐ and density‐dependent effects of introduced oysters on biodiversity. Biological Invasions 16, 1145–1163.

[brv70124-bib-0300] Grez, A. A. , Zaviezo, T. , Roy, H. E. , Brown, P. M. J. & Bizama, G. (2016). Rapid spread of *Harmonia axyridis* in Chile and its effects on local coccinellid biodiversity. Diversity and Distributions 22, 982–994.

[brv70124-bib-0301] Gribsholt, B. & Kristensen, E. (2002). Effects of bioturbation and plant roots on salt marsh biogeochemistry: a mesocosm study. Marine Ecology Progress Series 241, 71–87.

[brv70124-bib-0302] Grimm, J. , Dick, J. T. A. , Verreycken, H. , Jeschke, J. M. , Linzmaier, S. & Ricciardi, A. (2020). Context‐dependent differences in the functional responses of conspecific native and non‐native crayfishes. NeoBiota 54, 71–88.

[brv70124-bib-0303] Grossart, H.‐P. , Van den Wyngaert, S. , Kagami, M. , Wurzbacher, C. , Cunliffe, M. & Rojas‐Jimenez, K. (2019). Fungi in aquatic ecosystems. Nature Reviews Microbiology 17, 339–354. 30872817 10.1038/s41579-019-0175-8

[brv70124-bib-0304] Guay, J. D. , Lennox, R. J. , Thorstad, E. B. , Vollset, K. W. , Stensland, S. , Erkinaro, J. & Nguyen, V. M. (2024). Recreational anglers in Norway report widespread dislike of invasive pink salmon. People and Nature 6, 41–53.

[brv70124-bib-0305] * Guerin, G. R. , Martín‐Forés, I. , Sparrow, B. & Lowe, A. J. (2018). The biodiversity impacts of non‐native species should not be extrapolated from biased single‐species studies. Biodiversity and Conservation 27, 785–790.

[brv70124-bib-0306] Guerin, G. R. , Martín‐Forés, I. , Munroe, S. E. M. , Sparrow, B. & Lowe, A. J. (2019). Alien plants alter the growth form ratio and structure of Australian grasslands. Applied Vegetation Science 22, 582–592.

[brv70124-bib-0307] * Guo, F. , Guan, R. , Li, Y. , Liu, Q. , Wang, X. , Yang, C. & Wang, J. (2025). Foundation models in bioinformatics. National Science Review 12, nwaf028. 40078374 10.1093/nsr/nwaf028 PMC11900445

[brv70124-bib-0308] * Guo, X. , Lao, J. , Dang, B. , Zhang, Y. , Yu, L. , Ru, L. , Zhong, L. , Huang, Z. , Wu, K. & Hu, D. (2024). Skysense: a multi‐modal remote sensing foundation model towards universal interpretation for earth observation imagery. In Proceedings of the IEEE/CVF Conference on Computer Vision and Pattern Recognition, pp. 27672–27683. IEEE, Piscataway, NJ.

[brv70124-bib-0309] Gurevitch, J. & Padilla, D. K. (2004). Are invasive species a major cause of extinctions? Trends in Ecology & Evolution 19, 470–474. 16701309 10.1016/j.tree.2004.07.005

[brv70124-bib-0310] Gutiérrez, J. L. , Jones, C. G. & Sousa, R. (2014). Toward an integrated ecosystem perspective of invasive species impacts. Acta Oecologica 54, 131–138.

[brv70124-bib-0311] Guzy, J. C. , Falk, B. G. , Smith, B. J. , Willson, J. D. , Reed, R. N. , Aumen, N. G. , Avery, M. L. , Bartoszek, I. A. , Campbell, E. , Cherkiss, M. S. , Claunch, N. M. , Currylow, A. F. , Dean, T. , Dixon, J. , Engeman, R. , *et al*. (2023). Burmese pythons in Florida: a synthesis of biology, impacts, and management tools. NeoBiota 80, 1–119.

[brv70124-bib-0312] Haines, A. M. , Costante, D. M. , Freed, C. , Achayaraj, G. , Bleyer, L. , Emeric, C. , Fenton, L. A. , Lielbriedis, L. , Ritter, E. , Salerni, G. I. , Stieha, C. R. , Isdell, R. E. & Leu, M. (2024). The impact of invasive alien species on threatened and endangered species: a geographic perspective. Wildlife Society Bulletin 48, e1552.

[brv70124-bib-0313] Haines‐Young, R. & Potschin‐Young, M. (2018). Revision of the common international classification for ecosystem services (CICES V5. 1): a policy brief. One Ecosystem 3, e27108.

[brv70124-bib-0314] Hajek, A. E. , Gardescu, S. & Delalibera, I. (2021). Summary of classical biological control introductions of entomopathogens and nematodes for insect control. BioControl 66, 167–180.

[brv70124-bib-0315] Hall, R. O. Jr. , Dybdahl, M. F. & VanderLoop, M. C. (2006). Extremely high secondary production of introduced snails in rivers. Ecological Applications 16, 1121–1131. 16827007 10.1890/1051-0761(2006)016[1121:ehspoi]2.0.co;2

[brv70124-bib-0316] Hare, D. J. (1980). Impact of defoliation by the Colorado potato beetle on potato yields. Journal of Economic Entomology 73, 369–373.

[brv70124-bib-0317] Harrington, L. A. , Harrington, A. L. , Yamaguchi, N. , Thom, M. D. , Ferreras, P. , Windham, T. R. & Macdonald, D. W. (2009). The impact of native competitors on an alien invasive: temporal niche shifts to avoid interspecific aggression. Ecology 90, 1207–1216. 19537542 10.1890/08-0302.1

[brv70124-bib-0318] Harvey, G. L. , Henshaw, A. J. , Brasington, J. & England, J. (2019). Burrowing invasive species: an unquantified erosion risk at the aquatic‐terrestrial interface. Reviews of Geophysics 57, 1018–1036.

[brv70124-bib-0319] Hasegawa, K. (2020). Invasions of rainbow trout and brown trout in Japan: a comparison of invasiveness and impact on native species. Ecology of Freshwater Fish 29, 419–428.

[brv70124-bib-0320] Hatcher, M. J. , Dick, J. T. A. , Bojko, J. , Stentiford, G. D. , Stebbing, P. & Dunn, A. M. (2019). Infection and invasion: study cases from aquatic communities. In Wildlife Disease Ecology (eds K. Wilson , A. Fenton and D. Tompkins ), pp. 262–295. Cambridge University Press, Cambridge, UK.

[brv70124-bib-0321] Hatcher, M. J. , Dick, J. T. A. & Dunn, A. M. (2012). Disease emergence and invasions. Functional Ecology 26, 1275–1287. 32313353 10.1111/j.1365-2435.2012.02031.x PMC7163950

[brv70124-bib-0322] Haubrock, P. J. , Ahmed, D. A. , Cuthbert, R. N. , Stubbington, R. , Domisch, S. , Marquez, J. R. G. , Beidas, A. , Amatulli, G. , Kiesel, J. , Shen, L. Q. , Soto, I. , Angeler, D. G. , Bonada, N. , Cañedo‐Argüelles, M. , Csabai, Z. , *et al*. (2022 *a*). Invasion impacts and dynamics of a European‐wide introduced species. Global Change Biology 28, 4620–4632. 35570183 10.1111/gcb.16207

[brv70124-bib-0323] Haubrock, P. J. , Balzani, P. , Macêdo, R. & Tarkan, A. S. (2023 *a*). Is the number of non‐native species in the European Union saturating? Environmental Sciences Europe 35, 48. 10.1186/s12302-023-00750-3 PMC10249565 37325080

[brv70124-bib-0324] Haubrock, P. J. , Bernery, C. , Cuthbert, R. N. , Liu, C. , Kourantidou, M. , Leroy, B. , Turbelin, A. J. , Kramer, A. M. , Verbrugge, L. N. H. , Diagne, C. , Courchamp, F. & Gozlan, R. E. (2022 *b*). Knowledge gaps in economic costs of invasive alien fish worldwide. Science of the Total Environment 803, 149875. 34478901 10.1016/j.scitotenv.2021.149875

[brv70124-bib-0325] Haubrock, P. J. , Carneiro, L. , Macêdo, R. L. , Balzani, P. , Soto, I. , Rasmussen, J. J. , Wiberg‐Larsen, P. , Csabai, Z. , Várbíró, G. , Murphy, J. F. , Jones, J. I. , Verdonschot, R. C. M. , Verdonschot, P. , van der Lee, G. & Ahmed, D. A. (2023 *b*). Advancing our understanding of biological invasions with long‐term biomonitoring data. Biological Invasions 25, 3637–3649.

[brv70124-bib-0326] Haubrock, P. J. , Cuthbert, R. N. , Balzani, P. , Briski, E. , Cano‐Barbacil, C. , De Santis, V. , Hudgins, E. J. , Kouba, A. , Macêdo, R. L. , Kourantidou, M. , Renault, D. , Rico‐Sánchez, A. E. , Soto, I. , Toutain, M. , Tricarico, E. , *et al*. (2024 *a*). Discrepancies between non‐native and invasive species classifications. Biological Invasions 26, 371–384.

[brv70124-bib-0327] Haubrock, P. J. , Cuthbert, R. N. , Hudgins, E. J. , Crystal‐Ornelas, R. , Kourantidou, M. , Moodley, D. , Liu, C. , Turbelin, A. J. , Leroy, B. & Courchamp, F. (2022 *c*). Geographic and taxonomic trends of rising biological invasion costs. Science of the Total Environment 817, 152948. 35032533 10.1016/j.scitotenv.2022.152948

[brv70124-bib-0328] Haubrock, P. J. , Cuthbert, R. N. , Ricciardi, A. , Diagne, C. & Courchamp, F. (2022 *d*). Economic costs of invasive bivalves in freshwater ecosystems. Diversity and Distributions 28, 1010–1021.

[brv70124-bib-0329] Haubrock, P. J. , Inghilesi, A. F. , Mazza, G. , Bendoni, M. , Solari, L. & Tricarico, E. (2019). Burrowing activity of *Procambarus clarkii* on levees: analysing behaviour and burrow structure. Wetlands Ecology and Management 27, 497–511.

[brv70124-bib-0330] Haubrock, P. J. , Parker, B. , Błońska, D. , Briski, E. , Everts, T. , Fernandez, R. D. , Kouba, A. , Kourantidou, M. , Kurtul, I. , Mammola, S. , Musolin, D. L. , Nuñez, M. A. , Olden, J. D. , Rasmussen, J. J. , Renault, D. , *et al*. (2025 *a*). Conceptual and ethical considerations in invasion science. Bioscience 75, 317–330. 40717710 10.1093/biosci/biae138 PMC12290455

[brv70124-bib-0331] Haubrock, P. J. , Pilotto, F. , Soto, I. , Kühn, I. , Verreycken, H. , Seebens, H. , Cuthbert, R. N. & Haase, P. (2023 *c*). Long‐term trends in abundances of non‐native species across biomes, realms, and taxonomic groups in Europe. Science of the Total Environment 884, 163808. 37127152 10.1016/j.scitotenv.2023.163808

[brv70124-bib-0332] Haubrock, P. J. & Soto, I. (2023). Valuing the information hidden in true long‐term data for invasion science. Biological Invasions 25, 2385–2394.

[brv70124-bib-0333] Haubrock, P. J. , Soto, I. , Ahmed, D. A. , Ansari, A. R. , Tarkan, A. S. , Kurtul, I. , Macêdo, R. L. , Lázaro‐Lobo, A. , Toutain, M. , Parker, B. , Błońska, D. , Guareschi, S. , Cano‐Barbacil, C. , Dominiguez Almela, V. , Adreou, D. , *et al*. (2024 *b*). Biological invasions are a population‐level rather than a species‐level phenomenon. Global Change Biology 30, e17312. 38736133 10.1111/gcb.17312

[brv70124-bib-0334] Haubrock, P. J. , Soto, I. , Cano‐Barbacil, C. , Theissinger, K. , Rios‐Henriquez, C. , Parker, B. , Weithoff, G. & Briski, E. (2025 *b*). Germany's established non‐native species: a comprehensive breakdown. Environmental Sciences Europe 37, 56.

[brv70124-bib-0335] Haubrock, P. J. , Tarkan, A. S. , Błońska, D. , Gora, A. , Pârvulescu, L. , Kouba, A. & Soto, I. (2025 *c*). Prioritising non‐native crayfish species for management in the Rhine‐Main observatory using the dispersal‐origin‐status‐impact (DOSI) scheme. Aquatic Conservation: Marine and Freshwater Ecosystems 35, e70191.

[brv70124-bib-0336] Hawkins, C. L. , Bacher, S. , Essl, F. , Hulme, P. E. , Jeschke, J. M. , Kühn, I. , Kumschick, S. , Nentwig, W. , Pergl, J. , Pyšek, P. , Rabitsch, W. , Richardson, D. M. , Vilà, M. , Wilson, J. R. U. , Genovesi, P. , *et al*. (2015). Framework and guidelines for implementing the proposed IUCN environmental impact classification for alien taxa (EICAT). Diversity and Distributions 21, 1360–1363.

[brv70124-bib-0337] He, Q. , Bertness, M. D. & Altieri, A. H. (2013). Global shifts towards positive species interactions with increasing environmental stress. Ecology Letters 16, 695–706. 23363430 10.1111/ele.12080

[brv70124-bib-0338] Hebert, C. E. , Chao, J. , Crump, D. , Johnson, T. B. , Rudy, M. D. , Sverko, E. , Williams, K. , Zaruk, D. & Arts, M. T. (2014). Ecological tracers track changes in bird diets and possible routes of exposure to type E botulism. Journal of Great Lakes Research 40, 64–70.

[brv70124-bib-0339] Helsen, K. , Kapás, R. E. , Rakvaag, G. , Speed, J. D. M. & Graae, B. J. (2018). Impacts of an invasive plant on primary production: testing a functional trait‐based framework with a greenhouse experiment. Journal of Vegetation Science 29, 157–166.

[brv70124-bib-0340] Helsen, K. , Matsushima, H. , Somers, B. & Honnay, O. (2021). A trait‐based approach across the native and invaded range to understand plant invasiveness and community impact. Oikos 130, 1001–1013.

[brv70124-bib-0341] Henderson, D. C. & Naeth, M. A. (2005). Multi‐scale impacts of crested wheatgrass invasion in mixed‐grass prairie. Biological Invasions 7, 639–650.

[brv70124-bib-0342] Henn, J. J. , Anderson, C. B. & Martínez Pastur, G. (2016). Landscape‐level impact and habitat factors associated with invasive beaver distribution in Tierra del Fuego. Biological Invasions 18, 1679–1688.

[brv70124-bib-0343] Henry, M. , Leung, B. , Cuthbert, R. N. , Bodey, T. W. , Ahmed, D. A. , Angulo, E. , Balzani, P. , Briski, E. , Courchamp, F. , Hulme, P. E. , Kouba, A. , Kourantidou, M. , Liu, C. , Macêdo, R. L. , Oficialdegui, F. J. , *et al*. (2023). Unveiling the hidden economic toll of biological invasions in the European Union. Environmental Sciences Europe 35, 43. 37325080 10.1186/s12302-023-00750-3 PMC10249565

[brv70124-bib-0344] Herms, D. A. & McCullough, D. G. (2014). Emerald ash borer invasion of North America: history, biology, ecology, impacts, and management. Annual Review of Entomology 59, 13–30. 10.1146/annurev-ento-011613-162051 24112110

[brv70124-bib-0345] Hill, M. , Holm, K. , Vel, T. , Shah, N. J. & Matyot, P. (2003). Impact of the introduced yellow crazy ant *Anoplolepis gracilipes* on Bird Island, Seychelles. Biodiversity and Conservation 12, 1969–1984.

[brv70124-bib-0346] * Hobson, E. A. , Smith‐Vidaurre, G. & Salinas‐Melgoza, A. (2017). History of nonnative monk parakeets in Mexico. PLoS One 12, e0184771. 28926594 10.1371/journal.pone.0184771 PMC5604984

[brv70124-bib-0347] Hoffmann, B. D. & Broadhurst, L. M. (2016). The economic cost of managing invasive species in Australia. NeoBiota 31, 1–18.

[brv70124-bib-0348] Holdich, D. M. , Reynolds, J. D. , Souty‐Grosset, C. & Sibley, P. J. (2009). A review of the ever increasing threat to European crayfish from non‐indigenous crayfish species. Knowledge & Management of Aquatic Ecosystems 11, 394–395.

[brv70124-bib-0349] Holdsworth, A. R. , Frelich, L. E. & Reich, P. B. (2007). Effects of earthworm invasion on plant species richness in northern hardwood forests. Conservation Biology 21, 997–1008. 17650250 10.1111/j.1523-1739.2007.00740.x

[brv70124-bib-0350] Holt, C. C. , Van der Giezen, M. , Daniels, C. L. , Stentiford, G. D. & Bass, D. (2020). Spatial and temporal axes impact ecology of the gut microbiome in juvenile European lobster (*Homarus gammarus*). The ISME Journal 14, 531–543. 31676854 10.1038/s41396-019-0546-1 PMC6976562

[brv70124-bib-0351] Holt, R. D. & Bonsall, M. B. (2017). Apparent competition. Annual Review of Ecology, Evolution, and Systematics 48, 447–471.

[brv70124-bib-0352] Hooker, J. D. (1864). Handbook of the New Zealand Flora. Reeve, London.

[brv70124-bib-0353] Hopkins, J. M. , Edwards, W. & Schwarzkopf, L. (2022). Invading the soundscape: exploring the effects of invasive species' calls on acoustic signals of native wildlife. Biological Invasions 24, 3381–3393.

[brv70124-bib-0354] Hossack, B. R. , Hall, D. , Crawford, C. L. , Goldberg, C. S. , Muths, E. , Sigafus, B. H. & Chambert, T. (2023). Successful eradication of invasive American bullfrogs leads to coextirpation of emerging pathogens. Conservation Letters 16, e12970.

[brv70124-bib-0355] Hou, G. , Bai, L. & Si, S. (2023). Ecosystem resilience and stability analysis against alien species invasion patterns. Physica A: Statistical Mechanics and its Applications 619, 128728.

[brv70124-bib-0356] Howard, B. R. , Barrios‐O'Neill, D. , Alexander, M. E. , Dick, J. T. A. , Therriault, T. W. , Robinson, T. B. & Côté, I. M. (2018). Functional responses of a cosmopolitan invader demonstrate intraspecific variability in consumer‐resource dynamics. PeerJ 6, e5634. 30280022 10.7717/peerj.5634 PMC6166631

[brv70124-bib-0357] Hoyle, H. , Hitchmough, J. & Jorgensen, A. (2017). Attractive, climate‐adapted and sustainable? Public perception of non‐native planting in the designed urban landscape. Landscape and Urban Planning 164, 49–63.

[brv70124-bib-0358] Hulme, P. E. (2014). Invasive species challenge the global response to emerging diseases. Trends in Parasitology 30, 267–270. 24862566 10.1016/j.pt.2014.03.005

[brv70124-bib-0359] * Hulme, P. E. (2015). New law risks release of invasive species. Nature 517, 21. 10.1038/517021a 25557705

[brv70124-bib-0360] Hulme, P. E. (2024). Networks of risk: international tourists as a biosecurity pathway into national parks. Biological Invasions 26, 4317–4330.

[brv70124-bib-0361] Hulme, P. E. , Pyšek, P. , Jarošík, V. , Pergl, J. , Schaffner, U. & Vilà, M. (2013). Bias and error in understanding plant invasion impacts. Trends in Ecology & Evolution 28, 212–218. 23153723 10.1016/j.tree.2012.10.010

[brv70124-bib-0362] Hulme‐Beaman, A. , Dobney, K. , Cucchi, T. & Searle, J. B. (2016). An ecological and evolutionary framework for commensalism in anthropogenic environments. Trends in Ecology & Evolution 31, 633–645. 27297117 10.1016/j.tree.2016.05.001

[brv70124-bib-0363] Human, K. G. & Gordon, D. M. (1996). Exploitation and interference competition between the invasive Argentine ant, *Linepithema humile*, and native ant species. Oecologia 105, 405–412. 28307114 10.1007/BF00328744

[brv70124-bib-0364] Huxel, G. R. (1999). Rapid displacement of native species by invasive species: effects of hybridization. Biological Conservation 89, 143–152.

[brv70124-bib-0365] Iacarella, J. C. , Dick, J. T. A. , Alexander, M. E. & Ricciardi, A. (2015 *a*). Ecological impacts of invasive alien species along temperature gradients: testing the role of environmental matching. Ecological Applications 25, 706–716. 26214916 10.1890/14-0545.1

[brv70124-bib-0366] Iacarella, J. C. , Dick, J. T. A. & Ricciardi, A. (2015 *b*). A spatio‐temporal contrast of the predatory impact of an invasive freshwater crustacean. Diversity and Distributions 21, 803–812.

[brv70124-bib-0367] Ilarri, M. I. , Freitas, F. , Costa‐Dias, S. , Antunes, C. , Guilhermino, L. & Sousa, R. (2012). Associated macrozoobenthos with the invasive Asian clam *Corbicula fluminea* . Journal of Sea Research 72, 113–120.

[brv70124-bib-0368] Iwanowicz, D. D. , Sanders, L. R. , Schill, W. B. , Xayavong, M. V. , da Silva, A. J. , Qvarnstrom, Y. & Smith, T. (2015). Spread of the rat lungworm (*Angiostrongylus cantonensis*) in giant African land snails (*Lissachatina fulica*) in Florida, USA. Journal of Wildlife Diseases 51, 749–753. 25973628 10.7589/2014-06-160

[brv70124-bib-0369] Jackson, M. C. , Britton, J. R. , Cucherousset, J. , Guo, Z. , Stakėnas, S. , Gozlan, R. E. , Godard, M. G. , Roussel, J.‐M. & Copp, G. H. (2016). Do non‐native pumpkinseed *Lepomis gibbosus* affect the growth, diet and trophic niche breadth of native brown trout *Salmo trutta*? Hydrobiologia 772, 63–75.

[brv70124-bib-0370] Jackson, M. C. , Ruiz‐Navarro, A. & Britton, J. R. (2015). Population density modifies the ecological impacts of invasive species. Oikos 124, 880–887.

[brv70124-bib-0371] * Jakubik, J. , Roy, S. , Phillips, C. E. , Fraccaro, P. , Godwin, D. , Zadrozny, B. , Szwarcman, D. , Gomes, C. , Nyirjesy, G. & Edwards, B. (2023). Foundation models for generalist geospatial artificial intelligence. *arXiv preprint arXiv:2310.18660*.

[brv70124-bib-0372] Jarić, I. , Courchamp, F. , Correia, R. A. , Crowley, S. L. , Essl, F. , Fischer, A. , González‐Moreno, P. , Kalinkat, G. , Lambin, X. , Lenzner, B. , Meinard, Y. , Mill, A. , Musseau, C. , Novoa, A. , Pergl, J. , *et al*. (2020). The role of species charisma in biological invasions. Frontiers in Ecology and the Environment 18, 345–353.

[brv70124-bib-0373] Jarić, I. , Fernández‐Llamazares, Á. , Molnár, Z. , Arbieu, U. , Correia, R. A. , Courchamp, F. , Essl, F. , Ladle, R. J. , Maurice, A.‐C. , Meinard, Y. , Novoa, A. , Nuñez, M. A. , Pyšek, P. , Roll, U. , Sbragaglia, V. , *et al*. (2024). Cultural inception of invasive species. npj Biodiversity 4, 1–12. 10.1038/s44185-025-00097-3 PMC12202807 40571732

[brv70124-bib-0374] Jemal, A. & Hugh‐Jones, M. (1993). A review of the red imported fire ant (*Solenopsis invicta* Buren) and its impacts on plant, animal, and human health. Preventive Veterinary Medicine 17, 19–32.

[brv70124-bib-0375] Jeschke, J. M. , Bacher, S. , Blackburn, T. M. , Dick, J. T. A. , Essl, F. , Evans, T. , Gaertner, M. , Hulme, P. E. , Kühn, I. , Mrugała, A. , Pergl, J. , Pyšek, P. , Rabitsch, W. , Ricciardi, A. , Richardson, D. M. , *et al*. (2014). Defining the impact of non‐native species. Conservation Biology 28, 1188–1194. 24779412 10.1111/cobi.12299 PMC4282110

[brv70124-bib-0376] Jeschke, J. M. , Gómez Aparicio, L. , Haider, S. , Heger, T. , Lortie, C. J. , Pyšek, P. & Strayer, D. L. (2012). Support for major hypotheses in invasion biology is uneven and declining. NeoBiota 14, 1–20.

[brv70124-bib-0377] Jeschke, J. M. & Heger, T. (2018). Invasion Biology: Hypotheses and Evidence. CAB International, Wallingford, Oxfordshire, UK.

[brv70124-bib-0378] Jones, B. A. (2017). Invasive species impacts on human well‐being using the life satisfaction index. Ecological Economics 134, 250–257.

[brv70124-bib-0379] Jones, C. G. , Lawton, J. H. & Shachak, M. (1994). Organisms as ecosystem engineers. Oikos 69, 373–386.

[brv70124-bib-0380] Jones, C. G. , Lawton, J. H. & Shachak, M. (1997). Positive and negative effects of organisms as physical ecosystem engineers. Ecology 78, 1946–1957.

[brv70124-bib-0381] Jones, P. & Closs, G. (2017). The introduction of brown trout to New Zealand and their impact on native fish communities. In Brown Trout: Biology, Ecology and Management, pp. 545–567. Wiley‐Blackwell, Hoboken, NJ.

[brv70124-bib-0382] Juliano, S. A. & Philip Lounibos, L. (2005). Ecology of invasive mosquitoes: effects on resident species and on human health. Ecology Letters 8, 558–574. 17637849 10.1111/j.1461-0248.2005.00755 PMC1920178

[brv70124-bib-0383] Jussila, J. , Edsman, L. , Maguire, I. , Diéguez‐Uribeondo, J. & Theissinger, K. (2021). Money kills native ecosystems: European crayfish as an example. Frontiers in Ecology and Evolution 9, 648495.

[brv70124-bib-0384] Kalisz, S. , Kivlin, S. N. & Bialic‐Murphy, L. (2021). Allelopathy is pervasive in invasive plants. Biological Invasions 23, 367–371.

[brv70124-bib-0385] Kamaru, D. N. , Palmer, T. M. , Riginos, C. , Ford, A. T. , Belnap, J. , Chira, R. M. , Githaiga, J. M. , Gituku, B. C. , Hays, B. R. , Kavwele, C. M. , Kibungei, A. K. , Lamb, C. T. , Maiyo, N. J. , Milligan, P. D. , Mutisya, S. , *et al*. (2024). Disruption of an ant‐plant mutualism shapes interactions between lions and their primary prey. Science 383, 433–438. 38271503 10.1126/science.adg1464

[brv70124-bib-0386] Kandori, I. , Hirao, T. , Matsunaga, S. & Kurosaki, T. (2009). An invasive dandelion unilaterally reduces the reproduction of a native congener through competition for pollination. Oecologia 159, 559–569. 19153768 10.1007/s00442-008-1250-4

[brv70124-bib-0387] Kapitza, K. , Zimmermann, H. , Martín‐López, B. & von Wehrden, H. (2019). Research on the social perception of invasive species: a systematic literature review. NeoBiota 43, 47–68.

[brv70124-bib-0388] Kato‐Noguchi, H. & Kato, M. (2024). Invasive characteristics of *Robinia pseudoacacia* and its impacts on species diversity. Diversity 16, 773.

[brv70124-bib-0389] Katsanevakis, S. , Deriu, I. , D'amico, F. , Nunes, A. L. , Sanchez, S. P. , Crocetta, F. , Arianoutsou, M. , Bazos, I. , Christopoulou, A. , Curto, G. , Delipetrou, P. , Kokkoris, Y. , Panov, V. E. , Rabitsch, W. , Roques, A. , *et al*. (2015). European alien species information network (EASIN): supporting European policies and scientific research. Management of Biological Invasions 6, 147.

[brv70124-bib-0390] Katsanevakis, S. & Moustakas, A. (2018). Uncertainty in marine invasion science. Frontiers in Marine Science 5, 38.

[brv70124-bib-0391] Katsanevakis, S. , Nikolaou, A. , Tsirintanis, K. & Rilov, G. (2025). Lessepsian migration in the Mediterranean Sea in an era of climate change: plague or boon? Science Talks 13, 100412.

[brv70124-bib-0392] Katsanevakis, S. , Tempera, F. & Teixeira, H. (2016). Mapping the impact of alien species on marine ecosystems: the Mediterranean Sea case study. Diversity and Distributions 22, 694–707.

[brv70124-bib-0393] Katsanevakis, S. , Wallentinus, I. , Zenetos, A. , Leppäkoski, E. , Çinar, M. E. , Oztürk, B. , Grabowski, M. , Golani, D. & Cardoso, A. C. (2014). Impacts of invasive alien marine species on ecosystem services and biodiversity: a pan‐European review. Aquatic Invasions 9, 391–423.

[brv70124-bib-0394] Keane, R. M. & Crawley, M. J. (2002). Exotic plant invasions and the enemy release hypothesis. Trends in Ecology & Evolution 17, 164–170.

[brv70124-bib-0395] * Keck, F. , Broadbent, H. & Altermatt, F. (2025 *a*). Extracting massive ecological data on state and interactions of species using large language models. bioRxiv. 10.1101/2025.01.24.634685.

[brv70124-bib-0396] Keck, F. , Peller, T. , Alther, R. , Barouillet, C. , Blackman, R. , Capo, E. , Chonova, T. , Couton, M. , Fehlinger, L. , Kirschner, D. , Knüsel, M. , Muneret, L. , Oester, R. , Tapolczai, K. , Zhang, H. , *et al*. (2025 *b*). The global human impact on biodiversity. Nature 641, 395–400. 40140566 10.1038/s41586-025-08752-2 PMC12058524

[brv70124-bib-0397] Kenis, M. , Auger‐Rozenberg, M.‐A. , Roques, A. , Timms, L. , Péré, C. , Cock, M. J. W. , Settele, J. , Augustin, S. & Lopez‐Vaamonde, C. (2009). Ecological effects of invasive alien insects. Biological Invasions 11, 21–45.

[brv70124-bib-0398] Kenis, M. , Nacambo, S. , Van Vlaenderen, J. , Zindel, R. & Eschen, R. (2020). Long term monitoring in Switzerland reveals that *Adalia bipunctata* strongly declines in response to *Harmonia axyridis* invasion. Insects 11, 883. 33322836 10.3390/insects11120883 PMC7764166

[brv70124-bib-0399] Kenis, M. , Roques, A. , Santini, A. & Liebhold, A. M. (2017). Impact of non‐native invertebrates and pathogens on market Forest tree resources. In Impact of Biological Invasions on Ecosystem Services, pp. 103–117. Springer International Publishing, Cham.

[brv70124-bib-0400] * Keller, R. P. , Geist, J. , Jeschke, J. M. & Kühn, I. (2011). Invasive species in Europe: ecology, status, and policy. Environmental Sciences Europe 23, 1–17.

[brv70124-bib-0401] * Kelly, R. , Harrod, C. , Maggs, C. A. & Reid, N. (2015). Effects of Elodea nuttallii on temperate freshwater plants, microalgae and invertebrates: small differences between invaded and uninvaded areas. Biological Invasions 17, 2123–2138.

[brv70124-bib-0402] Kingsolver, C. H. , Melching, J. S. & Bromfield, K. R. (1983). The threat of exotic plant pathogens to agriculture in the United States. Plant Disease 67, 595–600.

[brv70124-bib-0403] Kirch, P. V. (2017). On the Road of the Winds: An Archaeological History of the Pacific Islands before European Contact. Univ. of California Press, Berkeley, CA.

[brv70124-bib-0404] Kirkpatrick, M. & Ravigné, V. (2002). Speciation by natural and sexual selection: models and experiments. The American Naturalist 159, S22–S35. 10.1086/338370 18707367

[brv70124-bib-0405] Klimaszyk, P. , Klimaszyk, D. , Piotrowiak, M. & Popiołek, A. (2014). Unusual complications after occupational exposure to giant hogweed (*Heracleum mantegazzianum*): a case report. International Journal of Occupational Medicine and Environmental Health 27, 141–144. 24549994 10.2478/s13382-014-0238-z

[brv70124-bib-0406] Kollars, N. M. , Byers, J. E. & Sotka, E. E. (2016). Invasive décor: an association between a native decorator worm and a non‐native seaweed can be mutualistic. Marine Ecology Progress Series 545, 135–145.

[brv70124-bib-0407] Konishi, M. & Takata, K. (2004). Impact of asymmetrical hybridization followed by sterile F_1_ hybrids on species replacement in *Pseudorasbora* . Conservation Genetics 5, 463–474.

[brv70124-bib-0408] Kouba, A. , Oficialdegui, F. J. , Cuthbert, R. N. , Kourantidou, M. , South, J. , Tricarico, E. , Gozlan, R. E. , Courchamp, F. & Haubrock, P. J. (2022). Identifying economic costs and knowledge gaps of invasive aquatic crustaceans. Science of the Total Environment 813, 152325. 34971690 10.1016/j.scitotenv.2021.152325

[brv70124-bib-0409] Kourantidou, M. , Haubrock, P. J. , Cuthbert, R. N. , Bodey, T. W. , Lenzner, B. , Gozlan, R. E. , Nuñez, M. A. , Salles, J.‐M. , Diagne, C. & Courchamp, F. (2022). Invasive alien species as simultaneous benefits and burdens: trends, stakeholder perceptions and management. Biological Invasions 24, 1905–1926.

[brv70124-bib-0410] Kuebbing, S. E. (2020). How direct and indirect non‐native interactions can promote plant invasions, lead to invasional meltdown and inform management decisions. In Plant Invasions: The Role of Biotic Interactions, pp. 153–176. CAB International, Wallingford, Oxfordshire, UK.

[brv70124-bib-0411] Kueffer, C. , Klingler, G. , Zirfass, K. , Schumacher, E. , Edwards, P. J. & Güsewell, S. (2008). Invasive trees show only weak potential to impact nutrient dynamics in phosphorus‐poor tropical forests in the Seychelles. Functional Ecology 22, 359–366.

[brv70124-bib-0412] * Kumschick, S. , Gaertner, M. , Vilà, M. , Essl, F. , Jeschke, J. M. , Pyšek, P. , Ricciardi, A. , Bacher, S. , Blackburn, T. M. & Dick, J. T. A. (2015). Ecological impacts of alien species: quantification, scope, caveats, and recommendations. Bioscience 65, 55–63.

[brv70124-bib-0414] Kurle, C. M. , Croll, D. A. & Tershy, B. R. (2008). Introduced rats indirectly change marine rocky intertidal communities from algae‐to invertebrate‐dominated. Proceedings of the National Academy of Sciences 105, 3800–3804. 10.1073/pnas.0800570105 PMC2268820 18308929

[brv70124-bib-0415] Lacey, L. A. , Grzywacz, D. , Shapiro‐Ilan, D. I. , Frutos, R. , Brownbridge, M. & Goettel, M. S. (2015). Insect pathogens as biological control agents: Back to the future. Journal of Invertebrate Pathology 132, 1–41. 26225455 10.1016/j.jip.2015.07.009

[brv70124-bib-0416] Langhammer, P. F. , Bull, J. W. , Bicknell, J. E. , Oakley, J. L. , Brown, M. H. , Bruford, M. W. , Butchart, S. H. M. , Carr, J. A. , Church, D. , Cooney, R. , Cutajar, S. , Foden, W. , Foster, M. N. , Gascon, C. , Geldmann, J. , *et al*. (2024). The positive impact of conservation action. Science 384, 453–458. 38662833 10.1126/science.adj6598

[brv70124-bib-0417] Lant, C. L. , Ruhl, J. B. & Kraft, S. E. (2008). The tragedy of ecosystem services. Bioscience 58, 969–974.

[brv70124-bib-0418] * Lapeyrolerie, M. , Chapman, M. S. , Norman, K. E. A. & Boettiger, C. (2022). Deep reinforcement learning for conservation decisions. Methods in Ecology and Evolution 13, 2649–2662.

[brv70124-bib-0419] Largiadèr, C. R. (2007). Hybridization and introgression between native and alien species. Biological Invasions 16, 275–292.

[brv70124-bib-0420] Larkin, D. J. (2012). Lengths and correlates of lag phases in upper‐Midwest plant invasions. Biological Invasions 14, 827–838.

[brv70124-bib-0421] Larson, B. M. (2007). An alien approach to invasive species: objectivity and society in invasion biology. Biological Invasions 9, 947–956.

[brv70124-bib-0422] Larson, B. M. H. (2011). Embodied realism and invasive species. In Philosophy of Ecology, pp. 129–146. Elsevier, Amsterdam, The Netherlands.

[brv70124-bib-0423] Larson, B. M. H. & Kueffer, C. (2013). Managing invasive species amidst high uncertainty and novelty. Trends in Ecology & Evolution 28, 255–256. 23434389 10.1016/j.tree.2013.01.013

[brv70124-bib-0424] Latombe, G. , Catford, J. A. , Essl, F. , Lenzner, B. , Richardson, D. M. , Wilson, J. R. U. & McGeoch, M. A. (2022). GIRAE: a generalised approach for linking the total impact of invasion to species' range, abundance and per‐unit effects. Biological Invasions 24, 3147–3167. 36131994 10.1007/s10530-022-02836-0 PMC9482606

[brv70124-bib-0425] Latombe, G. , Seebens, H. , Lenzner, B. , Courchamp, F. , Dullinger, S. , Golivets, M. , Kühn, I. , Leung, B. , Roura‐Pascual, N. , Cebrian, E. , Dawson, W. , Diagne, C. , Jeschke, J. M. , Pérez‐Granados, C. , Moser, D. , *et al*. (2023). Capacity of countries to reduce biological invasions. Sustainability Science 18, 771–789. 37012996 10.1007/s11625-022-01166-3 PMC10063504

[brv70124-bib-0426] Laverty, C. , Green, K. D. , Dick, J. T. A. , Barrios‐O'Neill, D. , Mensink, P. J. , Médoc, V. , Spataro, T. , Caffrey, J. M. , Lucy, F. E. & Boets, P. (2017). Assessing the ecological impacts of invasive species based on their functional responses and abundances. Biological Invasions 19, 1653–1665.

[brv70124-bib-0427] Laverty, C. , Nentwig, W. , Dick, J. & Lucy, F. (2015). Alien aquatics in Europe: assessing the relative environmental and socio‐economic impacts of invasive aquatic macroinvertebrates and other taxa. Management of Biological Invasions 6, 341–350.

[brv70124-bib-0428] Lawson, L. L. , Hill, J. E. , Vilizzi, L. , Hardin, S. & Copp, G. H. (2013). Revisions of the fish invasiveness screening kit (FISK) for its application in warmer climatic zones, with particular reference to peninsular Florida. Risk Analysis 33, 1414–1431. 23035930 10.1111/j.1539-6924.2012.01896.x

[brv70124-bib-0429] Le Hen, G. , Balzani, P. , Haase, P. , Kouba, A. , Liu, C. , Nagelkerke, L. A. J. , Theissen, N. , Renault, D. , Soto, I. & Haubrock, P. J. (2023). Alien species and climate change drive shifts in a riverine fish community and trait compositions over 35 years. Science of the Total Environment 867, 161486. 36626991 10.1016/j.scitotenv.2023.161486

[brv70124-bib-0430] Le Roux, J. J. , Brown, G. K. , Byrne, M. , Ndlovu, J. , Richardson, D. M. , Thompson, G. D. & Wilson, J. R. U. (2011). Phylogeographic consequences of different introduction histories of invasive Australian *acacia* species and *Paraserianthes lophantha* (Fabaceae) in South Africa. Diversity and Distributions 17, 861–871.

[brv70124-bib-0431] Le Roux, J. J. , Ellis, A. G. , van Zyl, L. , Hosking, N. D. , Keet, J. & Yannelli, F. A. (2018). Importance of soil legacy effects and successful mutualistic interactions during Australian *cacia* invasions in nutrient‐poor environments. Journal of Ecology 106, 2071–2081.

[brv70124-bib-0432] LeBrun, E. G. , Ottens, K. J. & Gilbert, L. E. (2018). The microsporidian pathogen *Myrmecomorba nylanderiae*: Intracolony transmission and impact upon tawny crazy ant (*Nylanderia fulva*) colonies. Journal of Applied Entomology 142, 114–124.

[brv70124-bib-0433] Lenhardt, M. B. , Smederevac‐Lalić, M. M. , Spasić, S. Z. , Honţ, Ş. , Paraschiv, M. , Iani, M. I. , Nikčević, M. V. , Klimley, P. A. & Suciu, R. (2021). Seasonal changes in depth position and temperature of European catfish (*Silurus glanis*) tracked by acoustic telemetry in the Danube River. International Review of Hydrobiology 106, 191–201.

[brv70124-bib-0434] Lenzner, B. , Latombe, G. , Schertler, A. , Seebens, H. , Yang, Q. , Winter, M. , Weigelt, P. , Van Kleunen, M. , Pyšek, P. , Pergl, J. , Kreft, H. , Dawson, W. , Dullinger, S. & Essl, F. (2022). Naturalized alien floras still carry the legacy of European colonialism. Nature Ecology & Evolution 6, 1723–1732. 36253544 10.1038/s41559-022-01865-1

[brv70124-bib-0435] Leung, B. , Lodge, D. M. , Finnoff, D. , Shogren, J. F. , Lewis, M. A. & Lamberti, G. (2002). An ounce of prevention or a pound of cure: bioeconomic risk analysis of invasive species. Proceedings of the Royal Society of London. Series B: Biological Sciences 269, 2407–2413. 10.1098/rspb.2002.2179 PMC1691180 12495482

[brv70124-bib-0436] Leung, B. , Roura‐Pascual, N. , Bacher, S. , Heikkilä, J. , Brotons, L. , Burgman, M. A. , Dehnen‐Schmutz, K. , Essl, F. , Hulme, P. E. , Richardson, D. M. , Sol, D. & Vilà, M. (2012). TEASIng apart alien species risk assessments: a framework for best practices. Ecology Letters 15, 1475–1493. 23020170 10.1111/ele.12003

[brv70124-bib-0437] Levi, T. , Keesing, F. , Holt, R. D. , Barfield, M. & Ostfeld, R. S. (2016). Quantifying dilution and amplification in a community of hosts for tick‐borne pathogens. Ecological Applications 26, 484–498. 27209790 10.1890/15-0122

[brv70124-bib-0438] Lewis, S. L. & Maslin, M. A. (2015). Defining the anthropocene. Nature 519, 171–180. 25762280 10.1038/nature14258

[brv70124-bib-0439] Li, F.‐F. , Hao, Q. , Cui, X. , Lin, R.‐Z. , Luo, B.‐S. & Ma, J.‐S. (2024). Global invasive alien plant management lists: assessing current practices and adapting to new demands. Plant Diversity 47, 666–680. 40734832 10.1016/j.pld.2024.11.002 PMC12302625

[brv70124-bib-0440] Li, S. , Wei, H. , Kang, B. , Li, F. , Xu, D. , Xu, X. , Gu, D. & Stauffer, J. R. (2025). Application of the European non‐native species in aquaculture risk analysis scheme to evaluate *Micropterus nigricans* in China. Management of Biological Invasions 16, 153–165.

[brv70124-bib-0441] Linders, T. E. W. , Bekele, K. , Schaffner, U. , Allan, E. , Alamirew, T. , Choge, S. K. , Eckert, S. , Haji, J. , Muturi, G. , Mbaabu, P. R. , Shiferaw, H. & Eschen, R. (2020). The impact of invasive species on social‐ecological systems: relating supply and use of selected provisioning ecosystem services. Ecosystem Services 41, 101055.

[brv70124-bib-0442] * Lipták, B. , Kouba, A. , Zorić, K. , Salvaras, L. , Prokop, P. & Paunović, M. (2023). The attractiveness of freshwater species correlates positively with conservation support. Anthrozoös 36, 971–984.

[brv70124-bib-0443] * Litchman, E. (2010). Invisible invaders: non‐pathogenic invasive microbes in aquatic and terrestrial ecosystems. Ecology Letters 13, 1560–1572. 21054733 10.1111/j.1461-0248.2010.01544.x

[brv70124-bib-0444] Liu, D. , Semenchuk, P. , Essl, F. , Lenzner, B. , Moser, D. , Blackburn, T. M. , Cassey, P. , Biancolini, D. , Capinha, C. , Dawson, W. , Dyer, E. E. , Guénard, B. , Economo, E. P. , Kreft, H. , Pergl, J. , *et al*. (2023). The impact of land use on non‐native species incidence and number in local assemblages worldwide. Nature Communications 14, 2090. 10.1038/s41467-023-37571-0 PMC10097616 37045818

[brv70124-bib-0445] Liu, S.‐S. , De Barro, P. J. , Xu, J. , Luan, J.‐B. , Zang, L.‐S. , Ruan, Y.‐M. & Wan, F.‐H. (2007). Asymmetric mating interactions drive widespread invasion and displacement in a whitefly. Science 318, 1769–1772. 17991828 10.1126/science.1149887

[brv70124-bib-0446] Liu, X. , McGarrity, M. E. , Bai, C. , Ke, Z. & Li, Y. (2013). Ecological knowledge reduces religious release of invasive species. Ecosphere 4, 1–12.

[brv70124-bib-0447] Liu, X. , McGarrity, M. E. & Li, Y. (2012). The influence of traditional Buddhist wildlife release on biological invasions. Conservation Letters 5, 107–114.

[brv70124-bib-0448] Lloveras, L. , Maroto, J. , Soler, J. , Thomas, R. , Moreno‐García, M. , Nadal, J. & Soler, N. (2016). The role of small prey in human subsistence strategies from Early upper Palaeolithic sites in Iberia: the rabbits from the evolved Aurignacian level of Arbreda cave. Journal of Quaternary Science 31, 458–471.

[brv70124-bib-0449] Lockwood, J. L. , Hoopes, M. F. & Marchetti, M. P. (2013). Invasion ecology. John Wiley & Sons, Hoboken, NJ.

[brv70124-bib-0450] Löfqvist, S. , Kleinschroth, F. , Bey, A. , De Bremond, A. , DeFries, R. , Dong, J. , Fleischman, F. , Lele, S. , Martin, D. A. , Messerli, P. , Meyfroidt, P. , Pfeifer, M. , Rakotonarivo, S. O. , Ramankutty, N. , Ramprasad, V. , *et al*. (2023). How social considerations improve the equity and effectiveness of ecosystem restoration. Bioscience 73, 134–148. 36896142 10.1093/biosci/biac099 PMC9991587

[brv70124-bib-0451] Lönnstedt, O. M. & McCormick, M. I. (2013). Ultimate predators: lionfish have evolved to circumvent prey risk assessment abilities. PLoS One 8, e75781. 24146775 10.1371/journal.pone.0075781 PMC3797751

[brv70124-bib-0452] Lopez, B. E. , Allen, J. M. , Dukes, J. S. , Lenoir, J. , Vilà, M. , Blumenthal, D. M. , Beaury, E. M. , Fusco, E. J. , Laginhas, B. B. , Morelli, T. L. , O'Neill, M. W. , Sorte, C. J. B. , Maceda‐Veiga, A. , Whitlock, R. & Bradley, B. A. (2022). Global environmental changes more frequently offset than intensify detrimental effects of biological invasions. Proceedings of the National Academy of Sciences 119, e2117389119. 10.1073/pnas.2117389119 PMC9295750 35622892

[brv70124-bib-0453] Loureiro, V. R. & Barbosa, E. J. d. S. (2010). Cidade de Belém e natureza: uma relação problemática? Vol. 13, pp. 105–134. Novos Cadernos NAEA, Belém, PA, Brazil.

[brv70124-bib-0454] Lovett, G. M. , Canham, C. D. , Arthur, M. A. , Weathers, K. C. & Fitzhugh, R. D. (2006). Forest ecosystem responses to exotic pests and pathogens in eastern North America. Bioscience 56, 395–405.

[brv70124-bib-0455] Lovett, G. M. , Christenson, L. M. , Groffman, P. M. , Jones, C. G. , Hart, J. E. & Mitchell, M. J. (2002). Insect defoliation and nitrogen cycling in forests: laboratory, plot, and watershed studies indicate that most of the nitrogen released from forest foliage as a result of defoliation by insects is redistributed within the ecosystem, whereas only a small fraction of nitrogen is lost by leaching. Bioscience 52, 335–341.

[brv70124-bib-0456] Lovich, J. E. & Yamamoto, K. (2016). Measuring the impact of invasive species on popular culture: a case study based on toy turtles from Japan. Humans and Nature 27, 1–11.

[brv70124-bib-0457] Lucy, F. E. , Roy, H. , Simpson, A. , Carlton, J. T. , Hanson, J. M. , Magellan, K. , Campbell, M. L. , Costello, M. J. , Pagad, S. , Hewitt, C. L. , McDonald, J. , Cassey, P. C. , Thomaz, S. M. , Katsanevakis, S. , Zenetos, A. , *et al*. (2016). INVASIVESNET towards an international association for open knowledge on invasive alien species. Management of Biological Invasions 7, 131–139.

[brv70124-bib-0458] Lyach, R. (2022). Increasing dominance of non‐native fishes in the yield of central European streams and rivers. Fisheries Research 254, 106433.

[brv70124-bib-0459] Macdonald, I. A. W. & Richardson, D. M. (1986). Alien species in terrestrial ecosystems of the fynbos biome. In The Ecology and Management of Biological Invasions in Southern Africa (eds I. A. W. Macdonald *et al*.), pp. 77–91. Oxford University Press, Oxford, UK.

[brv70124-bib-0460] MacDougall, A. S. & Turkington, R. (2005). Are invasive species the drivers or passengers of change in degraded ecosystems? Ecology 86, 42–55.

[brv70124-bib-0461] Macêdo, R. L. , Haubrock, P. J. , Klippel, G. , Fernandez, R. D. , Leroy, B. , Angulo, E. , Carneiro, L. , Musseau, C. L. , Rocha, O. & Cuthbert, R. N. (2024). The economic costs of invasive aquatic plants: a global perspective on ecology and management gaps. Science of the Total Environment 908, 168217. 37952653 10.1016/j.scitotenv.2023.168217

[brv70124-bib-0462] Mack, M. C. & D'Antonio, C. M. (1998). Impacts of biological invasions on disturbance regimes. Trends in Ecology & Evolution 13, 195–198. 21238260 10.1016/S0169-5347(97)01286-X

[brv70124-bib-0463] Magliozzi, C. , Tsiamis, K. , Vigiak, O. , Deriu, I. , Gervasini, E. & Cardoso, A. C. (2020). Assessing invasive alien species in European catchments: distribution and impacts. Science of the Total Environment 732, 138677. 32422476 10.1016/j.scitotenv.2020.138677

[brv70124-bib-0464] Mahoney, P. J. , Beard, K. H. , Durso, A. M. , Tallian, A. G. , Long, A. L. , Kindermann, R. J. , Nolan, N. E. , Kinka, D. & Mohn, H. E. (2015). Introduction effort, climate matching and species traits as predictors of global establishment success in non‐native reptiles. Diversity and Distributions 21, 64–74.

[brv70124-bib-0465] Mallet, J. (2005). Hybridization as an invasion of the genome. Trends in Ecology & Evolution 20, 229–237. 16701374 10.1016/j.tree.2005.02.010

[brv70124-bib-0466] * Mallon, C. A. , Le Roux, X. , van Doorn, G. S. , Dini‐Andreote, F. , Poly, F. & Salles, J. F. (2018). The impact of failure: unsuccessful bacterial invasions steer the soil microbial community away from the invader's niche. The ISME Journal 12, 728–741. 29374268 10.1038/s41396-017-0003-y PMC5864238

[brv70124-bib-0467] Mammola, S. , Adamo, M. , Antić, D. , Calevo, J. , Cancellario, T. , Cardoso, P. , Chamberlain, D. , Chialva, M. , Durucan, F. , Fontaneto, D. , Goncalves, D. , Martínez, A. , Santini, L. , Rubio‐Lopez, I. , Sousa, R. , *et al*. (2023). Drivers of species knowledge across the tree of life. eLife 12, RP88251. 37846960 10.7554/eLife.88251 PMC10581686

[brv70124-bib-0468] Mammola, S. , Nanni, V. , Pantini, P. & Isaia, M. (2020). Media framing of spiders may exacerbate arachnophobic sentiments. People and Nature 2, 1145–1157.

[brv70124-bib-0469] Marchante, E. , Kjøller, A. , Struwe, S. & Freitas, H. (2008). Invasive *Acacia longifolia* induce changes in the microbial catabolic diversity of sand dunes. Soil Biology and Biochemistry 40, 2563–2568.

[brv70124-bib-0470] Marchessaux, G. , Mangano, M. C. , Bizzarri, S. , M'rabet, C. , Principato, E. , Lago, N. , Veyssiere, D. , Garrido, M. , Scyphers, S. B. & Sarà, G. (2023). Invasive blue crabs and small‐scale fisheries in the Mediterranean sea: local ecological knowledge, impacts and future management. Marine Policy 148, 105461.

[brv70124-bib-0471] * Marcot, B. G. , Hoff, M. H. , Martin, C. D. , Jewell, S. D. & Givens, C. E. (2019). A decision support system for identifying potentially invasive and injurious freshwater fishes. Management of Biological Invasions 10, 200–226.

[brv70124-bib-0472] Marr, S. M. , Olden, J. D. , Leprieur, F. , Arismendi, I. , Ćaleta, M. , Morgan, D. L. , Nocita, A. , Šanda, R. , Serhan Tarkan, A. & García‐Berthou, E. (2013). A global assessment of freshwater fish introductions in mediterranean‐climate regions. Hydrobiologia 719, 317–329.

[brv70124-bib-0473] Martin‐Albarracin, V. L. , Amico, G. C. , Simberloff, D. & Nuñez, M. A. (2015). Impact of non‐native birds on native ecosystems: a global analysis. PLoS One 10, e0143070. 26576053 10.1371/journal.pone.0143070 PMC4648570

[brv70124-bib-0474] Martín‐Forés, I. , Guerin, G. R. , Lewis, D. , Gallagher, R. V. , Vilà, M. , Catford, J. A. , Pauchard, A. & Sparrow, B. (2024). Towards integrating and harmonising information on plant invasions across Australia. NeoBiota 92, 61–83.

[brv70124-bib-0475] Matisoo‐Smith, E. & Robins, J. H. (2004). Origins and dispersals of Pacific peoples: evidence from mtDNA phylogenies of the Pacific rat. Proceedings of the National Academy of Sciences 101, 9167–9172. 10.1073/pnas.0403120101 PMC428491 15184658

[brv70124-bib-0476] Maunder, M. , Upson, T. , Spooner, B. & Kendle, T. (1995). Saint Helena: sustainable development and conservation of a highly Degraded Island ecosystem. In Islands: Biological Diversity and Ecosystem Function, pp. 205–217. Springer‐Verlag, Berlin, Germany.

[brv70124-bib-0477] Mayer, K. , Heger, T. , Kühn, I. , Nehring, S. & Gaertner, M. (2023). Germany's first action plan on the pathways of invasive alien species to prevent their unintentional introduction and spread. NeoBiota 89, 209–227.

[brv70124-bib-0478] Mazza, G. & Tricarico, E. (2018). Invasive Species and Human Health. CAB International, Wallingford, Oxfordshire, UK.

[brv70124-bib-0479] McGeoch, M. A. , Butchart, S. H. M. , Spear, D. , Marais, E. , Kleynhans, E. J. , Symes, A. , Chanson, J. & Hoffmann, M. (2010). Global indicators of biological invasion: species numbers, biodiversity impact and policy responses. Diversity and Distributions 16, 95–108.

[brv70124-bib-0480] McGeoch, M. A. , Ordonez, A. , Howard, P. L. , Groom, Q. J. , Shrestha, B. B. , Fernandez, M. , Brugnoli, E. , Bwalya, B. , Byun, C. , Ksenofontov, S. , Ojaveer, H. , Simberloff, D. , Mungi, N. A. & Rono, B. (2023). Chapter 6: governance and policy options for the management of biological invasions. In Thematic Assessment Report on Invasive Alien Species and their Control of the Intergovernmental Science‐Policy Platform on Biodiversity and Ecosystem Services. IPBES Secretariat, Bonn, Germany.

[brv70124-bib-0481] McKinney, M. L. & Lockwood, J. L. (1999). Biotic homogenization: a few winners replacing many losers in the next mass extinction. Trends in Ecology & Evolution 14, 450–453. 10511724 10.1016/s0169-5347(99)01679-1

[brv70124-bib-0482] Melbourne, B. A. , Cornell, H. V. , Davies, K. F. , Dugaw, C. J. , Elmendorf, S. , Freestone, A. L. , Hall, R. J. , Harrison, S. , Hastings, A. , Holland, M. , Holyoak, M. , Lambrinos, J. , Moore, K. & Yokomizo, H. (2007). Invasion in a heterogeneous world: resistance, coexistence or hostile takeover? Ecology Letters 10, 77–94. 17204119 10.1111/j.1461-0248.2006.00987.x

[brv70124-bib-0483] Meyer, L. & Hinrichs, D. (2000). Microhabitat preferences and movements of the weatherfish, *Misgurnus fossilis*, in a drainage channel. Environmental Biology of Fishes 58, 297–306.

[brv70124-bib-0484] Meyerson, L. A. , Chambers, R. M. & Vogt, K. A. (1999). The effects of *Phragmites* removal on nutrient pools in a freshwater tidal marsh ecosystem. Biological Invasions 1, 129–136.

[brv70124-bib-0485] Michailidis, N. , Katsanevakis, S. & Chartosia, N. (2020). Recreational fisheries can be of the same magnitude as commercial fisheries: the case of Cyprus. Fisheries Research 231, 105711.

[brv70124-bib-0486] Mitchell, E. & Dominguez Almela, V. (2025). Modelling the rise of invasive lionfish in the Mediterranean. Marine Biology 172, 18.

[brv70124-bib-0487] Mitchell, R. , Chitanava, S. , Dbar, R. , Kramarets, V. , Lehtijärvi, A. , Matchutadze, I. , Mamadashvili, G. , Matsiakh, I. , Nacambo, S. , Papazova‐Anakieva, I. , Sathyapala, S. , Tuniyev, B. , Vétek, G. , Zukhbaia, M. & Kenis, M. (2018). Identifying the ecological and societal consequences of a decline in *Buxus* forests in Europe and the Caucasus. Biological Invasions 20, 3605–3620.

[brv70124-bib-0488] Mitchell, R. J. , Hewison, R. L. , Hester, A. J. , Broome, A. & Kirby, K. J. (2016). Potential impacts of the loss of *Fraxinus excelsior* (Oleaceae) due to ash dieback on woodland vegetation in Great Britain. New Journal of Botany 6, 2–15.

[brv70124-bib-0489] Miura, O. & Torchin, M. E. (2023). Parasite release and biological invasions. In Parasites and Biological Invasions, pp. 24–41. CABI International, Wallingford, Oxfordshire, UK.

[brv70124-bib-0490] Miyamoto, K. , Fukuda, K. & Michita, Y. (2024). Evaluating the effectiveness of non‐native brown trout suppression to improve native white‐spotted charr stocking. Ichthyological Research 71, 522–528.

[brv70124-bib-0491] Moesch, S. S. , Jeschke, J. M. , Lokatis, S. , Peerenboom, G. , Kramer‐Schadt, S. , Straka, T. M. & Haase, D. (2024). The frequent five: insights from interviews with urban wildlife professionals in Germany. People and Nature 6, 2091–2108.

[brv70124-bib-0492] Mojžišová, M. , Svobodová, J. , Kozubíková‐Balcarová, E. , Štruncová, E. , Stift, R. , Bílý, M. , Kouba, A. & Petrusek, A. (2022). Long‐term changes in the prevalence of the crayfish plague pathogen and its genotyping in invasive crayfish species in Czechia. NeoBiota 74, 105–127.

[brv70124-bib-0493] Molinari, H. B. C. , Marur, C. J. , Daros, E. , De Campos, M. K. F. , De Carvalho, J. F. R. P. , Filho, J. C. B. , Pereira, L. F. P. & Vieira, L. G. E. (2007). Evaluation of the stress‐inducible production of proline in transgenic sugarcane (*Saccharum* spp.): osmotic adjustment, chlorophyll fluorescence and oxidative stress. Physiologia Plantarum 130, 218–229.

[brv70124-bib-0494] Molnar, J. L. , Gamboa, R. L. , Revenga, C. & Spalding, M. D. (2008). Assessing the global threat of invasive species to marine biodiversity. Frontiers in Ecology and the Environment 6, 485–492.

[brv70124-bib-0495] Moncada, M. , Nogueira, S. , Ribeiro, D. , Gago, J. , Rodrigues, M. , Alves, M. J. , Magalhães, M. F. , Curto, M. & Ribeiro, F. (2025). Hidden in the gut: Metabarcoding reveals overlooked predation by the invasive European catfish (*Silurus glanis*). Journal of Fish Biology 1–15. 10.1111/jfb.70152. 40831031

[brv70124-bib-0496] Montgomery, K. , Walden‐Schreiner, C. , Saffer, A. , Jones, C. , Seliger, B. J. , Worm, T. , Tateosian, L. , Shukunobe, M. , Kumar, S. & Meentemeyer, R. K. (2023). Forecasting global spread of invasive pests and pathogens through international trade. Ecosphere 14, e4740.

[brv70124-bib-0497] Moon, K. , Blackman, D. A. & Brewer, T. D. (2015). Understanding and integrating knowledge to improve invasive species management. Biological Invasions 17, 2675–2689.

[brv70124-bib-0498] Mooney, H. A. & Cleland, E. E. (2001). The evolutionary impact of invasive species. Proceedings of the National Academy of Sciences 98, 5446–5451. 10.1073/pnas.091093398 PMC33232 11344292

[brv70124-bib-0499] Morales, C. L. & Traveset, A. (2009). A meta‐analysis of impacts of alien vs. native plants on pollinator visitation and reproductive success of co‐flowering native plants. Ecology Letters 12, 716–728. 19453616 10.1111/j.1461-0248.2009.01319.x

[brv70124-bib-0500] * Morera, A. (2024). Foundation models in shaping the future of ecology. Ecological Informatics 80, 102545.

[brv70124-bib-0501] Mougi, A. (2016). The roles of amensalistic and commensalistic interactions in large ecological network stability. Scientific Reports 6, 29929. 27406267 10.1038/srep29929 PMC4942820

[brv70124-bib-0502] Moure, N. (1982). Impressionism, post‐impressionism, and the eucalyptus school in Southern California. Plein Air Painters of California: the Southland 1, 4–15.

[brv70124-bib-0503] Muhlfeld, C. C. , Kalinowski, S. T. , McMahon, T. E. , Taper, M. L. , Painter, S. , Leary, R. F. & Allendorf, F. W. (2009). Hybridization rapidly reduces fitness of a native trout in the wild. Biology Letters 5, 328–331. 19324629 10.1098/rsbl.2009.0033 PMC2679930

[brv70124-bib-0504] Muller, G. C. , Junnila, A. , Traore, M. M. , Traore, S. F. , Doumbia, S. , Sissoko, F. , Dembele, S. M. , Schlein, Y. , Arheart, K. L. , Revay, E. E. , Kravchenko, V. D. , Witt, A. & Beier, J. C. (2017). The invasive shrub *Prosopis juliflora* enhances the malaria parasite transmission capacity of *Anopheles* mosquitoes: a habitat manipulation experiment. Malaria Journal 16, 1–9. 28676093 10.1186/s12936-017-1878-9 PMC5497341

[brv70124-bib-0505] Mumford, J. D. , Booy, O. , Baker, R. H. A. , Rees, M. , Copp, G. H. , Black, K. , Holt, J. , Leach, A. W. & Hartley, M. (2010). Invasive non‐native species risk assessment in Great Britain. Aspects of Applied Biology 104, 49–54.

[brv70124-bib-0506] Munro, L. , Griffin, B. , Laginhas, B. B. & Bradley, B. A. (2024). Does invasion science encompass the invaded range? A comparison of the geographies of invasion science versus management in the US. Biological Invasions 26, 797–815.

[brv70124-bib-0507] * Murphy, C. A. , Grenouillet, G. & García‐Berthou, E. (2015). Natural abiotic factors more than anthropogenic perturbation shape the invasion of eastern mosquitofish (*Gambusia holbrooki*). Freshwater Science 34, 965–974.

[brv70124-bib-0508] Narščius, A. , Olenin, S. , Zaiko, A. & Minchin, D. (2012). Biological invasion impact assessment system: from idea to implementation. Ecological Informatics 7, 46–51.

[brv70124-bib-0509] Nentwig, W. , Bacher, S. , Pyšek, P. , Vilà, M. & Kumschick, S. (2016). The generic impact scoring system (GISS): a standardized tool to quantify the impacts of alien species. Environmental Monitoring and Assessment 188, 1–13. 27129597 10.1007/s10661-016-5321-4

[brv70124-bib-0510] Nentwig, W. , Kühnel, E. & Bacher, S. (2010). A generic impact‐scoring system applied to alien mammals in Europe. Conservation Biology 24, 302–311. 19604296 10.1111/j.1523-1739.2009.01289.x

[brv70124-bib-0511] Nentwig, W. , Mebs, D. & Vilà, M. (2017). Impact of non‐native animals and plants on human health. Impact of Biological Invasions on Ecosystem Services 12, 277–293.

[brv70124-bib-0512] Newfield, M. J. & Champion, P. D. (2010). Risk assessment for the New Zealand National Pest Plant Accord: which species should be banned from sale? Plant Protection Quarterly 25, 75–78.

[brv70124-bib-0513] Nikolaou, A. & Katsanevakis, S. (2023). Marine extinctions and their drivers. Regional Environmental Change 23, 88.

[brv70124-bib-0514] Northfield, T. D. , Laurance, S. G. W. , Mayfield, M. M. , Paini, D. R. , Snyder, W. E. , Stouffer, D. B. , Wright, J. T. & Lach, L. (2018). Native turncoats and indirect facilitation of species invasions. Proceedings of the Royal Society B: Biological Sciences 285, 20171936. 10.1098/rspb.2017.1936 PMC5805925 29367390

[brv70124-bib-0515] Novais, A. , Souza, A. T. , Ilarri, M. , Pascoal, C. & Sousa, R. (2015). Facilitation in the low intertidal: effects of an invasive species on the structure of an estuarine macrozoobenthic assemblage. Marine Ecology Progress Series 522, 157–167.

[brv70124-bib-0516] Novoa, A. , Dehnen‐Schmutz, K. , Fried, J. & Vimercati, G. (2017). Does public awareness increase support for invasive species management? Promising evidence across taxa and landscape types. Biological Invasions 19, 3691–3705.

[brv70124-bib-0517] Novoa, A. , Kaplan, H. , Wilson, J. R. U. & Richardson, D. M. (2016). Resolving a prickly situation: involving stakeholders in invasive cactus management in South Africa. Environmental Management 57, 998–1008. 26935429 10.1007/s00267-015-0645-3

[brv70124-bib-0518] Novoa, A. , Richardson, D. M. , Pyšek, P. , Meyerson, L. A. , Bacher, S. , Canavan, S. , Catford, J. A. , Čuda, J. , Essl, F. , Foxcroft, L. C. , Genovesi, P. , Hirsch, H. , Hui, C. , Jackson, M. C. , Kueffer, C. , *et al*. (2020). Invasion syndromes: a systematic approach for predicting biological invasions and facilitating effective management. Biological Invasions 22, 1801–1820.

[brv70124-bib-0519] Nuñez, M. A. , Dimarco, R. D. & Simberloff, D. (2018). Why some exotic species are deeply integrated into local cultures while others are reviled. In From Biocultural Homogenization to Biocultural Conservation, Ecology and Ethics, Vol. 3, pp. 219–231. Springer International Publishing, Cham, Switzerland.

[brv70124-bib-0520] Nuñez, M. A. , Pauchard, A. & Ricciardi, A. (2020). Invasion science and the global spread of SARS‐CoV‐2. Trends in Ecology & Evolution 35, 642–645. 32487347 10.1016/j.tree.2020.05.004 PMC7236691

[brv70124-bib-0521] Nuñez, M. A. & Simberloff, D. (2005). Invasive species and the cultural keystone species concept. Ecology and Society 10, r4.

[brv70124-bib-0522] Nyasembe, V. O. , Cheseto, X. , Kaplan, F. , Foster, W. A. , Teal, P. E. A. , Tumlinson, J. H. , Borgemeister, C. & Torto, B. (2015). The invasive American weed *Parthenium hysterophorus* can negatively impact malaria control in Africa. PLoS One 10, e0137836. 26367123 10.1371/journal.pone.0137836 PMC4569267

[brv70124-bib-0523] Odum, E. P. (1969). The strategy of ecosystem development. Science 164, 262–270. 5776636 10.1126/science.164.3877.262

[brv70124-bib-0524] Oficialdegui, F. J. , Clavero, M. , Sánchez, M. I. , Green, A. J. , Boyero, L. , Michot, T. C. , Klose, K. , Kawai, T. & Lejeusne, C. (2019). Unravelling the global invasion routes of a worldwide invader, the red swamp crayfish (*Procambarus clarkii*). Freshwater Biology 64, 1382–1400.

[brv70124-bib-0525] Oficialdegui, F. J. , Soto, I. , Balzani, P. , Cuthbert, R. N. , Haubrock, P. J. , Kourantidou, M. , Manfrini, E. , Tarkan, A. S. , Kurtul, I. , Macêdo, R. L. , Musseau, C. L. , Roy, K. & Kouba, A. (2025). Non‐native species in aquaculture: burgeoning production and environmental sustainability risks. Reviews in Aquaculture 17, e70037.

[brv70124-bib-0526] Ogden, N. H. , Wilson, J. R. U. , Richardson, D. M. , Hui, C. , Davies, S. J. , Kumschick, S. , Le Roux, J. J. , Measey, J. , Saul, W.‐C. & Pulliam, J. R. C. (2019). Emerging infectious diseases and biological invasions: a call for a one health collaboration in science and management. Royal Society Open Science 6, 181577. 31032015 10.1098/rsos.181577 PMC6458372

[brv70124-bib-0527] Ojaveer, H. , Galil, B. S. , Campbell, M. L. , Carlton, J. T. , Canning‐Clode, J. , Cook, E. J. , Davidson, A. D. , Hewitt, C. L. , Jelmert, A. , Marchini, A. , McKenzie, C. H. , Minchin, D. , Occhipinti‐Ambrogi, A. , Olenin, S. & Ruiz, G. (2015). Classification of non‐indigenous species based on their impacts: considerations for application in marine management. PLoS Biology 13, e1002130. 25875845 10.1371/journal.pbio.1002130 PMC4398364

[brv70124-bib-0528] Olden, J. D. , Poff, N. L. , Douglas, M. R. , Douglas, M. E. & Fausch, K. D. (2004). Ecological and evolutionary consequences of biotic homogenization. Trends in Ecology & Evolution 19, 18–24. 16701221 10.1016/j.tree.2003.09.010

[brv70124-bib-0529] O'Loughlin, L. S. & Green, P. T. (2017). Secondary invasion: when invasion success is contingent on other invaders altering the properties of recipient ecosystems. Ecology and Evolution 7, 7628–7637. 29043020 10.1002/ece3.3315 PMC5632608

[brv70124-bib-0530] Orive, M. E. & Barton, N. H. (2002). Associations between cytoplasmic and nuclear loci in hybridizing populations. Genetics 162, 1469–1485. 12454089 10.1093/genetics/162.3.1469 PMC1462324

[brv70124-bib-0531] Ostfeld, R. S. & Keesing, F. (2000). Biodiversity and disease risk: the case of Lyme disease. Conservation Biology 14, 722–728.

[brv70124-bib-0532] Palstra, F. P. & Ruzzante, D. E. (2008). Genetic estimates of contemporary effective population size: what can they tell us about the importance of genetic stochasticity for wild population persistence? Molecular Ecology 17, 3428–3447. 19160474 10.1111/j.1365-294x.2008.03842.x

[brv70124-bib-0533] Parker, I. M. , Simberloff, D. , Lonsdale, W. M. , Goodell, K. , Wonham, M. , Kareiva, P. M. , Williamson, M. H. , Von Holle, B. , Moyle, P. B. , Byers, J. E. & Goldwasser, L. (1999). Impact: toward a framework for understanding the ecological effects of invaders. Biological Invasions 1, 3–19.

[brv70124-bib-0534] Parra‐Tabla, V. & Arceo‐Gómez, G. (2021). Impacts of plant invasions in native plant–pollinator networks. New Phytologist 230, 2117–2128. 33710642 10.1111/nph.17339

[brv70124-bib-0535] Pärtel, M. , Szava‐Kovats, R. & Zobel, M. (2011). Dark diversity: shedding light on absent species. Trends in Ecology & Evolution 26, 124–128. 21195505 10.1016/j.tree.2010.12.004

[brv70124-bib-0536] Pärtel, M. , Tamme, R. , Carmona, C. P. , Riibak, K. , Moora, M. , Bennett, J. A. , Chiarucci, A. , Chytrý, M. , De Bello, F. , Eriksson, O. , Harrison, S. , Lewis, R. J. , Moles, A. T. , Öpik, M. , Price, J. N. , *et al*. (2025). Global impoverishment of natural vegetation revealed by dark diversity. Nature 641, 917–924. 40175550 10.1038/s41586-025-08814-5 PMC12095060

[brv70124-bib-0537] Pascual, M. A. , Cussac, V. , Dyer, B. , Soto, D. , Vigliano, P. , Ortubay, S. & Macchi, P. (2007). Freshwater fishes of Patagonia in the 21st century after a hundred years of human settlement, species introductions, and environmental change. Aquatic Ecosystem Health & Management 10, 212–227.

[brv70124-bib-0538] * Patoka, J. , Magalhães, A. L. B. , Kouba, A. , Faulkes, Z. , Jerikho, R. & Vitule, J. R. S. (2018). Invasive aquatic pets: failed policies increase risks of harmful invasions. Biodiversity and Conservation 27, 3037–3046.

[brv70124-bib-0539] Pearson, D. E. , Ortega, Y. K. , Eren, Ö. & Hierro, J. L. (2016). Quantifying “apparent” impact and distinguishing impact from invasiveness in multispecies plant invasions. Ecological Applications 26, 162–173. 27039517 10.1890/14-2345

[brv70124-bib-0540] Pejchar, L. & Mooney, H. A. (2009). Invasive species, ecosystem services and human well‐being. Trends in Ecology & Evolution 24, 497–504. 19577817 10.1016/j.tree.2009.03.016

[brv70124-bib-0541] Peltzer, D. A. , Allen, R. B. , Lovett, G. M. , Whitehead, D. & Wardle, D. A. (2010). Effects of biological invasions on forest carbon sequestration. Global Change Biology 16, 732–746.

[brv70124-bib-0542] Peñafiel‐Ricaurte, A. , Price, S. J. , Leung, W. T. M. , Alvarado‐Rybak, M. , Espinoza‐Zambrano, A. , Valdivia, C. , Cunningham, A. A. & Azat, C. (2023). Is Xenopus laevis introduction linked with Ranavirus incursion, persistence and spread in Chile? PeerJ 11, e14497. 36874973 10.7717/peerj.14497 PMC9979829

[brv70124-bib-0543] Penk, M. , Saul, W. , Dick, J. T. A. , Donohue, I. , Alexander, M. E. , Linzmaier, S. & Jeschke, J. M. (2017). A trophic interaction framework for identifying the invasive capacity of novel organisms. Methods in Ecology and Evolution 8, 1786–1794.

[brv70124-bib-0544] Pereyra, P. J. , de la Barra, P. , Amione, L. L. D. , Arcángel, A. , Marello Buch, B. M. , Rodríguez, E. , Maldonado, M. A. , Hünicken, L. , Lundgren, E. & Wallach, A. D. (2025). Human beings and the species they introduce are not a “cancer” of planet earth. Bioscience 75, 351–353.

[brv70124-bib-0545] Pereyra, P. J. , de la Barra, P. , Amione, L. L. D. , Arcángel, A. , Marello Buch, B. M. , Rodríguez, E. , Mazzolari, A. , Maldonado, M. A. , Hünicken, L. & Wallach, A. D. (2024). Systematic and persistent bias against introduced species. Bioscience 74, 44–53.

[brv70124-bib-0546] Pergl, J. , Pyšek, P. , Essl, F. , Jeschke, J. M. , Courchamp, F. , Geist, J. , Hejda, M. , Kowarik, I. , Mill, A. , Musseau, C. , Pipek, P. , Saul, W.‐C. , von Schmalensee, M. & Strayer, D. (2019). Need for routine tracking of biological invasions. Conservation Biology 34, 1311–1314. 31773813 10.1111/cobi.13445

[brv70124-bib-0547] Perpillou, A. (1933). Un fléau agricole: le doryphore. Annales de Géographie 236, 113–126.

[brv70124-bib-0548] Perrings, C. , Dehnen‐Schmutz, K. , Touza, J. & Williamson, M. (2005). How to manage biological invasions under globalization. Trends in Ecology & Evolution 20, 212–215. 16701371 10.1016/j.tree.2005.02.011

[brv70124-bib-0549] Peterson, M. I. , Kitano, S. , Yamamoto, S. , Kando, T. & Tsuda, Y. (2024). Species‐specific foraging behavior and diets of stream salmonids: an implication for negative impacts on native charr by nonnative trout in Japanese mountain streams. Ecological Research 39, 169–181.

[brv70124-bib-0550] Peyton, J. , Martinou, A. F. , Pescott, O. L. , Demetriou, M. , Adriaens, T. , Arianoutsou, M. , Bazos, I. , Bean, C. W. , Booy, O. , Botham, M. , Britton, J. R. , Lobon Cervia, J. , Charilaou, P. , Chartosia, N. , Dean, H. J. , *et al*. (2019). Horizon scanning for invasive alien species with the potential to threaten biodiversity and human health on a Mediterranean Island. Biological Invasions 21, 2107–2125.

[brv70124-bib-0551] Peyton, J. M. , Martinou, A. F. , Adriaens, T. , Chartosia, N. , Karachle, P. K. , Rabitsch, W. , Tricarico, E. , Arianoutsou, M. , Bacher, S. , Bazos, I. , Brundu, G. , Bruno‐McClung, E. , Charalambidou, I. , Demetriou, M. , Galanidi, M. , *et al*. (2020). Horizon scanning to predict and prioritize invasive alien species with the potential to threaten human health and economies on Cyprus. Frontiers in Ecology and Evolution 8, 566281.

[brv70124-bib-0552] Pfeiffer, J. M. & Voeks, R. A. (2008). Biological invasions and biocultural diversity: linking ecological and cultural systems. Environmental Conservation 35, 281–293.

[brv70124-bib-0553] Pheloung, P. C. , Williams, P. A. & Halloy, S. R. (1999). A weed risk assessment model for use as a biosecurity tool evaluating plant introductions. Journal of Environmental Management 57, 239–251.

[brv70124-bib-0554] Phillips, B. L. & Shine, R. (2004). Adapting to an invasive species: toxic cane toads induce morphological change in Australian snakes. Proceedings of the National Academy of Sciences 101, 17150–17155. 10.1073/pnas.0406440101 PMC535375 15569943

[brv70124-bib-0555] Piazzi, L. , Balata, D. , Foresi, L. , Cristaudo, C. & Cinelli, F. (2007). Sediment as a constituent of Mediterranean benthic communities dominated by *Caulerpa racemosa* var. *cylindracea* . Scientia Marina 71, 129–135.

[brv70124-bib-0556] Pimentel, D. , Zuniga, R. & Morrison, D. (2005). Update on the environmental and economic costs associated with alien‐invasive species in the United States. Ecological Economics 52, 273–288.

[brv70124-bib-0557] Pinero‐Rodríguez, M. J. , Fernández‐Zamudio, R. , Arribas, R. , Gomez‐Mestre, I. & Díaz‐Paniagua, C. (2021). The invasive aquatic fern *Azolla filiculoides* negatively impacts water quality, aquatic vegetation and amphibian larvae in Mediterranean environments. Biological Invasions 23, 755–769.

[brv70124-bib-0558] Piquet, J. C. & López‐Darias, M. (2021). Invasive snake causes massive reduction of all endemic herpetofauna on gran Canaria. Proceedings of the Royal Society B 288, 20211939. 34875190 10.1098/rspb.2021.1939 PMC8651408

[brv70124-bib-0559] Planchon, J.‐E. (1874). Le phylloxéra en Europe et en Amérique. J. Claye, Paris, France.

[brv70124-bib-0560] Plowes, R. M. , Becnel, J. J. , LeBrun, E. G. , Oi, D. H. , Valles, S. M. , Jones, N. T. & Gilbert, L. E. (2015). *Myrmecomorba nylanderiae* gen. Et sp. nov., a microsporidian parasite of the tawny crazy ant *Nylanderia fulva* . Journal of Invertebrate Pathology 129, 45–56. 26031565 10.1016/j.jip.2015.05.012

[brv70124-bib-0561] Polce, C. , Cardoso, A. C. , Deriu, I. , Gervasini, E. , Tsiamis, K. , Vigiak, O. , Zulian, G. & Maes, J. (2023). Invasive alien species of policy concerns show widespread patterns of invasion and potential pressure across European ecosystems. Scientific Reports 13, 8124. 37208377 10.1038/s41598-023-32993-8 PMC10199087

[brv70124-bib-0562] Polis, G. A. , Myers, C. A. & Holt, R. D. (1989). The ecology and evolution of intraguild predation: potential competitors that eat each other. Annual Review of Ecology, Evolution, and Systematics 20, 297–330.

[brv70124-bib-0563] Porretta, D. & Canestrelli, D. (2023). The ecological importance of hybridization. Trends in Ecology & Evolution 38, 1097–1108. 37620217 10.1016/j.tree.2023.07.003

[brv70124-bib-0564] Prahlow, J. A. & Barnard, J. J. (1998). Fatal anaphylaxis due to fire ant stings. The American Journal of Forensic Medicine and Pathology 19, 137–142. 9662108 10.1097/00000433-199806000-00007

[brv70124-bib-0565] Preisser, E. L. , Bolnick, D. I. & Benard, M. F. (2005). Scared to death? The effects of intimidation and consumption in predator–prey interactions. Ecology 86, 501–509.

[brv70124-bib-0566] Prestes, J. G. , Carneiro, L. , Miiller, N. O. R. , Neundorf, A. K. A. , Pedroso, C. R. , Braga, R. R. , Sousa, R. & Vitule, J. R. S. (2024). A systematic review of invasive non‐native freshwater bivalves. Biological Reviews 99, 2082–2107. 38973333 10.1111/brv.13113

[brv70124-bib-0567] Price, S. J. , Garner, T. W. J. , Nichols, R. A. , Balloux, F. , Ayres, C. , de Alba, A. M.‐C. & Bosch, J. (2014). Collapse of amphibian communities due to an introduced Ranavirus. Current Biology 24, 2586–2591. 25438946 10.1016/j.cub.2014.09.028

[brv70124-bib-0568] Pringle, R. M. (2005). The origins of the Nile perch in Lake Victoria. Bioscience 55, 780–787.

[brv70124-bib-0569] Prior, K. M. , Adams, D. C. , Klepzig, K. D. & Hulcr, J. (2018). When does invasive species removal lead to ecological recovery? Implications for management success. Biological Invasions 20, 267–283.

[brv70124-bib-0570] Pyšek, P. , Richardson, D. M. , Pergl, J. , Jarošík, V. , Sixtová, Z. & Weber, E. (2008). Geographical and taxonomic biases in invasion ecology. Trends in Ecology & Evolution 23, 237–244. 18367291 10.1016/j.tree.2008.02.002

[brv70124-bib-0571] Quilodrán, C. S. , Montoya‐Burgos, J. I. & Currat, M. (2020). Harmonizing hybridization dissonance in conservation. Communications Biology 3, 391. 32694629 10.1038/s42003-020-1116-9 PMC7374702

[brv70124-bib-0572] Rabenold, K. N. , Fauth, P. T. , Goodner, B. W. , Sadowski, J. A. & Parker, P. G. (1998). Response of avian communities to disturbance by an exotic insect in spruce‐fir forests of the southern Appalachians. Conservation Biology 12, 177–189.

[brv70124-bib-0573] Radinger, J. & García‐Berthou, E. (2020). The role of connectivity in the interplay between climate change and the spread of alien fish in a large Mediterranean river. Global Change Biology 26, 6383–6398. 32813898 10.1111/gcb.15320

[brv70124-bib-0574] Rahel, F. J. (2000). Homogenization of fish faunas across the United States. Science 288, 854–856. 10797007 10.1126/science.288.5467.854

[brv70124-bib-0575] Rajmis, S. , Thiele, J. C. & Marggraf, R. (2016). A cost‐benefit analysis of controlling giant hogweed (*Heracleum mantegazzianum*) in Germany using a choice experiment approach. NeoBiota 31, 19–41.

[brv70124-bib-0576] * Rato, J. , Brandão, P. , Anastácio, P. M. & Banha, F. (2024). First records of Mauremys sinensis in Portugal: a consequence of inadequate policies applied to the exotic pet market. Aquatic Ecology 58, 1091–1096.

[brv70124-bib-0577] Rayner, M. J. , Hauber, M. E. , Imber, M. J. , Stamp, R. K. & Clout, M. N. (2007). Spatial heterogeneity of mesopredator release within an oceanic Island system. Proceedings of the National Academy of Sciences 104, 20862–20865. 10.1073/pnas.0707414105 PMC2409232 18083843

[brv70124-bib-0578] Read, J. L. , Firn, J. , Grice, A. C. , Murphy, R. , Ryan‐Colton, E. & Schlesinger, C. A. (2020). Ranking buffel: comparative risk and mitigation costs of key environmental and socio‐cultural threats in central Australia. Ecology and Evolution 10, 12745–12763. 33304491 10.1002/ece3.6724 PMC7713970

[brv70124-bib-0579] Reaser, J. K. , Meyerson, L. A. , Cronk, Q. , De Poorter, M. A. J. , Eldrege, L. G. , Green, E. , Kairo, M. , Latasi, P. , Mack, R. N. , Mauremootoo, J. , O'Dowd, D. , Orapa, W. , Sastroutomo, S. , Saunders, A. , Shine, C. , *et al*. (2007). Ecological and socioeconomic impacts of invasive alien species in Island ecosystems. Environmental Conservation 34, 98–111.

[brv70124-bib-0580] Reed, T. E. , Kane, A. , McGinnity, P. & O'Sullivan, R. J. (2024). Competitive interactions affect introgression and population viability amidst maladaptive hybridization. Evolutionary Applications 17, e13746. 38957310 10.1111/eva.13746 PMC11217556

[brv70124-bib-0581] Régnier, C. , Fontaine, B. & Bouchet, P. (2009). Not knowing, not recording, not listing: numerous unnoticed mollusk extinctions. Conservation Biology 23, 1214–1221. 19459894 10.1111/j.1523-1739.2009.01245.x

[brv70124-bib-0582] * Reino, L. , Figueira, R. , Beja, P. , Araújo, M. B. , Capinha, C. & Strubbe, D. (2017). Networks of global bird invasion altered by regional trade ban. Science Advances 3, e1700783. 29181443 10.1126/sciadv.1700783 PMC5699901

[brv70124-bib-0583] Reo, N. J. & Ogden, L. A. (2018). Anishnaabe Aki: an indigenous perspective on the global threat of invasive species. Sustainability Science 13, 1443–1452.

[brv70124-bib-0584] Revilla, T. (2002). Effects of intraguild predation on resource competition. Journal of Theoretical Biology 214, 49–62. 11786031 10.1006/jtbi.2001.2448

[brv70124-bib-0585] * Reynolds, S. A. , Beery, S. , Burgess, N. , Burgman, M. , Butchart, S. H. M. , Cooke, S. J. , Coomes, D. , Danielsen, F. , Di Minin, E. & Durán, A. P. (2025). The potential for AI to revolutionize conservation: a horizon scan. Trends in Ecology & Evolution 40, 191–207. 39694720 10.1016/j.tree.2024.11.013

[brv70124-bib-0587] Ribeiro‐Silva, L. , Perrella, D. F. , Biagolini‐Jr, C. H. , Zima, P. V. Q. , Piratelli, A. J. , Schlindwein, M. N. , Galetti Junior, P. M. & Francisco, M. R. (2018). Testing camera traps as a potential tool for detecting nest predation of birds in a tropical rainforest environment. Zoologia (Curitiba) 35, e14678.

[brv70124-bib-0588] Ricciardi, A. (2001). Facilitative interactions among aquatic invaders: is an “invasional meltdown” occurring in the Great Lakes? Canadian Journal of Fisheries and Aquatic Sciences 58, 2513–2525.

[brv70124-bib-0589] Ricciardi, A. (2003). Predicting the impacts of an introduced species from its invasion history: an empirical approach applied to zebra mussel invasions. Freshwater Biology 48, 972–981.

[brv70124-bib-0590] Ricciardi, A. & Cohen, J. (2007). The invasiveness of an introduced species does not predict its impact. Biological Invasions 9, 309–315.

[brv70124-bib-0591] Ricciardi, A. , Hoopes, M. F. , Marchetti, M. P. & Lockwood, J. L. (2013). Progress toward understanding the ecological impacts of nonnative species. Ecological Monographs 83, 263–282.

[brv70124-bib-0592] Ricciardi, A. , Iacarella, J. C. , Aldridge, D. C. , Blackburn, T. M. , Carlton, J. T. , Catford, J. A. , Dick, J. T. A. , Hulme, P. E. , Jeschke, J. M. , Liebhold, A. M. , Lockwood, J. L. , MacIsaac, H. J. , Meyerson, L. A. , Pyšek, P. , Richardson, D. M. , *et al*. (2021). Four priority areas to advance invasion science in the face of rapid environmental change. Environmental Reviews 29, 119–141.

[brv70124-bib-0593] Ricciardi, A. & MacIsaac, H. J. (2008). The Book that Began Invasion Ecology. Nature 452, 34–36.

[brv70124-bib-0594] Ricciardi, A. & Ryan, R. (2018). Invasive species denialism revisited: response to Sagoff. Biological Invasions 20, 2731–2738.

[brv70124-bib-0595] Richardson, D. M. & Higgins, S. I. (2000). Pines as invaders in the southern hemisphere. In Ecology and Biogeography of Pinus (ed. D. M. Richardson ), pp. 450–473. Cambridge University Press, Cambridge.

[brv70124-bib-0596] Richardson, D. M. & Pyšek, P. (2008). Fifty years of invasion ecology–the legacy of Charles Elton. Diversity and Distributions 14, 161–168.

[brv70124-bib-0597] Riley, C. V. (1887). The Icerya, or Fluted Scale: Otherwise Known as the Cottony Cushion‐Scale. US Government Printing Office, Washington, DC.

[brv70124-bib-0598] Rilov, G. (2016). Multi‐species collapses at the warm edge of a warming sea. Scientific Reports 6, 36897. 27853237 10.1038/srep36897 PMC5113072

[brv70124-bib-0599] Rilov, G. , Canning‐Clode, J. & Guy‐Haim, T. (2024). Ecological impacts of invasive ecosystem engineers: a global perspective across terrestrial and aquatic systems. Functional Ecology 38, 37–51.

[brv70124-bib-0600] Rio‐Hortega, L. , Martín‐Forés, I. , Castro, I. , de Miguel, J. M. & Acosta Gallo, B. (2022). Network‐based analysis reveals differences in plant assembly between the native and the invaded ranges. NeoBiota 72, 157–181.

[brv70124-bib-0601] Rius, M. & Darling, J. A. (2014). How important is intraspecific genetic admixture to the success of colonising populations? Trends in Ecology & Evolution 29, 233–242. 24636862 10.1016/j.tree.2014.02.003

[brv70124-bib-0602] Roberts, J. , Florentine, S. , Fernando, W. G. D. & Tennakoon, K. U. (2022). Achievements, developments and future challenges in the field of bioherbicides for weed control: a global review. Plants 11, 2242. 36079623 10.3390/plants11172242 PMC9460325

[brv70124-bib-0603] Robertson, B. A. & Chalfoun, A. D. (2016). Evolutionary traps as keys to understanding behavioral maladapation. Current Opinion in Behavioral Sciences 12, 12–17.

[brv70124-bib-0604] Robertson, P. A. , Mill, A. C. , Adriaens, T. , Moore, N. , Vanderhoeven, S. , Essl, F. & Booy, O. (2021). Risk management assessment improves the cost‐effectiveness of invasive species prioritisation. Biology 10, 1320. 34943234 10.3390/biology10121320 PMC8698869

[brv70124-bib-0605] Rogers, H. S. , Buhle, E. R. , HilleRisLambers, J. , Fricke, E. C. , Miller, R. H. & Tewksbury, J. J. (2017). Effects of an invasive predator cascade to plants via mutualism disruption. Nature Communications 8, 14557. 10.1038/ncomms14557 PMC5344968 28270682

[brv70124-bib-0606] Rogosch, J. S. & Olden, J. D. (2020). Invaders induce coordinated isotopic niche shifts in native fish species. Canadian Journal of Fisheries and Aquatic Sciences 77, 1348–1358.

[brv70124-bib-0607] Romi, R. , Boccolini, D. , di Luca, M. , Medlock, J. M. , Schaffner, F. , Severini, F. & Toma, L. (2018). The invasive mosquitoes of medical importance. In Invasive Species and Human Health, pp. 76–90. CAB International, Wallingford, Oxfordshire, UK.

[brv70124-bib-0608] Rosnow, R. L. & Rosenthal, R. (1992). Statistical procedures and the justification of knowledge in psychological science. In Methodological Issues & Strategies in Clinical Research (ed. A. E. Kazdin ), pp. 295–314. American Psychological Association, Washington, DC.

[brv70124-bib-0609] Rotherham, I. D. (2021). The impacts of recolonisation of an urbanised river by native and non‐native species. Frontiers in Ecology and Evolution 9, 618371.

[brv70124-bib-0610] Rouget, M. , Robertson, M. P. , Wilson, J. R. U. , Hui, C. , Essl, F. , Renteria, J. L. & Richardson, D. M. (2016). Invasion debt–quantifying future biological invasions. Diversity and Distributions 22, 445–456.

[brv70124-bib-0611] Roy, H. , Pauchard, A. , Stoett, P. , Renard Truong, T. , Bacher, S. , Galil, B. , Hulme, P. , Ikeda, T. , Sankaran, K. V. , McGeoch, M. , Meyerson, L. , Nunez, M. , Ordonez, A. , Rahlao, S. , Schwindt, E. , *et al*. (2023 *a*). Summary for policymakers of the thematic assessment report on invasive alien species and their control. In IPBES Invasive alien species assessment pp. 1–56. IPBES Secretariat, Bonn, Germany.

[brv70124-bib-0612] Roy, H. E. , Bacher, S. , Essl, F. , Adriaens, T. , Aldridge, D. C. , Bishop, J. D. D. , Blackburn, T. M. , Branquart, E. , Brodie, J. , Carboneras, C. , Cottier‐Cook, E. J. , Copp, G. H. , Dean, H. J. , Eilenberg, J. , Gallardo, B. , *et al*. (2019). Developing a list of invasive alien species likely to threaten biodiversity and ecosystems in the European Union. Global Change Biology 25, 1032–1048. 30548757 10.1111/gcb.14527 PMC7380041

[brv70124-bib-0613] Roy, H. E. , Hesketh, H. , Purse, B. V. , Eilenberg, J. , Santini, A. , Scalera, R. , Stentiford, G. D. , Adriaens, T. , Bacela‐Spychalska, K. , Bass, D. , Beckmann, K. M. , Bessell, P. , Bojko, J. , Booy, O. , Cardoso, A. C. , *et al*. (2017). Alien pathogens on the horizon: opportunities for predicting their threat to wildlife. Conservation Letters 10, 477–484.

[brv70124-bib-0614] Roy, H. E. , Lawson Handley, L.‐J. , Schönrogge, K. , Poland, R. L. & Purse, B. V. (2011). Can the enemy release hypothesis explain the success of invasive alien predators and parasitoids? BioControl 56, 451–468.

[brv70124-bib-0615] Roy, H. E. , Pauchard, A. , Stoett, P. & Renard Truong, T. (2023 *b*). IPBES. Thematic Assessment Report on Invasive Alien Species and their Control of the Intergovernmental Science‐Policy Platform on Biodiversity and Ecosystem Services. Bonn, Germany.

[brv70124-bib-0616] Roy, H. E. , Pauchard, A. , Stoett, P. , Truong, T. R. , Lipinskaya, T. , Vincete, J. R. , Bacher, S. , Bliss, C. , Bullock, J. M. , Camacho‐Cervantes, M. , Courchamp, F. , Egawa, C. , Foxcroft, L. C. , Galil, B. S. , Hiremath, A. J. , *et al*. (2023 *c*). IPBES Invasive alien species assessment: Chapter 1. Introducing biological invasions and the IPBES thematic assessment of invasive alien species and their control.

[brv70124-bib-0617] Roy, H. E. , Peyton, J. , Aldridge, D. C. , Bantock, T. , Blackburn, T. M. , Britton, R. , Clark, P. , Cook, E. , Dehnen‐Schmutz, K. , Dines, T. , Dobson, M. , Edwards, F. , Harrower, C. , Harvey, M. C. , Minchin, D. , *et al*. (2014). Horizon scanning for invasive alien species with the potential to threaten biodiversity in Great Britain. Global Change Biology 20, 3859–3871. 24839235 10.1111/gcb.12603 PMC4283593

[brv70124-bib-0618] Roy, H. E. , Rabitsch, W. , Scalera, R. , Stewart, A. , Gallardo, B. , Genovesi, P. , Essl, F. , Adriaens, T. , Bacher, S. , Booy, O. , Branquart, E. , Brunel, S. , Copp, G. H. , Dean, H. , D'hondt, B. J. , *et al*. (2018). Developing a framework of minimum standards for the risk assessment of alien species. Journal of Applied Ecology 55, 526–538.

[brv70124-bib-0619] Ruesink, J. L. , Feist, B. E. , Harvey, C. J. , Hong, J. S. , Trimble, A. C. & Wisehart, L. M. (2006). Changes in productivity associated with four introduced species: ecosystem transformation of a ‘pristine'estuary. Marine Ecology Progress Series 311, 203–215.

[brv70124-bib-0620] Ruesink, J. L. , Lenihan, H. S. , Trimble, A. C. , Heiman, K. W. , Micheli, F. , Byers, J. E. & Kay, M. C. (2005). Introduction of non‐native oysters: ecosystem effects and restoration implications. Annual Review of Ecology, Evolution, and Systematics 36, 643–689.

[brv70124-bib-0621] Ruland, F. & Jeschke, J. M. (2020). How biological invasions affect animal behaviour: a global, cross‐taxonomic analysis. Journal of Animal Ecology 89, 2531–2541. 32745238 10.1111/1365-2656.13306

[brv70124-bib-0622] Rushton, S. P. , Lurz, P. W. W. , Gurnell, J. , Nettleton, P. , Bruemmer, C. , Shirley, M. D. F. & Sainsbury, A. (2006). Disease threats posed by alien species: the role of a poxvirus in the decline of the native red squirrel in Britain. Epidemiology & Infection 134, 521–533. 16238822 10.1017/S0950268805005303 PMC2870420

[brv70124-bib-0623] Russo, L. (2016). Positive and negative impacts of non‐native bee species around the world. Insects 7, 69. 27916802 10.3390/insects7040069 PMC5198217

[brv70124-bib-0624] Sala, E. , Kizilkaya, Z. , Yildirim, D. & Ballesteros, E. (2011). Alien marine fishes deplete algal biomass in the eastern Mediterranean. PLoS One 6, e17356. 21364943 10.1371/journal.pone.0017356 PMC3043076

[brv70124-bib-0625] Salguero‐Gómez, R. , Jones, O. R. , Archer, C. R. , Buckley, Y. M. , Che‐Castaldo, J. , Caswell, H. , Hodgson, D. , Scheuerlein, A. , Conde, D. A. , Brinks, E. , de Buhr, H. , Farack, C. , Gottschalk, F. , Hartmann, A. , Henning, A. , *et al*. (2015). The COMPADRE plant matrix database: an open online repository for plant demography. Journal of Ecology 103, 202–218.

[brv70124-bib-0626] Sandvik, H. , Sæther, B.‐E. , Holmern, T. , Tufto, J. , Engen, S. & Roy, H. E. (2013). Generic ecological impact assessments of alien species in Norway: a semi‐quantitative set of criteria. Biodiversity and Conservation 22, 37–62.

[brv70124-bib-0627] Santamaría, J. , Golo, R. , Verdura, J. , Tomas, F. , Ballesteros, E. , Alcoverro, T. , Arthur, R. & Cebrian, E. (2022). Learning takes time: biotic resistance by native herbivores increases through the invasion process. Ecology Letters 25, 2525–2539. 36209457 10.1111/ele.14115 PMC9828756

[brv70124-bib-0628] * Santini, A. & Migliorini, D. (2022). Invasive alien plant pathogens: the need of new detection methods. In Plant Pathology: Method and Protocols, pp. 111–118. Humana Press, New York, NY. 10.1007/978-1-0716-2517-0_7 35819601

[brv70124-bib-0629] * Santos, M. J. , Anderson, L. W. & Ustin, S. L. (2011). Effects of invasive species on plant communities: an example using submersed aquatic plants at the regional scale. Biological Invasions 13, 443–457.

[brv70124-bib-0630] Sarabeev, V. , Balbuena, J. A. , Desdevises, Y. & Morand, S. (2022). Host‐parasite relationships in invasive species: macroecological framework. Biological Invasions 24, 2649–2664.

[brv70124-bib-0631] Savidge, J. A. (1987). Extinction of an Island forest avifauna by an introduced snake. Ecology 68, 660–668.

[brv70124-bib-0632] Savvides, P. , Louca, V. & Sfenthourakis, S. (2015). Competition for shelter occupancy between a native freshwater crab and an invasive crayfish. Aquatic Ecology 49, 273–278.

[brv70124-bib-0633] Sax, D. F. & Gaines, S. D. (2008). Species invasions and extinction: the future of native biodiversity on islands. Proceedings of the National Academy of Sciences 105, 11490–11497. 10.1073/pnas.0802290105 PMC2556416 18695231

[brv70124-bib-0634] Sax, D. F. , Schlaepfer, M. A. & Olden, J. D. (2022). Valuing the contributions of non‐native species to people and nature. Trends in Ecology & Evolution 37, 1058–1066. 36210286 10.1016/j.tree.2022.08.005

[brv70124-bib-0635] Scalera, R. (2010). How much is Europe spending on invasive alien species? Biological Invasions 12, 173–177.

[brv70124-bib-0636] Scheele, B. C. , Pasmans, F. , Skerratt, L. F. , Berger, L. , Martel, A. N. , Beukema, W. , Acevedo, A. A. , Burrowes, P. A. , Carvalho, T. , Catenazzi, A. , De la Riva, I. , Fisher, M. C. , Flechas, S. V. , Foster, C. N. , Frías‐Álvarez, P. , *et al*. (2019). Amphibian fungal panzootic causes catastrophic and ongoing loss of biodiversity. Science 363, 1459–1463. 30923224 10.1126/science.aav0379

[brv70124-bib-0637] Schickele, A. , Guidetti, P. , Giakoumi, S. , Zenetos, A. , Francour, P. & Raybaud, V. (2021). Improving predictions of invasive fish ranges combining functional and ecological traits with environmental suitability under climate change scenarios. Global Change Biology 27, 6086–6102. 34543498 10.1111/gcb.15896

[brv70124-bib-0638] Schlaepfer, M. A. , Sax, D. F. & Olden, J. D. (2011). The potential conservation value of non‐native species. Conservation Biology 25, 428–437. 21342267 10.1111/j.1523-1739.2010.01646.x

[brv70124-bib-0639] Schrader, G. , MacLeod, A. , Petter, F. , Baker, R. H. A. , Brunel, S. , Holt, J. , Leach, A. W. & Mumford, J. D. (2012). Consistency in pest risk analysis–how can it be achieved and what are the benefits? EPPO Bulletin 42, 3–12.

[brv70124-bib-0640] Schreiber, B. , Petrenz, M. , Monka, J. , Drozd, B. , Hollert, H. & Schulz, R. (2017). Weatherfish (*Misgurnus fossilis*) as a new species for toxicity testing? Aquatic Toxicology 183, 46–53. 27992775 10.1016/j.aquatox.2016.12.006

[brv70124-bib-0641] Schreiner, I. H. & Nafus, D. M. (1993). Population increases of native moths following biological control of an introduced pest moth. Micronesica 4, 49–56.

[brv70124-bib-0642] Schwindt, E. , Iribarne, O. O. & Isla, F. I. (2004). Physical effects of an invading reef‐building polychaete on an Argentinean estuarine environment. Estuarine, Coastal and Shelf Science 59, 109–120.

[brv70124-bib-0643] Seebens, H. , Blackburn, T. M. , Dyer, E. E. , Genovesi, P. , Hulme, P. E. , Jeschke, J. M. , Pagad, S. , Pyšek, P. , Winter, M. , Arianoutsou, M. , Bacher, S. , Blasius, B. , Brundu, G. , Capinha, C. , Celesti‐Grapow, L. , *et al*. (2017). No saturation in the accumulation of alien species worldwide. Nature Communications 8, 14435. 10.1038/ncomms14435 PMC5316856 28198420

[brv70124-bib-0644] Seehausen, O. L. E. , Takimoto, G. , Roy, D. & Jokela, J. (2008). Speciation reversal and biodiversity dynamics with hybridization in changing environments. Molecular Ecology 17, 30–44. 18034800 10.1111/j.1365-294X.2007.03529.x

[brv70124-bib-0645] Semenya, S. S. , Potgieter, M. J. & Erasmus, L. J. C. (2013). Exotic and indigenous problem plants species used, by the Bapedi, to treat sexually transmitted infections in Limpopo Province, South Africa. African Health Sciences 13, 320–326. 24235930 10.4314/ahs.v13i2.17 PMC3824514

[brv70124-bib-0646] Sergio, F. , Marchesi, L. & Pedrini, P. (2003). Spatial refugia and the coexistence of a diurnal raptor with its intraguild owl predator. Journal of Animal Ecology 72, 232–245.

[brv70124-bib-0647] Settle, W. H. & Wilson, L. T. (1990). Invasion by the variegated leafhopper and biotic interactions: parasitism, competition, and apparent competition. Ecology 71, 1461–1470.

[brv70124-bib-0648] Shackleton, R. T. , Adriaens, T. , Brundu, G. , Dehnen‐Schmutz, K. , Estévez, R. A. , Fried, J. , Larson, B. M. H. , Liu, S. , Marchante, E. , Marchante, H. , Marchante, H. , Moshobane, M. C. , Novoa, A. , Reed, M. & Richardson, D. M. (2019 *a*). Stakeholder engagement in the study and management of invasive alien species. Journal of Environmental Management 229, 88–101. 30077401 10.1016/j.jenvman.2018.04.044

[brv70124-bib-0649] Shackleton, R. T. , Larson, B. M. H. , Novoa, A. , Richardson, D. M. & Kull, C. A. (2019 *b*). The human and social dimensions of invasion science and management. Journal of Environmental Management 229, 1–9. 30172420 10.1016/j.jenvman.2018.08.041

[brv70124-bib-0650] Shackleton, R. T. , Le Maitre, D. C. , Pasiecznik, N. M. & Richardson, D. M. (2014). Prosopis: a global assessment of the biogeography, benefits, impacts and management of one of the world's worst woody invasive plant taxa. AoB Plants 6, plu027. 24899150 10.1093/aobpla/plu027 PMC4086457

[brv70124-bib-0651] Shackleton, R. T. , Richardson, D. M. , Shackleton, C. M. , Bennett, B. , Crowley, S. L. , Dehnen‐Schmutz, K. , Estévez, R. A. , Fischer, A. , Kueffer, C. , Kull, C. A. , Marchante, E. , Novoa, A. , Potgieter, L. J. , Vaas, J. , Vaz, A. S. , *et al*. (2019 *c*). Explaining people's perceptions of invasive alien species: a conceptual framework. Journal of Environmental Management 229, 10–26. 30077400 10.1016/j.jenvman.2018.04.045

[brv70124-bib-0652] Sharifian‐Fard, M. , Pasmans, F. , Adriaensen, C. , Devisscher, S. , Adriaens, T. , Louette, G. & Martel, A. (2011). Ranavirosis in invasive bullfrogs, Belgium. Emerging Infectious Diseases 17, 2371. 22172553 10.3201/eid1712.110236 PMC3311206

[brv70124-bib-0653] Shechonge, A. , Ngatunga, B. P. , Tamatamah, R. , Bradbeer, S. J. , Harrington, J. , Ford, A. G. P. , Turner, G. F. & Genner, M. J. (2018). Losing cichlid fish biodiversity: genetic and morphological homogenization of tilapia following colonization by introduced species. Conservation Genetics 19, 1199–1209. 30363773 10.1007/s10592-018-1088-1 PMC6182432

[brv70124-bib-0654] * Shen, L. , LaRue, E. , Fei, S. & Zhang, H. (2024). Spatial prediction of plant invasion using a hybrid of machine learning and geostatistical method. Ecology and Evolution 14, e11605. 38932949 10.1002/ece3.11605 PMC11199124

[brv70124-bib-0655] Shine, C. , Williams, N. & Gündling, L. (2000). A Guide to Designing Legal and Institutional Frameworks on Alien Invasive Species. IUCN – The World Conservation Union, Gland, Switzerland.

[brv70124-bib-0656] Shine, R. (2010). The ecological impact of invasive cane toads (*Bufo marinus*) in Australia. The Quarterly Review of Biology 85, 253–291. 20919631 10.1086/655116

[brv70124-bib-0657] Shine, R. (2012). Invasive species as drivers of evolutionary change: cane toads in tropical Australia. Evolutionary Applications 5, 107–116. 25568034 10.1111/j.1752-4571.2011.00201.x PMC3353345

[brv70124-bib-0658] Sih, A. , Bolnick, D. I. , Luttbeg, B. , Orrock, J. L. , Peacor, S. D. , Pintor, L. M. , Preisser, E. , Rehage, J. S. & Vonesh, J. R. (2010). Predator–prey naïveté, antipredator behavior, and the ecology of predator invasions. Oikos 119, 610–621.

[brv70124-bib-0659] Sih, A. , Ferrari, M. C. O. & Harris, D. J. (2011). Evolution and behavioural responses to human‐induced rapid environmental change. Evolutionary Applications 4, 367–387. 25567979 10.1111/j.1752-4571.2010.00166.x PMC3352552

[brv70124-bib-0660] Silva, Z. L. d. (2022). The representations, the meanings and the “beat” of Bumba‐meu‐Boi festivities, from Maranhão, and Boi‐Bumbá festivities, from middle Amazonas state and Parintins. Advances in Social Sciences Research Journal 9, 706–732.

[brv70124-bib-0661] * Silvestro, D. , Goria, S. , Sterner, T. & Antonelli, A. (2022). Improving biodiversity protection through artificial intelligence. Nature Sustainability 5, 415–424. 10.1038/s41893-022-00851-6 PMC7612764 35614933

[brv70124-bib-0662] Simberloff, D. (2006). Risk assessments, blacklists, and white lists for introduced species: are predictions good enough to be useful? Agricultural and Resource Economics Review 35, 1–10.

[brv70124-bib-0663] Simberloff, D. , Bortolus, A. , Carlton, J. T. , Courchamp, F. , Cuthbert, R. N. , Hulme, P. E. , Lockwood, J. L. , Meyerson, L. A. , Nuñez, M. A. , Ricciardi, A. , Richardson, D. M. & Schwindt, E. (2024). Systematic and persistent bias against invasion science: framing conservation scientists. Bioscience 74, 312–314.

[brv70124-bib-0664] Simberloff, D. , Martin, J.‐L. , Genovesi, P. , Maris, V. , Wardle, D. A. , Aronson, J. , Courchamp, F. , Galil, B. , García‐Berthou, E. , Pascal, M. , Pyšek, P. , Sousa, R. , Tabacchi, E. & Vilà, M. (2013). Impacts of biological invasions: what's what and the way forward. Trends in Ecology & Evolution 28, 58–66. 22889499 10.1016/j.tree.2012.07.013

[brv70124-bib-0665] Simberloff, D. & Meyerson, L. A. (2024). Yet another call for the end of invasion biology. Biological Invasions 26, 3975–3978.

[brv70124-bib-0666] Simberloff, D. & Von Holle, B. (1999). Positive interactions of nonindigenous species: invasional meltdown? Biological Invasions 1, 21–32.

[brv70124-bib-0667] Sinclair, J. S. , Lockwood, J. L. , Hasnain, S. , Cassey, P. & Arnott, S. E. (2020). A framework for predicting which non‐native individuals and species will enter, survive, and exit human‐mediated transport. Biological Invasions 22, 217–231.

[brv70124-bib-0668] Singh, A. K. & Lakra, W. S. (2011). Risk and benefit assessment of alien fish species of the aquaculture and aquarium trade into India. Reviews in Aquaculture 3, 3–18.

[brv70124-bib-0669] Siqueira, B. V. L. , Sakuragui, C. M. , Soares, B. E. & de Oliveira, D. R. (2018). The rise of medicalization of plants in Brazil: a temporal perspective on vernacular names. Journal of Ethnopharmacology 224, 535–540. 29933011 10.1016/j.jep.2018.06.024

[brv70124-bib-0670] Snyder, W. E. & Evans, E. W. (2006). Ecological effects of invasive arthropod generalist predators. Annual Review of Ecology, Evolution, and Systematics 37, 95–122.

[brv70124-bib-0671] Sobral, F. L. , Lees, A. C. & Cianciaruso, M. V. (2016). Introductions do not compensate for functional and phylogenetic losses following extinctions in insular bird assemblages. Ecology Letters 19, 1091–1100. 27353518 10.1111/ele.12646

[brv70124-bib-0672] Sofaer, H. R. , Jarnevich, C. S. & Pearse, I. S. (2018). The relationship between invader abundance and impact. Ecosphere 9, e02415.

[brv70124-bib-0673] Soga, M. , Gaston, K. J. , Fukano, Y. & Evans, M. J. (2023). The vicious cycle of biophobia. Trends in Ecology & Evolution 38, 512–520. 36707258 10.1016/j.tree.2022.12.012

[brv70124-bib-0674] Sol, D. , Griffin, A. S. , Bartomeus, I. & Boyce, H. (2011). Exploring or avoiding novel food resources? The novelty conflict in an invasive bird. PLoS One 6, e19535. 21611168 10.1371/journal.pone.0019535 PMC3097186

[brv70124-bib-0675] Soliman, T. , Mourits, M. C. M. , Oude Lansink, A. G. J. M. & van der Werf, W. (2010). Economic impact assessment in pest risk analysis. Crop Protection 29, 517–524.

[brv70124-bib-0676] Soto, I. , Ahmed, D. A. , Balzani, P. , Cuthbert, R. N. & Haubrock, P. J. (2023 *a*). Sigmoidal curves reflect impacts and dynamics of aquatic invasive species. Science of the Total Environment 872, 161818. 36801313 10.1016/j.scitotenv.2023.161818

[brv70124-bib-0677] Soto, I. , Ahmed, D. A. , Beidas, A. , Oficialdegui, F. J. , Tricarico, E. , Angeler, D. G. , Amatulli, G. , Briski, E. , Datry, T. , Dohet, A. , Domisch, S. , England, J. , Feio, M. J. , Forcellini, M. , Johnson, R. K. , *et al*. (2023 *b*). Long‐term trends in crayfish invasions across European rivers. Science of the Total Environment 867, 161537. 36640879 10.1016/j.scitotenv.2023.161537

[brv70124-bib-0678] Soto, I. , Balzani, P. , Carneiro, L. , Cuthbert, R. N. , Macêdo, R. , Serhan Tarkan, A. , Ahmed, D. A. , Bang, A. , Bacela‐Spychalska, K. , Bailey, S. A. , Baudry, T. , Ballesteros‐Mejia, L. , Bortolus, A. , Briski, E. , Britton, J. R. , *et al*. (2024 *a*). Taming the terminological tempest in invasion science. Biological Reviews 99, 1357–1390. 38500298 10.1111/brv.13071

[brv70124-bib-0679] Soto, I. , Balzani, P. , Oficialdegui, F. J. , Molinero, C. , Kouba, A. , Ahmed, D. A. , Turbelin, A. J. , Hudgins, E. J. , Bodey, T. W. , Gojery, S. A. , Courchamp, F. , Cuthbert, R. N. & Haubrock, P. J. (2024 *b*). The wild cost of invasive feral animals worldwide. Science of the Total Environment 912, 169281. 38101642 10.1016/j.scitotenv.2023.169281

[brv70124-bib-0680] Soto, I. , Courtois, P. , Pili, A. , Tordoni, E. , Manfrini, É. , Angulo, E. , Bellard, C. , Briski, E. , Buřič, M. , Cuthbert, R. N. , Kouba, A. , Kourantidou, M. , Macêdo, R. L. , Leroy, B. , Haubrock, P. J. , *et al*. (2025). Using species ranges and macroeconomic data to fill the gap in costs of biological invasions. Nature Ecology & Evolution 9, 1021–1030. 40419738 10.1038/s41559-025-02697-5

[brv70124-bib-0681] Soto, I. , Cuthbert, R. N. , Kouba, A. , Capinha, C. , Turbelin, A. , Hudgins, E. J. , Diagne, C. , Courchamp, F. & Haubrock, P. J. (2022). Global economic costs of herpetofauna invasions. Scientific Reports 12, 10829. 35902706 10.1038/s41598-022-15079-9 PMC9334389

[brv70124-bib-0682] Soto, I. , Cuthbert, R. N. , Ricciardi, A. , Ahmed, D. A. , Altermatt, F. , Schäfer, R. B. , Archambaud‐Suard, G. , Bonada, N. , Cañedo‐Argüelles, M. , Csabai, Z. , Datry, T. , Dick, J. T. A. , Floury, M. , Forio, M. A. E. , Forcellini, M. , *et al*. (2023 *c*). The faunal Ponto‐Caspianization of central and western European waterways. Biological Invasions 25, 2613–2629.

[brv70124-bib-0683] Soto, I. , Haubrock, P. J. , Cuthbert, R. N. , Renault, D. , Probert, A. F. & Tarkan, A. S. (2023 *d*). Monetary impacts should be considered in biological invasion risk assessments. Journal of Applied Ecology 60, 2309–2313.

[brv70124-bib-0684] Soto, I. , Macêdo, R. L. , Carneiro, L. , Briski, E. , Kouba, A. , Cuthbert, R. N. & Haubrock, P. J. (2024 *c*). Divergent temporal responses of native macroinvertebrate communities to biological invasions. Global Change Biology 30, e17521. 39344526 10.1111/gcb.17521

[brv70124-bib-0685] Sousa, R. , Gutiérrez, J. L. & Aldridge, D. C. (2009). Non‐indigenous invasive bivalves as ecosystem engineers. Biological Invasions 11, 2367–2385.

[brv70124-bib-0686] Sousa, R. , Morais, P. , Dias, E. & Antunes, C. (2011). Biological invasions and ecosystem functioning: time to merge. Biological Invasions 13, 1055–1058.

[brv70124-bib-0687] Sousa, R. , Nogueira, A. J. A. , Gaspar, M. B. , Antunes, C. & Guilhermino, L. (2008). Growth and extremely high production of the non‐indigenous invasive species *Corbicula fluminea* (Müller, 1774): possible implications for ecosystem functioning. Estuarine, Coastal and Shelf Science 80, 289–295.

[brv70124-bib-0688] Sousa, R. , Nogueira, J. G. , Ferreira, A. , Carvalho, F. , Lopes‐Lima, M. , Varandas, S. & Teixeira, A. (2019). A tale of shells and claws: the signal crayfish as a threat to the pearl mussel *Margaritifera margaritifera* in Europe. Science of the Total Environment 665, 329–337. 30772562 10.1016/j.scitotenv.2019.02.094

[brv70124-bib-0689] Sousa, R. , Nogueira, J. G. & Padilha, J. (2024). Moving from the species to the population level in biological invasions. Global Change Biology 30, e17396. 38958102 10.1111/gcb.17396

[brv70124-bib-0690] South, J. , Dickey, J. W. E. , Cuthbert, R. N. & Dick, J. T. A. (2022). Combining resource population dynamics into impact assessments of native and invasive species under abiotic change. Ecological Indicators 142, 109260.

[brv70124-bib-0691] South, P. M. , Lilley, S. A. , Tait, L. W. , Alestra, T. , Hickford, M. J. H. , Thomsen, M. S. & Schiel, D. R. (2015). Transient effects of an invasive kelp on the community structure and primary productivity of an intertidal assemblage. Marine and Freshwater Research 67, 103–112.

[brv70124-bib-0692] Spear, M. J. , Walsh, J. R. , Ricciardi, A. & Zanden, M. J. V. (2021). The invasion ecology of sleeper populations: prevalence, persistence, and abrupt shifts. Bioscience 71, 357–369.

[brv70124-bib-0693] Spinage, C. (2012). Cattle Plague: A History. Springer Science & Business Media, Berlin, Germany.

[brv70124-bib-0694] Srėbalienė, G. , Olenin, S. , Minchin, D. & Narščius, A. (2019). A comparison of impact and risk assessment methods based on the IMO guidelines and EU invasive alien species risk assessment frameworks. PeerJ 7, e6965. 31218119 10.7717/peerj.6965 PMC6563794

[brv70124-bib-0695] Stachowicz, J. J. , Whitlatch, R. B. & Osman, R. W. (1999). Species diversity and invasion resistance in a marine ecosystem. Science 286, 1577–1579. 10567267 10.1126/science.286.5444.1577

[brv70124-bib-0696] Stellati, L. , Borgianni, N. , Bissattini, A. M. , Buono, V. , Haubrock, P. J. , Balzani, P. , Tricarico, E. , Inghilesi, A. F. , Tancioni, L. , Martinoli, M. , Luiselli, L. & Vignoli, L. (2019). Living with aliens: suboptimal ecological condition in semiaquatic snakes inhabiting a hot spot of allodiversity. Acta Oecologica 100, 103466.

[brv70124-bib-0697] Stepp, J. R. & Moerman, D. E. (2001). The importance of weeds in ethnopharmacology. Journal of Ethnopharmacology 75, 19–23. 11282438 10.1016/s0378-8741(00)00385-8

[brv70124-bib-0698] Strayer, D. L. (2012). Eight questions about invasions and ecosystem functioning. Ecology Letters 15, 1199–1210. 22694728 10.1111/j.1461-0248.2012.01817.x

[brv70124-bib-0699] Strayer, D. L. , Caraco, N. F. , Cole, J. J. , Findlay, S. & Pace, M. L. (1999). Transformation of freshwater ecosystems by bivalves: a case study of zebra mussels in the Hudson River. Bioscience 49, 19–27.

[brv70124-bib-0700] Strayer, D. L. , D'Antonio, C. M. , Essl, F. , Fowler, M. S. , Geist, J. , Hilt, S. , Jarić, I. , Jöhnk, K. , Jones, C. G. , Lambin, X. , Latzka, A. W. , Pergl, J. , Pyšek, P. , von Robertson, P. , Schmalensee, M. , *et al*. (2017). Boom‐bust dynamics in biological invasions: towards an improved application of the concept. Ecology Letters 20, 1337–1350. 28834087 10.1111/ele.12822

[brv70124-bib-0701] Strayer, D. L. , Eviner, V. T. , Jeschke, J. M. & Pace, M. L. (2006). Understanding the long‐term effects of species invasions. Trends in Ecology & Evolution 21, 645–651. 16859805 10.1016/j.tree.2006.07.007

[brv70124-bib-0702] Stringham, O. C. & Lockwood, J. L. (2018). Pet problems: biological and economic factors that influence the release of alien reptiles and amphibians by pet owners. Journal of Applied Ecology 55, 2632–2640.

[brv70124-bib-0703] Strive, T. & Cox, T. E. (2019). Lethal biological control of rabbits–the most powerful tools for landscape‐scale mitigation of rabbit impacts in Australia. Australian Zoologist 40, 118–128.

[brv70124-bib-0704] Subalusky, A. L. , Sethi, S. A. , Anderson, E. P. , Jiménez, G. , Echeverri‐Lopez, D. , García‐Restrepo, S. , Nova‐León, L. J. , Reátiga‐Parrish, J. F. , Post, D. M. & Rojas, A. (2023). Rapid population growth and high management costs have created a narrow window for control of introduced hippos in Colombia. Scientific Reports 13, 6193. 37062768 10.1038/s41598-023-33028-y PMC10106455

[brv70124-bib-0705] Subramaniam, K. , Behringer, D. C. , Bojko, J. , Yutin, N. , Clark, A. S. , Bateman, K. S. , van Aerle, R. , Bass, D. , Kerr, R. C. , Koonin, E. V. , Stentiford, G. D. & Waltzek, T. B. (2020). A new family of DNA viruses causing disease in crustaceans from diverse aquatic biomes. MBio 11, 10–1128. 10.1128/mBio.02938-19 PMC6960288 31937645

[brv70124-bib-0706] Sun, Y. , Ren, Z. , Müller‐Schärer, H. , Callaway, R. M. , van Kleunen, M. & Huang, W. (2024). Increasing and fluctuating resource availability enhances invasional meltdown. Ecology 105, e4387. 39016245 10.1002/ecy.4387

[brv70124-bib-0707] Svoboda, J. , Mrugała, A. , Kozubíková‐Balcarová, E. & Petrusek, A. (2017). Hosts and transmission of the crayfish plague pathogen *Aphanomyces astaci*: a review. Journal of Fish Diseases 40, 127–140. 27111501 10.1111/jfd.12472

[brv70124-bib-0708] Szuroczki, D. & Richardson, J. M. L. (2011). Palatability of the larvae of three species of *Lithobates* . Herpetologica 67, 213–221.

[brv70124-bib-0709] Tablado, Z. , Tella, J. L. , Sánchez‐Zapata, J. A. & Hiraldo, F. (2010). The paradox of the long‐term positive effects of a north American crayfish on a European community of predators. Conservation Biology 24, 1230–1238. 20337679 10.1111/j.1523-1739.2010.01483.x

[brv70124-bib-0710] Taillie, P. J. , Hart, K. M. , Sovie, A. R. & McCleery, R. A. (2021). Native mammals lack resilience to invasive generalist predator. Biological Conservation 261, 109290.

[brv70124-bib-0711] Talley, T. S. , Crooks, J. A. & Levin, L. A. (2001). Habitat utilization and alteration by the invasive burrowing isopod, *Sphaeroma quoyanum*, in California salt marshes. Marine Biology 138, 561–573.

[brv70124-bib-0712] Tambo, J. A. , Kansiime, M. K. , Mugambi, I. , Agboyi, L. K. , Beseh, P. K. & Day, R. (2023). Economic impacts and management of fall armyworm (*Spodoptera frugiperda*) in smallholder agriculture: a panel data analysis for Ghana. CABI Agriculture and Bioscience 4, 38.

[brv70124-bib-0713] Tarkan, A. S. , Bayçelebi, E. , Giannetto, D. , Özden, E. D. , Yazlık, A. , Emiroğlu, Ö. , Aksu, S. , Uludağ, A. , Aksoy, N. , Baytaşoğlu, H. , Kaya, C. , Mutlu, T. , Kırankaya, Ş. G. , Ergüden, D. , Per, E. , *et al*. (2024 *a*). Economic costs of non‐native species in Türkiye: a first national synthesis. Journal of Environmental Management 358, 120779. 38599083 10.1016/j.jenvman.2024.120779

[brv70124-bib-0714] Tarkan, A. S. , Emiroğlu, Ö. , Aksu, S. , Kurtul, I. , Błońska, D. , Bayçelebi, E. , Soto, I. , Chan, S. S. , Haubrock, P. J. & Bradshaw, C. J. A. (2024 *b*). Testing the dispersal‐origin‐status‐impact (DOSI) scheme to prioritise non‐native and translocated species management. Scientific Reports 14, 31059. 39730848 10.1038/s41598-024-82284-z PMC11680831

[brv70124-bib-0715] Tarkan, A. S. , Yoğurtçuoğlu, B. , Ekmekçi, F. G. , Clarke, S. A. , Wood, L. E. , Vilizzi, L. & Copp, G. (2020). First application in Turkey of the European non‐native species in aquaculture risk analysis scheme to evaluate the farmed non‐native fish, striped catfish *Pangasianodon hypophthalmus* . Fisheries Management and Ecology 27, 123–131.

[brv70124-bib-0716] Tassin, J. & Kull, C. A. (2015). Facing the broader dimensions of biological invasions. Land Use Policy 42, 165–169.

[brv70124-bib-0717] Telfer, S. , Bown, K. J. , Sekules, R. , Begon, M. , Hayden, T. & Birtles, R. (2005). Disruption of a host‐parasite system following the introduction of an exotic host species. Parasitology 130, 661–668. 15977903 10.1017/s0031182005007250

[brv70124-bib-0718] Terborgh, J. & Estes, J. A. (2013). Trophic Cascades: Predators, Prey, and the Changing Dynamics of Nature. Island Press, Washington, DC.

[brv70124-bib-0719] Thieltges, D. W. , Strasser, M. & Reise, K. (2006). How bad are invaders in coastal waters? The case of the American slipper limpet *Crepidula fornicata* in western Europe. Biological Invasions 8, 1673–1680.

[brv70124-bib-0720] Thomas, J. R. , Robinson, C. V. , Mrugała, A. , Ellison, A. R. , Matthews, E. , Griffiths, S. W. , Consuegra, S. & Cable, J. (2020). Crayfish plague affects juvenile survival and adult behaviour of invasive signal crayfish. Parasitology 147, 706–714. 32046798 10.1017/S0031182020000165 PMC10317613

[brv70124-bib-0721] Thomsen, M. S. , Byers, J. E. , Schiel, D. R. , Bruno, J. F. , Olden, J. D. , Wernberg, T. & Silliman, B. R. (2014 *a*). Impacts of marine invaders on biodiversity depend on trophic position and functional similarity. Marine Ecology Progress Series 495, 39–47.

[brv70124-bib-0722] * Thomsen, M. , Wernberg, T. , Olden, J. , Byers, J. E. , Bruno, J. , Silliman, B. & Schiel, D. (2014b). Forty years of experiments on aquatic invasive species: are study biases limiting our understanding of impacts? NeoBiota 22, 1–22.

[brv70124-bib-0723] Todesco, M. , Pascual, M. A. , Owens, G. L. , Ostevik, K. L. , Moyers, B. T. , Hübner, S. , Heredia, S. M. , Hahn, M. A. , Caseys, C. , Bock, D. G. & Rieseberg, L. H. (2016). Hybridization and extinction. Evolutionary Applications 9, 892–908. 27468307 10.1111/eva.12367 PMC4947151

[brv70124-bib-0724] * Toland, E. , Bando, M. , Hamers, M. , Cadenas, V. , Laidlaw, R. , Martínez‐Silvestre, A. & van der Wielen, P. (2020). Turning negatives into positives for pet trading and keeping: a review of positive lists. Animals 10, 2371. 33322002 10.3390/ani10122371 PMC7763047

[brv70124-bib-0725] Tollington, S. , Turbe, A. , Rabitsch, W. , Groombridge, J. J. , Scalera, R. , Essl, F. & Shwartz, A. (2017). Making the EU legislation on invasive species a conservation success. Conservation Letters 10, 112–120.

[brv70124-bib-0726] Top, N. , Tarkan, A. S. , Vilizzi, L. & Karakuş, U. (2016). Microhabitat interactions of non‐native pumpkinseed *Lepomis gibbosus* in a Mediterranean‐type stream suggest no evidence for impact on endemic fishes. Knowledge & Management of Aquatic Ecosystems 417, 36.

[brv70124-bib-0727] Torchin, M. E. , Lafferty, K. D. , Dobson, A. P. , McKenzie, V. J. & Kuris, A. M. (2003). Introduced species and their missing parasites. Nature 421, 628–630. 12571595 10.1038/nature01346

[brv70124-bib-0728] Torchin, M. E. & Mitchell, C. E. (2004). Parasites, pathogens, and invasions by plants and animals. Frontiers in Ecology and the Environment 2, 183–190.

[brv70124-bib-0729] Trowbridge, C. D. (2004). Emerging associations on marine rocky shores: specialist herbivores on introduced macroalgae. Journal of Animal Ecology 73, 294–308.

[brv70124-bib-0730] Tsiamis, K. , Gervasini, E. , Deriu, I. , D'Amico, F. , Nunes, A. , Addamo, A. , Cardoso, A. C. , D'Amico, F. , Nunes, A. L. & Addamo, A. M. (2017). Baseline distribution of invasive alien species of union concern. Ispra (Italy): Publications Office of the European Union, Luxembourg, 1–96.

[brv70124-bib-0731] Tsirintanis, K. , Azzurro, E. , Crocetta, F. , Dimiza, M. , Froglia, C. , Gerovasileiou, V. , Langeneck, J. , Mancinelli, G. , Rosso, A. , Stern, N. , Triantaphyllou, M. , Tsiamis, K. , Turon, X. , Verlaque, M. , Zenetos, A. , *et al*. (2022). Bioinvasion impacts on biodiversity, ecosystem services, and human health in the Mediterranean Sea. Aquatic Invasions 17, 308–352.

[brv70124-bib-0732] Turbé, A. , Strubbe, D. , Mori, E. , Carrete, M. , Chiron, F. , Clergeau, P. , González‐Moreno, P. , Le Louarn, M. , Luna, A. , Menchetti, M. , Nentwig, W. , Pârâu, L. G. , Postigo, J.‐L. , Rabitsch, W. , Senar, J. C. , *et al*. (2017). Assessing the assessments: evaluation of four impact assessment protocols for invasive alien species. Diversity and Distributions 23, 297–307.

[brv70124-bib-0733] Turbelin, A. J. , Cuthbert, R. N. , Essl, F. , Haubrock, P. J. , Ricciardi, A. & Courchamp, F. (2023). Biological invasions are as costly as natural hazards. Perspectives in Ecology and Conservation 21, 143–150.

[brv70124-bib-0734] Turbelin, A. J. , Hudgins, E. J. , Catford, J. A. , Cuthbert, R. N. , Diagne, C. , Kourantidou, M. , Roiz, D. & Courchamp, F. (2024). Biological invasions as burdens to primary economic sectors. Global Environmental Change 87, 102858.

[brv70124-bib-0735] Twardochleb, L. A. , Olden, J. D. & Larson, E. R. (2013). A global meta‐analysis of the ecological impacts of nonnative crayfish. Freshwater Science 32, 1367–1382.

[brv70124-bib-0736] Tyagi, V. , Yadav, R. , Sukumaran, D. & Veer, V. (2015). Larvicidal activity of invasive weed *Prosopis juliflora* against mosquito species *Anopheles subpictus*, *Culex quinquefasciatus* and *Aedes aegypti* . International Journal of Applied Research 1, 285–288.

[brv70124-bib-0737] Ungureanu, E. , Mojžišová, M. , Tangerman, M. , Ion, M. C. , Pârvulescu, L. & Petrusek, A. (2020). The spatial distribution of Aphanomyces astaci genotypes across Europe: introducing the first data from Ukraine. Freshwater Crayfish 25, 77–87.

[brv70124-bib-0738] U.S. Department of the Interior (2021). U.S. Department of the Interior Invasive Species Strategic Plan, Fiscal Years 2021–2025. U.S. Department of the Interior, Washington, D.C.

[brv70124-bib-0739] Vaes‐Petignat, S. & Nentwig, W. (2014). Environmental and economic impact of alien terrestrial arthropods in Europe. NeoBiota 22, 23–42.

[brv70124-bib-0740] Van der Putten, W. H. , Klironomos, J. N. & Wardle, D. A. (2007). Microbial ecology of biological invasions. The ISME Journal 1, 28–37. 18043611 10.1038/ismej.2007.9

[brv70124-bib-0741] van der Veer, G. & Nentwig, W. (2015). Environmental and economic impact assessment of alien and invasive fish species in Europe using the generic impact scoring system. Ecology of Freshwater Fish 24, 646–656.

[brv70124-bib-0742] Van Wilgen, B. W. & Richardson, D. M. (1985). The effects of alien shrub invasions on vegetation structure and fire behaviour in South African fynbos shrublands: a simulation study. Journal of Applied Ecology 22, 955–966.

[brv70124-bib-0743] Vanderhoeven, S. , Adriaens, T. , D'hondt, B. , Van Gossum, H. , Vandegehuchte, M. , Verreycken, H. , Cigar, J. & Branquart, E. (2015). A science‐based approach to tackle invasive alien species in Belgium–the role of the ISEIA protocol and the Harmonia information system as decision support tools. Management of Biological Invasions 6, 197–208.

[brv70124-bib-0744] Vanderhoeven, S. , Branquart, E. , Casaer, J. , D'hondt, B. , Hulme, P. E. , Shwartz, A. , Strubbe, D. , Turbé, A. , Verreycken, H. & Adriaens, T. (2017). Beyond protocols: improving the reliability of expert‐based risk analysis underpinning invasive species policies. Biological Invasions 19, 2507–2517.

[brv70124-bib-0745] Vanderhoeven, S. , Dassonville, N. & Meerts, P. (2005). Increased topsoil mineral nutrient concentrations under exotic invasive plants in Belgium. Plant and Soil 275, 169–179.

[brv70124-bib-0746] Vaz, A. S. , Kueffer, C. , Kull, C. A. , Richardson, D. M. , Schindler, S. , Muñoz‐Pajares, A. J. , Vicente, J. R. , Martins, J. , Hui, C. & Kühn, I. (2017). The progress of interdisciplinarity in invasion science. Ambio 46, 428–442. 28150137 10.1007/s13280-017-0897-7 PMC5385671

[brv70124-bib-0747] Verhoeven, K. J. F. , Biere, A. , Harvey, J. A. & Van Der Putten, W. H. (2009). Plant invaders and their novel natural enemies: who is naive? Ecology Letters 12, 107–117. 19143824 10.1111/j.1461-0248.2008.01248.x

[brv70124-bib-0748] Vilà, M. , Bartomeus, I. , Dietzsch, A. C. , Petanidou, T. , Steffan‐Dewenter, I. , Stout, J. C. & Tscheulin, T. (2009). Invasive plant integration into native plant–pollinator networks across Europe. Proceedings of the Royal Society B: Biological Sciences 276, 3887–3893. 10.1098/rspb.2009.1076 PMC2817287 19692403

[brv70124-bib-0749] Vilà, M. , Gallardo, B. , Preda, C. , García‐Berthou, E. , Essl, F. , Kenis, M. , Roy, H. E. & González‐Moreno, P. (2019). A review of impact assessment protocols of non‐native plants. Biological Invasions 21, 709–723.

[brv70124-bib-0750] Vilà, M. , Trillo, A. , Castro‐Díez, P. , Gallardo, B. & Bacher, S. (2024). Field studies of the ecological impacts of invasive plants in Europe. NeoBiota 90, 139–159.

[brv70124-bib-0751] Vilizzi, L. , Copp, G. H. , Adamovich, B. , Almeida, D. , Chan, J. , Davison, P. I. , Dembski, S. , Ekmekçi, F. G. , Ferincz, Á. , Forneck, S. C. , Hill, J. E. , Kim, J.‐E. , Koutsikos, N. , Leuven, R. S. E. W. , Luna, S. A. , *et al*. (2019). A global review and meta‐analysis of applications of the freshwater fish invasiveness screening kit. Reviews in Fish Biology and Fisheries 29, 529–568.

[brv70124-bib-0752] Vilizzi, L. , Copp, G. H. , Hill, J. E. , Adamovich, B. , Aislabie, L. , Akin, D. , Al‐Faisal, A. J. , Almeida, D. , Azmai, M. N. A. , Bakiu, R. , Bellati, A. , Bernier, R. , Bies, J. M. , Bilge, G. , Branco, P. , *et al*. (2021). A global‐scale screening of non‐native aquatic organisms to identify potentially invasive species under current and future climate conditions. Science of the Total Environment 788, 147868. 34134389 10.1016/j.scitotenv.2021.147868

[brv70124-bib-0753] Vilizzi, L. , Hill, J. E. , Piria, M. & Copp, G. H. (2022 *a*). A protocol for screening potentially invasive non‐native species using weed risk assessment‐type decision‐support tools. Science of the Total Environment 832, 154966. 35367540 10.1016/j.scitotenv.2022.154966

[brv70124-bib-0754] Vilizzi, L. , Piria, M. , Herczeg, G. , Almeida, D. , Al‐Wazzan, Z. , Bakiu, R. , Boggero, A. , Chaichana, R. , Dashinov, D. , De Zoysa, M. , Gilles, A. S. J. , Goulletquer, P. , Interesova, E. , Kopecký, O. , Koutsikos, N. , *et al*. (2025). Questionnaire improvements in second‐generation, multilingual decision support tools for invasion risk screening of non‐native taxa. Management of Biological Invasions 16, 33–44.

[brv70124-bib-0755] Vilizzi, L. , Piria, M. , Pietraszewski, D. , Giannetto, D. , Flory, S. L. , Herczeg, G. , Sermenli, H. B. , Britvec, M. , Jukoniene, I. , Petrulaitis, L. , Vitasović‐Kosić, I. , Almeida, D. , Al‐Wazzan, Z. , Bakiu, R. , Boggero, A. , *et al*. (2024). Development and application of a second‐generation multilingual tool for invasion risk screening of non‐native terrestrial plants. Science of the Total Environment 917, 170475. 38296092 10.1016/j.scitotenv.2024.170475

[brv70124-bib-0756] Vilizzi, L. , Piria, M. , Pietraszewski, D. , Kopecky, O. , Spelic, I. , Radocaj, T. , Sprem, N. , Ta, K. , Tarkan, A. , Weiperth, A. , Yoğurtçuoğlu, B. , Candan, O. , Herczeg, G. , Killi, N. , Lemić, D. , *et al*. (2022 *b*). Development and application of a multilingual electronic decision‐support tool for risk screening non‐native terrestrial animals under current and future climate conditions. NeoBiota 76, 211–236.

[brv70124-bib-0757] Villéger, S. , Blanchet, S. , Beauchard, O. , Oberdorff, T. & Brosse, S. (2011). Homogenization patterns of the world's freshwater fish faunas. Proceedings of the National Academy of Sciences 108, 18003–18008. 10.1073/pnas.1107614108 PMC3207649 22025692

[brv70124-bib-0758] Vimercati, G. , Kumschick, S. , Probert, A. F. , Volery, L. & Bacher, S. (2020). The importance of assessing positive and beneficial impacts of alien species. NeoBiota 62, 525–545.

[brv70124-bib-0759] Vimercati, G. , Probert, A. F. , Volery, L. , Bernardo‐Madrid, R. , Bertolino, S. , Céspedes, V. , Essl, F. , Evans, T. , Gallardo, B. , Gallien, L. , González‐Moreno, P. , Grange, M. C. , Hui, C. , Jeschke, J. M. , Katsanevakis, S. , *et al*. (2022). The EICAT+ framework enables classification of positive impacts of alien taxa on native biodiversity. PLoS Biology 20, e3001729. 35972940 10.1371/journal.pbio.3001729 PMC9380921

[brv70124-bib-0760] Vitousek, P. M. (1990). Biological invasions and ecosystem processes: towards an integration of population biology and ecosystem studies. Oikos 57, 7–13.

[brv70124-bib-0761] Vitousek, P. M. , d'Antonio, C. M. , Loope, L. L. & Westbrooks, R. (1996). Biological invasions as global environmental change. American Scientist 84, 468–478.

[brv70124-bib-0762] Volery, L. , Blackburn, T. M. , Bertolino, S. , Evans, T. , Genovesi, P. , Kumschick, S. , Roy, H. E. , Smith, K. G. & Bacher, S. (2020). Improving the environmental impact classification for alien taxa (EICAT): a summary of revisions to the framework and guidelines. NeoBiota 62, 547–567.

[brv70124-bib-0763] * Voglmayr, H. , Schertler, A. , Essl, F. & Krisai‐Greilhuber, I. (2023). Alien and cryptogenic fungi and oomycetes in Austria: an annotated checklist. Biological Invasions 25, 27–38. 36643959 10.1007/s10530-022-02896-2 PMC9832105

[brv70124-bib-0764] Walsh, J. R. , Carpenter, S. R. & Vander Zanden, M. J. (2016). Invasive species triggers a massive loss of ecosystem services through a trophic cascade. Proceedings of the National Academy of Sciences 113, 4081–4085. 10.1073/pnas.1600366113 PMC4839401 27001838

[brv70124-bib-0765] Wanzenböck, J. , Hopfinger, M. , Wanzenböck, S. , Fuxjäger, L. , Rund, H. & Lamatsch, D. K. (2021). First successful hybridization experiment between native European weatherfish (*Misgurnus fossilis*) and non‐native oriental weatherfish (*M. Anguillicaudatus*) reveals no evidence for postzygotic barriers. NeoBiota 69, 29–50.

[brv70124-bib-0766] Warren, R. J. , King, J. R. , Tarsa, C. , Haas, B. & Henderson, J. (2017). A systematic review of context bias in invasion biology. PLoS One 12, e0182502. 28817593 10.1371/journal.pone.0182502 PMC5560718

[brv70124-bib-0767] Waser, N. M. & Price, M. V. (1994). Crossing‐distance effects in *Delphinium nelsonii*: outbreeding and inbreeding depression in progeny fitness. Evolution 48, 842–852. 28568280 10.1111/j.1558-5646.1994.tb01366.x

[brv70124-bib-0768] Watkins, H. V. , Yan, H. F. , Dunic, J. C. & Côté, I. M. (2021). Research biases create overrepresented “poster children” of marine invasion ecology. Conservation Letters 14, e12802.

[brv70124-bib-0769] Wattier, R. A. , Haine, E. R. , Beguet, J. , Martin, G. , Bollache, L. , Muskó, I. B. , Platvoet, D. & Rigaud, T. (2007). No genetic bottleneck or associated microparasite loss in invasive populations of a freshwater amphipod. Oikos 116, 1941–1953.

[brv70124-bib-0770] Wayne, R. K. & Shaffer, H. B. (2016). Hybridization and endangered species protection in the molecular era. Molecular Ecology 25, 2680–2689. 27064931 10.1111/mec.13642

[brv70124-bib-0771] * Weir, J. L. , Daniel, W. , Hyder, K. , Skov, C. & Venturelli, P. A. (2024). Artificial intelligence applied to big data reveals that lake invasions are predicted by human traffic and co‐occurring invasions. Biological Invasions 26, 3163–3178.

[brv70124-bib-0772] White, W. B. & Schneeberger, N. F. (1981). Socioeconomic impacts. In The Gpsy Moth: Research Towards Integrated Pest Management, Forest Service Technical Bulletin 1584 (), pp. 681–694. U.S. Department of Agriculture, Washington D.C.

[brv70124-bib-0773] * White, R. L. , Strubbe, D. , Dallimer, M. , Davies, Z. G. , Davis, A. J. S. , Edelaar, P. , Groombridge, J. , Jackson, H. A. , Menchetti, M. & Mori, E. (2019). Assessing the ecological and societal impacts of alien parrots in Europe using a transparent and inclusive evidence‐mapping scheme. NeoBiota 48, 45–69.

[brv70124-bib-0774] Wightman, J. A. (1979). Energetics as an approach to estimating the economic impact of pasture pests. New Zealand Journal of Zoology 6, 509–517.

[brv70124-bib-0775] Wilcox, M. A. , Johnson, D. , Dyke, K. , Gunsch, D. , Lyons, D. A. , Dibacco, C. & Therriault, T. W. (2025). Identifying higher risk invaders to the Columbia glaciated freshwater ecoregion using a new screening tool: the non‐indigenous species screening tool (NISST). Management of Biological Invasions 16, 187–210.

[brv70124-bib-0776] Williams, S. L. & Smith, J. E. (2007). A global review of the distribution, taxonomy, and impacts of introduced seaweeds. Annual Review of Ecology, Evolution, and Systematics 38, 327–359.

[brv70124-bib-0777] Wilson, J. R. U. , Procheş, Ş. , Braschler, B. , Dixon, E. S. & Richardson, D. M. (2007). The (bio) diversity of science reflects the interests of society. Frontiers in Ecology and the Environment 5, 409–414.

[brv70124-bib-0778] Winter, M. , Devictor, V. & Schweiger, O. (2013). Phylogenetic diversity and nature conservation: where are we? Trends in Ecology & Evolution 28, 199–204. 23218499 10.1016/j.tree.2012.10.015

[brv70124-bib-0779] Woch, M. W. , Kapusta, P. , Stanek, M. , Możdżeń, K. , Grześ, I. M. , Rożej‐Pabijan, E. & Stefanowicz, A. M. (2023). Effects of invasive Rosa rugosa on Baltic coastal dune communities depend on dune age. NeoBiota 82, 163–187.

[brv70124-bib-0780] Wolf, D. E. , Takebayashi, N. & Rieseberg, L. H. (2001). Predicting the risk of extinction through hybridization. Conservation Biology 15, 1039–1053.

[brv70124-bib-0781] Wong, M. K. L. (2024). Misrepresentation of invasive species in the mass media with images of unrelated organisms. Conservation Biology 38, e14382. 39286930 10.1111/cobi.14382 PMC11589081

[brv70124-bib-0782] Wood, L. E. , Clinton, M. , Bass, D. , Bojko, J. , Foster, R. , Guilder, J. , Kennerley, A. , Peeler, E. , Waine, A. & Tidbury, H. (2023). Parasite invasions and food security. In Parasites and Biological Invasions, pp. 141–158. CABI International, Wallingford, Oxfordshire, UK.

[brv70124-bib-0783] * Xing, J. , Jia, X. , Wang, H. , Ma, B. , Falcão Salles, J. & Xu, J. (2021). The legacy of bacterial invasions on soil native communities. Environmental Microbiology 23, 669–681. 32419297 10.1111/1462-2920.15086

[brv70124-bib-0784] Xu, Y. , Huang, J. , Zhou, A. & Zeng, L. (2012). Prevalence of *Solenopsis invicta* (Hymenoptera: Formicidae) venom allergic reactions in mainland China. Florida Entomologist 95, 961–965.

[brv70124-bib-0785] Yan, J. , Mackay, A. J. & Stone, C. M. (2024). Dynamics of invasive mosquitoes: introduction pathways, limiting factors, and their potential role in vector‐borne pathogen transmission. Frontiers in Tropical Diseases 5, 1503120.

[brv70124-bib-0786] Yang, T. , Wei, Z. , Friman, V. , Xu, Y. , Shen, Q. , Kowalchuk, G. A. & Jousset, A. (2017). Resource availability modulates biodiversity‐invasion relationships by altering competitive interactions. Environmental Microbiology 19, 2984–2991. 28229529 10.1111/1462-2920.13708

[brv70124-bib-0787] Yelenik, S. G. , Stock, W. D. & Richardson, D. M. (2007). Functional group identity does not predict invader impacts: differential effects of nitrogen‐fixing exotic plants on ecosystem function. Biological Invasions 9, 117–125.

[brv70124-bib-0788] Yeruham, E. , Rilov, G. , Shpigel, M. & Abelson, A. (2015). Collapse of the echinoid *Paracentrotus lividus* populations in the eastern Mediterranean—result of climate change? Scientific Reports 5, 13479. 26315893 10.1038/srep13479 PMC4551984

[brv70124-bib-0789] Yeruham, E. , Shpigel, M. , Abelson, A. & Rilov, G. (2020). Ocean warming and tropical invaders erode the performance of a key herbivore. Ecology 101, e02925. 31660585 10.1002/ecy.2925

[brv70124-bib-0790] Yokomizo, H. , Possingham, H. P. , Thomas, M. B. & Buckley, Y. M. (2009). Managing the impact of invasive species: the value of knowing the density–impact curve. Ecological Applications 19, 376–386. 19323196 10.1890/08-0442.1

[brv70124-bib-0791] Zavaleta, E. S. , Hobbs, R. J. & Mooney, H. A. (2001). Viewing invasive species removal in a whole‐ecosystem context. Trends in Ecology & Evolution 16, 454–459.

[brv70124-bib-0792] Zedler, J. B. & Kercher, S. (2004). Causes and consequences of invasive plants in wetlands: opportunities, opportunists, and outcomes. Critical Reviews in Plant Sciences 23, 431–452.

[brv70124-bib-0793] Zenni, R. D. , Lamy, J.‐B. , Lamarque, L. J. & Porté, A. J. (2014). Adaptive evolution and phenotypic plasticity during naturalization and spread of invasive species: implications for tree invasion biology. Biological Invasions 16, 635–644.

[brv70124-bib-0794] Zhang, H. , Liu, Z. , Chen, H. & Tang, M. (2016). Symbiosis of arbuscular mycorrhizal fungi and *Robinia pseudoacacia* L. improves root tensile strength and soil aggregate stability. PLoS One 11, e0153378. 27064570 10.1371/journal.pone.0153378 PMC4827865

[brv70124-bib-0795] Zhang, L. & Wang, B. (2016). Intraspecific interactions shift from competitive to facilitative across a low to high disturbance gradient in a salt marsh. Plant Ecology 217, 959–967.

[brv70124-bib-0796] Zhang, R. , Li, Y. , Liu, N. & Porter, S. D. (2007). An overview of the red imported fire ant (hymenoptera: Formicidae) in mainland China. Florida Entomologist 90, 723–731.

[brv70124-bib-0797] Zmora, N. , Suez, J. & Elinav, E. (2019). You are what you eat: diet, health and the gut microbiota. Nature Reviews Gastroenterology & Hepatology 16, 35–56. 30262901 10.1038/s41575-018-0061-2

