# Supplementary material for: The impacts of biological invasions
Source: Biol Rev Camb Philos Soc. 2025 Dec 30;101(3):1255–310. doi: 10.1002/brv.70124 (PMC13149820; doi:10.1002/brv.70124)
Supplement: Supplementary file 1 — Appendix S1. Conducted literature search. [file BRV-101-1255-s005.docx]

**Appendix S1. Conducted literature search**

We conducted a scoping literature search using the *Web of Science* (WoS) *Core Collection* to retrieve peer-reviewed publications, book chapters, and relevant literature on the impacts of non-native species using the following combination of key words and Boolean characters (impact AND invasive OR non-native OR nonnative OR alien OR exotic OR non-indigenous). This search resulted in 196,291 documents that, after removing WoS categories and research areas not relevant to our field (e.g. Endocrinology, Neurosciences or Construction & Building Technology) retrieved 23,599 publications between 1945 and 2025. We grouped the retrieved documents by publication year and calculated the number of publications per year to examine the temporal trend. To identify if there is an increasing rate in the number of publications on the topic of impacts of non-native species over time, we divided the number of papers on invasion impacts per year (total 23,599), by the total number of publications published in each year across all scientific fields (Fig. 1) or across the field of Ecology only (Fig. S1).
