# Supplementary material for: The impacts of biological invasions
Source: Biol Rev Camb Philos Soc. 2025 Dec 30;101(3):1255–310. doi: 10.1002/brv.70124 (PMC13149820; doi:10.1002/brv.70124)
Supplement: Supplementary file 2 — Appendix S2. The need for a standardised terminological framework. [file BRV-101-1255-s002.docx]

**Appendix S2. The need for a standardised terminological framework**

Inconsistent terminology has burdened invasion science for decades. From a scientific viewpoint ‘spread’ is the defining criterion for a species ‘invasiveness’ (Ricciardi & Cohen, 2007; Soto *et al.*, 2024*a*), but policy and management often rely the classification of a non-native species as ‘invasive’ on impacts (Roy *et al.*, 2019, 2023*b*). Conversely, other authors argue that there are compelling conceptual and practical reasons for ‘impact’ to be a defining criterion (Watkins *et al.*, 2021). Those in favour of this argue that the term ‘invasive’ inherently implies significant ecological change, making it more intuitive to classify non-native species that demonstrably affect ecosystems (Roy *et al.*, 2023*a*). From a practical standpoint, evidence of a negative impact often triggers conservation actions, helping to prioritise management efforts in resource-limited scenarios (Blackburn *et al.*, 2014), but reported ecological impacts have been designed using single-species studies, creating biased results (Guerin *et al.*, 2018). Because of incomplete data on non-native species impacts, it is often necessary to consider both the actual (including elsewhere) and potential impacts, in some cases documented by anecdotal data only. However, the complexity of collecting evidence of impacts differs among taxa, being more straightforward for some (e.g. sessile organisms, mammals, plants) and less so for others [e.g. mobile and aquatic organisms, fungi and microbes (Thomsen *et al.*, 2014*b*; Kumschick *et al.*, 2015)]. Yet defining invasiveness solely through impact assessment presents significant challenges for policy and management. If impact were the only criterion for classifying a species as invasive, then management interventions would be postponed until sufficient evidence of an actual harm was gathered in a specific location, and would ultimately result in inaction, allowing species to spread unchecked (Soto *et al.*, 2024*a*). Such an approach fails to consider the broader ecological and long-term consequences of species introductions, particularly in light of shifting environmental conditions that may eventually amplify current and future impacts (Spear *et al.*, 2021). Moreover, species that spread rapidly but lack well-documented immediate impacts often receive less attention than species with known but localised effects, even though the former could cause substantial long-term harm. This discrepancy is evident in the case of emerging invaders, such as the western waterweed (*Elodea nuttallii*) or the Brazilian pondweed (*Egeria densa*) in Europe (Santos, Anderson & Ustin, 2011; Kelly *et al.*, 2015). Although both species spread aggressively, in some cases they have not yet been classified as exerting severe ecological, economic, or socio-cultural impacts, leading to delayed management responses. Other species with well-documented negative effects and higher management feasibility, such as the Eastern grey squirrel, tend to be prioritised for control, even when their spread potential is lower in certain regions (Di Febbraro *et al.*, 2013).
