# Supplementary material for: The impacts of biological invasions
Source: Biol Rev Camb Philos Soc. 2025 Dec 30;101(3):1255–310. doi: 10.1002/brv.70124 (PMC13149820; doi:10.1002/brv.70124)
Supplement: Supplementary file 3 — Appendix S3. Directions for future research. [file BRV-101-1255-s006.docx]

**Appendix S3. Directions for future research**

Despite major advancements in recent years (Everts *et al.*, 2024; Vilà *et al.*, 2024), non-native species risk analysis and impact assessment remain an evolving frontier. A critical challenge lies in expanding the existing scope of attempts beyond traditional ecological and economic metrics to incorporate under-represented domains such as socio-cultural impacts, non-market costs, and the influence of so far rarely considered or critically understudied organisms (from an invasion science perspective) such as non-native microbes, fungi, or, among others, viruses or unsuccessful invasions (Litchman, 2010; Mallon *et al.*, 2018). For example, microbial invasions that mediate nutrient cycling may significantly alter ecosystem function, yet their impacts remain largely speculative and overlooked by assessment tools​ due to data limitations, taxonomic uncertainties, and functional redundancy (Xing *et al.*, 2021; Santini & Migliorini, 2022; Voglmayr *et al.*, 2023). Effective assessments depend on harmonisation between different frameworks. Discrepancies in methodologies can lead to inconsistencies in species rankings, complicating policy decisions. Standardising species’ risk rankings and assessment criteria and improving cross-framework comparability are essential for enhancing the reliability of risk evaluations and ensuring their applicability across different conservation contexts. Existing screening tools frequently employ rigid scoring systems that attempt to quantify impacts in a standardised manner. These tools rely on the knowledge of the assessors and extent of available data on non-native impacts, which is affected by several biases (Marcot *et al.*, 2019) and could skew the assessment to favour the ‘poster children’ of biological invasions over others that are potentially more damaging (Watkins *et al.*, 2021). Advancements in risk screening and assessment methodologies should emphasise improving standardisation across frameworks refining methods for uncertainty quantification (Wilcox *et al.*, 2025) and fostering greater compatibility between different assessment approaches (Boggero *et al.*, 2025). Refining these risk analysis and impact assessment tools will be essential for accurately identifying high-risk species, guiding effective management strategies, and minimising both ecological and economic harm. The ENSARS and the Modular Management Tool are currently only available in a rudimentary spreadsheet format, which limits their deployability, ease of use, and, ultimately, adoption. Strengthening risk assessments will therefore play a critical role in ensuring proactive and evidence-based decision-making for biodiversity conservation.

In terms of methodology, efforts are under way to refine and harmonise impact assessment frameworks. Projects such as *InvaCost* (Diagne *et al.*, 2020) represent the next generation of standardised information systems. By leveraging large language models to extract data automatically from a large number of articles (Reynolds *et al.*, 2025; Keck, Broadbent & Altermatt, 2025*a*), these tools aim to reduce subjectivity in impact scoring, improve reproducibility, and enable more robust meta-analyses. However, major gaps remain in global data coverage, particularly for the Global South, freshwater and marine systems, and socio-cultural contexts (Bacher *et al.*, 2023b). Global data mobilisation, including citizen science, local ecological knowledge, and historical records, are therefore urgently required and should be prioritised to achieve a more balanced understanding of invasion impacts.

Emerging technologies offer transformative potential: Artificial Intelligence and big data analytics are increasingly being applied to detect, classify, and predict invasion dynamics and their associated impacts (Shen *et al.*, 2024; Weir *et al.*, 2024; Fenollosa & Salguero-Gomez, 2025; Reynolds *et al.*, 2025). Foundation Models (large-scale AI systems pre-trained on diverse data) offer promising new tools for predicting and monitoring biological invasions (Guo *et al.*, 2025; Reynolds *et al.*, 2025). By generating embeddings that capture complex similarities between concepts, these models can integrate multimodal data such as species records, satellite imagery, climate variables, and global transport routes to forecast invasion risks across taxa and regions (Murphy, Grenouillet & García-Berthou, 2015; Bae, Murphy & García-Berthou, 2018). Despite current challenges in interpretability and training efficiency (Morera, 2024), their ability to generalise across tasks and domains makes them a compelling alternative to traditional species distribution models. For instance, a Foundation Model could combine habitat descriptions, climate projections, and image-based species traits to identify emerging invasion risks in poorly monitored regions. These models can also be continuously updated as new data become available, making them dynamic and responsive tools for early warning systems. Geospatial Foundation Models are a particularly promising tool for non-native species modelling due to their focus on embeddings of geospatial and temporal data. Models such as Clay (<https://madewithclay.org/>), Prithvi (Jakubik *et al.*, 2023), and [SkySense](https://arxiv.org/abs/2312.10115)(Guo *et al.*, 2024) derive geo-spatiotemporal embeddings from large Earth Observation data sets and have already shown promise in tasks such as deforestation mapping, land use classification and satellite image segmentation.

Another recent research direction is the use of machine learning to aid the development of conservation management strategies. These methods involve models that integrate geospatial, climate, species distribution and other data to simulate the distribution of species and vegetation types under different land use and conservation planning scenarios. Reinforcement Learning algorithms can then be used to learn a management policy that attains a specified quantifiable objective (such as minimising the number of individuals from a non-native species) under constraints such as cost or the amount of land that can be protected. Dietterich, Taleghan & Crowley (2013) demonstrated how Reinforcement Learning can be applied to managing a river system to control the expansion of a non-native plant species. Lapeyrolerie *et al*. (2022) demonstrated the potential of Reinforcement Learning for resource management problems, such as setting fishing quotas. Conservation area prioritisation through artificial intelligence (CAPTAIN) (Silvestro *et al.*, 2022) is a general simulation framework that allows the user to define flexibly management objectives to be optimised. The unprecedented rate at which new technologies are emerging holds great promise for improving impact assessments related to biological invasions.
