# Supplementary material for: The impacts of biological invasions
Source: Biol Rev Camb Philos Soc. 2025 Dec 30;101(3):1255–310. doi: 10.1002/brv.70124 (PMC13149820; doi:10.1002/brv.70124)
Supplement: Supplementary file 4 — Appendix S4. Criticism of so‐called ‘black lists’. [file BRV-101-1255-s001.docx]

**Appendix S4. Criticism of so-called ‘black lists’**

The use of black lists to regulate species introductions is one of the most familiar and politically acceptable tools to put into practice the precautionary approach required to manage non-native species. A key limitation of black lists, however, is that restrictions on one species may stimulate the trade and consequently trigger the escape or release of alternative, potentially invasive non-native species (Hulme, 2015). For instance, in Portugal, after *Trachemys scripta* was listed under EU Regulation 1143/2014 and its trade banned, pet shops began selling other freshwater turtles such as river cooter (*Pseudemys concinna*) and false map turtle (*Graptemys pseudogeographica*), which subsequently became widespread in the pet trade and were released into the wild (Banha, Diniz & Anastácio, 2019). In response, national Portuguese legislation extended the ban to additional turtle genera. Yet this, too, was followed by the emergence of new species in the market, such as Chinese stripe-necked turtle (*Mauremys sinensis*), illustrating how targeted bans can lead to unintended substitution effects (Rato *et al.*, 2024). Similarly, following the European Union’s ban on wild-caught birds in 2005 due to avian influenza concerns, which was made permanent in 2007 for conservation reasons, the international pet trade for monk parakeets shifted from European to Mexican markets. In 2008, the Mexican government enacted regulations prohibiting the trade of native parrots to protect them from illegal overharvesting. As a result, demand for non-native parrots surged, with monk parakeets from Uruguay becoming the primary species imported. This influx led to a significant increase in sightings of feral monk parakeets across Mexico (Hobson, Smith-Vidaurre & Salinas-Melgoza, 2017). While the EU’s bird ban effectively reduced invasion levels within EU countries (Cardador *et al.*, 2019), it also triggered unintended consequences, potentially exacerbating ecological impacts in other regions (Reino *et al.*, 2017). For this reason, an alternative approach may be the development of a ‘positive list’, which is a tool aimed at listing species that are considered to pose a low risk, and that could be allowed to be kept and/or traded without harm to biodiversity, environment, etc. (Toland *et al.*, 2020). This approach may be more effective, and there were suggestions to amend the current EU Regulation No. 1143/2014 by creating a whitelist that would allow certain species to enter the EU while banning all others. In the meantime, this approach is being progressively adopted by some countries.

Another critique is that the effectiveness of black lists depends heavily on accurately identifying species with the potential to become invasive, and especially their implementation (Patoka *et al.*, 2018). Relying heavily on predefined lists of (potentially) invasive species can introduce biases in decision-making, where species that have not yet been formally assessed or whose impacts remain unknown are overlooked (Cuthbert *et al.*, 2022), potentially allowing for the establishment of future problematic invaders while focusing resources on already invasive species. Enforcing black lists can also be challenging, as species can still enter unintentionally (e.g. *via* hitchhiking) or intentionally (e.g. *via* illegal smuggling). For example, Coughlan *et al*. (2020) questioned the effectiveness of the Union list, particularly for aquatic invasive species. They noted that the Regulation prioritises deliberately traded species, such as pets or ornamentals, while unintentionally introduced species, e.g. *via* boating, fishing gear, or biofouling, remain insufficiently addressed. The authors proposed that these could be more effectively managed through systematic decontamination measures. Nevertheless, the Regulation also provides for the mandatory implementation of ‘pathways action plans’ to address unintentional introductions of listed species (Article 13, EU 1143/2014). Furthermore, blacklists are critiqued for placing a strong enforcement emphasis on penalties, which may detract from the importance of complementary strategies such as education, public outreach, and proactive prevention measures aimed at reducing introduction risks in the first place (Patoka *et al.*, 2018). They are also critiqued for their limited adaptability to the dynamic nature of biological invasions, which are driven by climate change, shifting trade routes, and changing ecosystems, as the bureaucratic hurdles involved in updating such lists, including the EU list, can hinder timely responses to emerging threats (Caffrey *et al.*, 2014). In addition, they raise ethical and conservation concerns particularly when proposed eradication measures involve non-native species that have become culturally significant or are valued for economic reasons. These issues mostly appear when large, charismatic and empathetic species are targeted (Estévez *et al.*, 2015; De Groot *et al.*, 2020; Lipták *et al.*, 2023). For example, the monk parakeet is considered invasive and potentially harmful in parts of Europe and the USA due to its impact on infrastructure and agriculture (White *et al.*, 2019), yet in some areas, public opposition to culling efforts has hindered management actions (Crowley, Hinchliffe & McDonald, 2019). The Eastern grey squirrel rapidly colonised natural landscapes, transfers infection causing epizooty in the related Eurasian red squirrel and damages trees by stripping, but lethal management actions are complicated by public concern as the species is loved by part of the citizenry (De Groot *et al.*, 2020).
