# Supplementary material for: The impacts of biological invasions
Source: Biol Rev Camb Philos Soc. 2025 Dec 30;101(3):1255–310. doi: 10.1002/brv.70124 (PMC13149820; doi:10.1002/brv.70124)
Supplement: Supplementary file 5 — Appendix S5. Conflicting goals in environmental governance. [file BRV-101-1255-s003.docx]

**Appendix S5. Conflicting goals in environmental governance**

Developing policies to manage biological invasions presents unique challenges. In Europe, the impacts of non-native species are compounded by difficulties in reducing these impacts and the complexity of policy options under consideration (Keller *et al.*, 2011). The unique nature of non-native species requires tailored policy responses, and implementing effective management programmes necessitates careful design and execution addressing the impact of non-native species (Barney & Tekiela, 2020; García-Díaz *et al.*, 2021).

​The management of biological invasions also intersects with other environmental governance goals, such as animal protection. Animals deemed non-native are part of biodiversity, yet their management can conflict with conservation goals (Bliss, Visseren-Hamakers & Liefferink, 2023). This duality poses challenges for global environmental politics, as governance systems strive to balance biodiversity conservation with animal protection. Conflicts often arise due to differing public perceptions and values associated with non-native species management. Understanding these social dimensions is crucial for developing effective management strategies that are both scientifically sound and publicly acceptable (Estévez *et al.*, 2015). ​To address these challenges, it is essential to anticipate disagreements, consider the social-ecological contexts of management, adopt inclusive engagement mechanisms, and foster open, responsive communication. Such approaches can transform conflicts in non-native species management into constructive dialogues, facilitating more effective and ethically sound outcomes (Crowley, Hinchliffe & McDonald, 2017*a*). ​A focus on the known impacts of non-native species often misguides political actions, as is common in present-day policymaking, leading to a disproportionate emphasis on species that have visible, immediate effects, while neglecting those that may spread undetected but still pose long-term risks (Leung *et al.*, 2002). Political actions often prioritise species with well-documented impacts, advancing them on species lists, while species without clear known impacts may be overlooked, even if their potential for future spread remains high (Cuthbert *et al.*, 2022; Garcia‐Lozano *et al.*, 2025) This approach can lead to reactive rather than proactive management, where the focus shifts from preventing invasions to responding to their consequences. Consequently, effective management requires a more balanced approach that considers both current impacts and the potential for future spread.

Addressing biological invasions therefore necessitates a comprehensive understanding that transcends local impacts, as non-native species often pose challenges that span regional and global contexts. Focusing solely on immediate local effects can lead to policy actions that are misaligned with the broader, more complex realities of biological invasions (Garcia‐Lozano *et al.*, 2025). Many non-native species cannot be eradicated and require sustained management efforts. However, policies often lack the necessary long-term strategies, focusing instead on short-term, localised interventions that fail to address the ongoing nature of invasions (García-Díaz *et al.*, 2021). Existing legislation to manage non-native species is often fragmented and lacks international coherence, leading to inconsistent enforcement and gaps in coverage. This patchwork approach hampers effective management and control efforts. Overall, policies should consider the full spectrum of impacts – environmental, economic, and social – associated with non-native species, recognising that local issues are part of larger, interconnected systems.​
