# Supplementary material for: The impacts of biological invasions
Source: Biol Rev Camb Philos Soc. 2025 Dec 30;101(3):1255–310. doi: 10.1002/brv.70124 (PMC13149820; doi:10.1002/brv.70124)

**Fig. S1.** Annual number of publications on the impact of non-native species (A) and against all publications listed in the field of Ecology in the *Web of Science* (B), highlighting the rise of invasion biology in the context of overall science output. Details on data extraction are provided in Appendix S1.


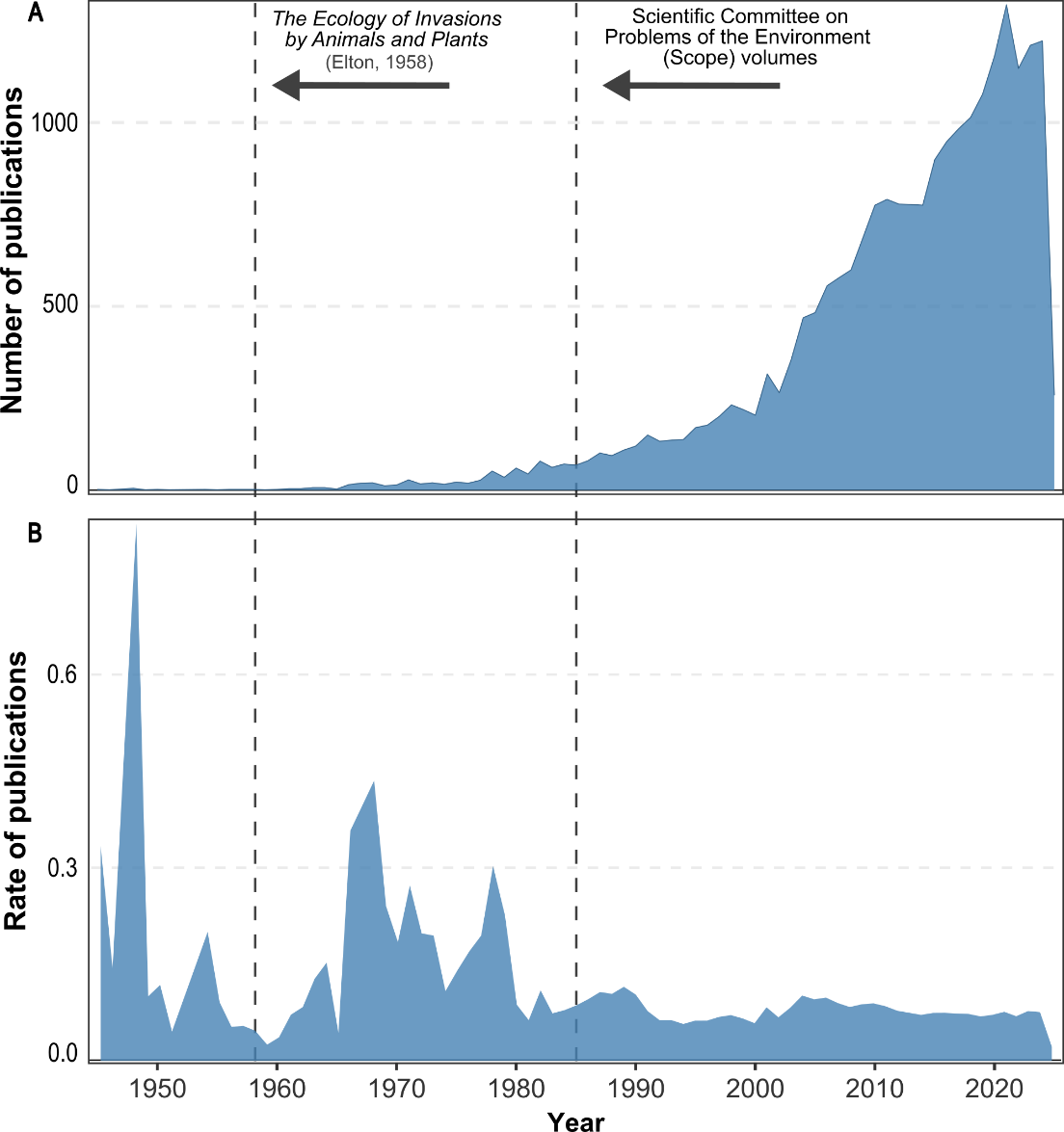

Supplement: Supplementary file 6 — Fig. S1. Annual number of publications on the impact of non‐native species and as a proportion of all publications listed in the field of Ecology in the Web of Science, highlighting the rise of invasion biology in the context of overall science output. [file BRV-101-1255-s004.docx]
